# Revision and Phylogenetic Analysis of the Genus *Phonoctonus* Stål, 1853 (Heteroptera, Reduviidae, Harpactorinae)

**DOI:** 10.3390/insects12121100

**Published:** 2021-12-08

**Authors:** Agnieszka Bugaj-Nawrocka, Dominik Chłond

**Affiliations:** Institute of Biology, Biotechnology and Environmental Protection, Faculty of Natural Sciences, University of Silesia in Katowice, Bankowa 9, 40-007 Katowice, Poland; agnieszka.bugaj-nawrocka@us.edu.pl

**Keywords:** Africa, assassin bugs, mimetism, natural enemies

## Abstract

**Simple Summary:**

Taxonomic revisions, especially in the case of genera where the number of newly described species has increased over the years, are essential. The example of the genus *Phonoctonus* discussed in this paper shows that there are problematic taxa that, if described separately, without analysis of comparative material, may lead to unnecessary synonyms. Alternatively, individuals that exhibit features that qualify them as representatives of a new species are described as colour variants or varieties. The results of our research are a perfect example of this. Over the years, many colour varieties have been described, some of which have been proven to be the ‘correct’ species. Others are simply individuals of the same species that have a wide variation in colouration. These details should be considered, especially since we know from previous studies that representatives of the genus *Phonoctonus* show aposematic colouration to other true bugs they hunt.

**Abstract:**

This paper presents a taxonomic revision and phylogenetic analysis of nine known species of the genus *Phonoctonus* Stål, 1853, distributed exclusively in the Afrotropical region. The revision and phylogenetic analysis were performed using morphological data. A full redescription for all species is provided, taxonomical problems are clarified, and diagnostic characters are presented and illustrated. Based on the analysis, thirteen species are recognised as valid: *Phonoctonus bifasciatus* stat. nov., *P*. *caesar*, *P*. *elegans*, *P*. *fairmairei* stat. nov., *P*. *fasciatus*, *P*. *grandis*, *P*. *immitis* stat. rev., *P*. *luridus*, *P*. *lutescens*, *P*. *nigrofasciatus* stat. rev., *P*. *picta* stat. nov., *P*. *picturatus* stat. rev., and *P*. *principalis*. New synonymies are proposed: *Phonoctonus nigrofasciatus* *= P. fasciatus* var. *poultoni* syn. nov., *P. picturatus* = *P. fasciatus* var. *discalis* syn. nov., and *P. principalis* = *P. validus* syn. nov. An identification key to separate the species, drawings of dorsal habitus, and distribution maps of all valid species are presented. Performed morphological phylogenetic analyses indicate monophyly of the genus *Phonoctonus*.

## 1. Introduction

Assassin bugs (Heteroptera: Reduviidae) are the most diverse Heteroptera family both in terms of biology and morphology. Reduviidae comprise almost 7000 described species, distributed in almost all zoogeographical regions. However, the greatest diversity has been recorded in tropical parts worldwide [1,2,3,4].

Harpactorinae is the largest reduviid subfamily (about 2000 described species), consisting predominantly of generalist predators [2,5,6,7,8,9], but among them, there are also taxa with high prey specificity such as the genus *Phonoctonus* Stål, 1853 [10,11,12]. Representatives of the genus *Phonoctonus* are of great economic importance as natural enemies of the crop pest species of the genus *Dysdercus* Guérin-Méneville, 1831 [13] and *Probergrothius* Kirkaldy, 1904 [14] (formerly known as *Odontopus* Laporte, 1833 [15]) (Heteroptera: Pyrrhocoridae). Their role in the biological control of these pests is related to their mimetic colouration. Individuals belonging to different species of the genus *Phonoctonus* have colour patterns very similar to the individual species of the genus *Dysdercus* or *Probergrothius* [11,12,16,17,18,19,20,21,22]. On the other hand, parasitoid individuals of wasp species *Anastatus phonoctoni* Risbec, 1955 [23] (Hymenoptera: Eupelmidae) were obtained from eggs of *P. lutescens* [24].

The genus *Phonoctonus* was established by Stål [10] based on *Reduvius fasciatus* Beauvois, 1805 [25]. Since the proposal of the genus in the mid-nineteenth century, it has been studied so far in terms of biology—mimetism, food preferences, and sexual behaviours [12,17,18,26]. The only detailed taxonomic descriptions concerning the three *Phonoctonus* species’ nymphal stages were carried out by Stride [17,18]. The present contribution offers a taxonomic revision and phylogenetic study of *Phonoctonus*, with a review of the morphological characteristics. Based on the analysis, thirteen species were recognised as valid. *Phonoctonus nigrofasciatus* Stål, 1855 [27] stat. rev. and *P. picturatus* Fairmaire, 1858 [28] stat. rev., were erroneously synonymised with *P. fasciatus* by previous authors, and are now recognised as distinct species. The following variations of *Phonoctonus fasciatus*: *P. fasciatus* var. *bifasciatus* Villiers, 1948 [11] stat. nov., *P. fasciatus* var. *fairmairei* Villiers, 1948 [11] stat. nov. and *P. fasciatus* var. *picta* Schouteden, 1932 [29] stat. nov., erroneously described as colour variations of mentioned species by previous authors, are recognised as distinct species. The following new synonymy is proposed: *Phonoctonus nigrofasciatus* Stål, 1855 = *P. fasciatus* var. *poultoni* Villiers, 1953 [30] syn. nov., *P. picturatus* Fairmaire, 1858 = *P. fasciatus* var. *discalis* Schouteden, 1932 [29] syn. nov., *Phonoctonus principalis* Gerstaecker, 1892 [31] = *P. validus* Horváth, 1892 [32] syn. nov. Neotypes are designated for *P. fasciatus* (Beauvois, 1805) and *P. lutescens* (Guérin-Méneville & Percheron, 1834) [33]. Lectotypes are designated for *P. caesar* Haglund, 1895 [34], *P. elegans* Varela, 1904 [35], *P. immitis* Stål, 1855 [27], *P. luridus* Miller, 1950 [36], *P. nigrofasciatus* Stål, 1855, and *P. validus* Horváth, 1892. Lectotype and paralectotypes have been designed for *P. grandis* Signoret, 1860 [37] and *P. principalis* Gerstaecker, 1892. All species are redescribed, and dorsal habitus and male and female genitalia drawings are provided. Images of selected morphological features obtained using light microscopy and scanning electron microscopy as well as distribution maps and identification keys are provided.

## 2. Material and Methods

### 2.1. The Structure of the Taxonomic Revision

The genus description provides the revised diagnosis and lists the morphological features (as redescription) that allow for the identification of any representative of the genus *Phonoctonus*. General information about the colouration and details of the structure and vestiture are given—this section is separated into the principal anatomical portions: head, thorax, hemelytra, legs and abdomen. Then, the key to identifying each species is provided. *Phonoctonus immitis* is listed twice in the key as it can be identified according to two criteria.

Descriptions of individual species consist of identifying possible synonyms and previous combinations, data specification on typical material, and other analysed specimens (available in the Supplementary Materials). Next, the diagnosis and redescription are given. Redescription is divided into the following sections: colouration; structure (both separating into following parts: head, thorax (with legs and hemelytra) and abdomen; with an additional part within the structure—genitalia); measurements; distribution; and comments.

### 2.2. Species Concept

The *Phonoctonus* species exhibit various colouration patterns, which are reflected, for example, in the many varieties of *P. fasciatus* proposed by different authors [11,28,29,30,38]. Despite being very similar concerning colour patterns, other constant, discrete characters help delimit different colour forms of species. Here, species concepts are based mainly on specific differences in the genitals, especially in males. Particular attention should be paid to the sizes and shapes of the pygophore, parameres, pedicel, endosomal struts of the aedeagus, and basal plate.

### 2.3. Entomological Collections

All type material was studied from specimens. The vast majority of the material was also studied based on specimens, and only a few were included in the materials based on photos provided by the curators of the collection.

The study was based on material deposited in the following museums: HNHM—Hungarian Natural History Museum, Budapest, Hungary; MHNG—Muséum d’Histoire Naturelle, Geneva, Switzerland; MMBC—Moravske Muzeum, Brno, Czech Republic; MNCN—Museo Nacional de Ciencias Naturales, Madrid, Spain; MNHN—Muséum National d’Histoire Naturelle, Paris, France; MSNM—Museo Civico di Storia Naturale, Milano, Italy; MZH—Finnish Museum of Natural History, Helsinki, Finland; NHMUK—The Natural History Museum, London, United Kingdom; NHMW—Naturhistorisches Museum Wien, Wien, Austria; NHRS—Naturhistoriska riksmuseet, Stockholm, Sweden; NMPC—National Museum (Natural History), Prague, Czech Republic; RBINS—Royal Belgian Institute of Natural Sciences, Brussels, Belgium; RMCA—Musee Royal de l’Afrique Centrale, Tervuren, Belgium; SAMC—Iziko Museum of Capetown, Cape Town, South Africa; USNM—National Museum of Natural History, Washington D.C., USA; ZMMU—Zoological Museum of the Moscow State University, Moscow, Russia; ZMUH—Zoologisches Institut und Zoologisches Museum, Universität von Hamburg, Hamburg, Germany; ZMUC—University of Copenhagen, Zoological Museum, Copenhagen, Denmark; and ZSM—Zoologische Staatssammlung, Munich, Germany.

Quoting the labels of the specimens: a slash (/) is used to divide data on different rows of a label, a semicolon (;) is used to divide data on different labels, and the author’s comments are provided in square brackets ([]).

### 2.4. Morphological Methods

A total of 1776 specimens were examined in this work. We used a Nikon SMZ25 stereoscopic microscope and a Nikon NiU compound microscope to examine the external morphology and genitalia of dry-mounted specimens. To examine the genitalia, they were first removed from the body and then cleared in warm 10% potassium hydroxide (KOH) solution (3 to 5 min) and washed in distilled water. After analysing the anatomical structure of the genitalia, they were stored in glycerol (pinned in vials under the specimens). All measurements are given in millimetres; measurements for males are given in parentheses.

**Figure 1:**
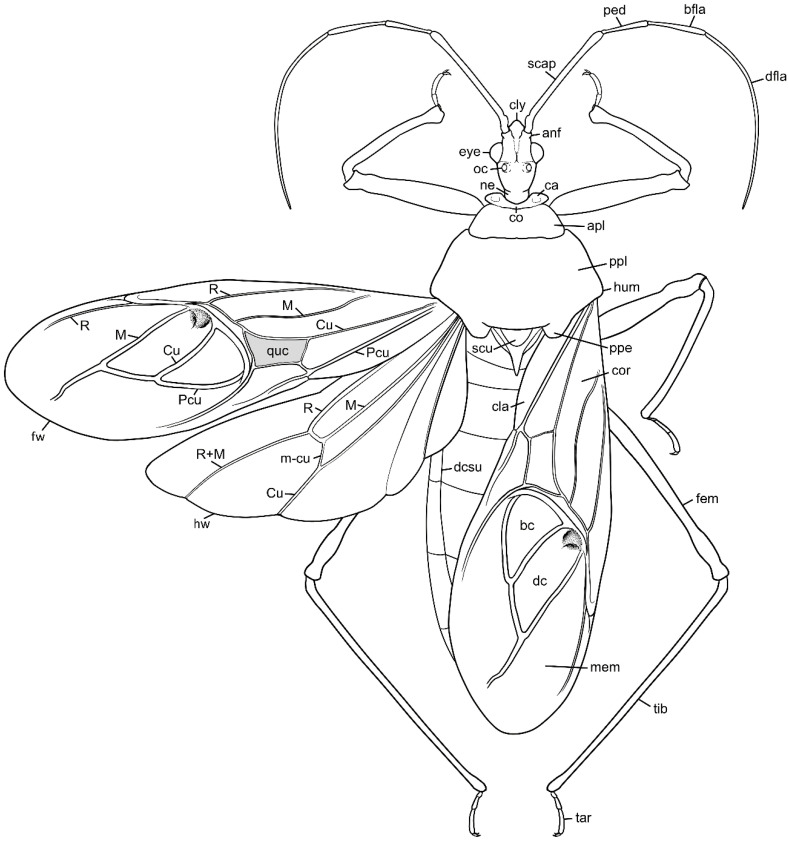
General morphology of *Phonoctonus*: **and**—antennifer; **apl**—anterior pronotal lobe; **bc**—basal cell; **bfla**—basiflagellomere; **ca**—callus (pl. calli); **cla**—clavus; **cly**—clypeus; **co**—collar; **cor**—corium; **Cu**—cubital vein; **dc**—distal cell; **dfla**—distiflagellomere; **dcsu**—dorsal connexival suture; **fem**—femur; **fw**—forewing; **hum**—humerus (pl. humeri); **hw**—hindwing; **M**—medial vein; **m-cu**—crossvein between media and cubitus; **mem**—membrane; **ne**—neck; **oc**—ocellus (pl. ocelli); **Pcu**—postcubital vein; **ped**—pedicellus of the antenna; **ppe**—posterior pronotal extensions; **ppl**—posterior pronotal lobe; **quc**—quadrate cell on the corium formed by the cubitus; **R**—radial vein; **scu**—scutellum; **scap**—scapus of the antenna; **tar**—tarsus; **tib**—tibia.

**Figure 2:**
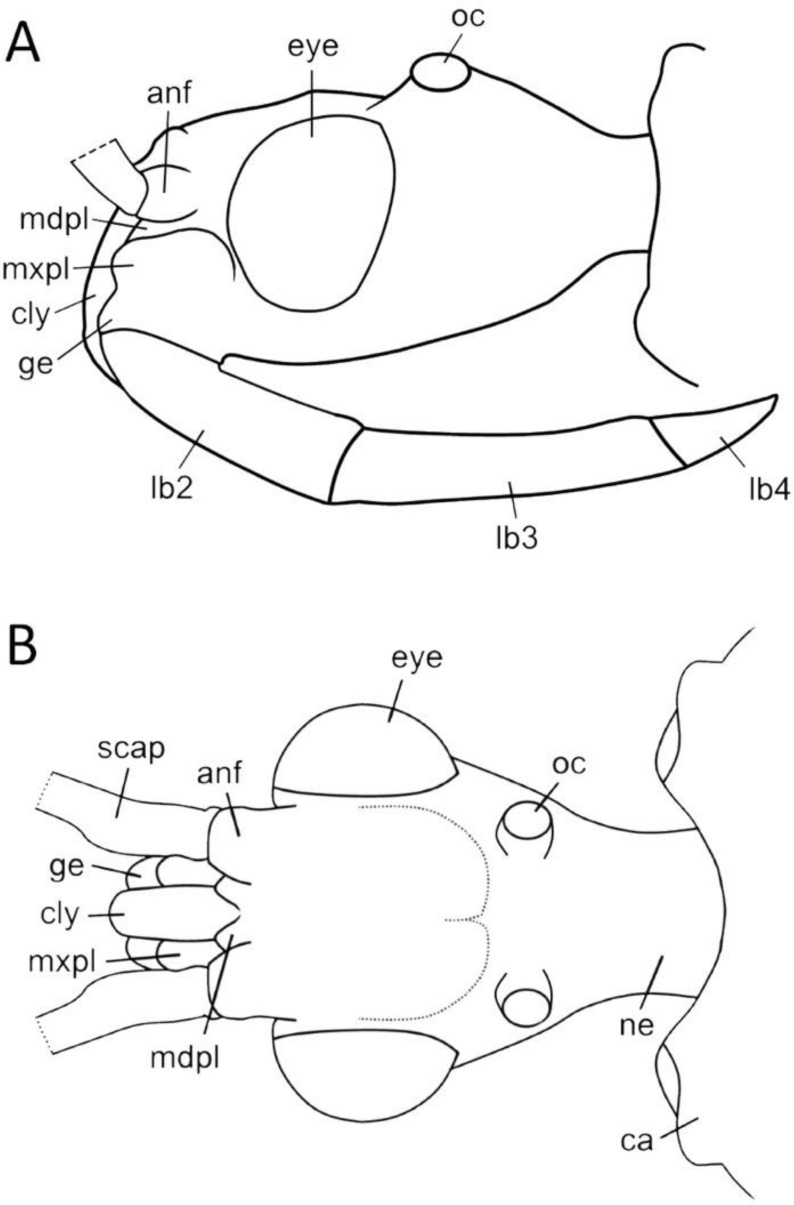
(**A**) Lateral and (**B**) dorsal view of general head morphology of *Phonoctonus*: **anf**—antennifer; **ca**—callus (pl. calli); **cly**—clypeus; **ge**—gena; **lb2-4**—second to fourth labial segments; **mdpl**—mandibular plate; **mxpl**—maxillary plate; **ne**—neck; **oc**—ocellus (pl. ocelli); **scap**—scapus of the antenna.

We used a Phenom XL scanning electron microscope (Phenom-World B.V., The Netherlands) (low vacuum conditions at 10, 15, and 20 accelerating voltages with a secondary electron detector) to take SEM photos. We used our method, based on using aluminium stubs with double-sided adhesive carbon tape to stabilise dry specimens covered with gold in a Pelco SC-6 sputter coater (Ted Pella Inc., Redding, CA, USA), resulting in a 35 nm layer. The morphological terminology for genitalia and external morphology follows [4,39,40] (Figure 1 and Figure 2). For extensions on the posterior edge of the pronotum, the name of the posterior pronotal extensions was adopted. The terminology used for sensilla follows [41,42,43].

Documentation of the selected morphological characteristics used in the phylogenetic analysis was prepared using a Nikon SMZ25 stereoscopic microscope and photographed using a Nikon DS-Fi2 camera with NIS-Elements D 4.50.00 64-Bit. The figures were prepared in FireAlpaca ver. 2.3.8 (PGN Inc., Japan), PhotoScape X ver. 4.0.2 (Mooii Tech, South Korea) and Image Composite Editor ver. 2.0.3.0 (Microsoft Corporation, Redmond, WA, USA). Photographs of all available type specimens are given with all labels.

### 2.5. Morphological Phylogenetic Analysis

A total of 25 characters (scored for both sexes) of 15 taxa were used to prepare a morphological matrix (Harpactorinae Amyot and Serville, 1843 [44]: *Coranus* sp., thirteen *Phonoctonus* species, and *Pseudophonoctonus* sp.). Representatives of the genus *Phonoctonus* showed slight variation. We did not want to use colour variation in the analyses because this one is very varied. Furthermore, this led to the description of many colour forms and species that turned out to be incorrect.

A matrix was prepared using Mesquite ver. 3.61 [45]. Unknown character states were coded with a “?”, while inapplicable states were coded with “–”. A csv file containing the character matrix is provided in Supplementary Material S1. Analyses were run using TNT ver. 1.5 [46,47] and NONA [48] implemented in Winclada ver. 1.61 [49,50], with parsimony as the optimality criterion. A swapping procedure TBR was employed for traditional searches with 1000 replications and ten trees saved per replication. All characters were unweighted and unordered.

**Figure 3:**
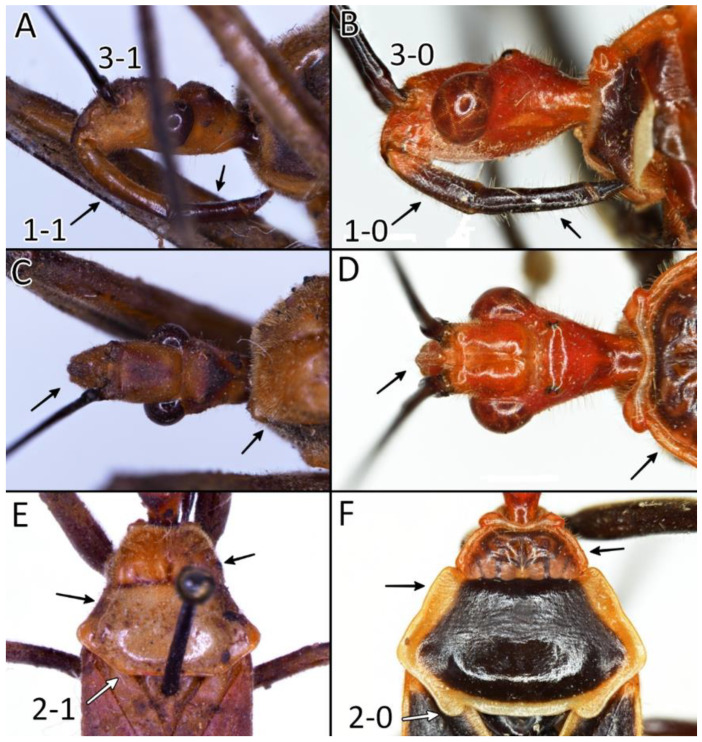
Comparison of the distinctive characters of representatives of the genera *Pseudophonoctonus* Schouteden, 1913 (*Pseudophonoctonus* sp. at **A**,**C**,**E**) and *Phonoctonus* Stål, 1853 (*P. fairmairei* Villiers, 1948 at **B**,**D**,**F**). (**A**,**B**) head in lateral view; (**C**,**D**) head in dorsal view; (**E**,**F**) anterior and posterior pronotal lobes. The arrows indicate the features described in the text.

**Figure 4:**
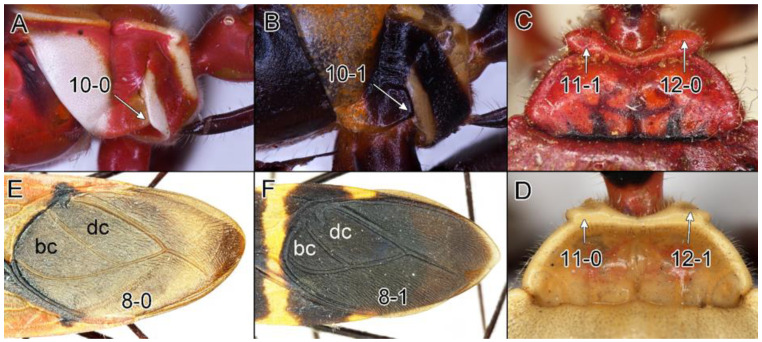
Morphological characters: (**A**) *P. caesar* Haglund, 1895 and (**B**) *P. bifasciatus* Villiers, 1948, thorax; (**C**) *P. caesar* Haglund, 1895 and (**D**) *P. nigrofasciatus* Stål, 1855, anterior pronotal lobe with a collar; (**E**) *P. caesar* Haglund, 1895 and (**F**) *P. elegans* Varela, 1904, hemelytron.

**Figure 5:**
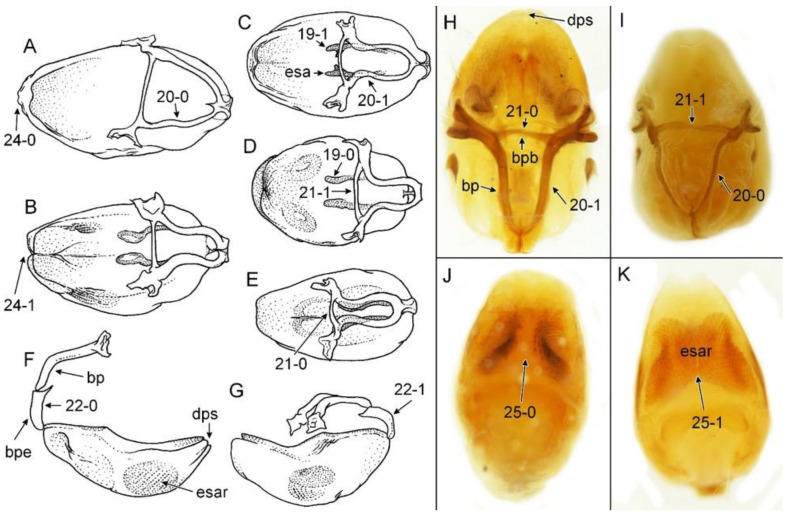
Morphological characters: (**A**) *Phonoctonus principalis* Gerstaecker, 1892; (**B**) *P. lutescens* (Guérin-Méneville & Percheron, 1834); (**C**) *P. immitis* Stål, 1865; (**D**) *P. nigrofasciatus* Stål, 1855; (**E**,**F**) *P. luridus* Miller, 1950; (**G**) *P. bifasciatus* Villiers, 1948; (**H**) *P. fasciatus* (Beauvois, 1805); (**I**) *P. principalis* Gerstaecker, 1892; (**J**) *P. luridus* Miller, 1950; and (**K**) *P. lutescens* (Guérin-Méneville & Percheron, 1834), aedeagus.

Below are provided descriptions and documentation of the characters (Figure 3, Figure 4 and Figure 5) used in the analysis and the states with their distribution among the analysed taxa. Descriptions are given only for the features of crucial importance at the level of the analysed genera.

1. **Second (first visible) labial segment**: (0) shorter than third (second visible) labial segment (Figure 3A); (1) longer than the third (second visible) labial segment (Figure 3B). Second (first visible) labial segment for all *Phonoctonus* species is shorter than the third (second visible).
2. **Posterior pronotal extensions**: (0) present (Figure 3F); (1) absent (Figure 3E). Among all analysed species, only representatives of the genus *Phonoctonus* have well-developed posterior pronotal extensions.
3. **Point of antennal insertion**: (0) close to the anterior margin of an eye (Figure 3B); (1) in half of the anteocular portion of the head (Figure 3A).
4. **Length of scapus**: (0) shorter than head and pronotum; (1) longer than head and pronotum.
5. **Length ratio of pedicellus to basiflagellomere**: (0) pedicellus longer or same as basiflagellomere; (1) basiflagellomere longer than pedicellus.
6. **Postocular portion of the head**: (0) same level or lower than anteocular portion of the head; (1) higher than anteocular portion of the head.
7. **Shape of the posterior pronotal lobe**: (0) straight; (1) arcuate.
8. **Size of basal cell and distal cell**: (0) same/similar size of basal cell and distal cell (Figure 4E); (1) basal cell visibly smaller than distal cell (Figure 4F).
9. **Shape of basal cell**: (0) triangular; (1) quadrangular.
10. **Position of proepimeron with respect to proepisternum**: (0) proepimeron and proepisternum touch their edges (Figure 4A); (1) proepimeron overlaps proepisternum (Figure 4B).
11. **Size of anterolateral angles of the anterior collar of the pronotum**: (0) enlarged (Figure 4D); (1) not enlarged (Figure 4C).
12. **Shape of anterolateral angles of the anterior collar of the pronotum**: (0) flattened (Figure 4C); (1) globular (Figure 4D).
13. **Apodeme depression of pronotum**: (0) shallow; (1) deep.
14. **Length of the apodeme depression of pronotum**: (0) short; (1) long.
15. **Lateral ridges of anterior pronotal lobe**: (0) enlarged; (1) not enlarged.
16. **Shape of anterior pronotal lobe**: (0) slightly convex; (1) strongly convex.
17. **Bright belt on the 4th antennal segment**: (0) absent; (1) present; (-) inapplicable.
18. **Shape of hairs on fossula**: (0) flat; (1) concave; (?) unknown; (-) inapplicable.
19. **Endosomal struts of aedeagus**: (0) rounded (Figure 5D); (1) divided (Figure 5C).
20. **Basal plate**: (0) wide (Figure 5A,I); (1) narrow (Figure 5C,H).
21. **Basal plate bridge**: (0) short (Figure 5E,H); (1) long (Figure 5D,I).
22. **Basal plate extension**: (0) delicately curved (Figure 5F); (1) distinctly curved (Figure 5G).
23. **Length of basal plate extension**: (0) long; (1) short.
24. **Dorsal phallothecal sclerite**: (0) not divided apex (Figure 5A); (1) divided apex (Figure 5B).
25. **Endosomal spine areas**: (0) not connected (Figure 5J); (1) connected (Figure 5K).

## 3. Taxonomic Accounts

### 3.1. Taxonomy

#### Genus Phonoctonus Stål, 1853

*Phonoctonus* Stål, 1853, 10: 262. Type species: *Reduvius fasciatus* Beauvois, 1805, p. 65, by subsequent designation (Stål, 1853, 10: 262) (Figure 1 and Figure 2A,B).

Revised diagnosis:

The representatives of the genus can be recognised by the following combination of characters: body large (18–32.5 mm) with medium-sized (2.7–4.5 mm), globular head. The dorsal surface of the postocular portion of the head is delicately gibbous, reaching over the margin of the dorsal surface of the anteocular portion of the head. Relatively large and globular eyes widely placed and not reaching dorsal and ventral margins of the head in lateral view. Short neck. Labial segments relatively thick. Pronotum gibbous in lateral view. Femur apically enlarged.

The representatives of genus *Phonoctonus* Stål, 1853 are similar to other Afrotropical genus *Pseudophonoctonus* Schouteden, 1913 [51], but have a distinctly larger body size (in length and width; the body length of *Pseudophonoctonus* species does not exceed 19 mm); first visible labial segment shorter than the second (in *Pseudophonoctonus* first visible labial segment distinctly longer than the second) (Figure 3A,B); head with anteocular portion of the head shorter than length of the diameter of an eye in dorsal view vs. long and robust anteocular portion in *Pseudophonoctonus* with a length of three diameters of an eye (Figure 3C,D); margins of the anterior pronotal lobe are enlarged in *Phonoctonus* (not enlarged in *Pseudophonoctonus*) (Figure 3C–F); lateral edges of posterior pronotal lobe strongly flattened (slightly flatted in *Pseudophonoctonus*) (Figure 3E,F); posterior pronotal extensions in *Phonoctonus* are visibly enlarged (not enlarged in *Pseudophonoctonus*) (Figure 3E,F). Representatives of any other genus found in Africa and Madagascar cannot be confused with *Phonoctonus*.

Redescription:

**Colouration**: Body ranging from orange, red, brown to black with dark or light patterns on pronotum and hemelytra.

**Structure**: Body length between 18 to 32.5 mm, slender (except for the robust females of *P. caesar* Haglund, 1895).

**Figure 6:**
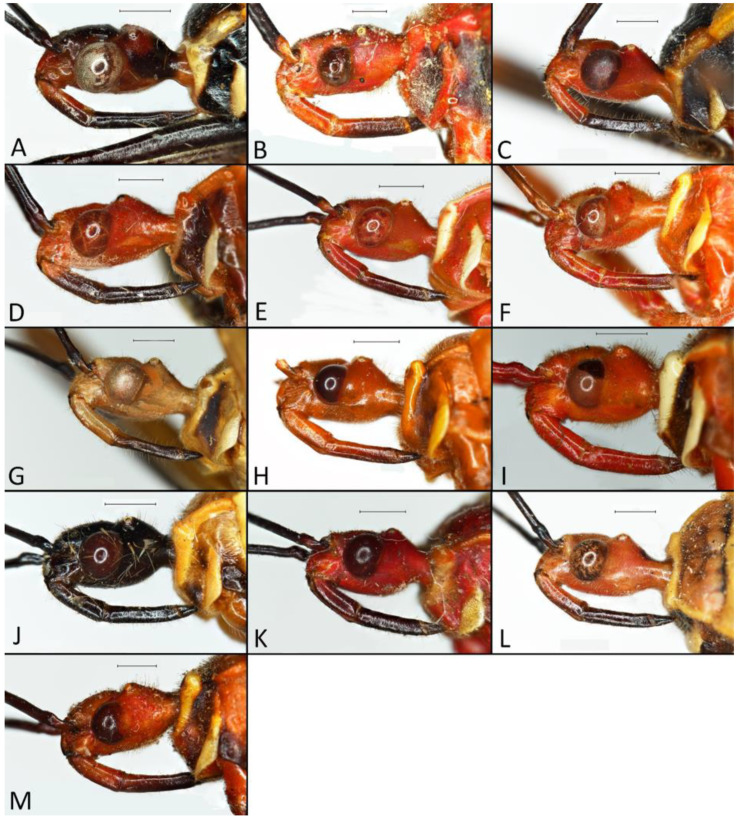
Head in lateral view: (**A**) *Phonoctonus bifasciatus* Villiers, 1948; (**B**) *P.* *caesar* Haglund, 1895; (**C**) *P. elegans* Varela, 1904; (**D**) *P. fairmairei* Villiers, 1948; (**E**) *P. fasciatus* (Beauvois, 1805); (**F**) *P. grandis* Signoret, 1860; (**G**) *P. immitis* Stål, 1865; (**H**) *P. luridus* Miller, 1950; (**I**) *P. lutescens* (Guérin-Méneville & Percheron, 1834); (**J**) *P. nigrofasciatus* Stål, 1855; (**K**) *P. picta* Schouteden, 1932; (**L**) *P. picturatus* Fairmaire, 1858; and (**M**) *P. principalis* Gerstaecker, 1892. Scale = 1 mm.

**Figure 7:**
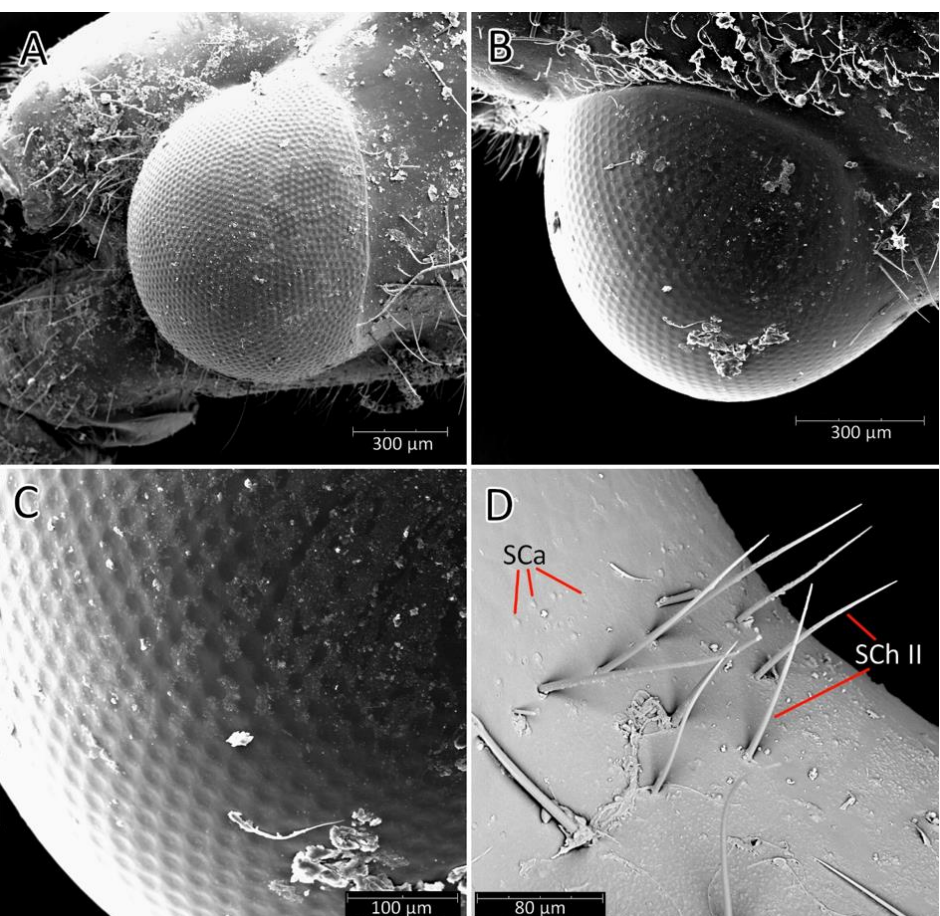
*Phonoctonus fasciatus* (Beauvois, 1805): (**A**) head with a compound eye, lateral view; (**B**) compound eye, ventral view; (**C**) magnification of the compound eye ommatidia; (**D**) base of the eye with sensilla chaetica type II (**SCh II**) and sensilla campaniformia (**SCa**).

**Head**: Medium-sized (length 2.7–4.5 mm), relatively short and ovoid in lateral view (Figure 2A and Figure 6A–M), with widely placed, large eyes not reaching dorsal and ventral margins of the head in lateral view; ommatidia covered by typical hexagonal but collapsing facets, without any trichoid sensilla (Figure 7); at the base of the eye in a ventral view, from the postocular side, a few sensilla chaetica type II (SChII) are situated as well as sensilla campaniformia (SCa) (Figure 7D).

**Figure 8:**
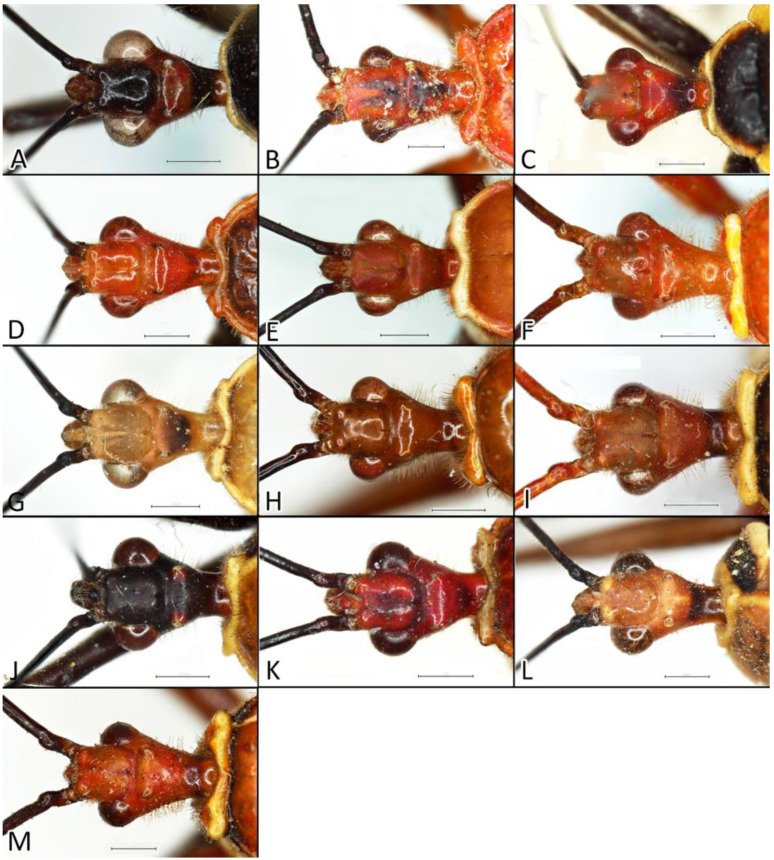
Head in dorsal view: (**A**) *Phonoctonus bifasciatus* Villiers, 1948; (**B**) *P.* *caesar* Haglund, 1895; (**C**) *P. elegans* Varela, 1904; (**D**) *P. fairmairei* Villiers, 1948; (**E**) *P. fasciatus* (Beauvois, 1805); (**F**) *P. grandis* Signoret, 1860; (**G**) *P. immitis* Stål, 1865; (**H**) *P. luridus* Miller, 1950; (**I**) *P. lutescens* (Guérin-Méneville & Percheron, 1834); (**J**) *P. nigrofasciatus* Stål, 1855; (**K**) *P. picta* Schouteden, 1932; (**L**) *P. picturatus* Fairmaire, 1858; and (**M**) *P. principalis* Gerstaecker, 1892. Scale = 1 mm.

Anteocular portion is relatively short, convex dorsally with the ventral surface flattened in lateral view; short or medium-sized setae on anteocular portion and relatively long and dense setae on postocular portion; postocular portion short, thickened basally with small ocelli placed widely on flattened tubercles (Figure 2B and Figure 8A–M). Clypeus was distinctly convex, directed downwards. Transversal suture of head curved posteriorly (Figure 2B). Neck short (Figure 6 and Figure 8).

**Figure 9:**
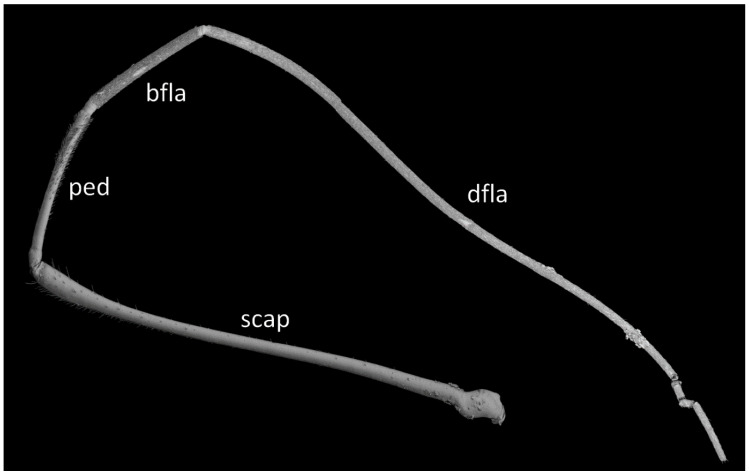
Antennae of *Phonoctonus fasciatus* (Beauvois, 1805). **bfla**—basiflagellomere; **dfla**—distiflagellomere; **ped**—pedicellus of antenna; **scap**—scapus of antenna.

**Figure 10:**
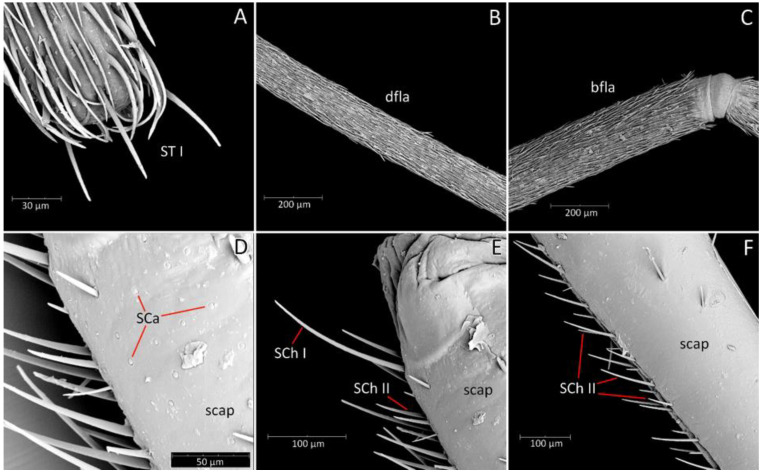
Antennae of *Phonoctonus fasciatus* (Beauvois, 1805): (**A**) distiflagellomere apex with sensilla trichoidea; (**B**) general view of the distiflagellomere showing the densely arranged sensilla chaetica type II; (**C**) general view of the basiflagellomere with sensilla chaetica type II; (**D**–**F**) scapus with a different type of sensilla. **bfla**—basiflagellomere; **dfla**—distiflagellomere; **SCa**—sensilla campaniformia; **scap**—scapus of the antenna; **SCh I**—sensillum chaeticum type I; **SCh II**—sensillum chaeticum type II; **ST I**—sensillum trichoideum type I.

Antennifers are of various sizes, with scapus and distiflagellomerus the longest, while pedicellus and basiflagellomerus are relatively short (Figure 9). Scapus enlarged apically. Apices of antennifers are directed frontally. All antennomeres are thin, covered by short setae. Apex of distiflagellomerus is covered by sensilla trichoidea type I (Figure 10A); pedicellus, basiflagellomerus, and distiflagellomerus densely covered with sensilla chaetica type II (SChII) (Figure 9 and Figure 10B,C); on scapus sensilla, chaetica type I and type II and sensilla campaniformia (Figure 10D–F). Most of the sensilla chaetica located in depression runs along the scapus (Figure 10F).

**Figure 11:**
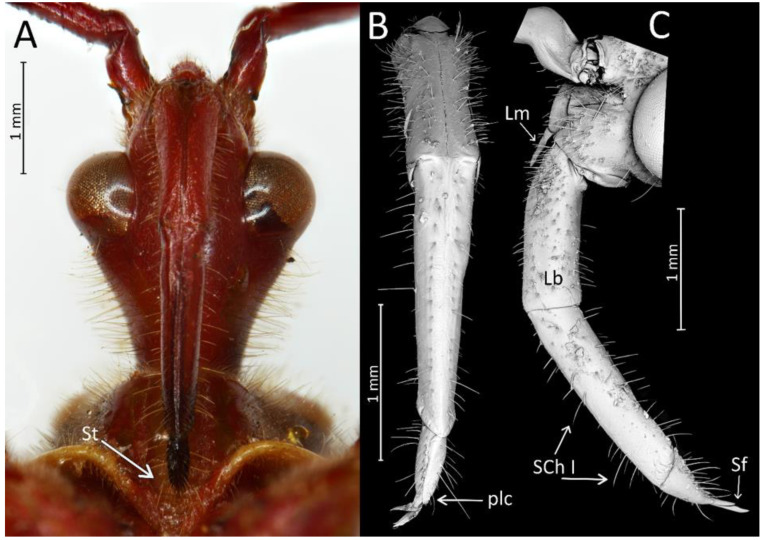
Head: (**A**) of *Phonoctonus lutescens* (Guérin-Méneville & Percheron, 1834), ventral view; (**B**) of *P. fasciatus* (Beauvois, 1805), ventral view; (**C**) of *P. fasciatus* (Beauvois, 1805), lateral view. **Lb**—labium; **Lm**—labrum; **plc**—plectrum; **SCh I**—sensillum chaeticum type I; **Sf**—stylet fascicle; **St**—stridulitrum.

**Figure 12:**
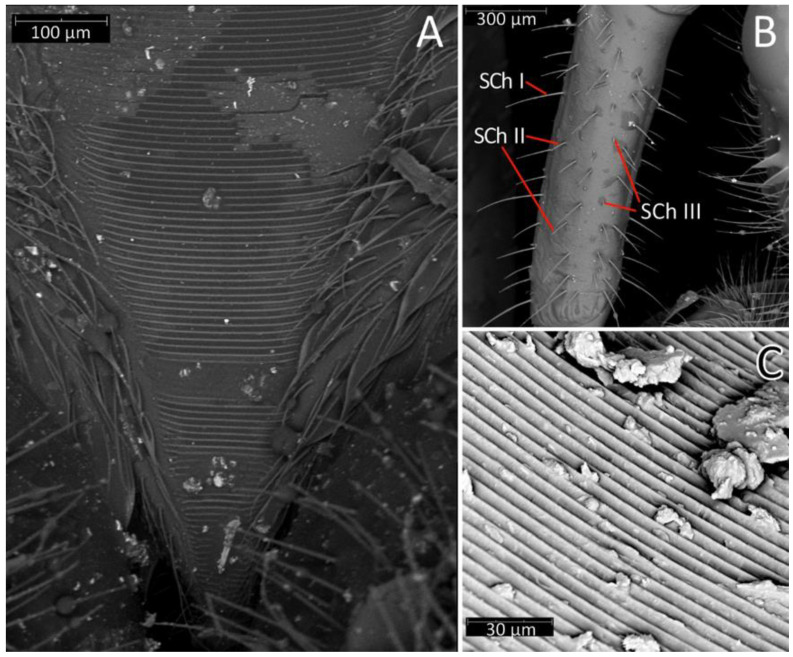
(**A**) Prostemal stridulatory organ of *Phonoctonus fasciatus* (Beauvois, 1805). (**B**) Third segment of the rostrum of *P. immitis* Stål, 1865 with different types of sensilla chaetica. (**C**) Magnification of the transverse ridges of prostemal stridulatory organ of *P. fasciatus* (Beauvois, 1805), which are rubbed by the plectrum. **SCh I**—sensillum chaeticum type I; **SCh II**—sensillum chaeticum type II; **SCh III**—sensillum chaeticum type III.

**Figure 13:**
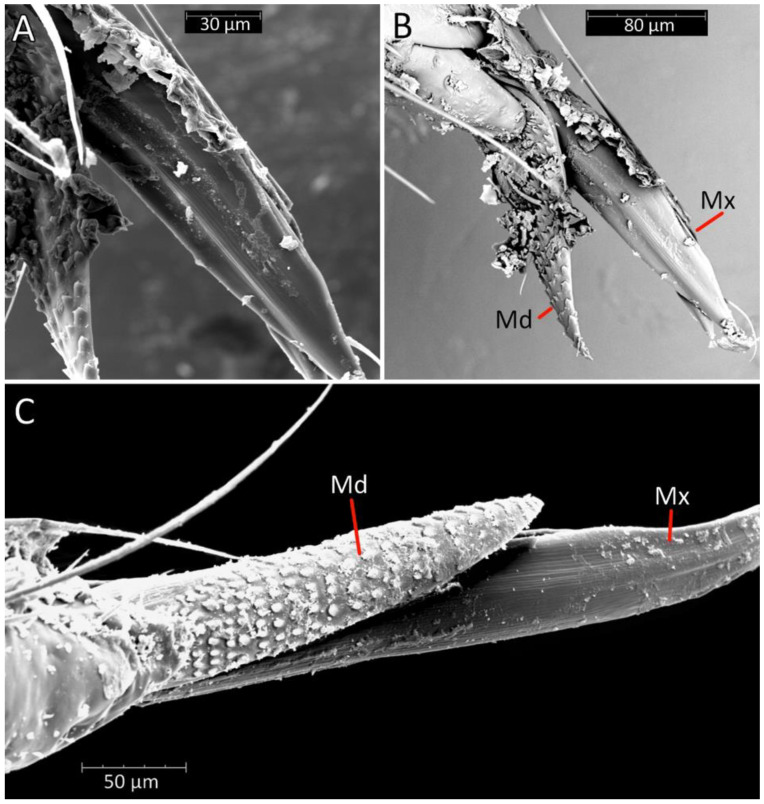
(**A**–**C**) Mandibular stylet (**Md**) and maxillary stylet (**Mx**) of *Phonoctonus fasciatus* (Beauvois, 1805).

Head in the ventral view shows the position of the labium—its apex (plectrum) rests in the cavity of prosterna with stridulitrum (Figure 11A). First visible labial segment is thick and rounded in cross-section. Second visible labial segment is thinner than the first, surpassing the posterior margin of the head (Figure 2A and Figure 11A–C). All visible segments of the labium are covered with numerous sensilla chaetica type I (SChI), sensilla chaetica type II (SChII), and much shorter sensilla chaetica type III (SChIII)—all placed in a flexible socket (Figure 11B,C and Figure 12B). Apex of mandibular stylets with separate, strong hooks. Apex of maxillary stylets is acute; subapical portion smooth, without blunt process; central portion of maxillary stylets is bulging, with well-marked longitudinal grooves (Figure 13A–C).

**Figure 14:**
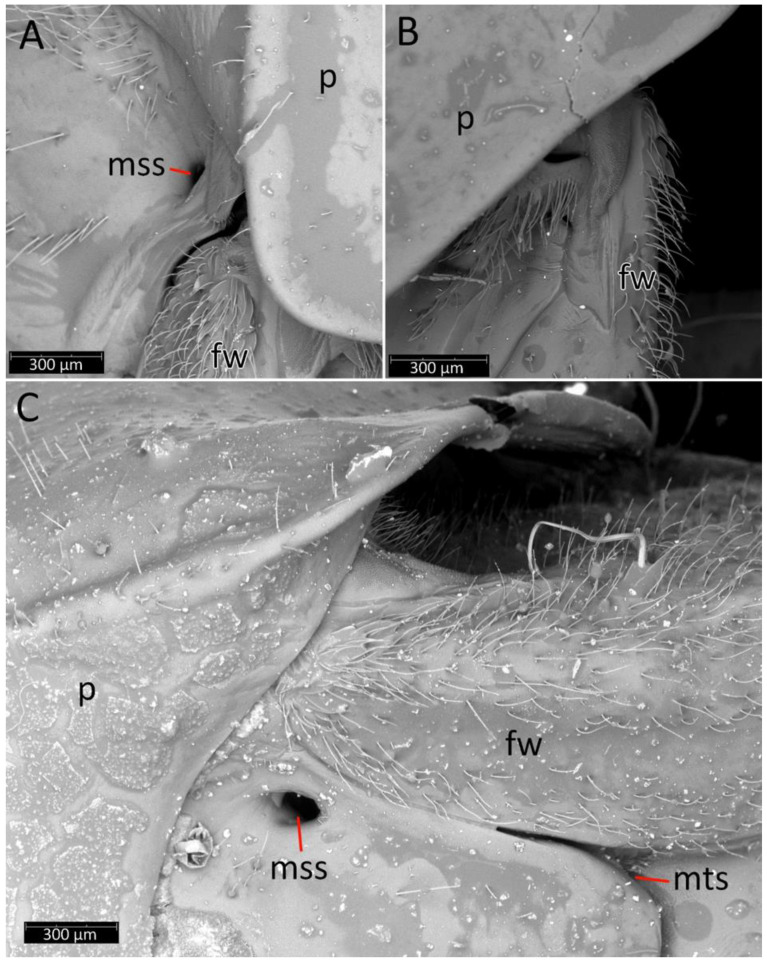
*Phonoctonus luridus* Miller, 1950. (**A**) mesothoracic spiracle with the left fore wing’s base, dorso-lateral view. (**B**) Structure of central depression of scutellum with sensilla, dorsal view. (**C**) Meso- and metathorax with spiracles, lateral view. **fw**—fore wing; **mss**—mesothoracic spiracle; **mts**—metathoracic spiracle; **p**—pronotum.

**Figure 15:**
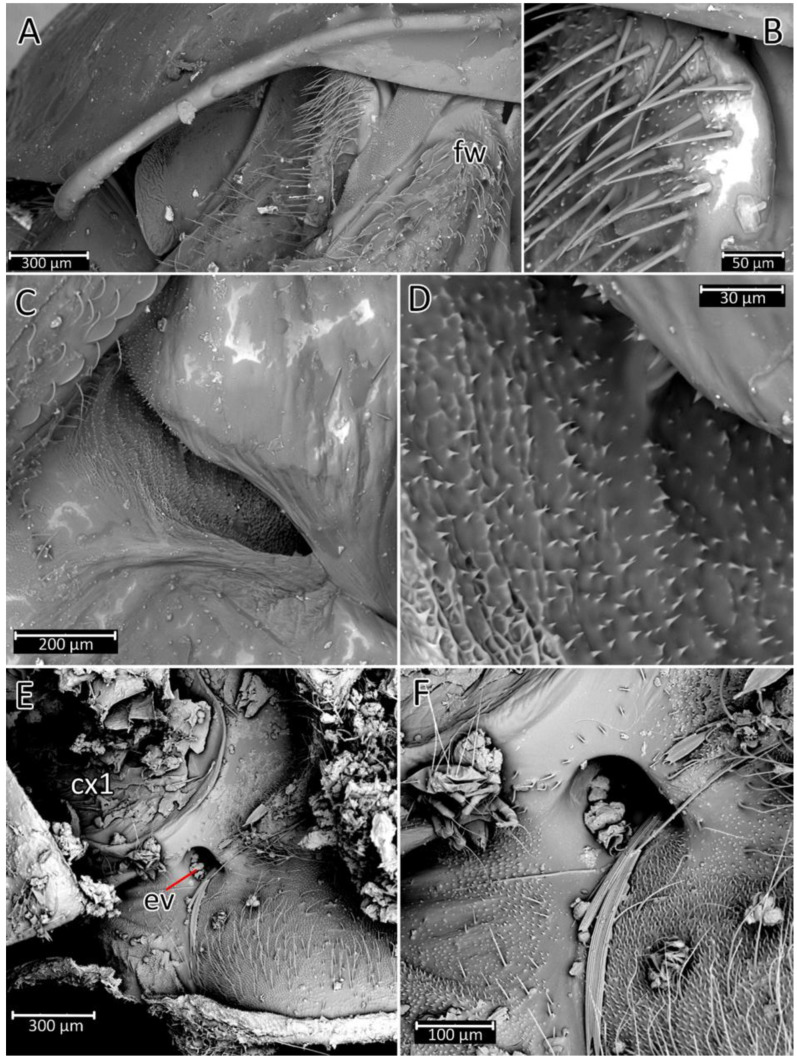
*Phonoctonus luridus* Miller, 1950: (**A**) structure of central depression of scutellum with sensilla under the pronotum, lateral view; (**B**) magnification of the plate with sensilla. *P. nigrofasciatus* Stål, 1855: (**C**) Metathoracic spiracle and (**D**) magnification of its inner surface covered by microtrichia. *P. fasciatus* (Beauvois, 1805): (**E**) Evaporatorium under procoxal and (**F**) its magnification. **cx1**—procoxal cavity; **ev**—evaporatorium; **fw**—fore wing.

**Thorax**: Prosterna is short and stridulitrum relatively wide, formed as simple striae, surrounded by various sized setae (Figure 12A,C). Mesosternum with a longitudinal depression is posteriorly delimited by distinct ridges (gradually narrowing in a triangular shape) connected with the metasternum. Metasternum is flattened and delicately depressed in the posterior portion. Meso- and metasternum, lateral portions of meso- and metathorax are covered by various sized, scarce setae. In lateral view, meso- and metathoracic spiracles are visible (Figure 14A–C). Mesothoracic spiracles are in the form of an ovoid hole, while metathoracic spiracles elongate and are irregular in shape (Figure 14C and Figure 15C,D). Under the pronotum is located a plate covered densely with sensilla (Figure 14B and Figure 15A,B)—a structure of central depression of scutellum. On the dorsal side, next to the fore legs at the prosterna is a single and clearly visible evaporatorium (Figure 15E,F).

**Figure 16:**
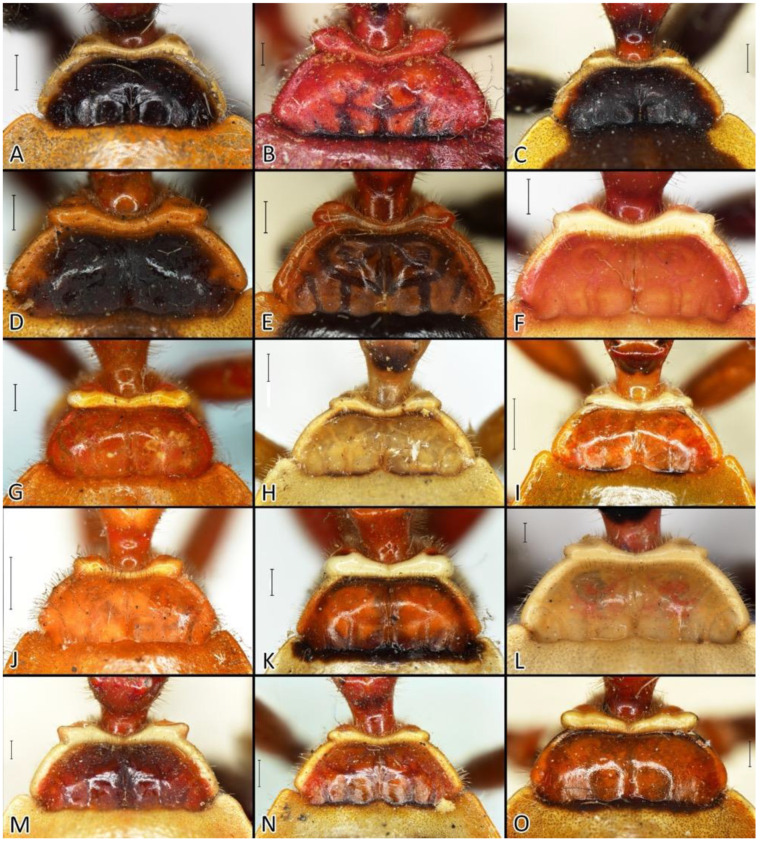
Anterior pronotal lobe, collar with calli and neck in dorsal view: (**A**) *Phonoctonus bifasciatus* Villiers, 1948; (**B**) *P.* *caesar* Haglund, 1895; (**C**) *P. elegans* Varela, 1904; (**D**,**E**) *P. fairmairei* Villiers, 1948; (**F**) *P. fasciatus* (Beauvois, 1805); (**G**) *P. grandis* Signoret, 1860; (**H**,**I**) *P. immitis* Stål, 1865; (**J**) *P. luridus* Miller, 1950; (**K**) *P. lutescens* (Guérin-Méneville & Percheron, 1834); (**L**) *P. nigrofasciatus* Stål, 1855; (**M**) *P. picta* Schouteden, 1932; (**N**) *P. picturatus* Fairmaire, 1858; and (**O**) *P. principalis* Gerstaecker, 1892. Scale = 0.5 mm.

**Figure 17:**
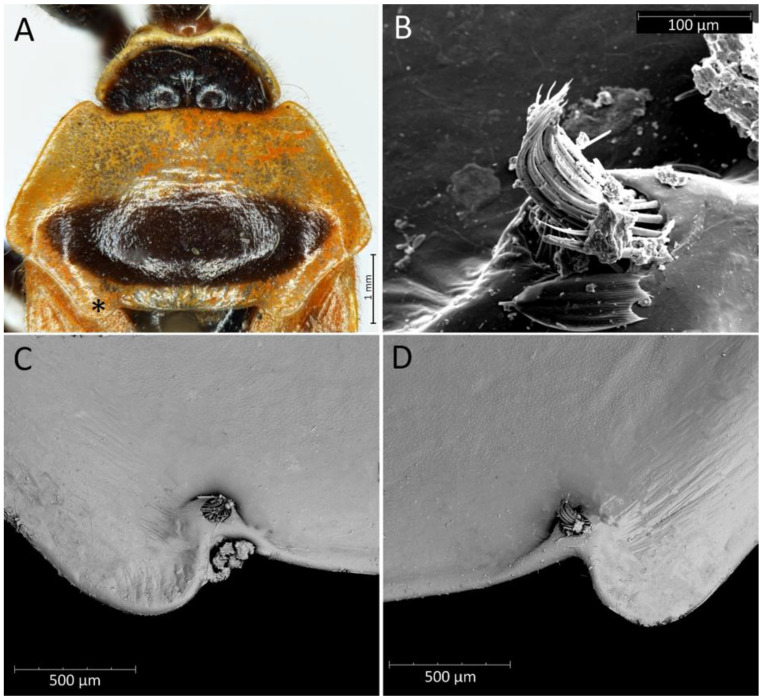
*Phonoctonus bifasciatus* Villiers, 1948: (**A**) anterior and posterior lobe of pronotum with humeri marked with *. *P. fasciatus* (Beauvois, 1805): (**B**) magnification of the bunches of sensilla at the underside of the posterior pronotal lobe using secondary electron detector (SED); view of the mentioned bunches of sensilla (**C**) on the right and (**D**) on the left side of the pronotum.

Anterior lobe of the pronotum is relatively small and distinctly narrower than the posterior lobe of the pronotum (Figure 3F and Figure 16). Anterior lobe has a distinct longitudinal groove. Collar of the anterior lobe is very distinct with various sized calli (Figure 8). Apices of calli are directed laterally (Figure 8). Margins of the anterior and posterior lobes of the pronotum have short and dense setae. Posterior lobe is two times longer than the anterior lobe. Humeral angles of the pronotum are rounded. Posterior pronotal extensions are clearly visible (Figure 1, Figure 3F and Figure 17A). On the underside of the posterior pronotal lobe, on the edge of posterior pronotal extensions, bunches of sensilla are located—probably sensilla trichoidea (Figure 17B,D). Posterior lobe has a delicate and wide depression between posterior pronotal extensions. Scutellum has a medial depression and Y-shaped calli (Figure 1); lateral margins of scutellum are covered by dense, medium-sized setae.

**Figure 18:**
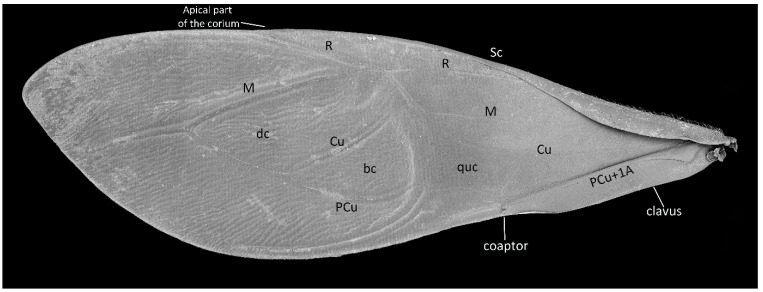
Ventral surface of the fore wing of *Phonoctonus fasciatus* (Beauvois, 1805). **bc**—basal cell; **Cu**—cubitus; **dc**—distal cell; **M**—media; **PCu**—poscubitus; **PCu+A1**—poscubitus and first anal vein; **quc**—quadrate cell; **R**—radius; **Sc**—subcostal.

**Figure 19:**
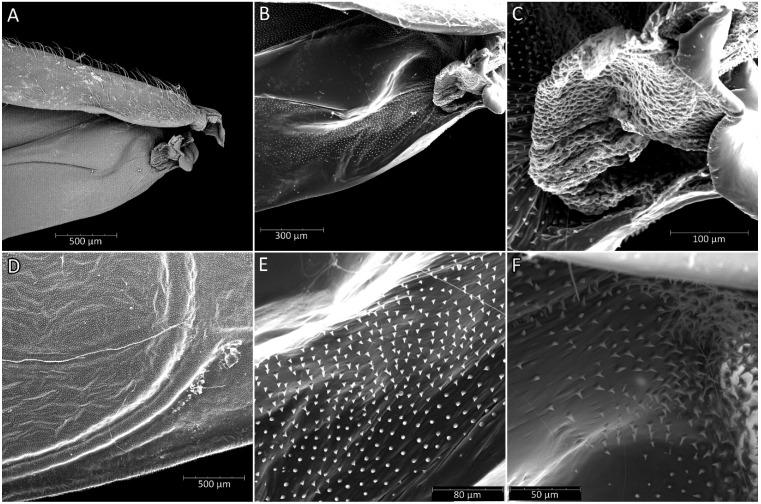
Details of the fore wing of *Phonoctonus fasciatus* (Beauvois, 1805), ventral side: (**A**) base of the wing with some muscles and fold of the wing with sensilla chaetica; (**B**) base of the wing using secondary electron detector (SED) and (**C**) magnification of the muscles; and (**D**–**F**) different magnifications of microtrichia at the ventral surface of the fore wing.

**Figure 20:**
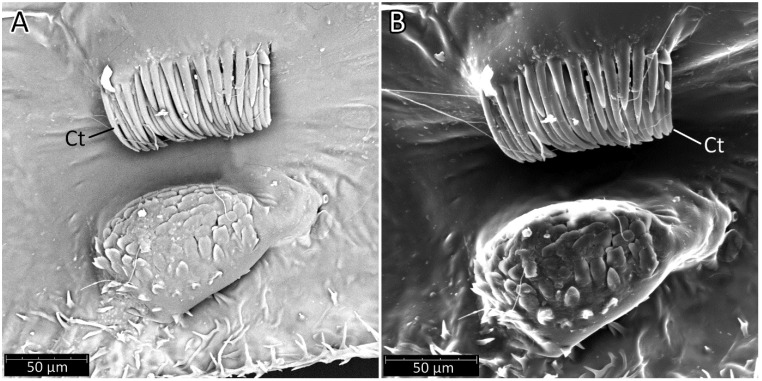
(**A**) Coaptor at the ventral side of the fore wing of *Phonoctonus fasciatus* (Beauvois, 1805). (**B**) Coaptor using a secondary electron detector (SED). **Ct**—ctenidia.

**Figure 21:**
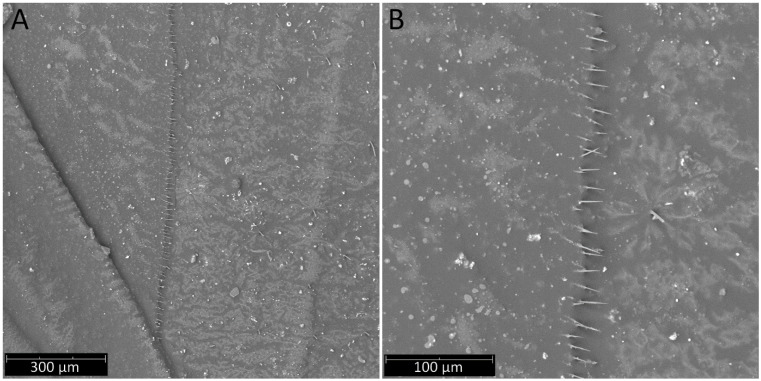
(**A**,**B**) Dorsal surface of the fore wing of *Phonoctonus fasciatus* (Beauvois, 1805).

**Hemelytra**: Slender and long, distinctly surpassing the apex of the abdomen. Corium is pigmented with very short setae. Membrane is dull, half-transparent, and dark. Basal cells are various sizes (smaller or similar in size to the distal cell), with large discal cell (Figure 1). The major veins on the forewing are well marked (Figure 18). Cubitus forms a quadrate cell—a characteristic feature for the subfamily Harpactorinae. Media, cubitus, and poscubitus veins designate discal and basal cells at the membranous portion of the forewing. Only the fold of the forewing is covered by sensilla chaetica (Figure 19A), while the whole ventral surface is covered by numerous microtrichia (Figure 19B–F). Microtrichia are present all over the membrane, corium, clavus, and veins. By the presence of the muscles on the torn forewing, it is possible to observe their structure—single fibres resemble starry plates (Figure 19C). Coaptor is located at the inner lower edge of the clavus—a wing-coupled structure consisting of three parts—ctenidia on top, a nonstructured area in the middle, and a rough protruding portion covered with tile-like flat structures at the bottom (Figure 18 and Figure 20). Dorsal surface of the forewing is covered by a single row of pointed setae where the veins run and irregularly spaced sensilla are placed in the inflexible socket (Figure 21).

**Figure 22:**
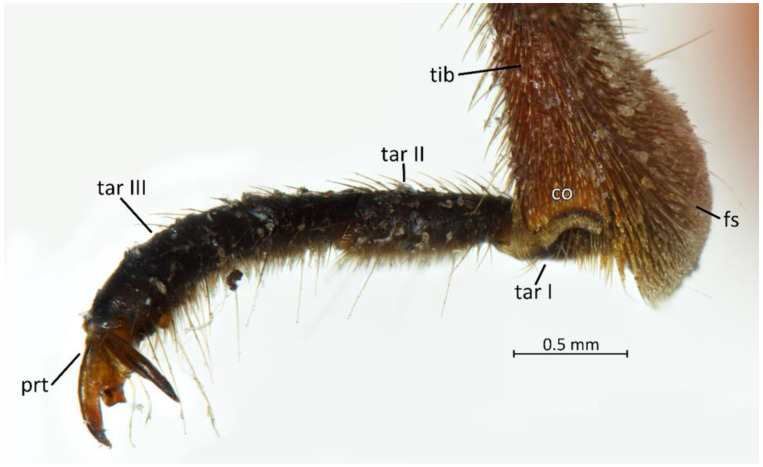
The tibiae with tarsi and pretarsi of the foreleg with fossula spongiosa of *Phonoctonus caesar* Haglund, 1895, lateral view. **co**—comb; **fs**—fossula spongiosa; **prt**—pretarsus; **tar I**—the first tarsomere; **tar II**—the second tarsomere; **tar III**—the third tarsomere; **tib**—tibia.

**Figure 23:**
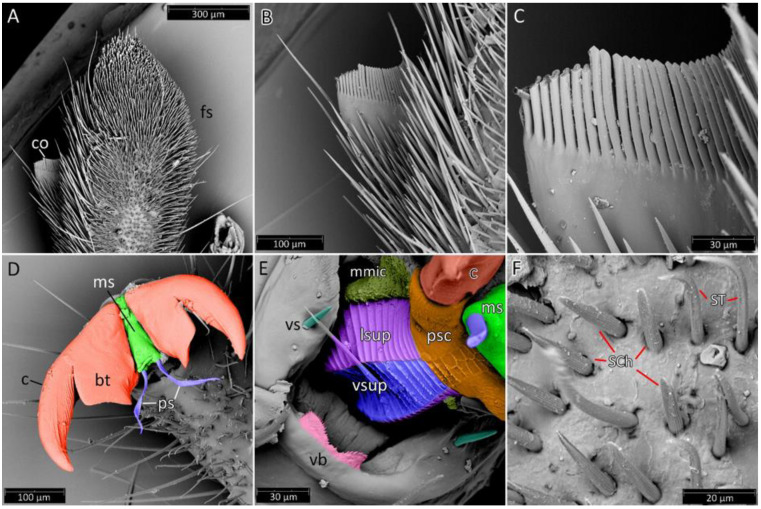
*Phonoctonus elegans* Varela, 1904: (**A**) foretibial fossula spongiosa, ventral view; (**B**,**C**) foretibial comb on the spur; (**D**) dorsal view of pretarsus. *Phonoctonus lutescens* (Guérin-Méneville & Percheron, 1834): (**E**) ventral view of the pretarsus. *Phonoctonus immitis* Stål, 1865: (**F**) sensilla trichoidea and sensilla chaetica at the dorsal surface of the third tarsomere apex. **bt**—basal tooth; **c**—claw; **co**—comb; **dl**—distal lamella; **fs**—fossula spongiosa; **lsup**—lateral surface of unguitractor plate; **mmic**—membrane with microtrichia; **ms**—median sclerite; **ps**—parempodial setae; **psc**—parempodial sclerite; **ST**—sensilla trichoidea; **SCh**—sensilla chaetica; **vsup**—ventral surface of unguitractor plate; **vb**—ventral brush; **vs**—ventrolateral seta of rim of distal tarsomere.

**Figure 24:**
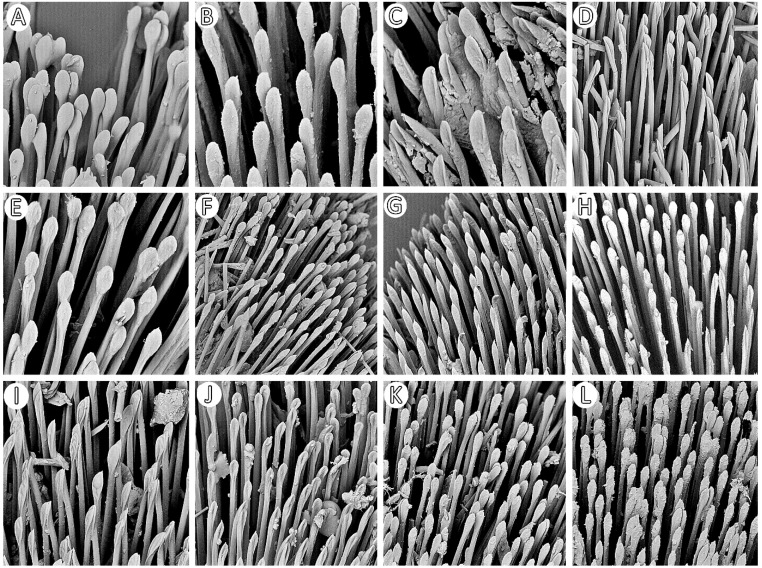
Tenant hairs on a fossula spongiosa of (**A**) *Phonoctonus fasciatus* (Beauvois, 1805); (**B**) *P. immitis* Stål, 1865; (**C**) *P. nigrofasciatus* Stål, 1855; (**D**) *P. elegans* Varela, 1904; (**E**) *P. fairmairei* Villiers, 1948; (**F**) *P. picturatus* Fairmaire, 1858; (**G**) *P. picta* Schouteden, 1932; (**H**) *P. luridus* Miller, 1950; (**I**) *P. lutescens* (Guérin-Méneville & Percheron, 1834); (**J**) *P. principalis* Gerstaecker, 1892; (**K**) *P. grandis* Signoret, 1860; and (**L**) *P. caesar* Haglund, 1895.

**Figure 25:**
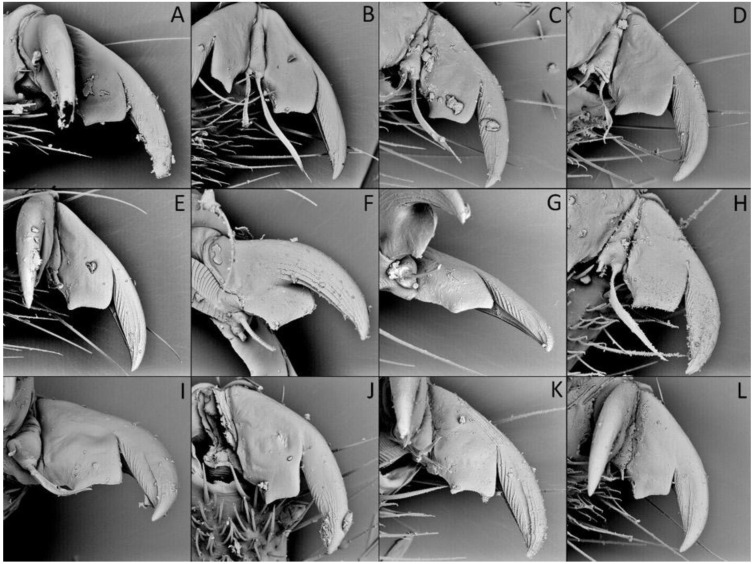
Dorsal view of pretarsus of (**A**) *Phonoctonus fasciatus* (Beauvois, 1805); (**B**) *P. immitis* Stål, 1865; (**C**) *P. nigrofasciatus* Stål, 1855; (**D**) *P. elegans* Varela, 1904; (**E**) *P. fairmairei* Villiers, 1948; (**F**) *P. picturatus* Fairmaire, 1858; (**G**) *P. picta* Schouteden, 1932; (**H**) *P. luridus* Miller, 1950; (**I**) *P. lutescens* (Guérin-Méneville & Percheron, 1834); (**J**) *P. principalis* Gerstaecker, 1892; (**K**) *P. grandis* Signoret, 1860; and (**L**) *P. caesar* Haglund, 1895.

**Legs**: Relatively long with short setae. Ventral side of the fore and middle femur is covered with very dense and short setae. Femur gradually narrows into the apex with a visible subapical enlargement; tibia is distinctly thinner than the femur, distinctly extended apically and covered with medium-sized and short setae. The third tarsomere, the longest (longer than the first and second combine), has claws the same length or longer than the first tarsomere. Fossula spongiosa and foretibial comb (a brush-like structure used for grooming) are placed on the spur at the fore tibia (Figure 22 and Figure 23A–C). Tenant hairs on the fossula spongiosa basically have two forms—rounded or pointed at the end and flat or spoon-shaped (Figure 24). Pretarsus has symmetrical claws and basal tooth on each claw (Figure 23D and Figure 25). Lateral and ventrolateral setae are on the rim on the distal tarsomere. Parempodial setae are flattened along the entire length. In the ventral view of the pretarsus are the lateral and ventral surfaces of the unguitractor plate, and parempodial sclerite and membrane with microtrichia (Figure 23E). At the end of the third tarsomere, next to the pretarsus, are sensilla trichoidea and chaetica (Figure 23F).

**Figure 26:**
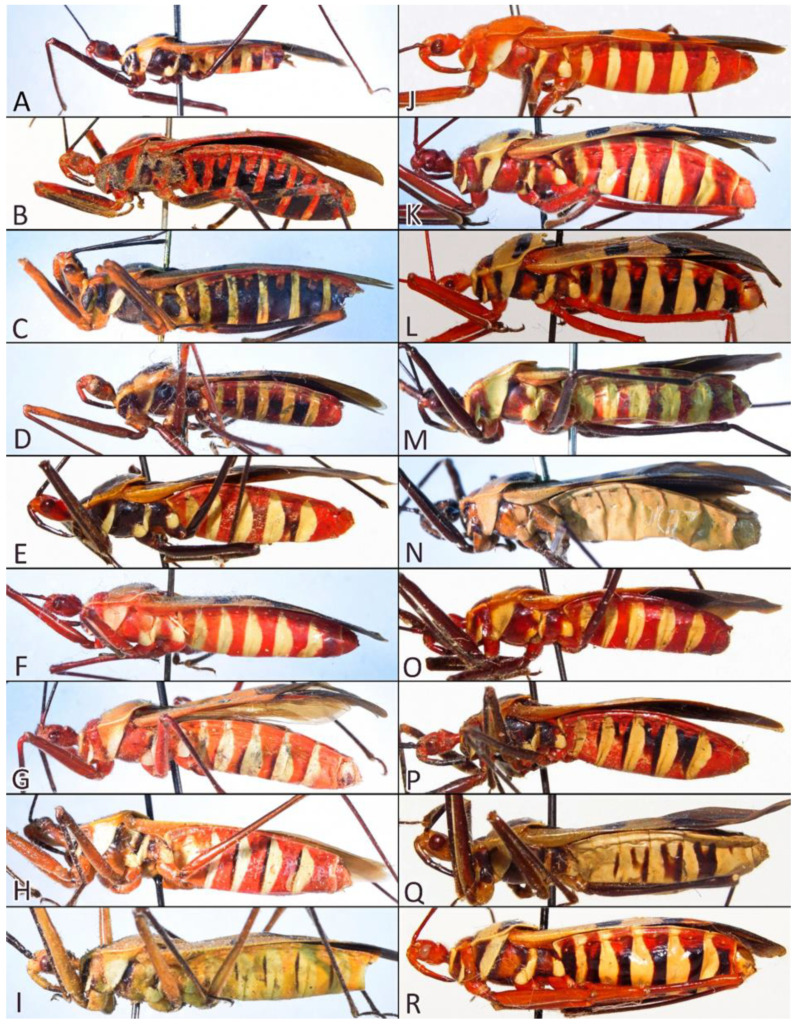
Lateral view of individuals representing species of the genus *Phonoctonus* Stål, 1853: (**A**) *Phonoctonus bifasciatus* Villiers, 1948; (**B**,**C**) *P.* *caesar* Haglund, 1895; (**D**) *P. elegans* Varela, 1904; (**E**) *P. fairmairei* Villiers, 1948; (**F**) *P. fasciatus* (Beauvois, 1805); (**G**) *P. grandis* Signoret, 1860; (**H**,**I**) *P. immitis* Stål, 1865; (**J**) *P. luridus* Miller, 1950; (**K**,**L**) *P. lutescens* (Guérin-Méneville & Percheron, 1834); (**M**,**N**) *P. nigrofasciatus* Stål, 1855; (**O**) *P. picta* Schouteden, 1932; (**P**,**Q**) *P. picturatus* Fairmaire, 1858; and (**R**) *P. principalis* Gerstaecker, 1892.

**Figure 27:**
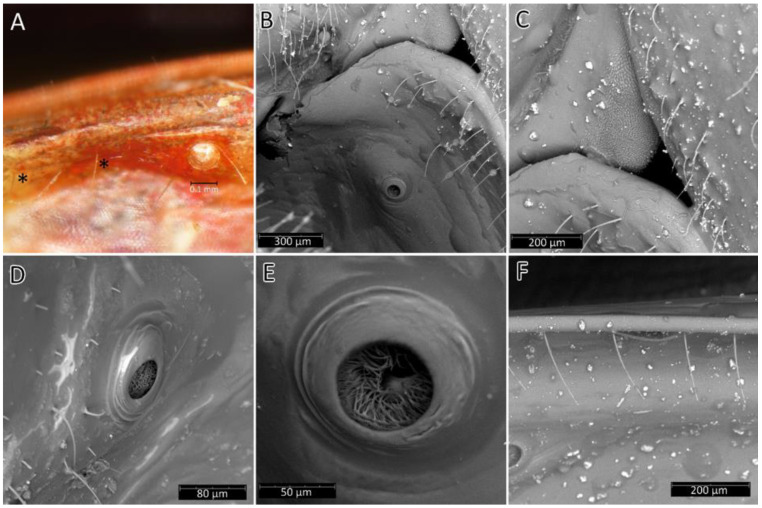
*Phonoctonus elegans* Varela, 1904: (**A**) second abdominal segment with spiracle and sensilla chaetica (marked by *), lateral view. *P. luridus* Miller, 1950: (**B**) contact area of thorax and abdomen, lateral view; (**C**) magnification of sensory field; (**D**,**E**) magnification of second abdominal spiracle; (**F**) sensilla chaetica at the lower portion of the external margin of connexiva, lateral view.

**Abdomen**: Fusiform; connexivum medium-sized, covered by hemelytra in the dorsal view. The ventral portion of the abdomen is u-shaped in cross-section with a slightly flattened medial portion (Figure 26). Spiracles are on each abdominal segment (Figure 27). Inside the spiracle are several rows of air-filtering cilia (Figure 27D,E). Sensory field is at the contact area of the thorax and abdomen (Figure 27C). All along the ventral, external margin of connexives are single sensilla chaetica type I (Figure 27F).

Identification key to species of the genus *Phonoctonus* Stål, 1853

**Figure 28:**
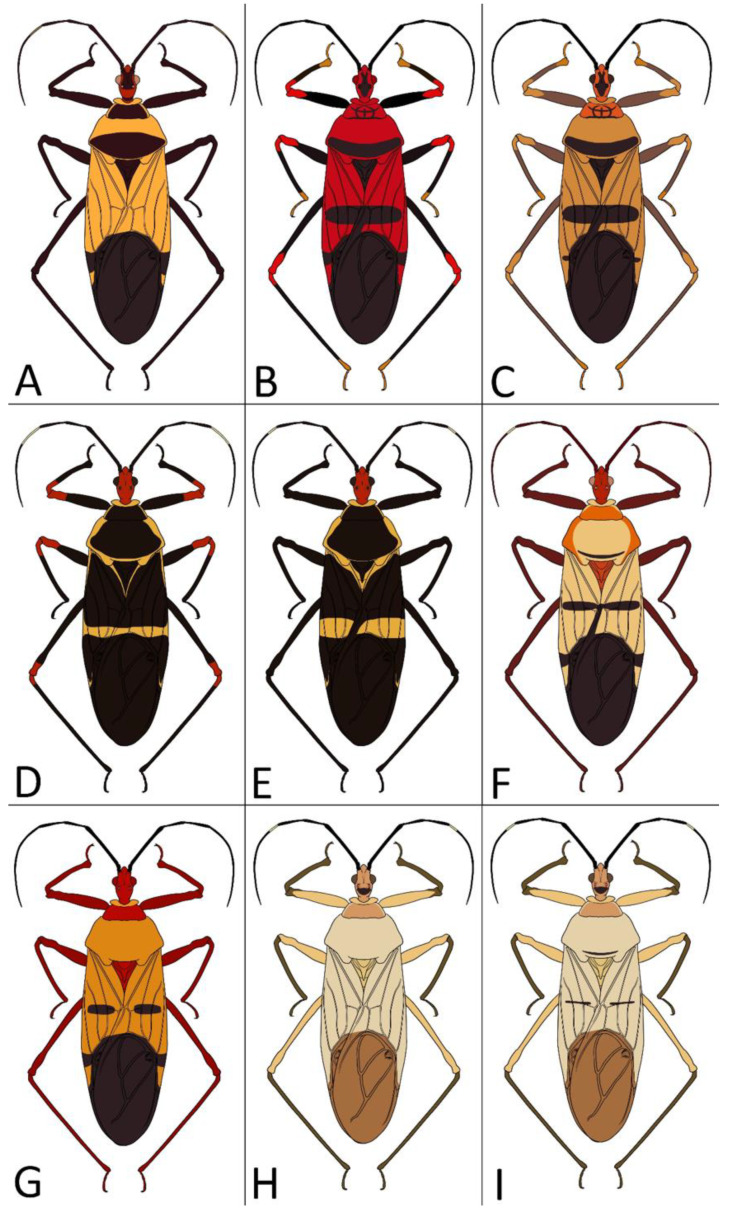
Dorsal colour patterns of the representatives of *Phonoctonus* Stål, 1853: (**A**) *Phonoctonus bifasciatus* Villiers, 1948; (**B**,**C**) *P.* *caesar* Haglund, 1895; (**D**) *P. elegans* Varela, 1904; (**E**) *P. fairmairei* Villiers, 1948; (**F**) *P. fasciatus* (Beauvois, 1805); (**G**) *P. grandis* Signoret, 1860; and (**H**,**I**) *P. immitis* Stål, 1865.

**Figure 29:**
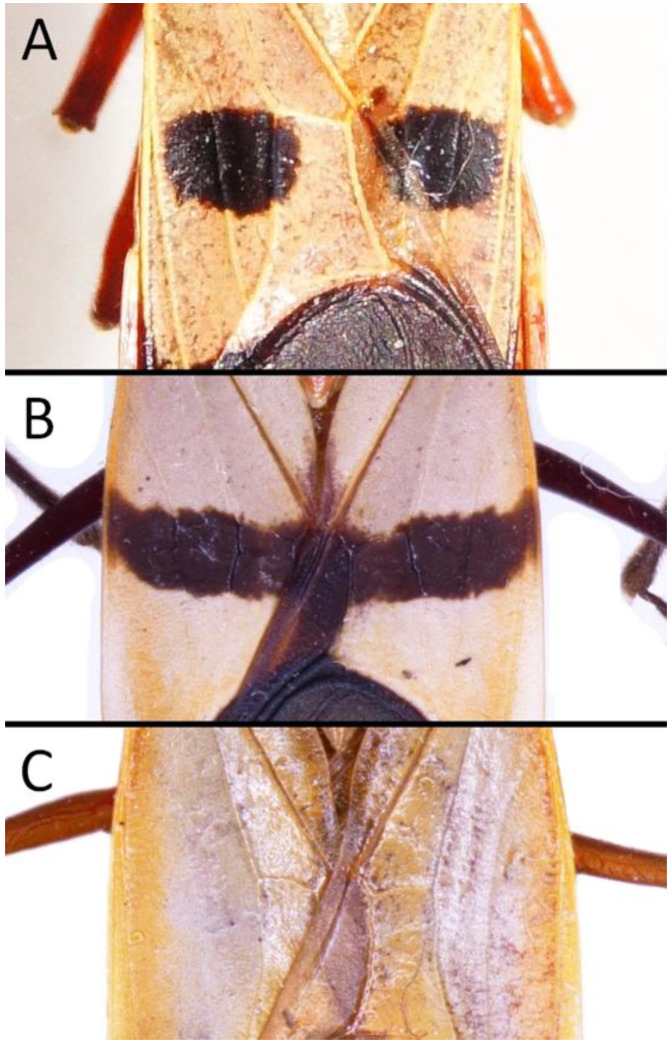
Black patterns in the middle portion of corium: (**A**) *Phonoctonus lutescens* (Guérin-Méneville & Percheron, 1834)—the shape of rounded or ovoid spots; (**B**) *P. fasciatus* (Beauvois, 1805)—the shape of transversal stripes; and (**C**) *P. immitis* Stål, 1865—absent.

**Figure 30:**
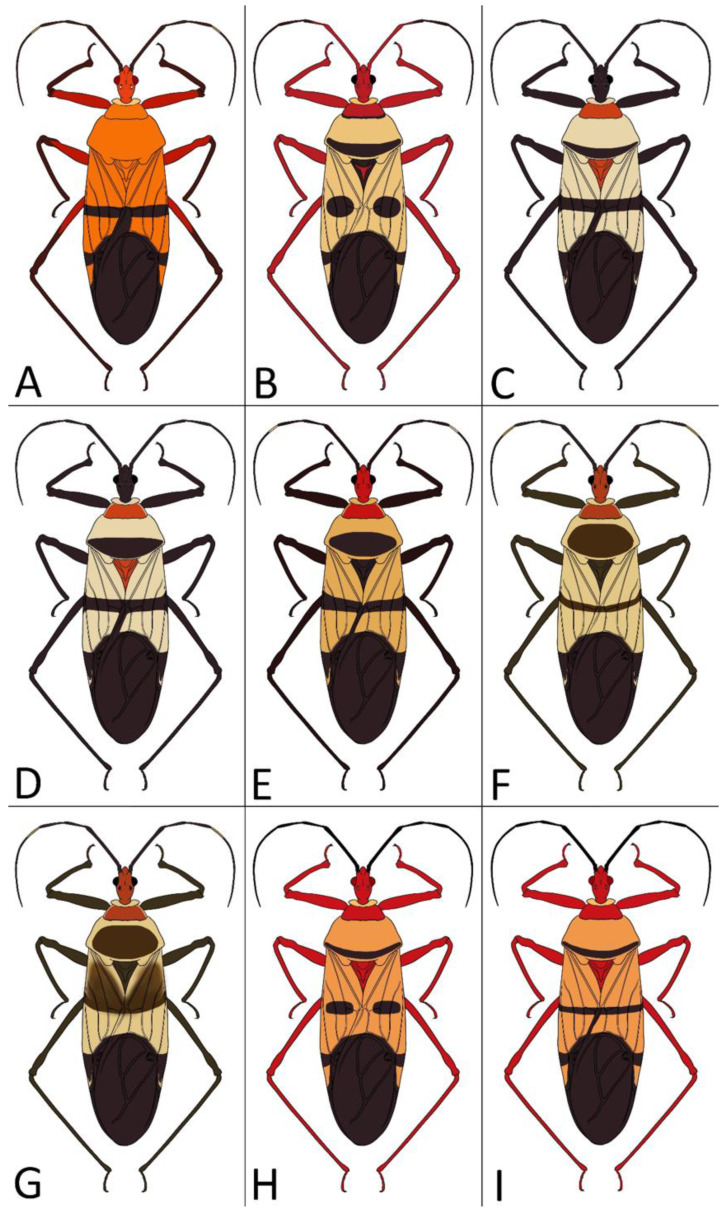
Dorsal colour patterns of the representatives of *Phonoctonus* Stål, 1853: (**A**) *P. luridus* Miller, 1950; (**B**) *P. lutescens* (Guérin-Méneville & Percheron, 1834); (**C**,**D**) *P. nigrofasciatus* Stål, 1855; (**E**) *P. picta* Schouteden, 1932; (**F**,**G**) *P. picturatus* Fairmaire, 1858; and (**H**,**I**) *P. principalis* Gerstaecker, 1892.

1. Body length over 28 mm, robust (Figure 28B,C)…………………………*P. caesar* Haglund
1′. Body length less than 27 mm, slender………………………………………………………2
2. Body black………………………………………………………………………………………3
2′. Body brightly coloured………………………………………………………………………4
3. Femurs with red or orange apical portion (Figure 28D)…………………*P. elegans* Varela
3′. Femurs black (Figure 28E)…………………………………………………*P. fairmairei* Villiers
4. Middle region of the corium with black spots or stripes (Figure 29A,B)………………5
4′. Middle region of the corium without black spots or stripes (Figure 29C)………………7
5. Middle region of the corium with a pair of black, rounded or ovoid spots (Figure 29A)……………………………………………………………………………………………6
5′. Middle region of the corium with a transversal stripe (Figure 29B)……………………8
6. Black spots on hemelytra are large and oval, hemelytra yellowish to greyish, posterior pronotal lobe with a black transversal stripe near posterior margin (Figure 30B)………………………………………………………*P. lutescens* (Guérin-Méneville & Percheron)
6′. Black spots on hemelytra medium or small and oblong, hemelytra bright orange, posterior pronotal lobe without black transversal stripe (Figure 28G)………………………………………………………………………*P. grandis* Signoret
7. Scapus very thin and long, only delicately enlarged in the apical portion; anterior pronotal lobe black (Figure 28A)………………………………………*P. bifasciatus* Villiers
7′. Scapus robust medium-sized distinctly enlarged in apical portion; anterior pronotal lobe light (Figure 28H,I)…………………………………………………………*P. immitis* Stål
8. Anterior and posterior pronotal lobes unicolorous (Figure 30A)………*P. luridus* Miller
8′. Pronotum with dark patterns…………………………………………………………9
9. Middle transversal stripes on the corium very thick (over 2 mm wide) in lateral portions, posterior pronotal lobe with very thick, black pattern (Figure 30E)…………………………*P. picta* Schouteden
9′. Middle transversal stripes on the corium thin…………………………………………10
10. Posterior pronotal lobe with a dark, thick stripe covering most of the middle portion (Figure 30F)………………………………………………….…………*P. picturatus* Fairmaire
10′. Posterior pronotal lobe with a dark stripe near the posterior margin of posterior pronotal lobe………………………………………………………………………………………11
11. Head and anterior pronotal lobe distinctly red……………………………………12
11′. Head and anterior pronotal lobe other than red…………………………………………13
12. Transversal suture of pronotum and lateral margins of posterior pronotal lobe red (Figure 28F)……………………………………………………………*P. fasciatus* (Beauvois)
12′. Transversal suture of pronotum black; lateral margins of posterior pronotal lobe concolor (Figure 30G,H)………………………………………………*P. principalis* Gerstaecker
13. Head and membrane black, anterior pronotal lobe darker than posterior pronotal lobe (Figure 30C,D)…………………………………………………………*P. nigrofasciatus* Stål
13′. Head pale with black postocular portion, membrane brown, anterior and posterior pronotal lobes concolor (Figure 28H,I)……….………………………………*P. immitis* Stål

1. ***Phonoctonus bifasciatus* Villiers, 1948** (Figure 4B, Figure 5H, Figure 6A, Figure 8A, Figure 16A, Figure 17A, Figure 26A, Figure 28A, Figure 31A and Figure 32) **stat. nov.**

*Phonoctonus fasciatus* var. *bifasciatus* Villiers, 1948: 9:124. Holotype (♂): Gabon: Oyem; MNHN.

**Figure 31:**
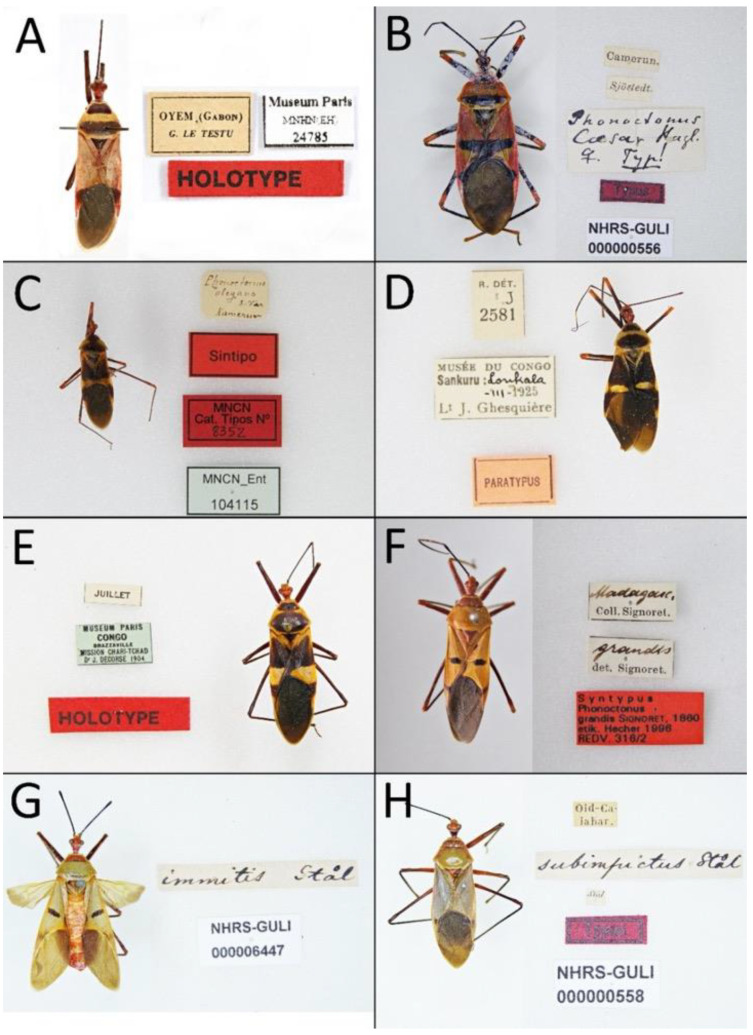
Type specimens of the representatives of *Phonoctonus* Stål, 1853: (**A**) *Phonoctonus bifasciatus* Villiers, 1948; (**B**) *P. caesar* Haglund, 1895; (**C**) *P. elegans* Varela, 1904; (**D***) P. elegans* var. *stricta* Schouteden, 1932; (**E**) *P. fairmairei* Villiers, 1948; (**F**) *P. grandis* Signoret, 1860; (**G**) *P. immitis* Stål, 1865; and (**H**) *P. subimpictus* Stål, 1865.

**Type material examined**: • OYEM (Gabon)/G. Le Testu; HOLOTYPE; Museum Paris (MNHN) (Figure 31A).

Additional material examined—see Supplementary Materials S2.

**Figure 32:**
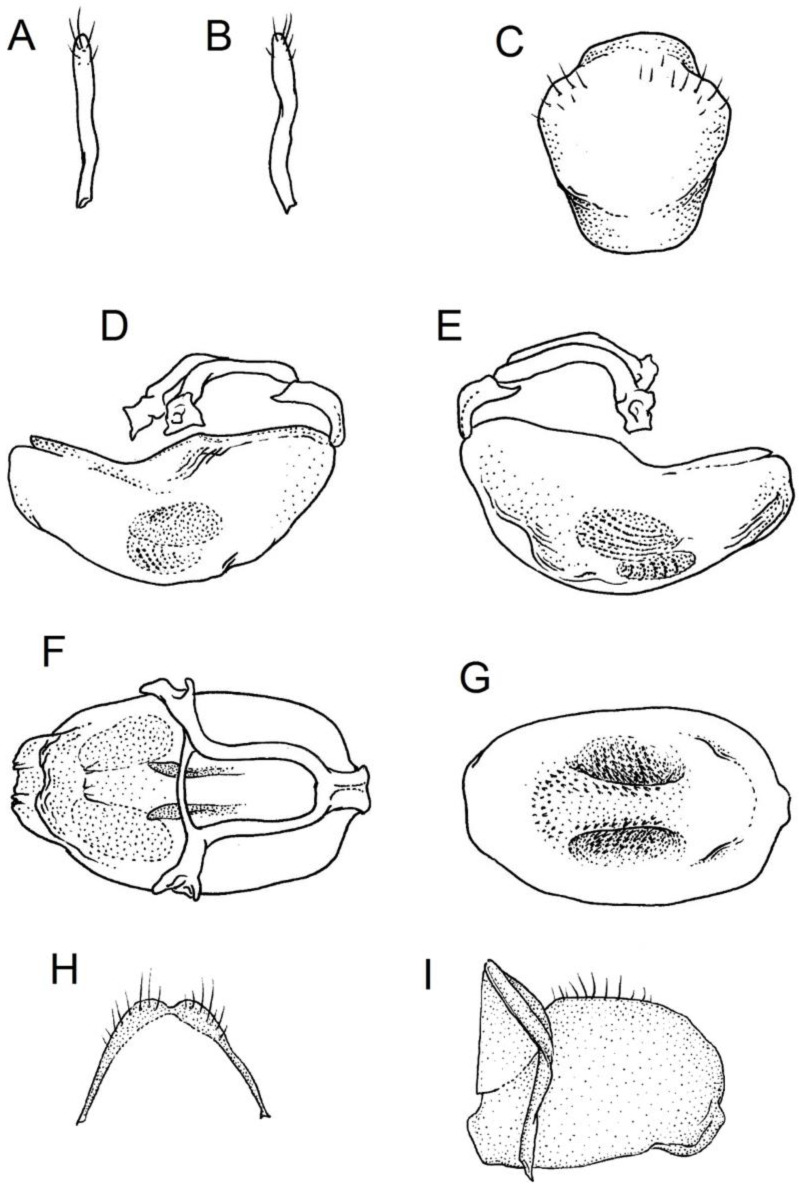
*Phonoctonus bifasciatus* Villiers, 1948: (**A**) left paramere; (**B**) right paramere; (**C**) pygophore, ventral view; (**D**) aedeagus, right lateral view; (**E**) aedeagus, left lateral view; (**F**) aedeagus, dorsal view; (**G**) aedeagus, ventral view; (**H**) styloids, outer view; and (**I**) gonocoxite of abdominal segment VIII.

**Diagnosis**: Recognised among other species in this genus by the combination of the following characters: black anterior pronotal lobe with yellowish collar and lateral margins (Figure 16A); dark posterior 1/2 of posterior pronotal lobe and legs (Figure 28A); aedeagus with membranous struts of endosoma with deeply depressed and elongated areas on both sides containing elongated spines (Figure 32G).

Redescription

**Colour**: Body generally pale—orange with brown and black patterns (Figure 28A).

**Head**: Red; interocular portion (except margins) and posterior portion of postocular portion dark brown. Antennal segments brown, basal portion of scapus reddish. First visible labial segment apical and ventrally red, dorsally black. Second and third visible labial segments black.

**Thorax**: Dark brown to black except for whitish pro-, meso- and metepisternum. Collar of anterior pronotal lobe yellowish with brown, flattened areas on calli (Figure 6A and Figure 8A). Anterior pronotal lobe dark brown, yellowish margins (Figure 16A). Transversal suture of the pronotum is dark brown. Posterior pronotal lobe orange with a large, brown pattern near the posterior margin. Scutellum black, apex pale. Coxa and trochanter brown with darker areas. Femur, tibia, and tarsus brown (femur with almost black dorsal surfaces). Thorax ventrally pale brown. Corium of hemelytra orange with a wide, black, transversal stripe on the basal portion of the external apical cell (Figure 28A). Membrane brown.

**Abdomen**: Ventrally yellow with a regular, reddish, transversal stripe on each abdominal segment (stripe reaching lateral margins of the segment and extended on connexiva). Each stripe is darker in the lateral portion (almost black), but the dark portion is not elongated on the abdominal stigmata area (Figure 26A).

**Structure**: Body medium-sized (19.5–20.1 mm), shiny with dull wings, with a shiny central portion of the posterior pronotal lobe.

**Head**: The second visible labial segment is about 1.4 times longer than the first. Visible labial segments with various sized (mostly medium-sized) erected setae. Scapus and pedicellus covered by scarce, short, erected setae. Basiflagellomerus and distiflagellomerus covered by very dense, relatively short, lying setae. Ocelli medium-sized, placed dorso-laterally on tubercles.

**Thorax**: Anterior pronotal lobe of pronotum deeply and widely hollowed in posterior portion. Lateral and anterior margins of anterior pronotal lobe as well as collar covered by medium-sized, scarce, and erected setae. Calli flat in dorsal view and depressed in frontal view (Figure 16A). Depressions are ovoid and elongated. Posterior pronotal lobe flattened in the middle with lateral margins covered by medium-sized, erected setae. Posterior margin of the posterior pronotal lobe is slightly curved. Humeral angles with rounded apices. Scutellum with central hollow and short setae. Trochanter, femur, and tibia with different sized setae (mostly medium-sized). Hemelytra long with only slightly curved anterior margin of the wing (a reason why the body seems to be slender), distinctly surpassing apex of abdomen. Basal cell distinctly smaller than the discal cell.

**Abdomen**: Ventrally with various sized (primarily long) setae, lateral portion of each segment wrinkled.

**Genitalia**: Male—Pygophore large, ovoid in dorsal view (Figure 32C). Parameres slender and slightly enlarged apically with various sized setae apically (Figure 32A,B). Pedicel short (Figure 32D,E). Endosomal struts of aedeagus long with enlarged, falciform apices (Figure 32F). Basal plate elongated, enlarged in the middle, with a relatively short basal plate bridge (Figure 32F). Dorsal phallothecal sclerite tongue-like (Figure 32F). Endosomal lobes with deeply depressed (all over the surface) and elongated areas on both sides containing elongated spines (Figure 32G).

Female—Styloids medium-sized, slender with a delicately enlarged apical portion (Figure 32H), covered by scarce, various-sized setae (in apical portion). Gonocoxite 8 is subquadrangular with scarce setae (Figure 32I). Gonapophyse 9 is subtriangular with rounded external margin (Figure 32I).

**Measurements**: Body length: 20.1 (19.5); maximum width of abdomen: 5.4 (5.1); head length: 3.0 (2.9); head width: 1.5 (1.4); length of anteocular portion: 0.75 (0.75); length of postocular portion: 1.5 (1.4); length of synthlipsis: 1.0 (1.0); interocellar distance: 0.8 (0.8); length of antennal segments I:II:III:IV: 7.3 (7.2):2.2 (2.1):—(1.8):—(–); length of labial segments I:II:III: 1.2 (1.2):1.6 (1.6):0.65 (0.6); maximum length of anterior pronotal lobe: 1.1 (1.0); maximum length of posterior pronotal lobe: 3.0 (2.9); maximum width of anterior pronotal lobe: 2.9 (2.7); maximum width of posterior pronotal lobe: 5.6 (5.3); length of scutellum: 1.6 (1.5); length of hemelytra: 14.8 (14.3).

**Figure 33:**
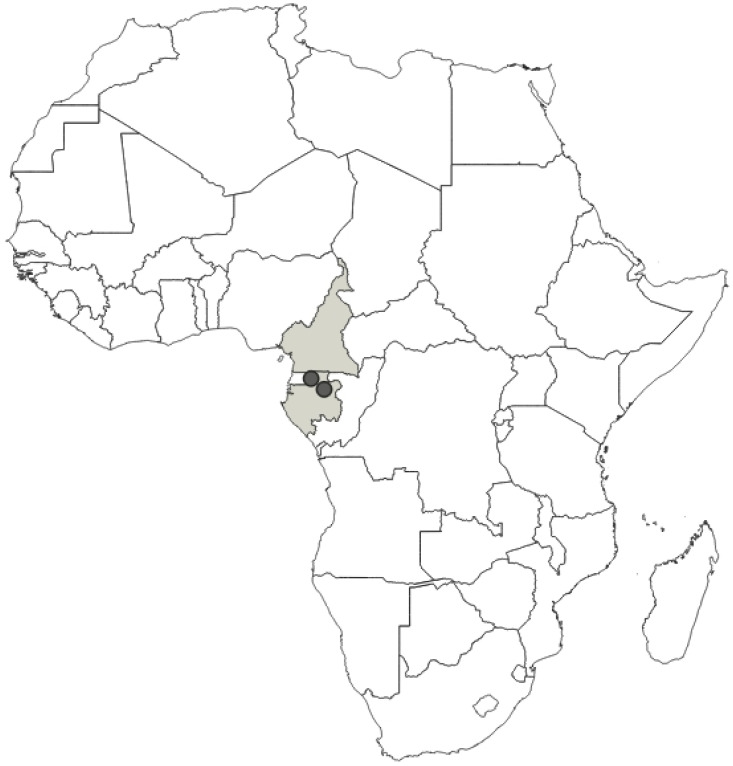
Known distribution of *Phonoctonus bifasciatus* Villiers, 1948.

**Distribution**: Republic of Cameroon, Gabonese Republic (Figure 33).

**Comments**: This species was described by Villiers [11], based on a male specimen deposited in MNHN, as the colour form of *P. fasciatus* (Beauvois, 1805). However, after examining the copulatory apparatus of males, we concluded that the two taxa are different at the species level, and hereby *P. bifasciatus* is elevated to the rank of a species. Despite the visible differences (in the species level) between *P. fasciatus* and *P. bifasciatus*, their previous status is mostly the result of a lack of examination of the genital structures, which were not illustrated in any of the original papers.

2. ***Phonoctonus caesar* Haglund, 1895** (Figure 4A, Figure 5G,I,J, Figure 6B, Figure 8B, Figure 16B, Figure 22, Figure 24L, Figure 25L, Figure 26B,C, Figure 28B,C, Figure 31B and Figure 34)

*Phonoctonus caesar* Haglund, 1895: 52:472. Syntype (♀): Cameroon; NHRS.

**Type material examined**: Lectotype (present designation): • Camerun.; Sjöstedt.; Phonoctonus/Caesar Hagl./♀ Typ!; Typus; NHRS-GULI/000000556 (NHRS) (Figure 31B).

Additional material examined—see Supplementary Materials S2.

**Diagnosis**: It can be easily recognised among other species in this genus by large (28.8–32.5 mm), robust body and red or reddish colouration of the head, pronotum, and corium.

Redescription

**Colour**: Body generally light—red, orange, and light brown with black patterns on head, legs, pronotum, hemelytra, and abdomen (Figure 28B,C).

**Head**: With two wide, black stripes from basal portions of antennifers to transversal sutura and black apices of antennifers, interocellar surface, labrum, ventral surface of first visible labial segment, second (except base) and third visible labial segments as well as the base of the neck (Figure 6B and Figure 8B). Scapus, pedicellus, and basiflagellomerus black. Distiflagellomerus dark brown.

**Thorax**: Collar of the anterior pronotal lobe is yellowish with black margins and red calli. Anterior pronotal lobe orange to red with black longitudinal sutura and lateral curved stripes (connected with sutura)—all black patterns of the anterior lobe of the pronotum are a trident like shape (Figure 16B). Transversal sutura of pronotum is black. Posterior pronotal lobe is orange with wide (wider in the middle portion), black transversal stripe with irregular margins. Lateral margins of posterior pronotal lobe is reddish. Prosternum black with orange margins and reddish stridulitrum. Meso- and metasternum with black lateral portions. Meso- and metepisternum black with yellowish margins. Mesoepimeron black. Scutellum black. Corium red or orange, laterally reddish. Clavus apically with a small, elongated black spot. Corium medially with a transversal, wide black stripe, connected with the black pattern along 1/3 of the posterior margin of the wing and basal portion of discal and basal cells. Black pattern on the discal cell base enlarged in an irregular spot (Figure 28B,C). Membrane brown. Coxa and trochanter red with orange apex. Femur black, basal and apical portion red. In some specimens, fore femur with thin, red lateral stripes connecting red basal and apical portions. Tibia black, apical and basal portion red or reddish. Tarsus black.

**Abdomen**: Abdomen ventrally red with the whitish or yellowish anterior portion of each segment and irregular black patterns on lateral sides of the abdominal segment (limited only to the posterior red area of each segment). Connexives red with whitish anterior and posterior margins (Figure 26B,C).

**Structure**: Body large (28.4–32.5 mm), dull, and relatively robust in both sexes.

**Head**: Postocular portion of the head covered by dense and relatively long setae. Second visible labial segment is about 1.35 times longer than the first. Visible labial segments have short, erected setae. Scapus covered by scarce, short setae (except club-shaped apical portion, covered by medium-sized, erected, and rather dense setae). Pedicellus covered by dense, medium-sized, erected setae. Basiflagellomerus and distiflagellomerus covered by very dense, rather short, lying setae. Ocelli medium-sized, placed dorso-laterally on small but distinct tubercles.

**Thorax**: Lateral and ventral portions visibly covered by very dense, medium-sized, semi-erected setae. Anterior pronotal lobe with deeply hollowed basal half of longitudinal sutura. Lateral and anterior margins of anterior pronotal lobe and collar covered by rather long, dense, and erected setae. Calli large with rounded apices and small depressions on the fore surface (Figure 16B). The posterior pronotal lobe flattened in the middle portion and delicately wrinkled near lateral margins. Lateral margins of posterior pronotal lobe covered by very short, erected setae. Posterior margin of posterior pronotal lobe straight. Humeral angles with rounded apices. Scutellum with distinct Y-shaped ridges. Trochanter, femur, and tibia with various sized, relatively short setae. Hemelytra robust and long, distinctly surpassing apex of abdomen. Basal and external cells similar in size or basal cell larger (females).

**Abdomen**: Ventrally with various sized setae, lateral portion of each segment wrinkled.

**Figure 34:**
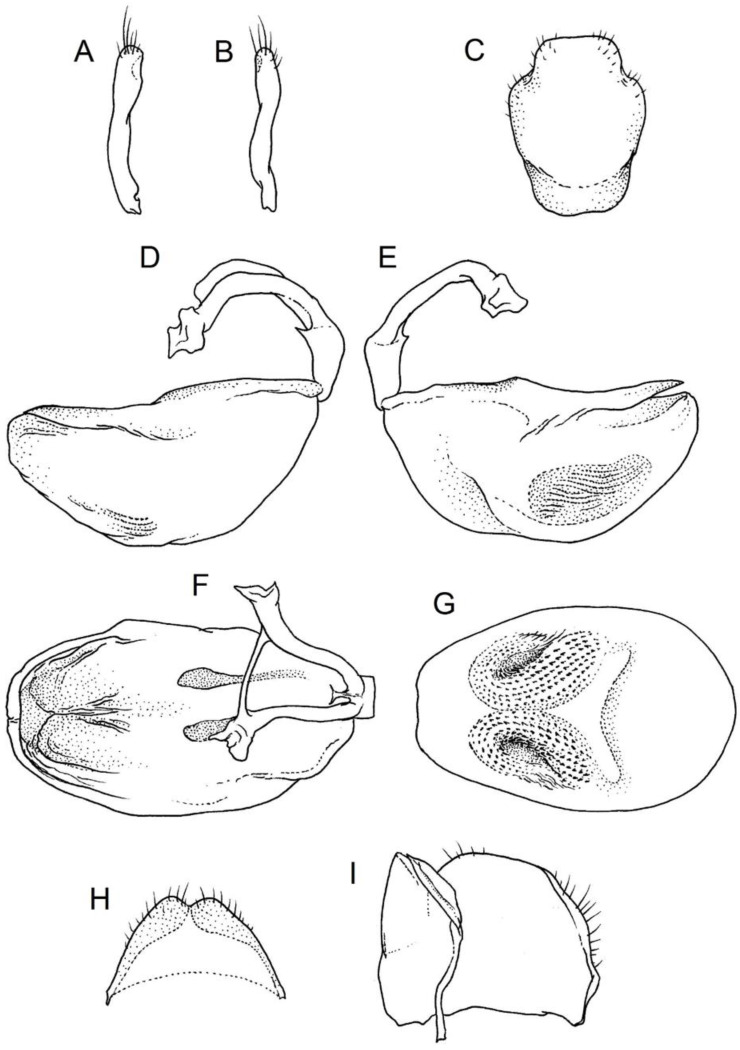
*Phonoctonus caesar* Haglund, 1895: (**A**) left paramere; (**B**) right paramere; (**C**) pygophore, ventral view; (**D**) aedeagus, right lateral view; (**E**) aedeagus, left lateral view; (**F**) aedeagus, dorsal view; (**G**) aedeagus, ventral view; (**H**) styloids, outer view; and (**I**) gonocoxite of abdominal segment VIII.

**Genitalia**: Male—Pygophore large, subquadrangular in dorsal view (Figure 34C). Parameres slender, delicately curved, sclerotised apically with distinctly visible long setae on apex (Figure 34A,B). Pedicel wide and medium-sized (Figure 34D,E). Endosomal struts of aedeagus relatively short with enlarged and elongated, crescent in shape apices (Figure 34F). Basal plate elongated, only slightly enlarged in the middle, with a relatively long basal plate bridge (Figure 34F). Dorsal phallothecal sclerite tongue-like, with divided apex (Figure 34F). Endosomal lobes with deeply depressed areas covered by short spines. Depressions elongated, places on dorsal portion (Figure 34G).

Female—Styloids, relatively large with distinctly club-shaped apical portions, covered by dense, medium-sized setae on the external margin (in apical portion) (Figure 34H). Gonocoxite 8 is ovoid with elongated basal portion and with various sized (primarily long) and dense setae (Figure 34I). Gonapophyse 9 is relatively large and triangular, without setae.

**Measurements**: Body length: 31.2–32.5 (28.4–28.8); maximum width of abdomen: 9.1–11.1 (8.3–9); head length: 4.2–4.5 (3.6–3.75); head width: 2.1–2.25 (1.9–1.95); length of anteocular portion: 1.1–1.4 (1.05–1.15); length of postocular portion: 1.7–2.05 (1.55–1.7); length of synthlipsis: 1.5 (1.35–1.4); interocellar distance: 1.0–1.1 (0.95); length of antennal segments I:II:III:IV: 8.1–8.6 (7.9–8.9):2.75–2.9 (2.65–2.7):3.55–4.0 (3.3–3.4):8.6–10.1 (8.2–9.5); length of labial segments I:II:III: 1.6–1.9 (1.6–1.75):2.2–2.45 (2.05–2.2):0.65–0.7 (0.65–0.7); maximum length of anterior pronotal lobe: 1.9 (1.6–1.8); maximum length of posterior pronotal lobe: 4.8–4.9 (4.2–4.3); maximum width of anterior pronotal lobe: 4.9–5.2 (4.3–4.4); maximum width of posterior pronotal lobe: 9.0–9.5 (7.9–8.2); length of scutellum: 2.4–3.2 (2.1–2.5); length of hemelytra: 23.2–23.6 (21.1–21.4).

**Figure 35:**
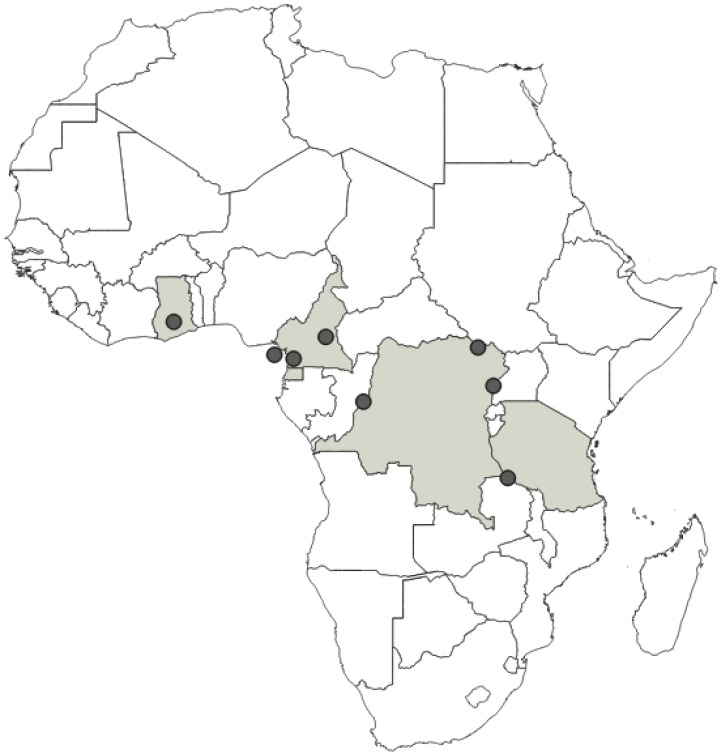
Known distribution of *Phonoctonus caesar* Haglund, 1895.

**Distribution**: Republic of Cameroon, Democratic Republic of the Congo, Equatorial Guinea (the Island of Bioko, formerly known as Fernando Po), Republic of Ghana, United Republic of Tanzania (Figure 35).

**Comments**: This species was described by Haglund [34] based on one female (information also confirmed in the original publication) labelled as a type (according to red label under the specimen), and we designated this specimen as a lectotype.

3. ***Phonoctonus elegans* Varela, 1904** (Figure 5F, Figure 6C, Figure 8C, Figure 16C, Figure 23A–D, Figure 24D, Figure 25D, Figure 26D, Figure 27A, Figure 28D, Figure 31C,D and Figure 36)

*Phonoctonus elegans* Varela, 1904: 4:56. Syntype (♂): Cameroon; MNCN.

*Phonoctonus elegans* var. *stricta* Schouteden, 1932: 1:201. Holotype (♂): Democratic Republic of the Congo: Bumbuli; RMCA.

**Type material examined**: Lectotype (present designation): • Phonoctonus/elegans/S. Var/Kamerun; Sintipo; MNCN/Cat. Tipos No./8352; MNCN_Ent/104115 (MNCN) (Figure 31C).

[for *P. elegans* var. *stricta*:] • HOLOTYPUS; Museé du Congo/Bumbuli/I-IV-1915/R. Mayné; R. Dét./J./2581; Phonoctonus elegans v. stricta Sch./Types (RMCA). • [7x] PARATYPUS; Museé du Congo/Bumbuli/I-IV-1915/R. Mayné; R. Dét./J./2581 (RMCA). • PARATYPUS; Museé du Congo Belge/Kasai: Hibo/(Don A. Shévy); R. Dét./J/2581 (RMCA). • PARATYPUS; Museé du Congo/Sankuru: Komi/23-IV-1930/J. Ghesquière; R. Dét./J/2581 (RMCA). • PARATYPUS; Museé du Congo/Ingende/24-XII-1914/R. Mayné; R. Dét./J./2581 (RMCA). • PARATYPUS; Museé du Congo/Bena Bendi/V-1915/R. Mayné; R. Dét./J./2581 (RMCA). • PARATYPUS; Museé du Congo/Equateur: Bokote/1-II-1927/R.P. Hulstaert; R. Dét./J./2581 (RMCA). • PARATYPUS; Museé du Congo/Sankuru: Lonkala/-III-1925/L^t^ J. Ghesquière; R. Dét./J./2581 (RMCA) (Figure 31D). • PARATYPUS; Museé du Congo/Sankuru: Yomi/VII-1930/J. Ghesquière; R. Dét./J./2581 (RMCA). • PARATYPUS; Museé du Congo/Sankuru: Lomela/-IV-1925/J. Ghesquière; R. Dét./J./2581 (RMCA).

Additional material examined—see Supplementary Materials S2.

**Diagnosis**: This species can be easily recognised by the following combination of characters: dark (often black) body with red head and black legs. Superficially resembles *P. fairmairei*, but is easily distinguished from it by the orange or red apical portion of the femur.

Redescription

**Colour**: Body generally dark—black with light head and patterns on pronotum, hemelytra, and abdomen (Figure 28D).

**Head**: Reddish, some specimens have a black falciform pattern directly before transversal sutura and dark longitudinal line on the postocular portion of the head (in some specimens extended on frons) (Figure 6C and Figure 8C). First visible labial segment is reddish, second and third visible labial segments are dark brown to black. Scapus black with a reddish basal portion. Pedicellus and basiflagellomerus black. Distiflagellomerus black with the yellow basal portion.

**Thorax**: Anterior and posterior pronotal lobes are black or dark brown, with yellowish margins (except transversal sutura) (Figure 16C). Light margins of pronotum extended laterally on the posterior pronotal lobe near transversal sutura. Prosternum dark brown to black with yellowish margins. Pro-, meso-, and metepisternum as well as pro- and mesoepimeron with yellowish or whitish patterns. Scutellum dark with pale lateral and apical portions. Corium dark brown or black with the pale basal portion, outer margin of clavus, costal margin, transversal stripe, and apex (Figure 28D). Membrane dark brown. Legs dark brown with reddish apices of femur and pale patterns on the coxa and trochanter.

**Abdomen**: Ventral portion of abdomen is red with whitish or yellowish anterior portion of each segment and irregular black patterns on the lateral sides of the abdominal segment (limited only to the posterior red portion of each segment). Connexives bicolorous whitish or yellowish and red (Figure 26D).

**Structure**: Body medium-sized (17.7–23.4 mm), dull and slender in both sexes.

**Head**: Thin setae on the head surface. First and second visible labial segments with medium-sized, erected setae. Third segment with short, erected setae. Antennal segments with short setae. Ocelli medium-sized, placed on very small tubercles.

**Thorax**: Anterior pronotal lobe with deeply hollowed basal portion of longitudinal sutura. Calli with small, rounded apices (Figure 16C). Posterior pronotal lobe with wide longitudinal, delicately hollowed line as well as large, delicate, lateral depressions in the medial portion. Surface of posterior pronotal lobe is delicately corrugated. Humeral angles with rounded apices. Scutellum with short, curved setae and corrugated lateral surfaces. Trochanter, femur, and tibia with various sized, rather short setae. Hemelytra is slender and long, distinctly surpassing the apex of the abdomen. Basal cell is slender and smaller than the discal cell.

**Abdomen**: Ventrally with medium-sized setae, lateral portion of each segment wrinkled.

**Figure 36:**
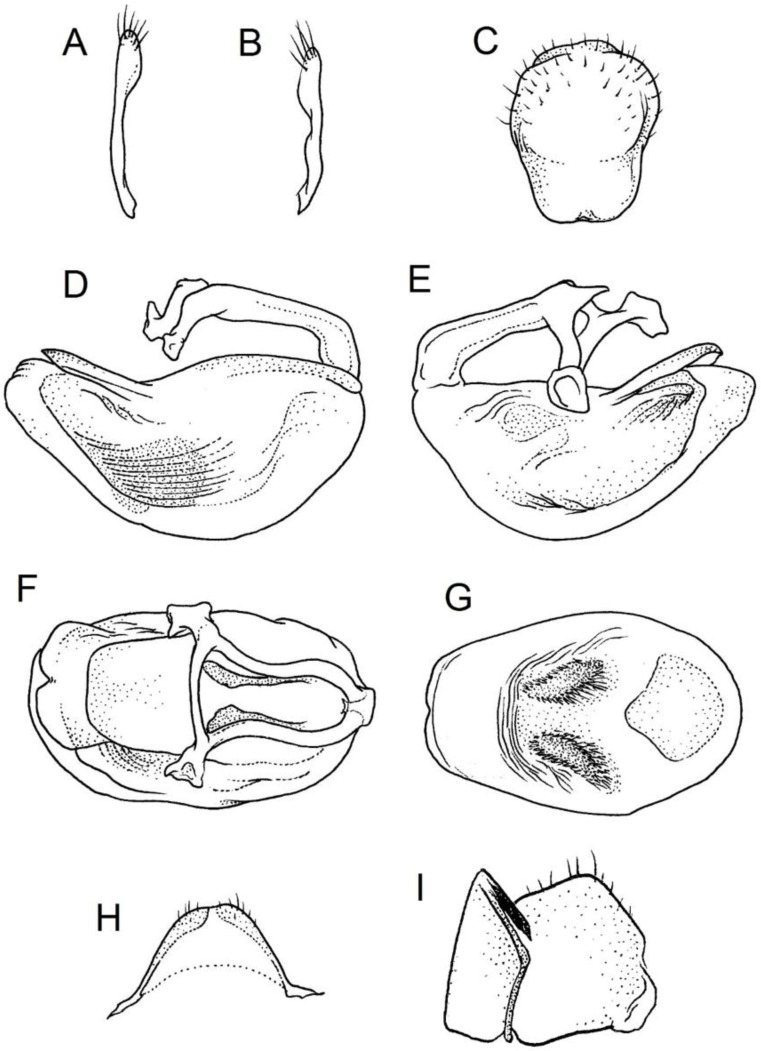
*Phonoctonus elegans* Varela, 1904: (**A**) left paramere; (**B**) right paramere; (**C**) pygophore, ventral view; (**D**) aedeagus, right lateral view; (**E**) aedeagus, left lateral view; (**F**) aedeagus, dorsal view; (**G**) aedeagus, ventral view; (**H**) styloids, outer view; and (**I**) gonocoxite of abdominal segment VIII.

**Genitalia**: Male—Pygophore slightly elongated in dorsal view (Figure 36C). Parameres are relatively thin, distinctly enlarged apically (club-shaped), with few relatively long setae on the apex (Figure 36A,B). Pedicel short and distinctly curved (Figure 36D,E). Endosomal struts of aedeagus are medium-sized with enlarged, rounded apices (Figure 36F). Basal plate elongated, with almost straight lateral margins, with a relatively short and thin basal plate bridge (Figure 36F). Dorsal phallothecal sclerite is tongue-like, with a convex apical portion (Figure 36F). Endosomal lobes have areas covered with relatively long, robust spines, deeply depressed on all areas covered by spines (Figure 36G).

Female—Styloids, medium-sized with distinctly club-shaped apical portions, covered by various sized (mostly medium-sized) setae on the apical portion (Figure 36H). Gonocoxite 8 is a trapeze-like shape with elongated basal portion and with various sized (primarily long) and scarce setae (Figure 36I). Gonapophyse 9 is relatively small (similar to styloids in size) and triangular with a bow like external margin.

**Measurements**: Body length: 20.4–23.4 (17.7–22.2); maximum width of abdomen: 5.1–6.4 (4.0–5.3); head length: 2.9–3.2 (2.6–3.1); head width: 1.4–1.6 (1.35–1.5); length of anteocular portion: 0.6–0.8 (0.6–0.7); length of postocular portion: 1.2–1.6 (1.0–1.5); length of synthlipsis: 1.0–1.2 (0.9–1.0); interocellar distance: 0.65–0.8 (0.5–0.8); length of antennal segments I:II:III:IV: 6.0–8.1 (6.8–7.8):2.1–2.8 (2.1–2.5):1.7–2.1 (1.7–2.1):9.3–12.9 (8.9–13.0); length of labial segments I:II:III: 1.2–1.4 (1.2–1.3):1.2–1.8 (1.4–1.7):0.5–0.7 (0.45–0.6); maximum length of anterior pronotal lobe: 1.1–1.35 (1.0–1.2); maximum length of posterior pronotal lobe: 2.9–3.1 (2.4–3.1); maximum width of anterior pronotal lobe: 2.8–3.1 (2.7–3.0); maximum width of posterior pronotal lobe: 5.4–6.0 (5.1–5.5); length of scutellum: 1.7–2.0 (1.5–2.0); length of hemelytra: 14.8–16.4 (13.3–15.9).

**Figure 37:**
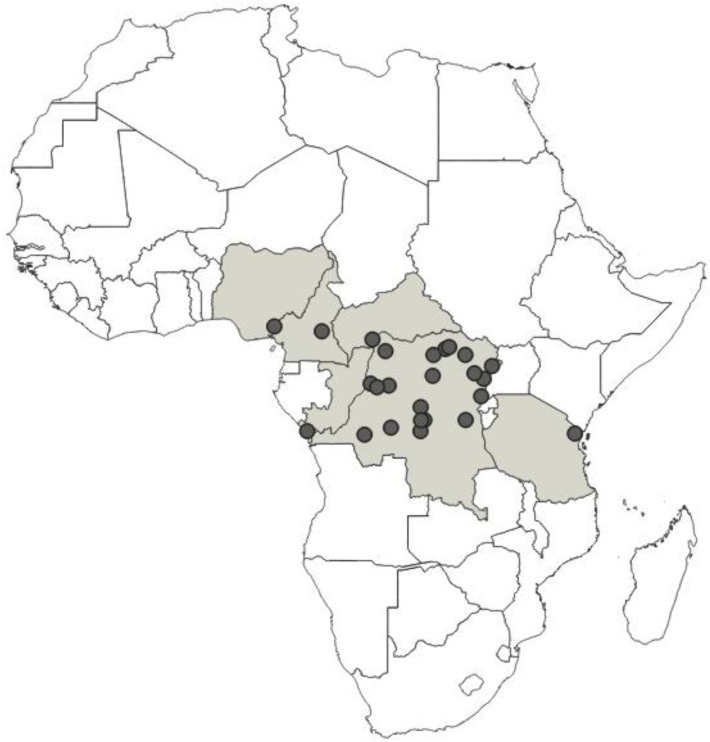
Known distribution of *Phonoctonus elegans* Varela, 1904.

**Distribution**: Republic of Cameroon, Central African Republic, Democratic Republic of the Congo, Federal Republic of Nigeria, Republic of the Congo, United Republic of Tanzania (Figure 37).

**Comments**: This species was originally described based on one female labelled as a syntype (according to red label under the specimen), and we designated this specimen as a lectotype.

4. ***Phonoctonus fairmairei* Villiers, 1948** (Figure 3B,D,E, Figure 6D, Figure 8D, Figure 16D,E, Figure 24E, Figure 25E, Figure 26E, Figure 28E, Figure 31E and Figure 38) **stat. nov.**

*Phonoctonus fasciatus* var. *fairmairei* Villiers, 1948: 9:124. Holotype (♀): Congo: Brazzaville; MNHN.

**Type material examined**: • Juillet; Museum Paris/Congo/Brazzaville/Mission Chari-Tchad/Dr J. Decorse 1904; HOLOTYPE (MNHN) (Figure 31E).

Additional material examined—see Supplementary Materials S2

**Diagnosis**: This species can be easily recognised by the following combination of characteristics: dark body (often black) with red head and black legs. Superficially resembles *P. elegans*, but is easily distinguished by whole black femurs.

Redescription

**Colour**: Body generally dark—black with pale head and patterns on pronotum, hemelytra, and abdomen (Figure 28E).

**Head**: Red with dark spot on the posterior portion of the postocular portion or with dark neck (some specimens with yellowish maxillary and mandibular plates). Apical portion of clypeus and labrum black. Apical portion of first visible labial segment is red (except dorsal surface), second and third visible labial segments are dark brown to black (Figure 6D and Figure 8D). Antennomeres black. Scapus with reddish basal portion, distiflagellomerus with yellow basal portion.

**Thorax**: Anterior pronotal lobe red or brown, posterior pronotal lobe dark brown to black; anterior and posterior pronotal lobes with yellow margins (except transversal sutura or anterior margin of posterior lobe also yellow) (Figure 16D,E). Calli yellow or whitish, in some specimens reddish in frontal view. Proepisternum yellow with black middle portion, proepimeron yellow with brown patterns or brown with small yellow patterns. Meso- and metathorax red to dark brown, with yellow or whitish mesoepisternum, mesoepimeron, and metepisternum. Scutellum black with pale lateral and apical portions. Corium dark brown to black with pale basal portion, 2/3 of basal portion or only outer margin of clavus, wide transversal stripe, and apical portion (Figure 28E). Membrane black. Legs dark brown or dark brown with red coxa (some specimens with red fore coxa and brown middle and hind coxa).

**Abdomen**: Ventral portion of abdomen is red with yellow or whitish anterior and posterior portion of each segment, and with irregular black patterns on the lateral sides of the abdominal segment (limited only to the posterior red area of each segment; on VI and VII abdominal segments, black lateral patterns can be connected on each segment by black line crossing near posterior margin). Connexives red (Figure 26E).

**Structure**: Medium-sized (19.7–26.0 mm), body dull and slender in both sexes.

**Head**: With thin setae. First and second visible labial segments with various sized, erected, and relatively dense setae. Antennomeres covered by short setae, except pedicellus with relatively long erected setae. Ocelli medium-sized, placed on very small tubercles. The postocular portion of the head is elevated.

**Thorax**: Anterior pronotal lobe with deeply hollowed basal portion of longitudinal sutura. Anterior pronotal lobe visibly gibbous in the lateral view. Calli with small, rounded apices, frontally flattened and with distinct depression (Figure 16D,E). Posterior pronotal lobe visibly wrinkled transversally with a distinctly curved posterior portion. Posterior margin of posterior pronotal lobe is delicately curved. Humeral angles with rounded apices. Trochanter, femur, and tibia with various sized, relatively short setae. Hemelytra slender, surpassing apex of abdomen. Basal cell is delicately smaller or similar in size to the distal cell.

**Abdomen**: Ventrally with medium-sized setae, lateral portion of each segment wrinkled.

**Figure 38:**
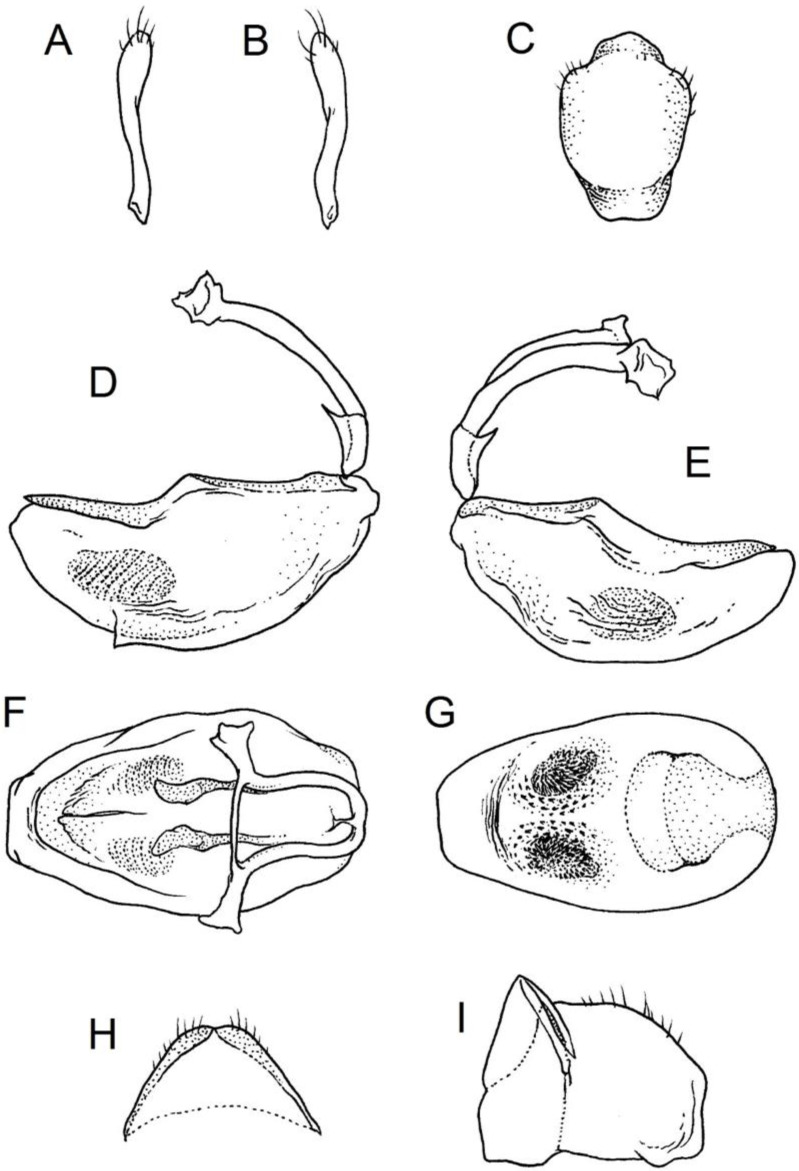
*Phonoctonus fairmairei* Villiers, 1948: (**A**) left paramere; (**B**) right paramere; (**C**) pygophore, ventral view; (**D**) aedeagus, right lateral view; (**E**) aedeagus, left lateral view; (**F**) aedeagus, dorsal view; (**G**) aedeagus, ventral view; (**H**) styloids, outer view; and (**I**) gonocoxite of abdominal segment VIII.

**Genitalia**: Male—Pygophore ovoid and relatively short in dorsal view (Figure 38C). Parameres are relatively long and club-shaped with various sized setae on the apex (Figure 38A,B). Pedicel short (Figure 38D,E). Endosomal struts of aedeagus long with enlarged and curved apices. Apices with distinctly visible, small nodule on the inner margin (Figure 38F). Basal plate elongated, with robust margins, with a relatively short and thin basal plate bridge (Figure 38F). Dorsal phallothecal sclerite is tongue-like, with a very delicately convex apex (Figure 38F). Endosomal lobes with areas covered by relatively long, robust spines, deeply depressed on all area covered by spines (Figure 38G).

Female—Styloids, medium-sized and relatively thin with distinctly club-shaped apical portions, covered by dense, relatively long setae on the apical half (Figure 38H). Gonocoxite 8 is subquadrangular and with various sized (primarily long) and scarce setae (Figure 38I). Gonapophyse 9 is relatively small (similar to styloids in size) and triangular with a bow like external margin.

**Measurements**: Body length: 22.1–26.0 (19.7–20.3); maximum width of abdomen: 6.2–8.5 (6.0–6.1); head length: 2.9–3.6 (2.7–2.9); head width: 1.5–1.8 (1.5–1.55); length of anteocular portion: 0.6–0.8 (0.5–0.7); length of postocular portion: 1.5–1.7 (1.4–1.5); length of synthlipsis: 1.1–1.3 (1.0–1.05); interocellar distance: 0.7–0.9 (0.75–0.8); length of antennal segments I:II:III:IV: 6.2–8.8 (5.9–8.2):2.1–2.7 (2.0–2.4):1.8–2.7 (1.8–2.2):9.0–11.6 (7.8–9.1); length of labial segments I:II:III: 1.1–1.4 (1.1–1.3):1.6–1.7 (1.55–1.6):0.5–0.6 (0.5); maximum length of anterior pronotal lobe: 1.35–1.4 (1.15–1.2); maximum length of posterior pronotal lobe: 3.5–3.8 (2.95–3.0); maximum width of anterior pronotal lobe: 3.5–3.6 (3.1–3.15); maximum width of posterior pronotal lobe: 6.3–6.6 (5.6–5.65); length of scutellum: 1.6–1.7 (1.5–1.55); length of hemelytra: 15.9–18.4 (14.3–14.5).

**Figure 39:**
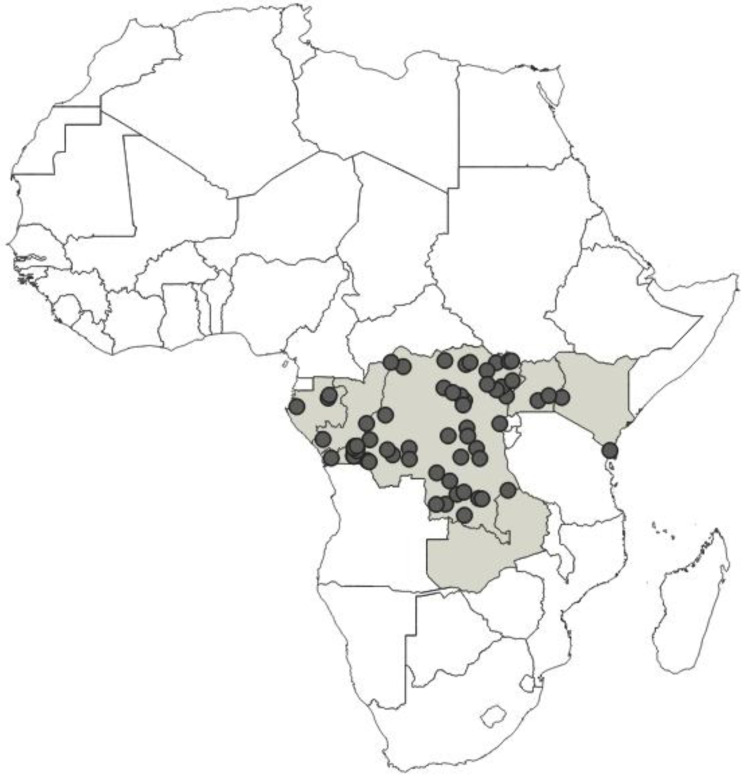
Known distribution of *Phonoctonus fairmairei* Villiers, 1948.

**Distribution**: Democratic Republic of the Congo, Gabonese Republic, Republic of Kenya, Republic of the Congo, Republic of Uganda, Republic of Zambia (Figure 39).

**Comments**: This species was described as a colour form of *P. fasciatus* by Villiers [11], who synonymised *P. fairmairei* with *P. fasciatus*. After examining the male copulatory apparatus (which has not been illustrated in any of the original papers) of specimens, we concluded that the two taxa are different at the species level. Hereby, *P. fairmairei* is elevated to the rank of a species. Despite the visible differences (in the species level) between the mentioned species, their previous status is mainly due to a lack of examination of the genital structures.

5. ***Phonoctonus fasciatus* (Beauvois, 1805)** (Figure 5H, Figure 6E, Figure 7, Figure 8E, Figure 9, Figure 10, Figure 11B,C, Figure 12A,C, Figure 13, Figure 15E,F, Figure 16F, Figure 17B,D, Figure 18, Figure 19, Figure 20, Figure 21, Figure 24A, Figure 25A, Figure 26F, Figure 28F, Figure 29B and Figure 40).

*Reduvius fasciatus* Beauvois, 1805: 65. Holotype (♂): Oware.

*Evagoras fasciatus* Schaum, 1862: 49.

*Phonoctonus fasciatus* Stål, 1865: 3:63.

*Harpactor fasciatus* Walker, 1873: 8:108.

**Type material examined**: Neotype (present designation): • R. fasciatus/Pal. de Bauv./Guinea; Mus. Westerm; ♂; Neotype [red label] (ZMUC).

Additional material examined—see Supplementary Materials S2.

**Diagnosis**: This species can be easily recognised by the following combination of characteristics: red head, brown legs, anterior pronotal lobe reddish with yellowish margins (Figure 28F); corium greyish marked in middle part by black, thin, transversal stripe with irregular margins, fused with black transversal and oblique stripe connected with the membrane (Figure 28F); black transversal stripe visible on apical portion of corium; aedeagus with membranous struts of endosoma covered by relatively long and robust spines.

Redescription

**Colour**: Body generally pale—greyish with reddish, brown, and black patterns (Figure 28F).

**Head**: Red with orange transversal sutura and basal portion of clypeus, brown labrum. Antennae (except red basal portion of scapus), apices of antennifers, apex of first visible labial segment, second and third visible labial segments are black.

**Thorax**: Collar of anterior pronotal lobe yellowish with black margins and reddish apices of calli (Figure 6E and Figure 8E). Anterior pronotal lobe reddish with yellowish margins (Figure 16F). Transversal sutura of pronotum is reddish. Posterior pronotal lobe greyish with relatively thin, brown transversal stripe near the posterior margin. Lateral portions of the posterior pronotal lobe are reddish (reddish area elongated on humeral angles of pronotum). Prosternum is red. Proepisternum is whitish. Mesopleuron red with dark, irregular spots near the anterior and posterior margin. Posterior margin of mesopleuron is whitish. Meso- and metepisternum as well as metacoxal cavity is whitish. Scutellum is red with dark lateral margins and a pale apex. Corium is greyish with a yellowish apical portion. Apex of the clavus is darker. In the middle portion of the corium (anterior margin on the height of the apex of clavus), there is a black and thin transversal stripe with irregular margins, and fused with a black transversal and oblique stripe connected with the membrane. Apical portion of the corium has a black transversal stripe (on the height of the middle portion of basal cell) (Figure 28F). Membrane brown. Coxa red. Trochanter, femur, tibia, and tarsus brown.

**Abdomen**: Ventral portion of the abdomen is red with a whitish anterior and posterior margin of each segment. Whitish areas on the ventral side of the abdomen is wider in lateral portions and with distinct margins. Connexives are red with a whitish inner, anterior corner (Figure 26F).

**Structure**: Body medium-sized (17.9–20.1 mm), dull with a shiny central portion of posterior pronotal lobe and wing venation.

**Head**: Second visible labial segment about 1.4 times longer than the first. Visible labial segments with medium-sized erected setae. Scapus and pedicellus covered by scarce, short, erected setae. Basiflagellomerus and distiflagellomerus covered by very dense, relatively short, lying setae. Ocelli medium-sized, placed dorso-laterally on distinctly visible tubercles.

**Thorax**: Anterior pronotal lobe with deeply hollowed basal half of longitudinal sutura. Lateral and anterior margins of anterior pronotal lobe and collar covered by rather long, dense, and erected setae. Calli medium-sized, anteriorly flattened with ovoid depressions and small, rounded apices (Figure 16F). Middle portion of posterior pronotal lobe flattened with lateral margins covered by various sized, erected setae. Posterior margin of posterior pronotal lobe straight. Humeral angles with rounded apices. Scutellum with a central hollow. Trochanter, femur, and tibia with different sized setae (mostly medium-sized). Hemelytra long, distinctly surpassing the apex of the abdomen. Basal cell is smaller than the discal cell.

**Abdomen**: Ventrally with various sized setae, lateral portion of each segment wrinkled.

**Figure 40:**
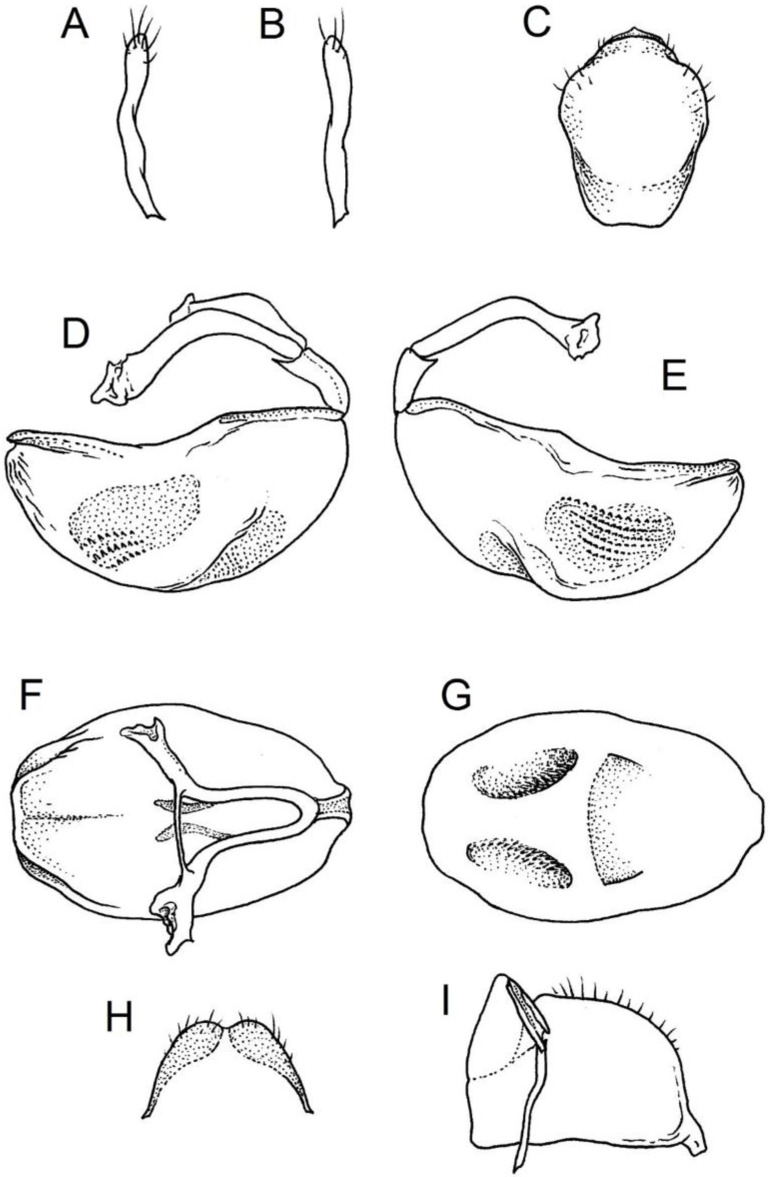
*Phonoctonus fasciatus* (Beauvois, 1805): (**A**) left paramere; (**B**) right paramere; (**C**) pygophore, ventral view; (**D**) aedeagus, right lateral view; (**E**) aedeagus, left lateral view; (**F**) aedeagus, dorsal view; (**G**) aedeagus, ventral view; (**H**) styloids, outer view; and (**I**) gonocoxite of abdominal segment VIII.

**Genitalia**: Male—Pygophore ovoid in dorsal view (Figure 40C). Parameres relatively robust and flattened, slightly curved, and spoon-shaped (Figure 40A,B). Pedicel short and wide (Figure 40D,E). Endosomal struts of the aedeagus are thin with distinctly enlarged apices (Figure 40F). Basal plate elongated v-shaped, with robust margins, with a relatively short basal plate bridge (Figure 40F). Dorsal phallothecal sclerite is tongue-like with delicately divided apex (Figure 40F). Endosomal lobes have areas covered by relatively long, robust spines, deeply depressed. (Figure 40G).

Female—Styloids, relatively large and robust with distinctly club-shaped apical portions, covered by short setae on the external margin (Figure 40H). Gonocoxite 8 is rectangular with various sized, rather scarce setae (Figure 40I). Gonapophyse 9 is relatively small and triangular.

**Measurements**: Body length: 17.9–20.1 (18.2–19.0); maximum width of abdomen: 4.6–5.5 (4.2–4.5); head length: 2.8–2.9 (2.7–2.9); head width: 1.4–1.5 (1.4–1.5); length of anteocular portion: 0.6–0.7 (0.65–0.7); length of postocular portion: 1.4–1.5 (1.3–1.4); length of synthlipsis: 1.0–1.1 (0.95–1.0); interocellar distance: 0.75–0.8 (0.7–0.8); length of antennal segments I:II:III:IV: 5.6–6.2 (5.4–6.0):1.9–2.2 (1.8–1.9):1.8–2.2 (1.8–2.1):6.5–9.1 (6.3–8.2); length of labial segments I:II:III: 1.1–1.2 (1.1–1.2):1.4–1.5 (1.4–1.5):0.5 (0.5); maximum length of anterior pronotal lobe: 1.2–1.3 (1.1–1.2); maximum length of posterior pronotal lobe: 2.8–3.0 (2.7–2.8); maximum width of anterior pronotal lobe: 3.0–3.2 (2.95–3.0); maximum width of posterior pronotal lobe: 5.3–5.7 (5.2–5.3); length of scutellum: 1.4–1.6 (1.4–1.6); length of hemelytra: 13.2–14.4 (12.8–13.8).

**Figure 41:**
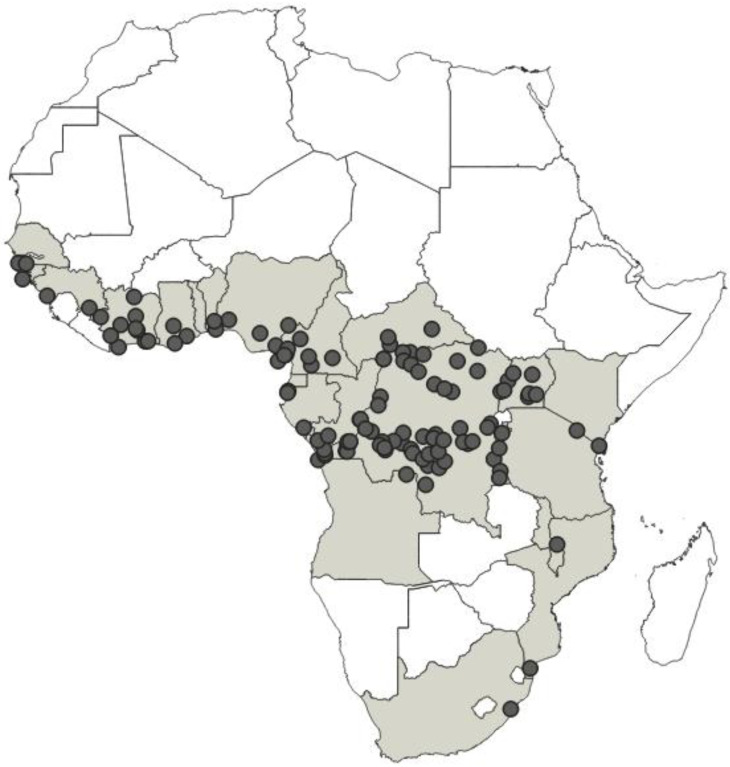
Known distribution of *Phonoctonus fasciatus* (Beauvois, 1805).

**Distribution**: Republic of Angola, Republic of Burundi, Republic of Benin, Republic of Cameroon, Central African Republic, Democratic Republic of the Congo, Republic of the Congo, Equatorial Guinea (the Island of Bioko, formerly known as Fernando Po), Gabonese Republic, Republic of Ghana, Republic of Guinea, Republic of Guinea-Bissau, Republic of Côte d’Ivoire (Ivory Coast), Republic of Kenya, Republic of Malawi, Republic of Mozambique, Federal Republic of Nigeria, Republic of Senegal, Republic of South Africa, United Republic of Tanzania, Togolese Republic, Republic of Uganda (Figure 41).

**Comments**: *Phonoctonus* was described by Stål [10] based on a species—*Reduvius fasciatus* named by Palisot de Beauvois [25]; however, both descriptions are very brief and inadequate. Since the original description of *R. fasciatus* (Beauvois, 1805), the type material of this species was not re-examined. The syntype/s were probably never designated or were lost, as the authors did not find these specimens in any entomological collection. Such information was also confirmed by Stål [52]. However, during our studies, we found a specimen from the collection of Palisot de Beauvois, from Guinea which did not belong to the original type series (type locality—Oware (currently Nigeria)), and which hereby is designated as a neotype under Articles 75.3.1 and 75.3.4 of the International Code of Zoological Nomenclature (4th edition, 1999) for clarifying uncertainties in the identification of this species and for fixing its identity, moreover, for solving the nomenclatural problem between *P. fasciatus* and other species (mostly treated as its colour variations by previous authors).

6. ***Phonoctonus grandis* Signoret, 1860** (Figure 6F, Figure 8F, Figure 16G, Figure 24K, Figure 25K, Figure 26G, Figure 28G, Figure 31F and Figure 42).

*Phonoctonus grandis* Signoret, 1860: 8:962 [published in 1861]. Holotype (♀): Mayotta Island; NHRS.

*Harpactor grandis* Walker, 1873, 8:108.

**Type material examined**: Lectotype (present designation): • [♀] Madagasc./Coll. Signoret; grandis/det. Signoret; SYNTUPUS/Phonoctonus/grandis Signoret. 1860/etik. Hecher 1996/REDV. 316/1 (NHMW). Paralectotype (present designation): • [♀] Madagasc./Coll. Signoret; grandis/det. Signoret; SYNTUPUS/Phonoctonus/grandis Signoret. 1860/etik. Hecher 1996/REDV. 316/2 (NHMW) (Figure 31F). Paralectotype (present designation): • [♀] Madagasc./Coll. Signoret; grandis/det. Signoret; SYNTUPUS/Phonoctonus/grandis Signoret. 1860/etik. Hecher 1996/REDV. 316/3 (NHMW).

Additional material examined—see Supplementary Materials S2.

**Diagnosis**: The only species of genus *Phonoctonus* distributed in Madagascar, Comoros Islands, and Seychelles, very easily recognised by a red head with black antenna, red anterior pronotal lobe, collar, posterior pronotal lobe, and orange corium (with black, transversally elongated patterns). Superficially resembles *P. principalis*, but is easy to distinguish by lack of a transversally elongated stripe on the posterior pronotal lobe and darker legs.

Redescription

**Colour**: Body generally pale—orange with black patterns on legs, hemelytra, and abdomen (Figure 28G).

**Head**: Red or orange with darker neck. First and basal half of second visible labial segments, labrum, antennae, and apical half of second and whole third visible labial segments are red. Black margins of clypeus (Figure 6F and Figure 8F).

**Thorax**: Anterior pronotal lobe of pronotum orange or red with paler collar (Figure 16G). Transversal sutura of pronotum black or partially black (middle portion). Posterior pronotal lobe orange with more intensive colour on the posterior and lateral areas. Prosternum is orange or red. If the prosternum is orange, the proepisternum is red (paralectotype); if the prosternum is red, the proepimeron is orange (lectotype). Mesosternum is orange with reddish lateral portions or is entirely red. Metasternum is orange with reddish middle portion of the posterior margin or entirely red. Mesoepisternum, mesoepimeron, metepisternum, and metacoxal cavity is yellowish. Meso- and metapleuron have a distinct, black, vertical line. Scutellum is orange. Corium is orange with black, transversal patterns, placed in the middle portion of the corium (anterior margin of the pattern below the apex of clavus) and not connected with the fore and hind margin of the wing. Black longitudinal pattern between patterns is described above along the PCu vein and connected with a membrane. The black transversal stripe is visible on the apical portion of the corium (on the height of the middle portion of the basal cell) (Figure 28G). Membrane is dark brown. Coxa and trochanter are red. Fore and middle femur are red with a black ventral surface. Hind femur is black with a red apical and basal portion. Tibia is dark brown to black, gradually paler into the basal portion, and the basal portion is reddish. Tarsus is black.

**Abdomen**: Ventral portion of abdomen is orange with the red posterior portion of each segment and black posterior margin of each segment (black colour of posterior margin presents only 2/3 of the middle portion of margin; red area is wider in lateral portions of each segment). Connexives are red with orange anterior margins (Figure 26G).

**Structure**: Body medium-sized (18.8–24.1 mm), dull with shiny pronotum.

**Head**: Second visible labial segment is 1.26 times longer than the first. Visible labial segments with various sized (primarily long), erected setae. Scapus covered by scarce short setae, longer on the apical portion. Pedicellus covered by very dense, medium-sized, semi-erected setae. Basiflagellomerus and distiflagellomerus covered by very dense, short, lying setae. Ocelli relatively small, placed on distinct tubercles.

**Thorax**: Anterior pronotal lobe of the pronotum with deeply hollowed basal 1/3 longitudinal sutura. Lateral and anterior margins of the anterior pronotal lobe and collar covered by rather long, dense, and erected setae. Calli large, flattened anteriorly with large globular apices and distinct, large ovoid depressions on the fore surface (Figure 16G). Posterior pronotal lobe gibbous and wide. Posterior margin of posterior pronotal lobe slightly curved. Humeral angles with rounded apices. Scutellum with very distinct Y-shaped ridges. Trochanter, femur, and tibia with various sized, relatively short setae. Hemelytra long, distinctly surpassing the apex of the abdomen. Basal cell is smaller than the discal cell.

**Abdomen**: Ventrally with various sized setae, distinctly wrinkled on the lateral portion of each segment.

**Figure 42:**
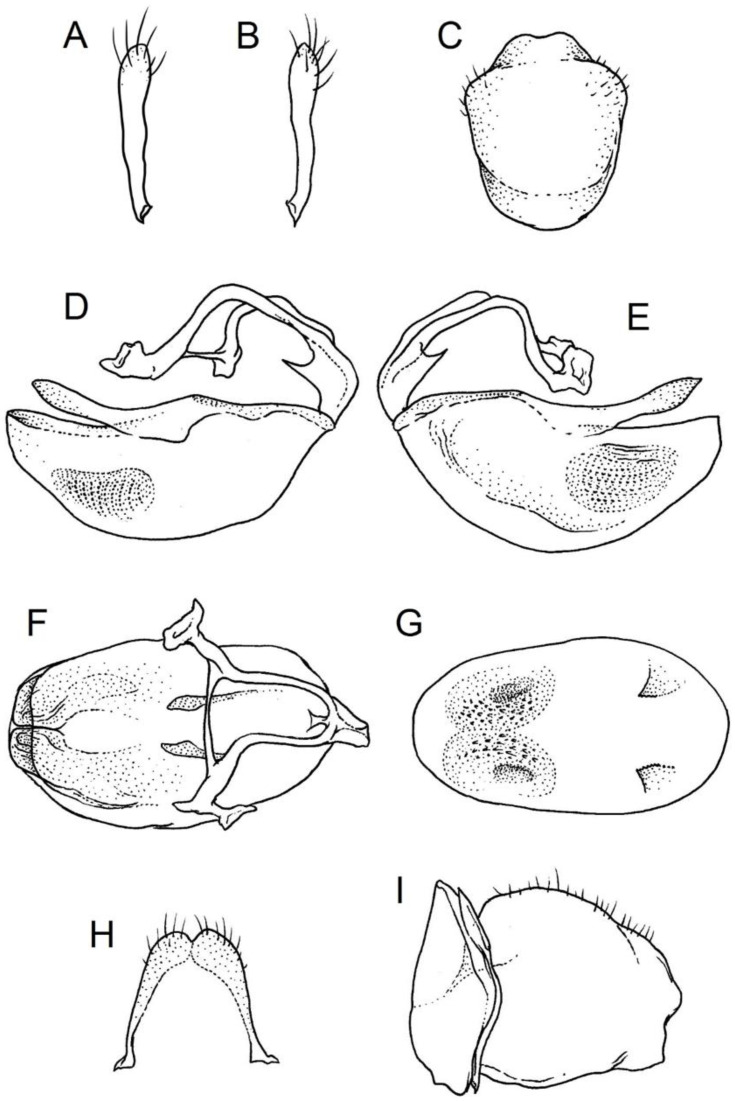
*Phonoctonus grandis* Signoret, 1860: (**A**) left paramere; (**B**) right paramere; (**C**) pygophore, ventral view; (**D**) aedeagus, right lateral view; (**E**) aedeagus, left lateral view; (**F**) aedeagus, dorsal view; (**G**) aedeagus, ventral view; (**H**) styloids, outer view; and (**I**) gonocoxite of abdominal segment VIII.

**Genitalia**: Male—Pygophore slightly elongated in dorsal view (Figure 42C). Parameres relatively robust and flattened, slightly curved with different sized setae on the apex (Figure 42A,B). Pedicel relatively long and distinctly curved at about 90 degrees, wide (Figure 42D,E). Endosomal struts of aedeagus long with enlarged, subtriangular apices. Subtriangular apices with a distinctly visible process on the inner margin (Figure 42F). Basal plate elongated, wide, with robust margins, with a long basal plate bridge (Figure 42F). Dorsal phallothecal sclerite is tongue-like, with divided apex (Figure 42F). Endosomal lobes with areas covered by short, robust spines, deeply depressed in a thin line near the dorsal margin. Both areas are connected longitudinally on the ventral side (Figure 42G).

Female—Styloids, relatively large and robust on all lengths, covered by very dense and long setae in the apical portion (Figure 42H). Gonocoxite 8 is quadrangular with rather long and dense setae (Figure 42I). Gonapophyse 9 is subtriangular in shape.

**Measurements**: Body length: 21.0–24.1 (18.8–20.8); maximum width of abdomen: 5.2–6.0 (4.2–5.5); head length: 3.0–3.3 (2.8–2.9); head width: 1.6–1.65 (1.5–1.6); length of anteocular portion: 0.75–0.9 (0.6–0.65); length of postocular portion: 1.4–1.5 (1.4); length of synthlipsis: 1.1 (1.0); interocellar distance: 0.8–0.9 (0.8–0.85); length of antennal segments I:II:III:IV: 5.0–5.5 (4.8): 2.0–2.1 (1.9–2.1): 2.2–2.3 (2.1–2.4): 5.6–6.1 (5.6); length of labial segments I:II:III: 1.1–1.2 (1.2): 1.5–1.6 (1.5–1.6): 0.5 (0.5); maximum length of anterior pronotal lobe: 1.5 (1.3); maximum length of posterior pronotal lobe: 3.4–3.7 (2.7–3.3); maximum width of anterior pronotal lobe: 3.3–3.5 (2.8–3.2); maximum width of posterior pronotal lobe: 6.4–7.0 (5.2–6.2); length of scutellum: 1.8–1.9 (1.5–1.6); length of hemelytra: 14.9–17.0 (13.4–15.1).

**Figure 43:**
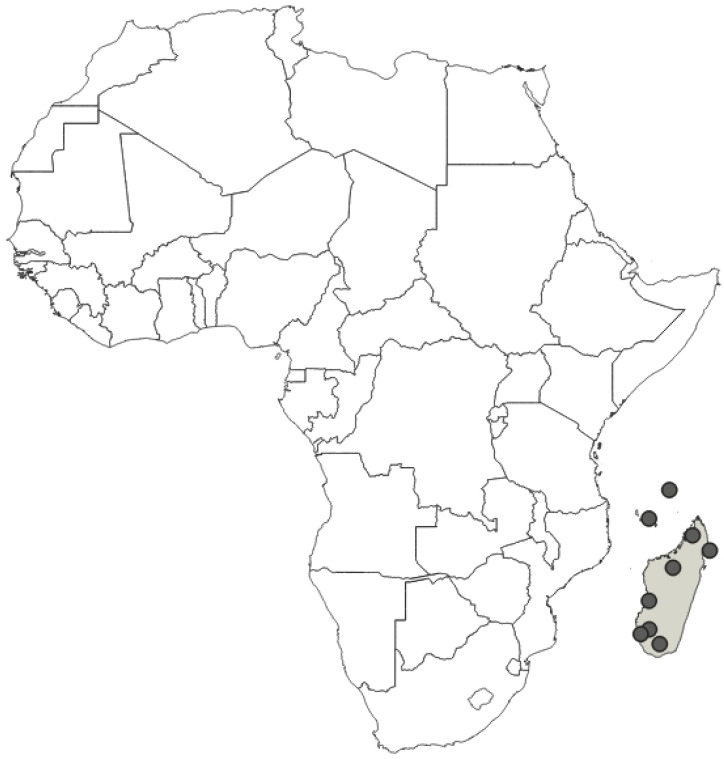
Known distribution of *Phonoctonus grandis* Signoret, 1860.

**Distribution**: Seychelles (Aldabra), Union of the Comoros (Anjouan and Mayotta), Madagascar (Figure 43).

**Comments**: All examined type specimens of this species, deposited in NHMW, are described as syntypes (according to the labels). There is no information in the original paper of Signoret [37] on the number of type specimens, and all examined specimens were from the original collection of Signoret. We considered these specimens as syntypes, and designated these specimens as a lectotype and paralectotypes. In collections, this species is often confused with *P. principalis*. However, *P. principalis* is found in mainland Africa and *P. grandis* is found only in Madagascar, the Comoros Islands (Anjouan and Mayotta), and Seychelles (Aldabra).

7. ***Phonoctonus immitis* Stål, 1865** (Figure 5C, Figure 6G, Figure 8G, Figure 12B, Figure 16H,I, Figure 23E, Figure 24B, Figure 25B, Figure 26H,I, Figure 28H,I, Figure 29C, Figure 31G,H and Figure 44) **stat. rev.**

*Phonoctonus immitis* Stål, 1865: 3:62. Syntypes (♂, ♀): Guinea; NHRS.

*Phonoctonus subimpictus* Stål, 1865: 3:63. Syntypes (♂, ♀): Guinea; NHRS.

*Phonoctonus immitis* var. *subimpictus* Stål, 1874: 4:21.

**Type material examined**: Lectotype (present designation): [♀] Guinea/NHRS-GULI; 000006531 (NHRS). • [♂] immitis Stål/NHRS-GULI; 000006447 (NHRS) (Figure 31G). Lectotype (present designation) [for *P. subimpictus*]: • [♂] SYNTYPUS/Phonoctonus subimpictus Stål, 1865/etik. Hecher 1996/REDV. 315/1; Guinea/Coll. Signoret; subimpictus/det. Stål (NHMW). Paralectotype (present designation): • [♀] Old-Ca-/labar./Stål; subimpictus Stål; Typus; NHRS-GULI/000000558 (NHRS) (Figure 31H). • [♀] Guinea/NHRS-GULI; 000006530 (NHRS).

Additional material examined—see Supplementary Materials S2.

**Diagnosis**: This species can be easily recognised by the following combination of characteristics: greyish body with lack of spots on corium or with very thin, dark transversal line in the middle; distiflagellomerus with pale basal portion; femur dark, tibia pale (Figure 28H,I).

Redescription

**Colour**: Body generally pale—greyish with a black spot on the head, dark antennae and legs (Figure 28H,I).

**Head**: Grey, light brown or yellowish with darker postocular portion and large black spot on the postocular area. Labrum and antennae (except yellow basal portion of distiflagellomerus) are black. Second and third visible labial segments are dark brown.

**Thorax**: Collar of anterior pronotal lobe and lateral margins of anterior pronotal lobe are yellow (Figure 6G and Figure 8G). Anterior pronotal lobe brownish (Figure 16H,I). Transversal sutura of pronotum in the middle portion black (some specimens with black anterior margin of the anterior pronotal lobe). Posterior pronotal lobe is greyish with yellowish lateral and posterior margins. Prosternum is yellowish. Proepisternum is yellow with a black, wide vertical line, proepimeron with a brown anterior margin. Mesopleuron is yellowish with dark irregular pattern on the anterior portion and posterior margin. Mesoepisternum is yellow, mesoepimeron is black (prolongated dark pattern from the posterior margin of mesopleuron). Metepisternum is yellow. Scutellum is greyish with distinctly wrinkled lateral margins. Corium is greyish with a paler costal margin (Figure 28H,I). Membrane is brown, coxa yellow, and the trochanter yellowish with a dark ventral surface. Femur brown. Tibia and tarsus dark brown to black.

**Abdomen**: Ventral portion of the abdomen is greyish or yellowish with a black transversal line near the posterior margin of III–VI abdominal segments (in some specimens, abdominal segments are red with yellow anterior and posterior portion of each segment, and on segments III–VI posterior margin of the red area is limited by a black line along the margin). Connexives are red with a whitish anterior margin (Figure 26H,I).

**Structure**: Body medium-sized (19.2–22.5 mm), dull.

**Head**: Head with thin setae. Second visible labial segment is about 1.15 times longer than the first. Visible labial segments with various sized, erected setae. Scapus covered by scarce, short, semi-erected setae. Pedicellus is covered by dense, medium-sized, erected setae. Basiflagellomerus and distiflagellomerus covered by very dense, short, lying setae. Ocelli medium-sized, placed on distinct tubercles.

**Thorax**: Anterior pronotal lobe with deeply hollowed 1/3 basal portion of longitudinal sutura. Lateral and anterior margins of the anterior pronotal lobe and collar covered by relatively short, dense, and erected setae. Calli large with rounded apices and small, rounded depressions on the fore surface (Figure 16H,I). Posterior pronotal lobe gibbous with lateral margins of the posterior pronotal lobe covered by short, erected setae. Posterior margin of the posterior pronotal lobe is straight. Humeral angles with rounded apices. Scutellum with very wide Y-shaped ridges. Trochanter, femur, and tibia with different sized setae. Hemelytra distinctly surpass the apex of the abdomen. Basal cell is visibly smaller than the discal cell.

**Abdomen**: Ventrally with various sized setae, lateral portion of each segment wrinkled.

**Figure 44:**
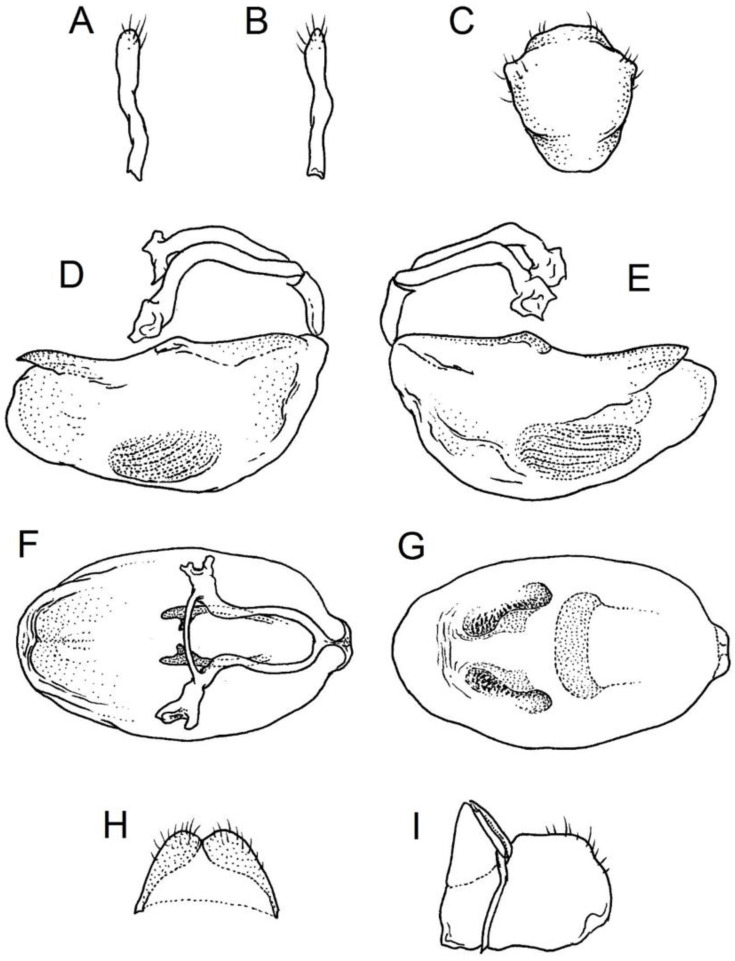
*Phonoctonus immitis* Stål, 1865: (**A**) left paramere; (**B**) right paramere; (**C**) pygophore, ventral view; (**D**) aedeagus, right lateral view; (**E**) aedeagus, left lateral view; (**F**) aedeagus, dorsal view; (**G**) aedeagus, ventral view; (**H**) styloids, outer view; and (**I**) gonocoxite of abdominal segment VIII.

**Genitalia**: Male—Pygophore ovoid in dorsal view (Figure 44C). Parameres are relatively robust, slightly curved with relatively long setae on apex (Figure 44A,B). Pedicels are medium-sized, wide and distinctly curved (Figure 44D,E). Endosomal struts of aedeagus are long with divided apices (Figure 44F). Basal plate elongated and narrow with medium-sized margins and relatively long basal plate bridge (Figure 44F). Dorsal phallothecal sclerite is tongue-like with a rounded apex (Figure 44F). Endosomal lobes with areas covered by short, robust spines, deeply depressed (all over the area) and kidney like shape (Figure 44G).

Female—Styloids, relatively large and robust with distinctly club-shaped apical portions and apically covered by dense, medium-sized setae (Figure 44H). Gonocoxite 8 is subquadrangular with elongated basal portion and various sized, scarce setae (Figure 44I). Gonapophyse 9 is relatively large and subtriangular in shape.

**Measurements**: Body length: 20.9–22.5 (19.2–22.1); maximum width of abdomen: 6.0–6.7 (5.1–5.7); head length: 3.0–3.3 (2.75–3.0); head width: 1.5–1.65 (1.35–1.65); length of anteocular portion: 0.75–0.8 (0.65–0.75); length of postocular portion: 1.25–1.6 (1.1–1.35); length of synthlipsis: 1.1–1.2 (1.05–1.2); interocellar distance: 0.7–0.9 (0.75.–0.8); length of antennal segments I:II:III:IV: 6.3–7.7 (6.4–7.0): 2.1–2.4 (2.1–2.2): 2.2–2.8 (2.15–2.5): 7.95–12.4 (8.6–11.2); length of labial segments I:II:III: 1.25–1.5 (1.2–1.4): 1.55–1.8 (1.65–1.7): 0.5–0.6 (0.55–0.6); maximum length of anterior pronotal lobe: 1.2–1.4 (1.0–1.3); maximum length of posterior pronotal lobe: 3.2–3.4 (2.8–3.2); maximum width of anterior pronotal lobe: 3.3–3.55 (2.9–3.25); maximum width of posterior pronotal lobe: 5.8–6.3 (5.3–5.95); length of scutellum: 1.5–1.8 (1.5–1.8); length of hemelytra: 14.9–15.9 (13.65–16.2).

**Figure 45:**
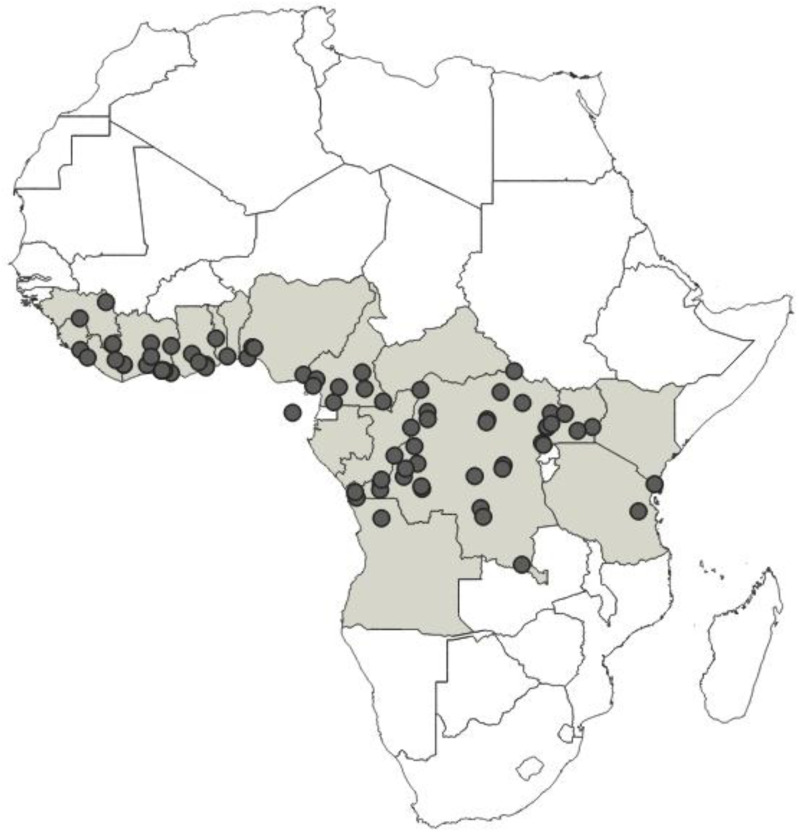
Known distribution of *Phonoctonus immitis* Stål, 1865.

**Distribution**: Republic of Angola, Republic of Benin, Republic of Cameroon, Central African Republic, Democratic Republic of the Congo, Republic of the Congo, Gabonese Republic, Republic of Ghana, Republic of Guinea, Republic of Côte d’Ivoire (Ivory Coast), Republic of Kenya, Republic of Liberia, Federal Republic of Nigeria, Democratic Republic of São Tomé and Príncipe, Republic of Sierra Leone, United Republic of Tanzania, Togolese Republic, Republic of Uganda (Figure 45).

**Comments**: *Phonoctonus immitis* and *P. subimpictus* were described simultaneously by Stål in 1865 [52]. In this situation, the First Reviser has the freedom to decide which of the two names to consider the valid name of the species—the act of selecting one of the two simultaneously published names is a First Reviser Act (Article 24.2). Stål conducted the First Reviser Act in 1874 when he downgraded *P. subimpictus* to a variety of *P. immitis*. Therefore, he effectively synonymised the two species, and he selected *P. immitis* as the valid name, and *P. subimpictus* is its junior synonym. All examined type specimens of *P. subimpictus* and *P. immitis*, deposited in NHMW and NHRS, are described as a syntype or type (according to labels), we considered those specimens as syntypes, and we designated those specimens as a lectotype and paralectotypes.

8. ***Phonoctonus luridus* Miller, 1950** (Figure 5E,F,J, Figure 6H, Figure 8H, Figure 14, Figure 15A,B, Figure 16J, Figure 24H, Figure 25H, Figure 26J, Figure 27B–F, Figure 30A, Figure 46A and Figure 47)

*Phonoctonus luridus* Miller, 1950: 3:504. Syntype (♀): Kenya: Uchweni Forest; NHMUK.

**Figure 46:**
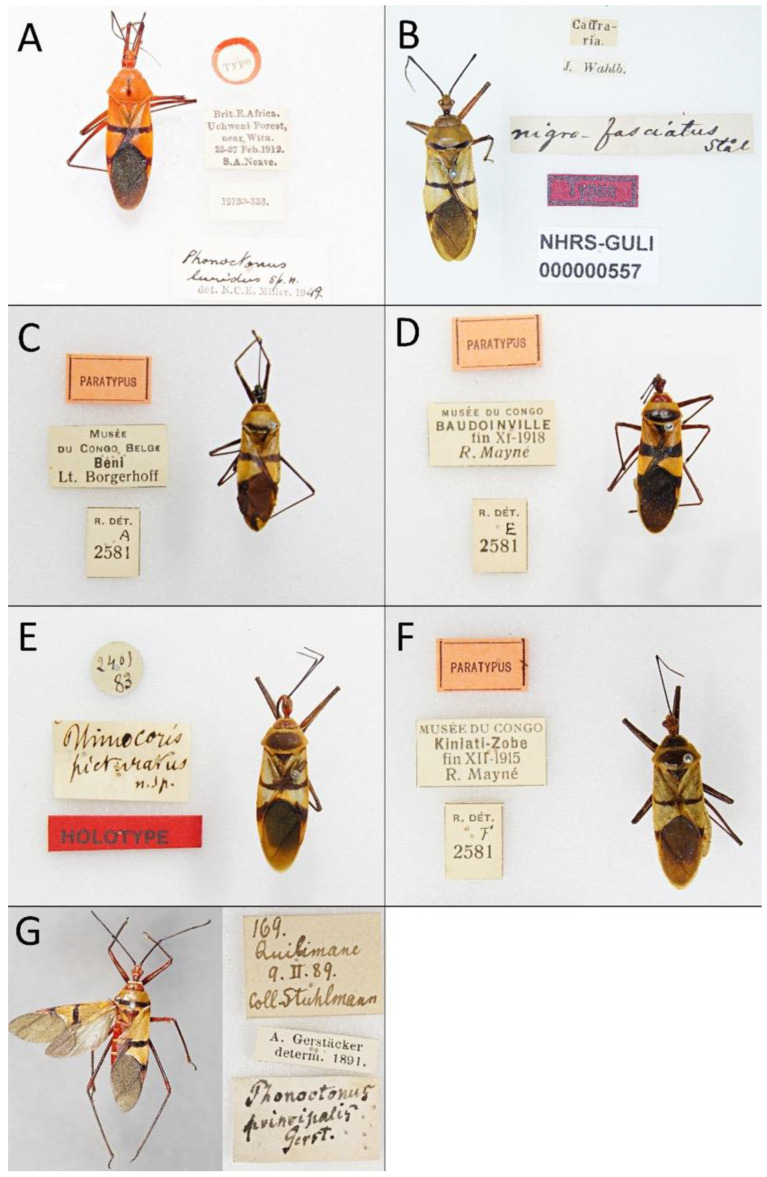
Type specimens of the representatives of *Phonoctonus* Stål, 1853: (**A**) *P. luridus* Miller, 1950; (**B**) *P. nigrofasciatus* Stål, 1855; (**C**) *P. fasciatus* var. *poultoni* Villiers, 1953; (**D***) P. picta* Schouteden, 1932; (**E**) *P. picturatus* Fairmaire, 1858; (**F**) *P. fasciatus* var. *discalis* Schouteden, 1932; and (**G**) *P. principalis* Gerstaecker, 1892.

**Type material examined**: Lectotype (present designation): • [♀] TYPE; Brit.E.Africa/Uchweni Forest,/near Witu./25–27 Feb.1912./S.A.Neave.; 1912-333.; Phonoctonus/luridus sp. n./det. N.C.E. Miller. 1949 (NHMUK) (Figure 46A).

Additional material examined—see Supplementary Materials S2.

**Diagnosis**: This species can be easily recognised by the following combination of characteristics: red or orange head; orange pronotum with yellow collar; posterior pronotal lobe lack of transversally elongated stripe; at least basal portion of femurs red (Figure 30A). Superficially resembles *P. grandis*, but is smaller and easy to distinguish by red femurs and a different pattern on the corium (transversal stripe running through both wings, while *P. grandis* has two unconnected strips) (Figure 28G and Figure 30A).

Redescription

**Colour**: Body generally pale—orange and red with black patterns (Figure 30A).

**Head**: Orange or red with red neck. All antennal segments are black, except the pale base of distiflagellomerus. First visible labial segment is red, second and third visible labial segments are brown (Figure 6H and Figure 8H).

**Thorax**: Collar of anterior pronotal lobe is yellowish with a red frontal surface (Figure 6H and Figure 8H). Anterior pronotal lobe is orange or reddish, lateral margins are paler (Figure 16J). Transversal sutura of the pronotum is reddish. Posterior pronotal lobe is orange. Prosternum is red with paler posterior portion. Meso- and metasternum are orange with yellowish middle portions. Propleuron is red with yellowish anterior and posterior margins. Meso- and metapleuron red with yellowish and dark patterns. Meso- and metepisternum are yellowish. Scutellum is orange. Corium is orange with a black transversal stripe reaching the costal vein and black vertical stripe connected with the transversal stripe and membrane (Figure 30A). Wide, black stripe on apical portion of corium placed on the base of discal cell (as wide as discal cell base). Membrane is dark brown. Coxa and trochanter are red (trochanter is darker). Fore, middle, and hind femur are brown with at least red basal portion. Tibia and tarsus are brown.

**Abdomen**: Ventral portion of the abdomen is red with the whitish or yellowish anterior and posterior portion of each segment. Connexives are red (Figure 26J).

**Structure**: Body medium-sized (21.2–25.2 mm), dull.

**Head**: Head with dense, medium-sized setae on anteocular portion. Second visible labial segments are 1.35 times longer than the first. Visible labial segments with medium-sized erected setae. Scapus and pedicellus are covered by scarce, short setae, except the club-shaped apical portion, covered by medium-sized, erected and relatively dense setae. Pedicellus is covered by dense, medium-sized, erected setae. Basi- and distiflagellomerus are covered by very dense, short, lying setae. Ocelli small, placed on small tubercles.

**Thorax**: Anterior pronotal lobe with a deep, wide and rounded hollow on the basal half of longitudinal sutura. All surfaces of the anterior pronotal lobe as well as the collar are covered by long, dense, and erected setae. Calli medium-sized with small, rounded apices and small, rounded, and distinct hollows on the fore surface (Figure 16J). The posterior pronotal lobe flattened in the middle portion and delicately depressed near lateral margins (in the middle portion). Anterior and lateral margins of the posterior pronotal lobe covered by relatively long, erected setae. Posterior margin of the posterior pronotal lobe curved inwardly. Humeral angles with rounded apices. Scutellum shiny with dull lateral margins. Trochanter, femur, and tibia with various sized (mostly medium-sized) setae. Hemelytra long, distinctly surpassing apex of abdomen with outwardly curved costal margin. Basal cell is smaller than the discal cell.

**Abdomen**: Ventrally with various sized setae, lateral portion of each segment wrinkled.

**Figure 47:**
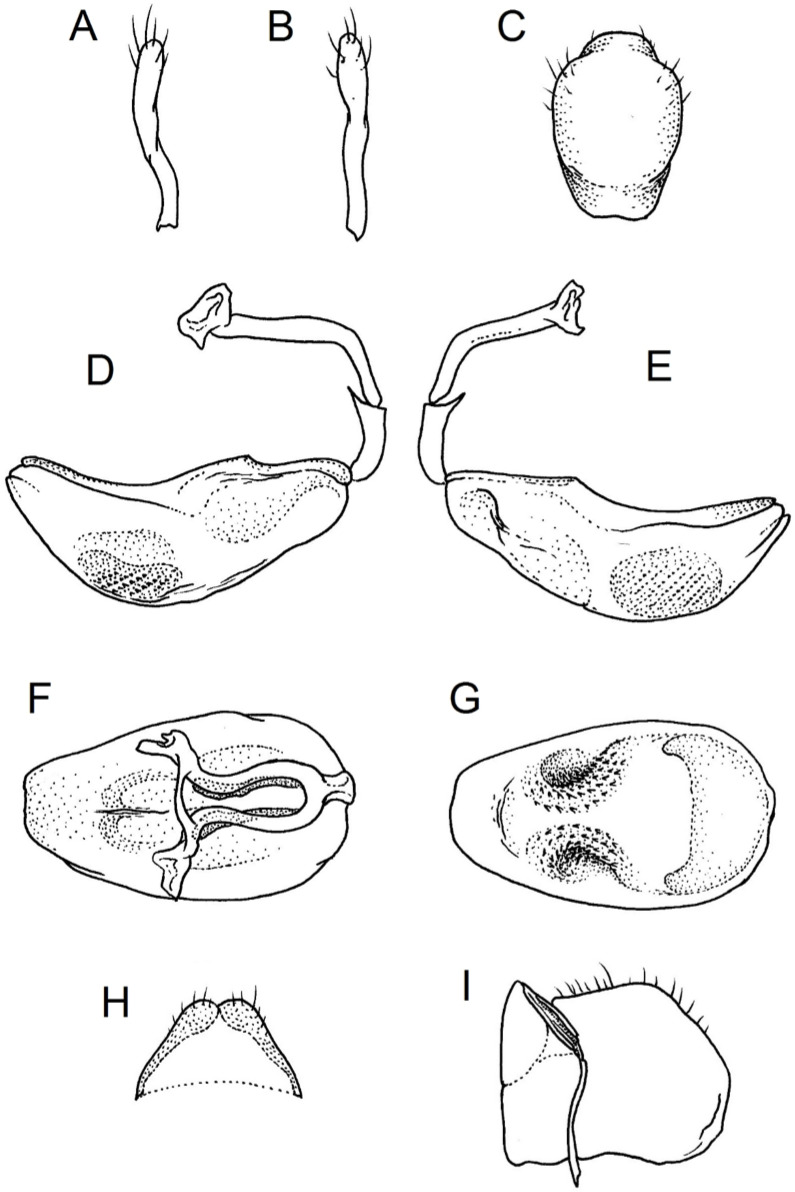
*Phonoctonus luridus* Miller, 1950: (**A**) left paramere; (**B**) right paramere; (**C**) pygophore, ventral view; (**D**) aedeagus, right lateral view; (**E**) aedeagus, left lateral view; (**F**) aedeagus, dorsal view; (**G**) aedeagus, ventral view; (**H**) styloids, outer view; and (**I**) gonocoxite of abdominal segment VIII.

**Genitalia**: Male—Pygophore elongated in dorsal view (Figure 47C). Parameres are relatively robust, slightly curved with relatively long and scarce setae on the apex (Figure 47A,B). Pedicel short, wide and only delicately curved (Figure 47D,E). Endosomal struts of the aedeagus are long with enlarged and elongated, spoon-like apices (Figure 47F). Basal plate is narrow and elongated, with very robust margins, placed very close to each other, with a relatively short and distinctly curved basal plate bridge (Figure 47F). Dorsal phallothecal sclerite is tongue-like, with a delicately curved inward apex (Figure 47F). Endosomal lobes have areas covered by long, robust spines that are deeply depressed. Depressions are elongated and placed on the middle portion of areas, wider apically (Figure 47G).

Female—Styloids, relatively small and thin with distinctly club-shaped apical portions, covered by dense, medium-sized and few very long setae in apical portion (Figure 47H). Gonocoxite 8 is subquadrangular with elongated basal portion and with various size (mostly medium-sized) and scarce setae (Figure 47I). Gonapophyse 9 is relatively large (larger than styloids) and triangular.

**Measurements**: Body length: 23.3–25.2 (21.2–21.3); maximum width of abdomen: 6.3–7.5 (5.2–5.5); head length: 3.2–3.4 (3.0–3.2); head width: 1.6–1.65 (1.5–1.6); length of anteocular portion: 0.8–0.95 (0.75–0.8); length of postocular portion: 1.4–1.65 (1.5–1.7); length of synthlipsis: 1.25 (1.1); interocellar distance: 1.0 (0.85–0.9); length of antennal segments I:II:III:IV: 6.6 (6.5): 2.2 (1.8): 2.3 (2.0): 9.6 (–); length of labial segments I:II:III: 1.35–1.4 (1.2–1.3): 1.6 (1.6): 0.55–0.6 (0.5–0.6); maximum length of anterior pronotal lobe: 1.45–1.5 (1.3–1.5); maximum length of posterior pronotal lobe: 3.6–3.8 (3.0–3.2); maximum width of anterior pronotal lobe: 3.7–3.8 (3.4–3.5); maximum width of posterior pronotal lobe: 6.8–7.0 (6.1–6.2); length of scutellum: 1.65–1.7 (1.6); length of hemelytra: 16.7–17.2 (14.9–15.2).

**Figure 48:**
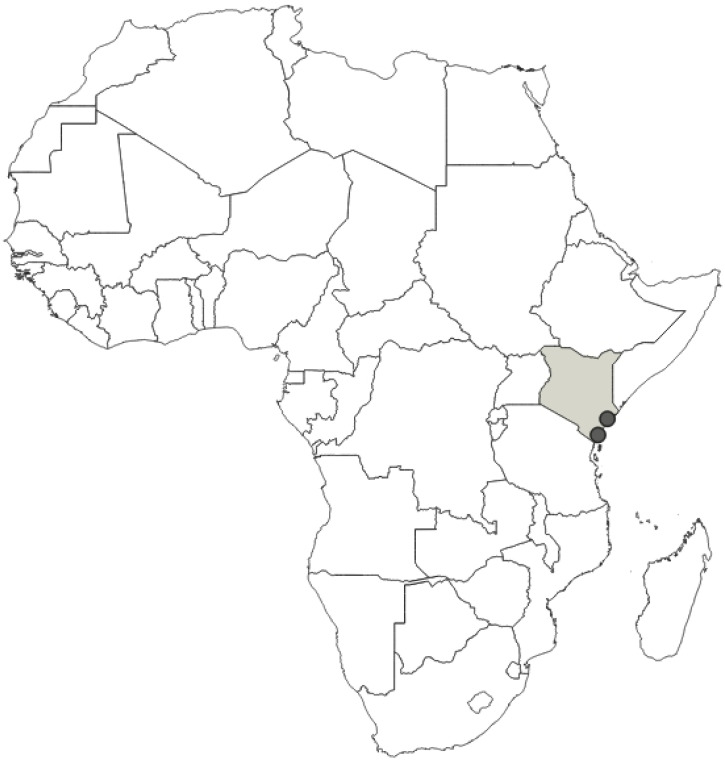
Known distribution of *Phonoctonus luridus* Miller, 1950.

**Distribution**: Republic of Kenya (Figure 48).

**Comments**: An examined type specimen (female) of this species, deposited in NHMUK, is described as a type (according to the labels and Miller’s original paper). We considered this specimen as a syntype, and designated it as a lectotype.

9. ***Phonoctonus lutescens* (Guérin-Méneville & Percheron, 1834)** (Figure 5B,K, Figure 6I, Figure 8I, Figure 11A, Figure 16K, Figure 23E,F, Figure 24I, Figure 25I, Figure 26K,L, Figure 29A, Figure 30B and Figure 49)

*Rhynocoris lutescens* Guérin-Méneville & Percheron, 1834: 8:962. Senegal.

*Phonoctonus lutescens* Stål, 1874: 4:21.

**Type material examined**: Neotype (present designation): • Guinea Portoghese/Bolama/VI-XII.1899 L Fea; Museum Civ./Genova; Neotype [red label] (MNHN).

Additional material examined—see Supplementary Materials S2.

**Diagnosis**: This species can be easily recognised by the following combination of characteristics: large body; head, scapus, legs, and anterior lobe of pronotum are red; large, rounded, black spot in the middle of the corium (Figure 30B).

Redescription

**Colour**: Body generally pale—red and light greyish with black patterns on pronotum, thorax, and hemelytra (Figure 30B).

**Head**: Red. Scapus and basal portion of pedicellus are red. Apical portion of pedicellus, basiflagellomerus, and distiflagellomerus is black.

**Thorax**: Collar of anterior pronotal lobe is yellowish with red ovoid depressions (Figure 6I and Figure 8I). Anterior pronotal lobe is red with brown anterior margin (brown tripe elongated also on lateral portions of anterior pronotal lobe), lateral margins are pale (Figure 16K). Transversal sutura of pronotum is dark. Posterior pronotal lobe is greyish with black anterior margin and wide, arcuate, black transversal stripe in the posterior portion. Anterior portion of the propleuron is dark brown, posterior portion is red. Proepisternum is whitish, proepimeron is dark brown. Prosternum is red. Meso- and metasternum are red with yellowish medial portions. Mesopleuron is red with dark brown anterior margin and black posterior margin. Mesoepisternum is whitish with dark anterior margin, mesoepimeron is black. Metapleuron is whitish with a brown medial portion. Scutellum is dark brown with black lateral portions and a reddish apex. Corium is greyish with a relatively large, rounded, black spot on the medial portion. Black pattern on an apical portion of the corium on the height of the base of the discal cell (Figure 30B). Membrane is brown. Coxa, trochanter, femur, tibia, and tarsus are red.

**Abdomen**: Ventral portion of the abdomen is red with a yellow anterior portion of each segment and black patterns on the posterior margin of the second visible abdominal segment. Connexives are red with yellow anterior margins (Figure 26K,L).

**Structure**: Body medium-sized (20.1–24.6 mm), dull and relatively slender in both sexes.

**Head**: Short setae on anteocular portion and long on postocular portion. Second visible labial segments are about 1.3 times longer than the first. All visible labial segments have various sized, erected setae. Scapus covered by regularly arranged, short and erected setae (except club-shaped apical portion, covered by medium-sized, erected, and dense setae). Pedicellus, basiflagellomerus, and distiflagellomerus covered by dense, short, and lying setae. Ocelli small-sized, placed dorso-laterally on laterally elongated, large, distinct tubercles.

**Thorax**: Anterior pronotal lobe with distinct longitudinal sutura and distinct rectangle area on the middle portion of posterior margin limited by sculpturation. Lateral and anterior margins of anterior pronotal lobe and collar covered by rather long, dense, and erected setae. Calli large with globular apices and small ovoid depressions on fore surface (Figure 16K). Posterior pronotal lobe flattened in lateral portions. Lateral margins of posterior pronotal lobe covered by medium-sized, erected setae. Posterior margin of the posterior pronotal lobe is almost straight (slightly curved in w-shaped). Humeral angles with rounded apices. Scutellum with very distinct Y-shaped ridges. Trochanter, femur, and tibia with various sized, dense setae. Hemelytra long, distinctly surpassing the apex of the abdomen; costal margin curved inwardly. Basal cell is the same size or slightly smaller than the discal cell.

**Abdomen**: Ventrally with various sized setae; lateral portion of each segment wrinkled.

**Figure 49:**
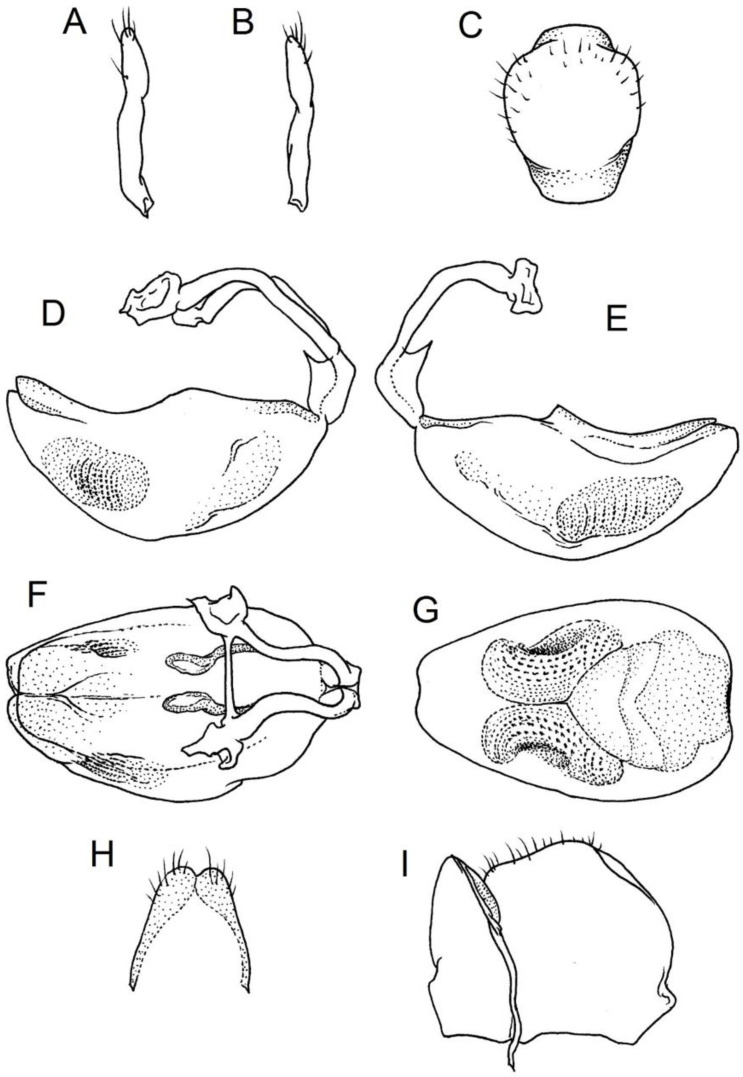
*Phonoctonus lutescens* (Guérin-Méneville & Percheron, 1834): (**A**) left paramere; (**B**) right paramere; (**C**) pygophore, ventral view; (**D**) aedeagus, right lateral view; (**E**) aedeagus, left lateral view; (**F**) aedeagus, dorsal view; (**G**) aedeagus, ventral view; (**H**) styloids, outer view; and (**I**) gonocoxite of abdominal segment VIII.

**Genitalia**: Male—Pygophore ovoid, slightly elongated in dorsal view (Figure 49C). Parameres relatively robust (narrow in the middle portion) with scarce, various sized setae on the apex (Figure 49A,B). Pedicel short and distinctly curved (Figure 49D,E). Endosomal struts of the aedeagus are relatively long with enlarged, spoon-like apices (Figure 49F). Basal plate elongated, with robust margins, with a relatively short basal plate bridge (Figure 49F). Dorsal phallothecal sclerite is tongue-like, with deeply divided apex (Figure 49F). Endosomal lobes with areas are covered by medium-sized, robust spines, deeply depressed fronto-dorsally (depressions elongated) and connected on the ventral side (Figure 49G).

Female—Styloids, relatively large and robust with distinctly club-shaped apical portions, covered by dense, medium-sized setae (in apical portion) (Figure 49H). Gonocoxite 8 is quadrangular with various sized (mostly short) and scarce setae; long setae on internal margin (Figure 49I). Gonapophyse 9 is relatively small and triangular.

**Measurements**: Body length: 24.4–24.6 (20.1–23.4); maximum width of abdomen: 6.1–6.9 (5.4–5.9); head length: 2.7–3.1 (2.6–2.7); head width: 1.5–1.6 (1.4–1.6); length of anteocular portion: 0.7–0.8 (0.7); length of postocular portion: 1.2–1.4 (1.2–1.3); length of synthlipsis: 1.1–1.2 (1.0–1.1); interocellar distance: 1.0 (0.9–1.0); length of antennal segments I:II:III:IV: 5.4–5.7 (4.6–5.3): 1.9–2.5 (1.9–2.1): 2.1–2.5 (2.0–2.6): 7.2–8.3 (5.4–5.8); length of labial segments I:II:III: 1.3 (1.1–1.2): 1.6–1.7 (1.5–1.7): 0.5–0.6 (0.5); maximum length of anterior pronotal lobe: 1.1–1.4 (1.1–1.3); maximum length of posterior pronotal lobe: 3.3–3.7 (3.2–3.6); maximum width of anterior pronotal lobe: 3.1–3.6 (3.0–3.3); maximum width of posterior pronotal lobe: 6.4–7.1 (6.1–6.8); length of scutellum: 2.1 (1.9–2.2); length of hemelytra: 17.6–18.2 (14.6–16.2).

**Figure 50:**
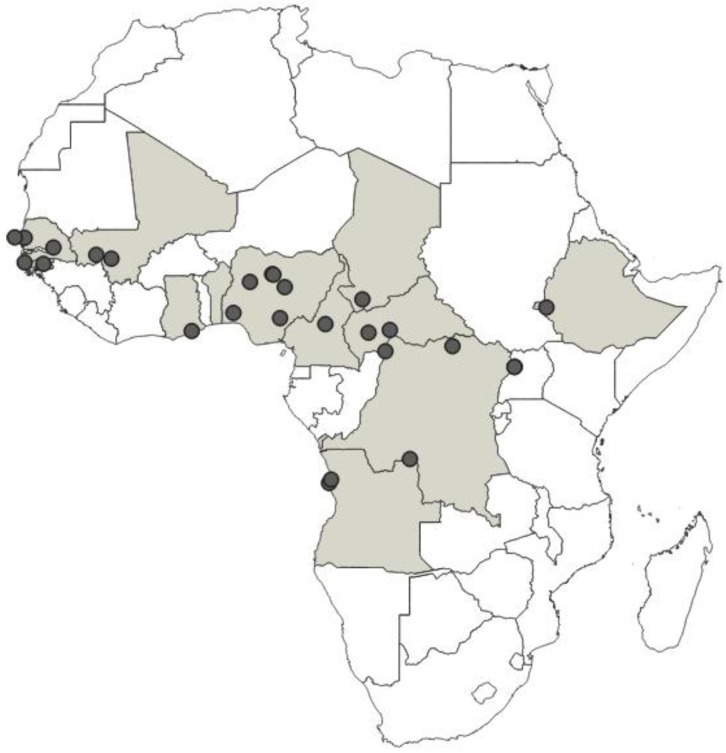
Known distribution of *Phonoctonus lutescens* (Guérin-Méneville & Percheron, 1834).

**Distribution**: Republic of Angola, Republic of Benin, Republic of Cameroon, Central African Republic, Republic of Chad, Democratic Republic of the Congo, Federal Democratic Republic of Ethiopia, Republic of Ghana, Republic of Guinea-Bissau, Republic of Mali, Federal Republic of Nigeria, Republic of Senegal (Figure 50).

**Comments**: In 1874 Stål transferred the species *Rhynocoris lutescens* (Guérin-Méneville & Percheron, 1834) to the genus *Phonoctonus*, and according to the original paper, the type specimens were not designated by the author. However, during our studies, we found the male specimen from Guinea-Bissau (formerly Portuguese Guinea), and to clarify uncertainties in the identification of this species and to fix its identity, a designation of a neotype is necessary, and we did so in the present paper under Articles 75.3.1 and 75.3.4 of the International Code of Zoological Nomenclature (4th edition, 1999).

10. ***Phonoctonus nigrofasciatus* Stål, 1855** (Figure 4D, Figure 5D, Figure 6J, Figure 8J, Figure 15B, Figure 16L, Figure 24C, Figure 25C, Figure 26M,N, Figure 30C,D, Figure 46B and Figure 51) **stat. rev.**

*Phonoctonus nigrofasciatus* Stål, 1855: 12:43. Holotype (♀): Caffraria; NHRS. Synonymised by Gerstaecker, 1873, 3:417.

*Harpactor nigrofasciatus* Walker, 1873: 8:108.

*Phonoctonus Poultoni* Schouteden, 1915: 4:258. Holotype (♀): Congo: Beni à Lesse; RMCA.

*Phonoctonus fasciatus* var. *poultoni* Villiers, 1953: 79:47. **syn. nov.**

**Type material examined**: Lectotype (present designation): • [♀] Caffra-/ria.; J. Wahlb.; nigro- fasciatus Stål; Typus; NHRS-GULI/000000557 (NHRS) (Figure 46B).

[for *P. poultoni*:] • [♀] HOLOTYPUS; Museé du Congo/Beni à Lesse/fin VII 1911/Dr. Murtula; R. Dét./A/2581 (RMCA). • [♀] PARATYPUS; Museé du Congo/Beni à Lesse/fin VII 1911/Dr. Murtula; R. Dét./A/2581 (RMCA). • [2x] PARATYPUS; Museé/du Congo Belge/Beni/Lt. Borgerhoff; R. Dét./A/2581 (RMCA) (Figure 46C).

Additional material examined—see Supplementary Materials S2.

**Diagnosis**: This species can be easily recognised by the following combination of characteristics: black head; antennae, legs, anterior pronotal lobe (except collar) and scutellum red; transversal stripe in the middle of the corium connected with the anterior margin of hemelytra and fused with black transversal and oblique stripe connected with the membrane (Figure 30C,D).

Redescription

**Colour**: Body generally pale—greyish to yellowish with reddish, black, and reddish patterns (Figure 30C,D).

**Head**: Black with pale (reddish or yellowish) neck (some specimens with reddish posterior portion of anteocular area and basal portion of clypeus; paler areas on ventral portion of head as well as maxillary and mandibular plates). Antennae black with a delicately paler base of distiflagellomerus. Labial segments are black (some specimens with the ventral side of the first visible labial segment).

**Thorax**: Collar of anterior pronotal lobe yellowish (Figure 6J and Figure 8J). Anterior pronotal lobe is brown, grey, or reddish (visibly darker than posterior pronotal lobe) (Figure 16L). Transversal sutura of pronotum in the colour of the pronotum. Posterior pronotal lobe is greyish or orange (same colour as corium) with various sized (mostly relatively thick), brown or black transversal stripe near the posterior margin. Transversal stripe is thinner in specimens with pale elements of the head. Pro-, meso, and metapleura in colour of the body with red or brown patterns in the middle. Proepimeron is partially black. Ventral portion of the thorax is yellow with red middle portions of meso- and metasternum. Scutellum yellow or reddish (some specimens with dark basal angles and half of lateral margins). Corium is greyish or orange with dark apical portion. Black, thick, transversal stripe with irregular margins, placed in the middle portion of the corium (anterior margin on the height of clavus apex) and fused with black transversal and oblique stripe connected with the membrane. Transversal stripe is connected with the anterior margin of hemelytra. Black transversal stripe visible on an apical portion of corium (on the height of the middle portion of basal cell) almost reached the corium apex (Figure 30C,D). Membrane is brown. Coxa is red. Trochanter, femur, tibia, and tarsus are dark brown to black.

**Abdomen**: Ventral portion of abdomen is red with yellow anterior and posterior margin of each segment. Yellow areas on ventral side of the abdomen not wider in lateral portions and with irregular margins. Connexives are red with yellow anterior and posterior margin (Figure 26M,N).

**Structure**: Body medium-sized (17.5–23.8 mm), dull with shiny central portion of posterior pronotal lobe and wing venation.

**Head**: Second visible labial segment is about 1.4 times longer than the first. Visible labial segments with medium-sized, erected setae. Scapus and pedicellus covered by scarce, short, erected setae. Basiflagellomerus and distiflagellomerus were covered by very dense, relatively short, lying setae. Ocelli medium-sized, placed dorso-laterally on distinctly visible tubercles.

**Thorax**: Anterior pronotal lobe with hollowed basal half of longitudinal sutura. Lateral and anterior margins of the anterior pronotal lobe and collar covered by medium-sized, dense, and erected setae. Anterior portion of anterior pronotal lobe covered by scarce, black setae. Calli medium-sized, anteriorly flattened with ovoid, deep depressions and small, rounded apices (Figure 16L). Posterior pronotal lobe is flattened in the middle with lateral margins covered by various sized, erected setae. Posterior margin of the posterior pronotal lobe is almost straight. Humeral angles with rounded apices. Scutellum with a central hollow. Trochanter, femur, and tibia with different sized setae (mostly medium-sized). Hemelytra surpassing the abdomen apex. Basal cell is visibly smaller than the discal cell.

**Abdomen**: Ventrally with various sized setae, lateral portion of each segment wrinkled.

**Figure 51:**
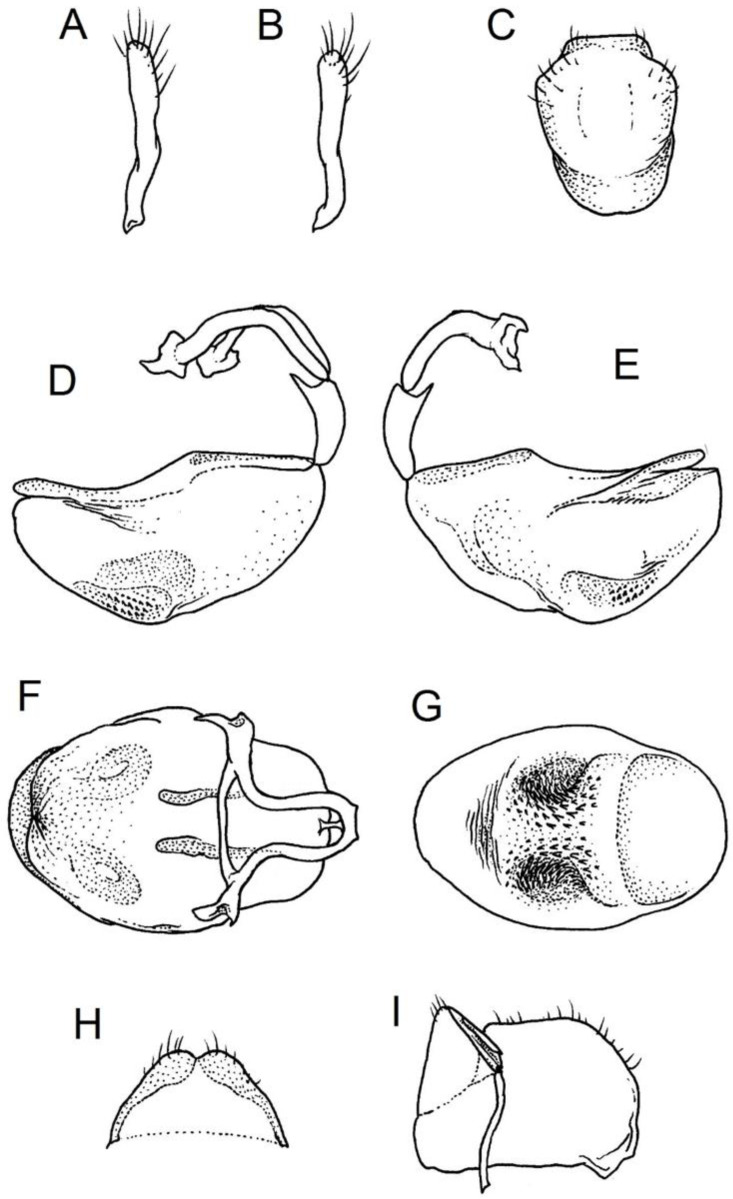
*Phonoctonus nigrofasciatus* Stål, 1855: (**A**) left paramere; (**B**) right paramere; (**C**) pygophore, ventral view; (**D**) aedeagus, right lateral view; (**E**) aedeagus, left lateral view; (**F**) aedeagus, dorsal view; (**G**) aedeagus, ventral view; (**H**) styloids, outer view; and (**I**) gonocoxite of abdominal segment VIII.

**Genitalia**: Male—Pygophore ovoid, rather short in dorsal view (Figure 51C). Parameres are relatively robust and short, slightly curved, narrow in the middle with relatively long setae on apex (Figure 51A,B). Pedicellus is medium-sized and delicately curved (Figure 51D,E). Endosomal struts of the aedeagus are short with enlarged, thong like apices, with irregular external margin (Figure 51F). Basal plate is elongated, v-shaped, with robust margins, with a relatively long basal plate bridge (Figure 51F). Dorsal phallothecal sclerite is tongue-like, with delicately divided apex (Figure 51F). Endosomal lobes have areas covered by medium-sized, robust spines, deeply depressed. Depressions elongated and places near the dorsal margin and the middle portion of areas (Figure 51G).

Female—Styloids, relatively large and robust with distinctly club-shaped apical portions, covered by very short and a few relatively long setae (Figure 51H). Gonocoxites of the eighth segment rectangular have elongated basal portion and with various size (mostly medium-sized) and scarce setae (Figure 51I). Gonapophyse 9 is relatively small (similar to styloids in size) and triangular.

**Measurements**: Body length: 18.6–23.8 (17.5–18.7); maximum width of abdomen: 5.2–6.6 (4.7–5.1); head length: 2.6–2.9 (2.5–2.7); head width: 1.3–1.6 (1.2–1.3); length of anteocular portion: 0.6–0.8 (0.6–0.7); length of postocular portion: 1.1–1.4 (1.0–1.2); length of synthlipsis: 1.0–1.1 (0.9–1.0); interocellar distance: 0.6–0.8 (0.7–0.8); length of antennal segments I:II:III:IV: 5.5–7.3 (4.7–5.2): 1.8–2.3 (1.6–2.0): 1.75–2.4 (1.8–2.0): 7.1–11.5 (7.0–7.6); length of labial segments I:II:III: 1.0–1.4 (1.0–1.1): 1.4–1.6 (1.4–1.5): 0.5–0.6 (0.5); maximum length of anterior pronotal lobe: 1.0–1.4 (0.9–1.0); maximum length of posterior pronotal lobe: 2.8–3.6 (2.6–2.8); maximum width of anterior pronotal lobe: 3.1–3.5 (2.7–2.8); maximum width of posterior pronotal lobe: 5.5–6.4 (4.9–5.4); length of scutellum: 1.4–1.9 (1.5–1.7); length of hemelytra: 12.9–17.3 (12.5–13.1).

**Figure 52:**
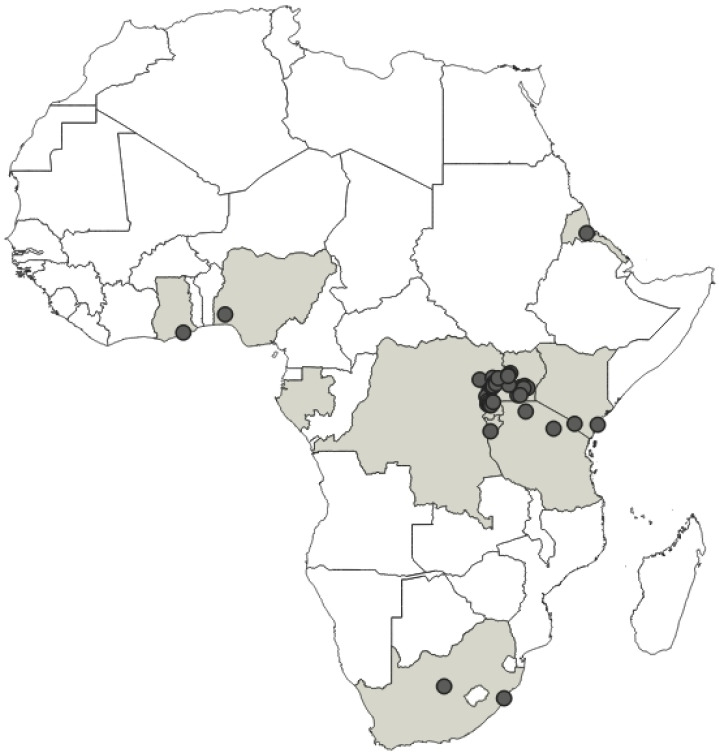
Known distribution of *Phonoctonus nigrofasciatus* Stål, 1855.

**Distribution**: Republic of Burundi, Democratic Republic of the Congo, State of Eritrea, Gabonese Republic, Republic of Ghana, Republic of Kenya, Federal Republic of Nigeria, Republic of Rwanda, Republic of South Africa, United Republic of Tanzania, Republic of Uganda (Figure 52).

**Comments**: *Phonoctonus nigrofasciatus* was described by Stål in 1855. In 1873, Walker [53] synonymised this species with genus *Harpactor*, and almost simultaneously, Gerstaecker [54] synonymised *P. nigrofasciatus* with *P. fasciatus*. The lack of communication between the authors meant that Schouteden (1915) described another species—*P. poultoni*, which has been synonymised by Villiers (1953) with *P. fasciatus* and treated by the author by its colour form (*P. fasciatus* var. *poultoni*). In summary, both described species were finally treated as synonyms or the colour forms of *P. fasciatus*. However, after the examination of type specimens of *P. nigrofasciatus* and *P. poultoni* and their male copulatory apparatus (which were not illustrated in any of the original papers), we concluded that these two taxa are not different at species level. However, they are not conspecific with *P. fasciatus*. As a consequence, *P. nigrofasciatus* was elevated to the rank of a species.

11. ***Phonoctonus picta* Schouteden, 1932** (Figure 6K, Figure 8K, Figure 16M, Figure 24G, Figure 25G, Figure 26O, Figure 30E, Figure 46D and Figure 53) **stat. nov.**

*Phonoctonus fasciatus* var. *picta* Schouteden, 1932: 1:202. Holotype (♀): Democratic Republic of the Congo: Baudoinville; RMCA.

**Type material examined**: • [♀] HOLOTYPUS/Museé du Congo/Baudoinville/fin XI-1918/R. Mayné; R. Dét./E/2581 (RMCA). • PARATYPUS/Museé du Congo/Baudoinville/fin XI-1918/R. Mayné; R. Dét./E/2581 (RMCA) (Figure 46D).

Additional material examined—see Supplementary Materials S2.

**Diagnosis**: This species can be recognised by the following combination of characters: head and anterior pronotal lobe (except collar) red; antennae, legs and scutellum black; transversal stripe in the middle of corium connected with anterior margin of hemelytra and fused with a thin, black, and oblique stripe connected with the membrane (Figure 30E). There are two superficially similar species: *P. nigrofasciatus* with black head and red scutellum (Figure 30C,D) and *P. picturatus* with a dark basal half of the corium (in some specimens) and transversal stripe of the corium connected only with the anterior margin of hemelytra (Figure 30F,G).

Redescription

**Colour**: Body generally pale—orange to yellowish with darker patterns (Figure 30E).

**Head**: Red or yellow with red neck. Posterior margin of interocular portion of the head, postocular portion of the head (some specimens with pale middle portion of postocular portion), and clypeus are darker. Antennal segments are dark brown. Labial segments are dark brown with reddish apical area and ventral side of the first visible labial segment.

**Thorax**: Collar of the anterior pronotal lobe is whitish or yellowish (Figure 6K and Figure 8K). Anterior pronotal lobe is light brown or red (visibly darker than posterior pronotal lobe) (Figure 16M). Transversal sutura of pronotum in the colour of the anterior pronotal lobe or darker. Posterior pronotal lobe is orange or yellowish (same colour as corium) with various sized (mostly relatively thick) and dark brown, wide transversal stripes near the posterior margin. Propleuron is red with yellowish proepisternum and brown proepimeron. Mesopleuron is red with yellow posterior margin, mesoepisternum, and mesoepimeron. Metapleura is red with yellow anterior margin and supracoxal portion. Ventral portion of thorax is red. Scutellum is black with a pale apex. Corium greyish or orange with black patterns. Black, wide, transversal stripe with irregular margins, placed in the middle portion of corium (anterior margin on the height of clavus apex) and fused with black transversal and oblique stripe connected with membrane. Transversal stripe not present on costal vein. Black transversal stripes visible on the apical portion of corium (on the height of the middle portion of basal cell), not reaching the corium apex (Figure 30E). Apical portion of the corium is pale. Membrane is brown. Coxa is red or dark brown. Trochanter, femur, tibia, and tarsus are dark brown (in some specimen basal portion of femur is red).

**Abdomen**: Ventral portion of the abdomen is red with a yellow anterior and posterior margin of each segment. Yellow areas on the ventral side of the abdomen are wider in lateral portions and with irregular margins. Connexives are red with yellow anterior and posterior margin (Figure 26O).

**Structure**: Body medium-sized (18.7–23.6 mm), dull with a shiny central portion of the posterior pronotal lobe and wing venation.

**Head**: Visible labial segments with various sized, erected setae. Scapus and pedicellus covered by scarce, short, erected setae. Basiflagellomerus and distiflagellomerus covered by very dense, rather short, lying setae. Ocelli medium-sized, placed dorso-laterally on distinctly visible tubercles.

**Thorax**: Anterior pronotal lobe with deeply hollowed basal half of longitudinal sutura. Lateral and anterior margins of the anterior pronotal lobe as well as collar covered by short, dense, and erected setae. Anterior portion of the anterior pronotal lobe covered by scarce, short, black setae. Calli medium-sized, anteriorly flattened, and deeply depressed with small, rounded apices (Figure 16M). Posterior pronotal lobe is flattened in the middle with lateral margins covered by various sized (rather short), erected setae. Posterior margin of the posterior pronotal lobe curved with an inward curve in the middle. Humeral angles with rounded apices. Ventral side of the thorax with different sized setae. Scutellum has a central hollow. Trochanter, femur, and tibia with different sized setae (mostly medium-sized). Hemelytra distinctly surpassing the abdomen apex. Basal cell is visibly smaller than the discal cell.

**Abdomen**: Ventrally with various sized setae, lateral portion of each segment wrinkled.

**Figure 53:**
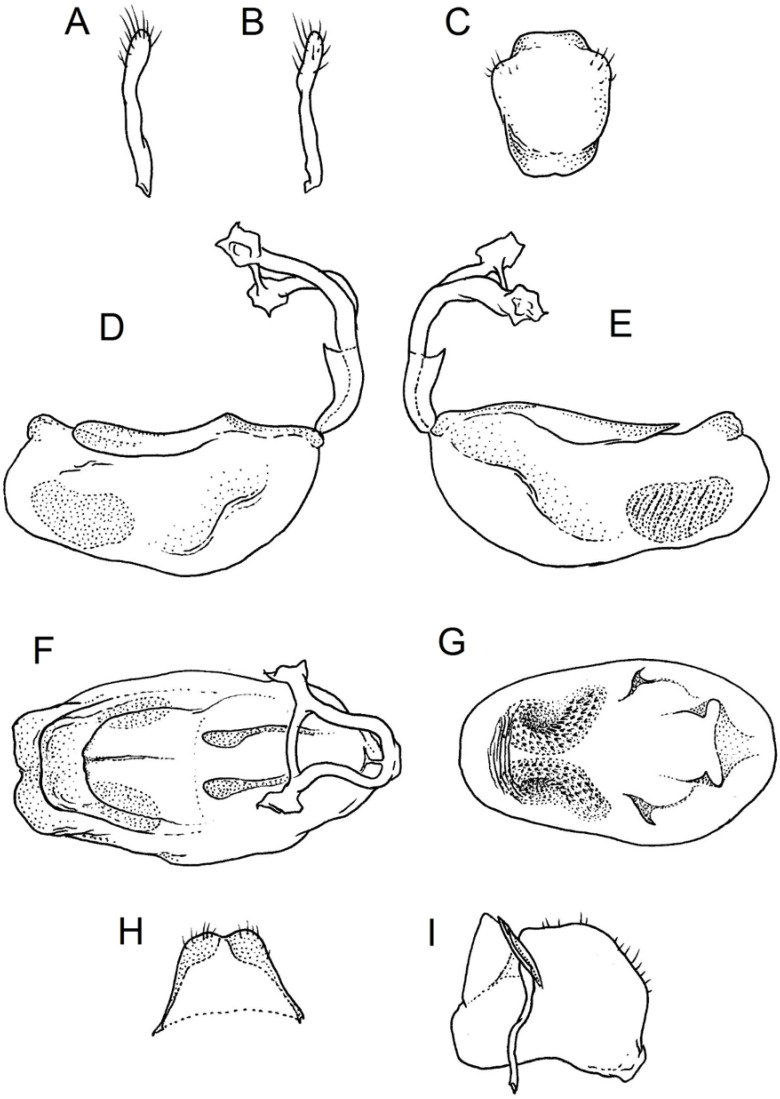
*Phonoctonus picta* Schouteden, 1932: (**A**) left paramere; (**B**) right paramere; (**C**) pygophore, ventral view; (**D**) aedeagus, right lateral view; (**E**) aedeagus, left lateral view; (**F**) aedeagus, dorsal view; (**G**) aedeagus, ventral view; (**H**) styloids, outer view; and (**I**) gonocoxite of abdominal segment VIII.

**Genitalia**: Male—Pygophore slightly elongated in dorsal view (Figure 53C). Parameres relatively robust, with club-shaped apical portion; slightly curved with medium-sized setae on the apex (Figure 53A,B). Pedicellus relatively long and wide (Figure 53D,E). Endosomal struts of the aedeagus are relatively long with enlarged, kidney like apices (Figure 53F). Basal plate elongated, with robust and straight margins, with a relatively short basal plate bridge (Figure 53F). Dorsal phallothecal sclerite is tongue-like, with divided apex (Figure 53F). Endosomal lobes with areas covered by short, robust spines, very deeply depressed on almost all surface (Figure 53G).

Female—Styloids, relatively large with robust and distinctly club-shaped apical portions, covered by dense, short, and a few long setae (in apical portion) (Figure 53H). Gonocoxite 8 is a rectangular shape with various sized (primarily long) and scarce setae (Figure 53I). Gonapophyse 9 is relatively large.

**Measurements**: Body length: 19.7–23.6 (18.7–21.1); maximum width of abdomen: 6.0–6.4 (4.8–5.9); head length: 2.7–3.0 (2.2–2.8); head width: 1.5–1.6 (1.3–1.5); length of anteocular portion: 0.5–0.7 (0.5–0.7); length of postocular portion: 1.1–1.4 (1.0–1.2); length of synthlipsis: 1.0–1.1 (0.9–1.1); interocellar distance: 0.7–0.8 (0.7–0.8); length of antennal segments I:II:III:IV: 5.1–6.6 (5.2–6.0): 1.8–2.3 (1.6–2.1): 1.5–2.4 (1.5–2.0): 6.7–9.7 (7.8–9.7); length of labial segments I:II:III: 1.1–1.2 (1.0–1.1): 1.5–1.6 (1.4–1.5): 0.5–0.6 (0.4–0.5); maximum length of anterior pronotal lobe: 1.0–1.3 (1.0–1.2); maximum length of posterior pronotal lobe: 2.8–3.7 (2.6–3.2); maximum width of anterior pronotal lobe: 3.0–3.6 (2.9–3.1); maximum width of posterior pronotal lobe: 5.5–6.7 (4.6–5.8); length of scutellum: 1.7–2.0 (1.3–2.0); length of hemelytra: 14.5–17.8 (13.3–15.5).

**Figure 54:**
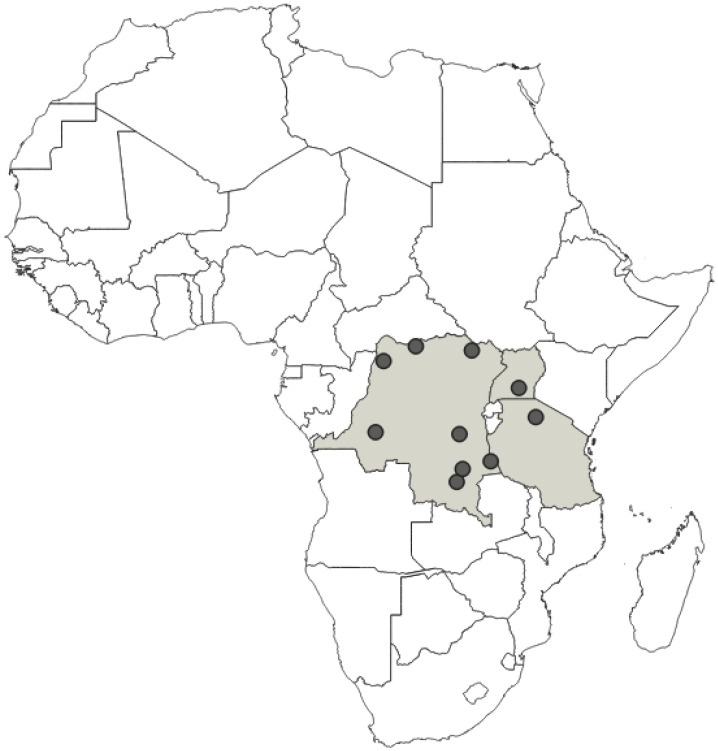
Known distribution of *Phonoctonus picta* Schouteden, 1932.

**Distribution**: Democratic Republic of the Congo, United Republic of Tanzania, Republic of Uganda (Figure 54).

**Comments**: The colour form of *P. fasciatus* var. *picta* was described by Schouteden [29]. After examining type specimens of *P. fasciatus* var. *picta* and specimens of *P. fasciatus* and their male copulatory apparatus, we concluded that these two taxa are different at species level. However, they are not conspecific with *P. fasciatus*. As a consequence, *P. picta* was elevated to the rank of a species.

12. ***Phonoctonus picturatus* Fairmaire, 1858** (Figure 6l, Figure 8L, Figure 16N, Figure 24F, Figure 25F, Figure 26P,Q, Figure 30F,G, Figure 46E,F and Figure 55) **stat. rev.**

*Phonoctonus picturatus* Fairmaire, 1858: 2:318. Holotype (♀): Gabon; MNHN.

*Harpactor picturatus* Walker, 1873: 8:109.

*Phonoctonus fasciatus var. picturatus* Villiers, 1948: 9:124. Gabon, Democratic Republic of Congo.

*Phonoctonus fasciatus* var. *discalis* Schouteden, 1932: 1:202. Holotype (♂): Belgian Congo: Mayumbe; MRAC. **syn. nov.**

**Type material examined**: • 2401/83; Nimocoris/picturatus/n.sp./Phonoctonus/picturatus/E03.; HOLOTYPE (MNHN) (Figure 46E).

[for *P. fasciatus* var. *discalis*:] • HOLOTYPUS; Museé du Congo/Mayumbe: Buku/Jembe -10-X-1924/A. Collart; R. Dét./F’/2581; Phonoctonus/fasciatus var./discalis Scht. (MRAC) (Figure 31C). • [2x] PARATYPUS; Museé du Congo/Congo da Lemba/V -1912/R. Mayné; R. Dét./F’/2581 (MRAC). • PARATYPUS; Museé du Congo/Mayumbe: Tshela/19-27-II-1916/R. Mayné; R. Dét./F’/2581 (MRAC). • [2x] PARATYPUS; Museé du Congo/Lualj/29-VIII-1913/Dr. Bequaert; R. Mayné; R. Dét./F’/2581 (MRAC). • [4x] PARATYPUS; Museé du Congo/Mayumbe 24-XI-15/Makaia N’Tete/R. Mayné; R. Dét./F’/2581 (MRAC). • [2x] PARATYPUS; Museé du Congo/Kiniati–Zobe/fin XII-1915/R. Mayné; R. Dét./F’/2581 (MRAC) (Figure 46F). • PARATYPUS; Museé du Congo/Mayumbe/Tsehobo/de Briey; R. Dét./F’/2581 (MRAC). • PARATYPUS; Museé du Congo/Mayumbe: Zobe/4an 12-I-1916/R. Mayné; R. Dét./F’/2581 (MRAC).

Additional material examined—see Supplementary Materials S2.

**Diagnosis**: This species can be recognised by the following combination of characteristics: head and anterior pronotal lobe (except collar) red; antennae, legs and scutellum black; transversal stripe in the middle of corium connected only with anterior margin of hemelytra (Figure 30F,G). There are two superficially similar species: *P. nigrofasciatus* with black head and red scutellum (Figure 30C,D), and *P. picta* with pale basal half of corium and thicker transversal stripe in the middle of the corium connected with the anterior margin of hemelytra and a black vertical stripe connected with a transversal stripe and membrane (Figure 30E).

Redescription

**Colour**: Body generally pale—greyish with reddish, orange, brown, and black patterns (Figure 30F,G).

**Head**: Reddish or light brown, paler antennifers and anterior portion of postocular portion. Posterior portion of postocular portion with dark patterns—mostly triangular (in some specimens, triangular spot divided into two smaller triangles along the longitudinal axis of the head). First visible labial segment is red or light brown (depend on the colour of the head) with a black ventral surface. Second and third visible labial segments are dark brown to black. Labrum is black.

**Thorax**: Collar of the anterior pronotal lobe is yellowish (Figure 6L and Figure 8L). Anterior pronotal lobe is reddish or brown with darker transversal sutura and pale anterior and lateral margins (Figure 16N). Posterior pronotal lobe is dark brown with pale margins. Prosternum is yellowish with a black pattern in the middle. Proepisternum is whitish, proepimeron is brown. Meso- and metapleura are brown. Mesopleuron, meso- and metepisternum whitish or yellow. Scutellum brown or dark brown with pale apical and lateral portions. Corium greyish or orange with pale apical portion. Apical portion of clavus darker (dark portion irregular and various). Relatively thin, transversal stripe with irregular margins, placed in the middle portion of the corium (anterior margin on the height of clavus apex) is dark brown. Additionally, some specimens with a darkened apical portion of corium (Figure 30G). Second brown transversal stripe is visible on the apical portion of the corium (on the height of the middle portion of basal cell) (Figure 30F,G). Membrane is brown. Coxa and trochanter are brown, femur, tibia and tarsus are brown to black (if femur brown then tibia is distinctly darker or black).

**Abdomen**: Ventral portion of abdomen is whitish or yellow with dark patterns on the lateral portion of each segment. Some pale specimens have only a thin brown line near the posterior margin of each abdominal tergite. Connexives are whitish, orange, or reddish (Figure 26P,Q).

**Structure**: Body medium-sized (19.8–24.5 mm), dull.

**Head**: Visible labial segments with short, erected setae. Scapus and pedicellus covered by scarce, short, erected setae. Basiflagellomerus and distiflagellomerus covered by very dense, rather short, lying setae. Ocelli relatively large, placed widely on distinctly visible tubercles. 

**Thorax**: Anterior pronotal lobe with deeply hollowed basal portion of longitudinal sutura. Lateral and anterior margins of anterior pronotal lobe and collar covered by medium-sized, dense, and erected setae. Calli medium-sized, distinctly flattened in dorsal and frontal view with small depression and rounded apices (Figure 16L). Posterior pronotal lobe flattened in the middle with a curved posterior portion. Lateral margins are covered by various sized, erected setae. Posterior margin of posterior pronotal lobe is almost straight. Humeral angles with rounded apices. Scutellum with a central hollow. Trochanter, femur, and tibia have different sized setae (mostly medium-sized). Hemelytra surpasses the abdomen apex. Basal cell distinctly smaller than the discal cell.

**Abdomen**: Ventrally with various sized setae, lateral portion of each segment wrinkled.

**Figure 55:**
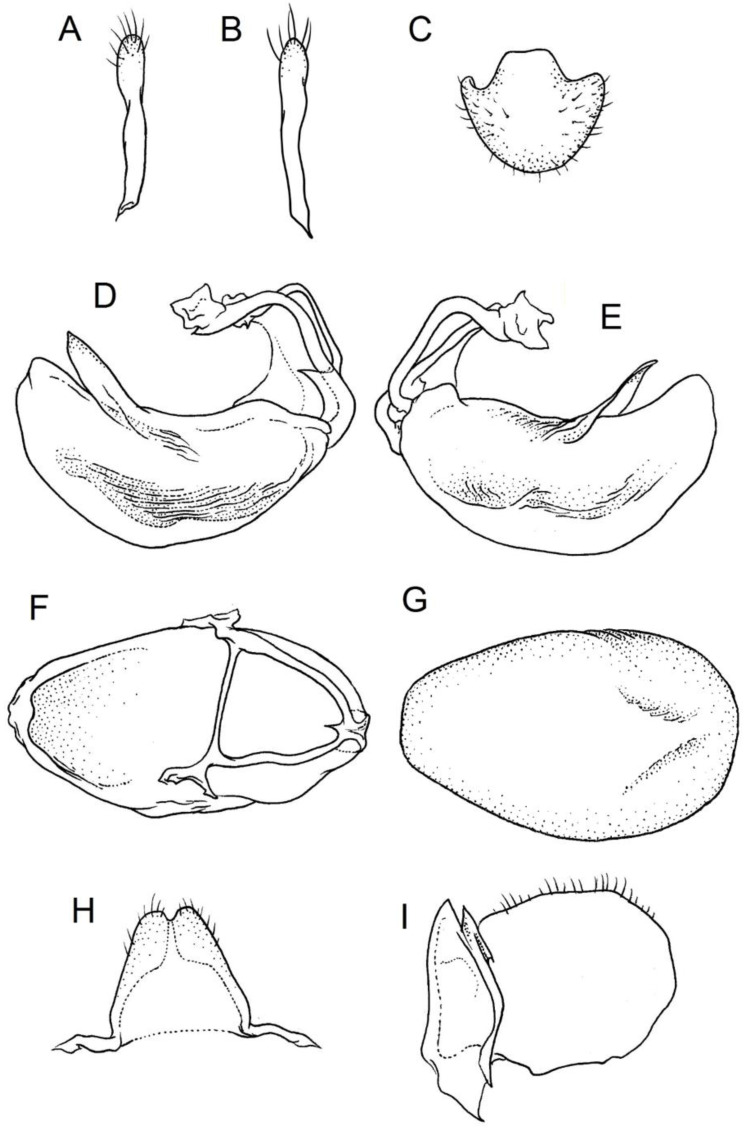
*Phonoctonus picturatus* Fairmaire, 1858: (**A**) left paramere; (**B**) right paramere; (**C**) pygophore, ventral view; (**D**) aedeagus, right lateral view; (**E**) aedeagus, left lateral view; (**F**) aedeagus, dorsal view; (**G**) aedeagus, ventral view; (**H**) styloids, outer view; and (**I**) gonocoxite of abdominal segment VIII.

**Genitalia**: Male—Pygophore ovoid in dorsal view (Figure 55C). Parameres are relatively robust, slightly curved with relatively long setae on apex (Figure 55A,B). Pedicel is short and wide (Figure 55D,E). Endosomal struts of aedeagus is long with enlarged, crescent in shape apices. Crescent-like portion with distinctly visible, small nodules on the inner margin (Figure 55F). Basal plate elongated, with robust margins, with a relatively long basal plate bridge (Figure 55F). Dorsal phallothecal sclerite is tongue-like with a divided apex (Figure 54F). Endosomal lobes have areas covered by short, robust spines, deeply depressed. Depressions are elongated and placed in the middle portion of areas (Figure 55G).

Female—Styloids, relatively large and robust with distinctly club-shaped apical portions, covered by dense, medium-sized setae on the external margin (in apical portion) (Figure 55H). Gonocoxite 8 is trapeze-like shape with elongated basal portion and with various sized (primarily long) and scarce setae (Figure 55I). Gonapophyse 9 is relatively small (similar to styloids in size) and triangular, without setae.

**Measurements**: Body length: 20.5–24.5 (19.8–23.8); maximum width of abdomen: 5.3–6.4 (4.5–6.7); head length: 2.9–3.2 (2.7–3.5); head width: 1.4–1.7 (1.4–1.6); length of anteocular portion: 0.6–0.7 (0.7–0.8); length of postocular portion: 1.1–1.5 (1.2–1.7); length of synthlipsis: 1.0–1.3 (1.0–1.2); interocellar distance: 0.7–0.8 (0.7–0.8); length of antennal segments I:II:III:IV: 7.1–8.5 (6.4–8.0): 2.1–2.6 (2.0–2.4): 2.2–2.8 (2.3–2.5): 11.3–12.2 (9.8–11.2); length of labial segments I:II:III: 1.1–1.5 (1.0–1.4): 1.6–1.9 (1.5–1.7): 0.5–0.6 (0.5); maximum length of anterior pronotal lobe: 1.0–1.6 (1.0–1.5); maximum length of posterior pronotal lobe: 2.9–3.4 (2.7–3.3); maximum width of anterior pronotal lobe: 3.1–3.6 (3.0–3.9); maximum width of posterior pronotal lobe: 5.7–6.4 (5.4–6.8); length of scutellum: 1.7–2.2 (1.5–1.9); length of hemelytra: 14.7–17.3 (14.3–17.1).

**Figure 56:**
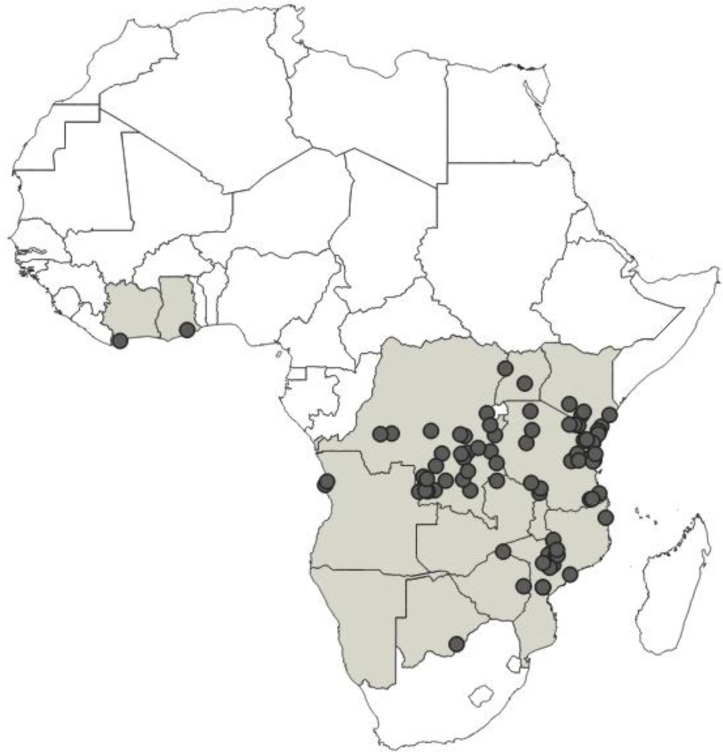
Known distribution of *Phonoctonus picturatus* Fairmaire, 1858.

**Distribution**: Republic of Angola, Democratic Republic of the Congo, Republic of the Congo, Gabonese Republic (Figure 56).

**Comments**: *Phonoctonuspicturatus* was described by Fairmaire [28]. In 1873, Walker [53] synonymised this species with the genus *Harpactor*. Villiers [11] synonymised this species with *P. fasciatus* and lowered its taxonomic rank to colour form (*P. fasciatus* var. *picturatus*). During the examination of the material of *P. fasciatus* and *P. picturatus* and their male copulatory apparatus, we concluded that those two taxa are not conspecific. While examining the type material of *P. picturatus* and another colour form of *P. fasciatus* var. *discalis* described by Schouteden (1932) and their male copulatory apparatus, we concluded that these two taxa are not different at the species level. Consequently, *P. picturatus* was elevated to the rank of a species and *P. fasciatus* var. *discalis* is its junior synonym.

13. ***Phonoctonus principalis* Gerstaecker, 1892** (Figure 5A,I, Figure 6M, Figure 8M, Figure 16O, Figure 24J, Figure 25J, Figure 26R, Figure 30H,I, Figure 46G and Figure 57).

*Phonoctonus principalis* Gerstaecker, 1892: 9:52 (10). Syntype (♂): Mozambique: Quilimane; ZMUH.

*Phonoctonus validus* Horváth, 1892: 15:263 [published in 1893]. Syntype (♀): Mozambique: Quilimane; HNHM. **syn. nov.**

**Type material examined**: Lectotype (present designation): • [♀] 169./Quilimane/9.II.89./Coll. Stuhlmann; A. Gerstäcker/determ. 1891.; Phonoctonus/principalis/Gerst. (ZMUH) (Figure 46G).

Lectotype (present designation) [for *P. validus*]: • [♀] Quilimane; Phonoctonus/principalis Gerst; Phonoctonus/validus Horv.; Phonocotnus/validus; Hung. Nat. Hist. Mus/Budapest/coll. Hemiptera (HNHM).

Additional material examined—see Supplementary Materials S2.

**Diagnosis**: This species can be easily recognised by the following combination of characters: head, anterior pronotal lobe (except collar), scutellum and legs are red; antennae black; large, transversal, subrectangular spots in the middle of corium or thin transversal stripe in the middle of corium connected with anterior margin of hemelytra (Figure 30H,I). Superficially resembles *P. grandis*, but is easy to distinguish through a transversally elongated stripe on posterior pronotal lobe and lighter legs. Moreover, *P. grandis* is not found in mainland Africa.

Redescription

**Colour**: Body generally pale—greyish, brown to orange and red with black patterns on thorax, hemelytra and abdomen (Figure 30H,I).

**Head**: Red. First visible labial segment and basal portion of second segment are red. Labrum, apical half of the second visible labial segment, and third segment are black (Figure 6M and Figure 8M). All antennal segments are black.

**Thorax**: Collar of anterior pronotal lobe is yellowish with red hollows on calli. Anterior pronotal lobe is red (Figure 16O). Posterior pronotal lobe is greyish to brown with a thin black line along the transversal sutura (not reaching lateral margins of the posterior pronotal lobe). Posterior margin of the posterior pronotal lobe with a black, arcuate stripe with irregular margins. Lateral and posterior margins of posterior pronotal lobe is yellowish. Propleuron with a dark brown anterior and red posterior portion. Proepisternum is whitish, proepimeron, brown to reddish. Prosternum is red. Meso- and metasternum are red. Mesopleuron is red with a dark brown anterior and posterior margins. Mesoepisternum and mesoepimeron are whitish with black patterns. Metapleuron is whitish with a brown medial portion. Metacoxal cavity is whitish. Scutellum is red. Corium is greyish with reddish or red apical portion with distinct, transversally elongated, black spot in the middle portion, or thin transversal stripe in the middle of the corium connected with anterior margin of the hemelytra. Black, transversal pattern on the discal cell base, connected with the costal vein (Figure 30H,I). Membrane is brown to black. Coxa and trochanter are red. Fore and middle femur are red with brown longitudinal patterns on the ventral surface. Hind femur is brown with red apical and basal portion. Tibia is brown with red apex. Tarsus is brown.

**Abdomen**: The ventral portion of the abdomen is red with the whitish or yellowish anterior and posterior portion of each segment (VII abdominal segment with whitish anterior margin only). Connexives are red with whitish anterior margins (Figure 26R).

**Structure**: Body large (23.2–26.5) and dull.

**Head**: Short setae on the anteocular portion and long on the postocular portion. Visible labial segments have various sized, erected setae. Scapus covered by scarce, short setae, except the club-shaped apical portion, covered by medium-sized, erected, and rather dense setae. Pedicellus is covered by dense, medium-sized, erected setae. Basiflagellomerus and distiflagellomerus are covered by very dense, rather short, lying setae. Ocelli medium-sized, placed dorso-laterally on distinct tubercles. Anterior pronotal lobe has hollowed basal half of longitudinal sutura. Collar, lateral, and anterior margins of the anterior pronotal lobe covered by rather long, dense and erected setae. Calli medium-sized, relatively short, rounded apices and small hollows on fore surface (Figure 16O). The posterior pronotal lobe flattened laterally. Lateral and anterior margins of the posterior pronotal lobe are covered by various sized, erected setae. Posterior margin of the posterior pronotal lobe curved inwardly. Humeral angles with rounded apices. Scutellum with delicate, central depression. Trochanter, femur, and tibia with various sized, rather short setae. Hemelytra robust and long, distinctly surpassing abdomen apex. Basal cell is smaller or similar in size to discal cell.

**Abdomen**: Ventrally with various sized setae, lateral portion of each segment wrinkled.

**Figure 57:**
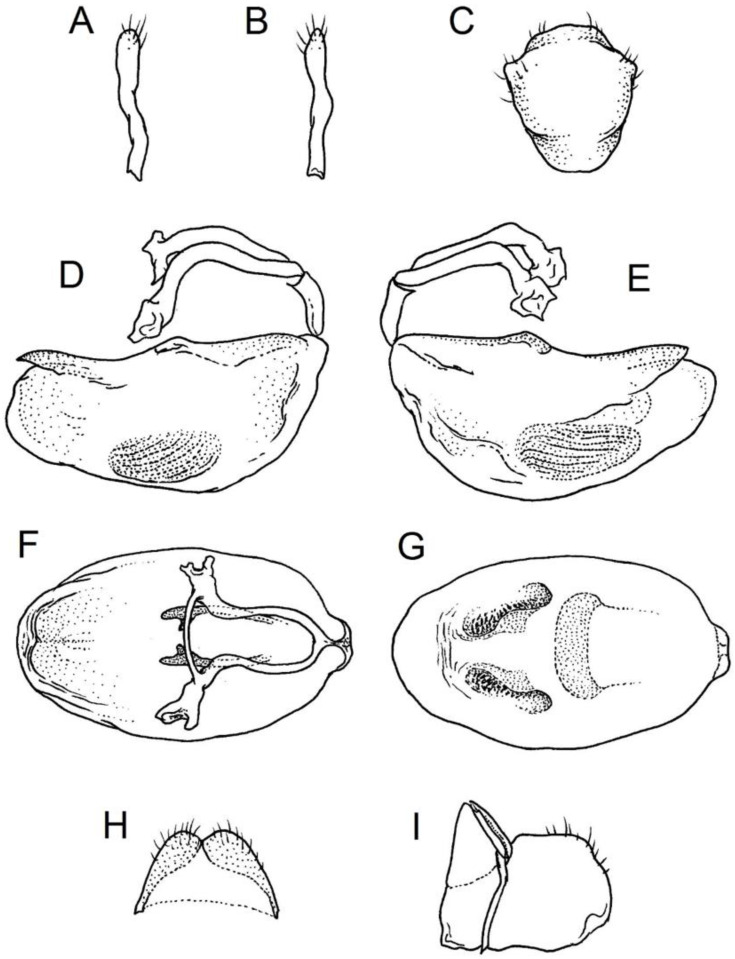
*Phonoctonus principalis* Gerstaecker, 1892: (**A**) left paramere; (**B**) right paramere; (**C**) pygophore, ventral view; (**D**) aedeagus, right lateral view; (**E**) aedeagus, left lateral view; (**F**) aedeagus, dorsal view; (**G**) aedeagus, ventral view; (**H**) styloids, outer view; (**I**) gonocoxite of abdominal segment VIII.

**Genitalia**: Male—Pygophore ovoid, rather short in dorsal view (Figure 57C). Parameres relatively long with a slightly enlarged apical portion with relatively long setae on apex (Figure 57A,B). Pedicellus short and delicately curved (Figure 57D,E). Endosomal struts of aedeagus are long with enlarged, crescent in shape apices (Figure 57F). Basal plate is wide, with thin margins and long basal plate bridge (Figure 57F). Dorsal phallothecal sclerite is tongue-like, with divided apex (Figure 57F). Endosomal lobes have areas covered by short, robust spines, that are deeply depressed. Depressions are elongated and placed in the middle portion of areas (Figure 57G).

Female—Styloids, relatively large and robust with distinctly club-shaped apical portions, covered by dense, medium-sized setae on external margin (Figure 57H). Gonocoxite 8 is rectangular with elongated basal portion and various sized (primarily long) and scarce setae (Figure 57I). Gonapophyse 9 is triangular.

**Measurements**: Body length: 25.2–26.5 (23.2–25.1); maximum width of abdomen: 6.2–7.2 (5.5–7.4); head length: 3.4–3.5 (3.2–3.3); head width: 1.8–1.9 (1.6–1.7); length of anteocular portion: 0.9 (0.8); length of postocular portion: 1.7 (1.4–1.5); length of synthlipsis: 1.15–1.3 (1.1–1.2); interocellar distance: 0.9–1.0 (1.0); length of antennal segments I:II:III:IV: 6.0–6.3 (5.5–6.2):2.0–2.5 (1.8–2.5):2.5–3.0 (1.8–3.0):7.2–8.3 (5.9–9.0); length of labial segments I:II:III: 1.3–1.5 (1.3–1.5):1.6–2.0 (1.6–1.7):0.6–0.65 (0.6); maximum length of anterior pronotal lobe: 1.5–1.6 (1.5–1.6); maximum length of posterior pronotal lobe: 3.7–4.1 (3.6–4.2); maximum width of anterior pronotal lobe: 3.8–4.0 (3.5–3.8); maximum width of posterior pronotal lobe: 7.2–7.7 (6.6–7.8); length of scutellum: 1.6–2.1 (1.8–1.9); length of hemelytra: 17.8–19.2 (16.2–18.0).

**Figure 58:**
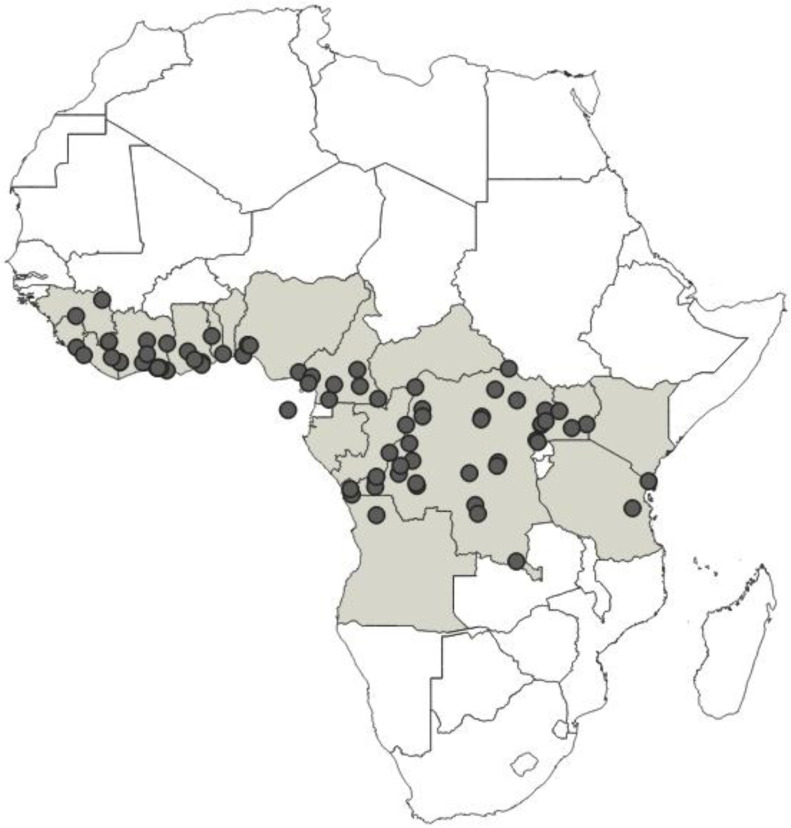
Known distribution of *Phonoctonus principalis* Gerstaecker, 1892.

**Distribution**: Republic of Angola, Republic of Botswana, Republic of Burundi, Democratic Republic of the Congo, Republic of The Gambia, Republic of Ghana, Republic of Côte d’Ivoire (Ivory Coast), Republic of Kenya, Republic of Malawi, Republic of Mozambique, Republic of Namibia, United Republic of Tanzania, Republic of Uganda, Republic of Zambia, Republic of Zimbabwe (Figure 58).

**Comments**: An examined type specimen of *P. principalis* deposited in ZMUH is considered a syntype (which is also consistent with the information contained in the original work of Gerstaecker, published in August 1892). We designated this specimen as a lectotype. After examining the type specimen of *P. validus* (described by G. Horváth in a paper published in March 1893), especially the copulatory apparatus, we concluded that those two taxa are conspecific. The name *P. principalis* was published first, then as a senior synonym, which under Article 23 of the International Code of Zoological Nomenclature (4th edition, 1999) is the valid species name.

Morphological phylogenetic analysis

**Figure 59:**
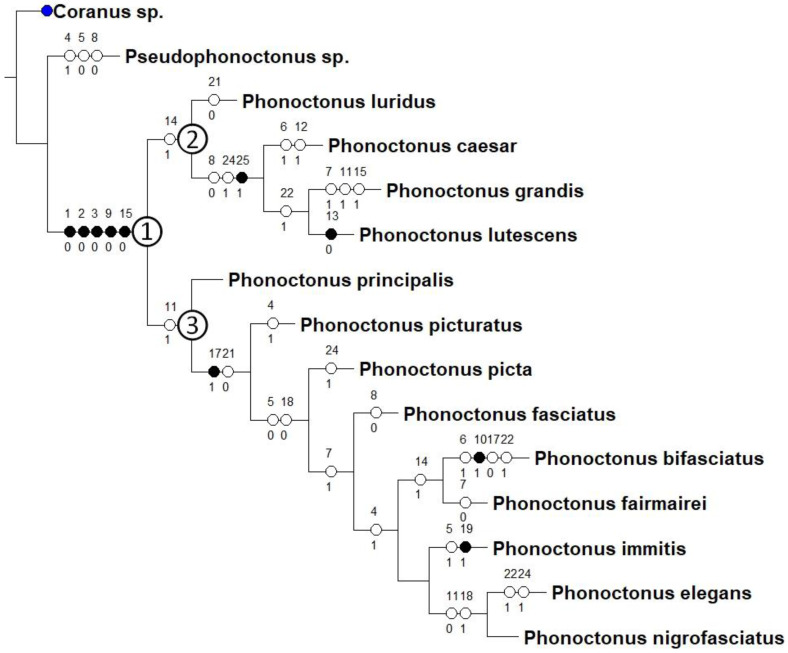
Phylogenetic hypothesis for *Phonoctonus* species. Most parsimonious tree based on morphological data. Node numbers placed in open circles. Synapomorphies are shown as black circles on branches. Contradicted apomorphies are shown as white circles. *Coranus* sp. as root of tree.

Morphological phylogenetic analysis recovered the genus *Phonoctonus* monophyletic (Figure 58). The unweighted analysis produced 307 trees in TNT. The minimum tree length found was 73 steps, with consistency index (CI) = 0.48 and retention index (RI) = 0.54. Nodes of major clades are numbered 1–9. Synapomorphies and contradicted apomorphies are indicated in Figure 59.

*Coranus* sp. roots the tree. *Pseudophonoctonus* sp. was inferred as the sister group of *Phonoctonus*. The distribution of character states by node found in this tree was as follows:

**Node 1.** Genus *Phonoctonus* is supported by five non-contradicted apomorphies: second (first visible) labial segment is shorter than the third (second visible) labial segment (1-0); presence of posterior pronotal extensions (2-0); antennal insertion pointed close to the anterior margin of an eye (3-0); triangular basal cell (9-0); enlarged lateral ridges of anterior pronotal lobe (15-0).

**Node 2.** *Phonoctonus luridus* (*P. caesar* (*P. grandis* + *P. lutescens*)). This clade is supported by one contradicted apomorphy: long apodeme depression of the pronotum (14-1). *Phonoctonus caesar* (*P. grandis* + *P. lutescens*) is supported by one non-contradicted apomorphy: endosomal spine areas are connected (25-1). It is also supported by two contradicted apomorphies: same/similar size of basal cell and distal cell (8-0); divided apex of dorsal phallothecal sclerite (24-1). *Phonoctonus lutescens* is also supported by one non-contradicted apomorphy: shallow apodeme depression of the pronotum (13-0).

**Node 3.** This clade is supported by one contradicted apomorphy: no enlarged anterolateral angles of the anterior collar of the pronotum (11-1). It includes most species belonging to the genus *Phonoctonus*. Apart from *P. principalis*, other species in this clade are supported by one non-contradicted apomorphy: presence of the bright belt on the 4th antennal segment (17-1).

## 4. Conclusions

Stål described the genus *Phonoctonus* based on a species previously described as *Reduvius fasciatus* by Palisot de Beauvois. He then described three new species—*Phonoctonus nigrofasciatus*, *P. immitis*, and *P. subimpictus*, the first of which were later synonymised to *P. fasciatus* as well as designated by Fairmaire *P. picturatus*. In 1860 (actually in 1861), Signoret [37] described *P. grandis* from Madagascar, and in 1874, Stål [55] transferred *Rhynocoris lutescens* to *Phonoctonus*. At the end of the nineteenth century and early twentieth century, another five species were classified to the genus *Phonoctonus—P. principalis*, *P. validus*, *P. caesar*, *P. elegans*, and *P. poultoni*. *Phonoctonus luridus* is the last known described species.

In addition, colour variations of different species were described—*P. immitis* var. *subimpictus* (synonymised by Villiers to *P. subimpictus*), *P. elegans* var. *stricta*, *P. fasciatus* var. *picta*, *P. fasciatus* var. *discalis*, *P. fasciatus* var. *bifasciatus*, *P. fasciatus* var. *picturatus*, *P. fasciatus* var. *fairmairei* as well as *P. fasciatus* var. *poultoni*.

The last known publication presenting numerous taxonomic problems within the genus *Phonoctonus* is the Maldonado Capriles catalogue [2], in which the author distinguished nine species. After examination of various morphological characters as well as the morphology of male and female genitalia of the type material and additional specimens of *P. bifasciatus*, *P. fairmairei*, *P. nigrofasciatus*, *P. picta*, and *P. picturatus*, which previous authors treated as the colour variations of *P. fasciatus*, we concluded that all mentioned taxa are not conspecific and were elevated to the rank of a species. Despite the visible differences (in the species level) between the mentioned species, their previous status was mainly the result of a lack of examination of the genital structures, which were not illustrated in any of the original papers.

After examining external morphology, colouration, and the morphology of male genital structures of the type specimens of *P. validus* and *P. principalis*, both species were recognised by us as conspecific. The authors prepared their descriptions almost simultaneously, which is probably the main reason for the current taxonomic confusion. In fact, Gerstaecker’s work was published in 1892 [31], while Horváth’s work appeared in March 1893 [32]. Therefore *P. validus* was placed as a junior synonym of *P. principalis*.

The previously mentioned morphological analyses identified *P. nigrofasciatus* and *P. fasciatus* var. *poultoni* as conspecific, respectively *P. picturatus* and *P. fasciatus* var. *discalis*. Therefore, appropriate synonymisations were proposed.

*Phonoctonus* was included in molecular phylogenetic analyses for the tribe Harpactorini before [56,57] and also found itself in the sister clade to the *Coranus* species. Moreover, it was most closely related to the representatives of the genus *Euagoras* (found in Asia and Australia). Here, *Phonoctonus* was recovered as monophyletic but only based on morphological data. Future analyses including molecular data might help resolve some relationships within this genus. For this purpose, however, it would be necessary to collect live insect specimens because our museum material did not allow for the isolation of satisfactory DNA sequences for all species.

## Data Availability

The data presented in this study are available in insert article or supplementary material here.

## Supplementary Materials

The following are available online at https://www.mdpi.com/article/10.3390/insects12121100/s1, Supplementary Materials S1: Morphological matrix; Supplementary Materials S2: Detailed list of examined materials.

## References

[1] Froeschner R.C., Kormilev N.A. (1989). Phymatidae or ambush bugs of the world: A synonymic list with keys to species, except Lo-phoscutus and Phymata (Hemiptera). Entomography.

[2] Capriles J.M. (1990). Systematic Catalogue of the Reduviidae of the World (Insecta: Heteroptera). Special Edition of the Caribbean Journal of Science.

[3] Cassis G., Gross G.F., Maynard G.V. (1995). Hemiptera: Heteroptera (Coleorrhyncha to Cimicomorpha). Houston WWK.

[4] Weirauch C., Berenger J.M., Berniker L., Forero D., Forthman M., Frankenberg S., Freedman A., Gordon E., Hoey-Chamberlain R., Hwang W.S. (2014). An illustrated identification key to as-sassin bug subfamilies and tribes. Can. J. Arthropod Identif..

[5] Readio P.A. (1927). Studies on the biology of the Reduviidae of America north of Mexico. Univ. Kans. Sci. Bull..

[6] Louis D. (1974). Biology of Reduviidae of Cocoa Farms in Ghana. Am. Midl. Nat..

[7] Putshkov V.G., Putshkov P.V. (1985). A Catalogue of Assassin-Bug Genera of the World (Heteroptera, Reduviidae).

[8] Putshkov V.G., Putshkov P.V. (1988). A Catalogue of Assassin-Bugs of the World (Heteroptera, Reduviidae). III. Harpactorinae.

[9] Hwang W.S., Weirauch C. (2012). Evolutionary History of Assassin Bugs (Insecta: Hemiptera: Reduviidae): Insights from Divergence Dating and Ancestral State Reconstruction. PLoS ONE.

[10] Stål C. (1853). Nya genera bland Hemiptera. Öfversigt Af Kongliga Vetensk.-Akad. Förhandlingar.

[11] Villiers A. (1948). Faune de L’Empire Français. IX. Hémiptères Rédiviidés de l’Afrique Noire. Paris: Éditions du Muséum.

[12] Evans D.E. (1962). The food requirements of phonoctonus nigrofasciatus stål. (hemiptera, reduviidae). Èntomol. Exp. Appl..

[13] Guérin-Méneville F.E., Duperrey L.-I. (1831). Crustacés, Arachnides et Insectes. Voyage Autour du Monde, Exécuté par Ordre du Roi, sur la Corvette de Sa Majesté “La Coquille”.

[14] Kirkaldy G.W. (1904). Bibliographical and Nomenclatorial Notes on the Hemiptera, No. 3. Entomologist.

[15] De Laporte F.L. (1833). Essai d’une classification systématique de l’ordre des Hémiptères (Hémiptères Hétéroptères, Latr.). Mag. Zool..

[16] Myers J.G. (1928). The biological control of cotton pests. Emp. Cotton Grow. Rev..

[17] Stride G.O. (1956). On the mimetic association between certain species of Phonoctonus (Hemiptera, Reduviidae) and the Pyrrhocori-dae. J. Entomol. Soc. South. Afr..

[18] Stride G.O. (1965). On the biology of certain West African species of Phonoctonus (Hemiptera, Reduviidae), mimetic predators of the Pyrrhocoridae. J. Entomol. Soc. S. Afr..

[19] Fuseini B.A., Kumar R. (1975). Biology and immature stages of cotton stainers (Heteroptera: Pyrrhocoridae) found in Ghana. Biol. J. Linn. Soc..

[20] Fuseini B.A., Kumar R. (1975). Ecology of cotton stainers (Heteroptera: Pyrrhocoridae) in southern Ghana. Biol. J. Linn. Soc..

[21] Fadare T.A. (1978). Efficiency of Phonoctonus spp. (Hemiptera; Reduviidae) as regulators of populations of Dysdercus spp. (Hemip-tera; Pyrrhocoridae). Niger. J. Entomol..

[22] Schaefer C.W., Ahmad I. (1987). Parasites and predators of pyrrhocoroidea (Hemiptera), and possible control of cotton stainers by Phonoctonus spp. (Hemiptera: Reduviidae). Entomophaga.

[23] Risbec J. (1955). Hymenopteres Parasites Du Cameroun. Bull. L’institut Français D’afrique Noire.

[24] Fusu L., Ebrahimi E., Siebold C., Villemant C. (2015). Revision of the Eupelmidae Walker, 1833 described by Jean Risbec. Part 1: The slide mounted specimens housed at the Muséum national d’Histoire naturelle in Paris. Zoosystema.

[25] De Beauvois P.A.M.F.J. (1805). La flore d’Oware et de Benin en Afrique.

[26] Parker A.H. (1972). The predatory and sexual behaviour of *Phonoctonus fasciatus* (P. de B.) and *P. subimpictus* Stål (Hem., Reduvi-idae). Bull. Entomol. Res..

[27] Stål C. (1855). Hemiptera fran Kafferlandet. Öfversigt Kongliga Vetensk.-Akad. Förhandlingar.

[28] Fairmaire L.M.H., Signoret A.V., Thomson M.J. (1858). Voyage au Gabon. V. Ordre. Hémiptères. Archives Entomologiques ou Recueil Contenant des Illustrations D’insectes Nouveaux ou Rares.

[29] Schouteden H. (1932). Catalogues raisonnés de la faune entomologique du Congo Belge. Tome II. Fascicule 2. Hémiptères-Réduviides. Ann. Du Musée Du Congo Belg. Zool. Série III Sect. II.

[30] Villiers A., Witte G.F. (1953). Reduviidae (Hemiptera, Heteroptera). Exploration du parc national Albert: Mission G. F. de Witte (1933–1935).

[31] Gerstaecker C.E.A. (1892). Bestimmung der von Herrn Dr. F. Stuhlmann in Ost-Arfika gesammelten Hemiptera. Jahrb. Ham-Burgischen Wiss. Anst..

[32] Horváth G. (1893). Hemiptera nova Africana. Természetrajzi Füzetek.

[33] Guérin-Méneville F.-É., Percheron A. (1835). Genera des insectes, ou, Exposition détaillée de tous les caractères propres à chacun des genres de cette classe d’animaux. Genera des Insectes, ou, Exposition Détaillée de Tous les Caracteères Propres à Chacun des Genres de cette Classe D’animaux.

[34] Haglund C.J.E. (1895). Beiträge zur Kenntniss der Insektenfauna von Kamerun. 4. Verzeichness der von Yngve Sjöstedt im nordwest-lichen Kamerungebiete eingesammelten Hemipteren. 2 Öfversigt Kongliga Vetensk.-Akad. Förhandlingar.

[35] Varela G.A. (1904). Notas Hemipterológicas. Reduvidos nuevos. Boletín Soc. Española Hist. Nat..

[36] Miller N. (1951). XLVI.—New Reduviidae in the collection of the British Museum (Natural History). J. Nat. Hist..

[37] Signoret A.V. (1861). Fauna des Hémiptères de Madagascar, 2 partie. (Suite et fin). Ann. Société Entomol. Fr..

[38] Schouteden H. (1915). Cas de mimétisme ches les Hémiptères africains. Rev. Zool. Afr..

[39] Weirauch C. (2008). Cladistic analysis of Reduviidae (Heteroptera: Cimicomorpha) based on morphological characters. Syst. Èntomol..

[40] ChŁond D. (2018). A taxonomic revision of the genus Sirthenea (Hemiptera: Heteroptera: Reduviidae) of the Old World. Zootaxa.

[41] Brożek J., Chłond D. (2010). Morphology, arrangement and classification of sensilla on the apical segment of labium in Peiratinae (Hemiptera: Heteroptera: Reduviidae). Zootaxa.

[42] Nowińska A., Brożek J. (2017). Morphological study of the antennal sensilla in Gerromorpha (Insecta: Hemiptera: Heteroptera). Zoomorphology.

[43] Taszakowski A., Nowińska A., Brożek J. (2019). Morphological study of the labial sensilla in Nabidae (Hemiptera: Heteroptera: Ci-micomorpha). Zoomorphology.

[44] Amyot C.J.B., Serville A. (1843). Histoire Naturelle des Insectes.

[45] Maddison W.P., Maddison D.R. Mesquite: A Modular System for Evolutionary Analysis, Version 3.5. http://www.mesquiteproject.org.

[46] Goloboff P.A., Farris J.S., Nixon K.C. (2003). T.N.T.: Tree Analysis Using New Technology. Program and Documentation. http://www.zmuc.dk/public/phylogeny/tnt.

[47] Goloboff P.A., Farris J.S., Nixon K.C. (2008). TNT, a free program for phylogenetic analysis. Cladistics.

[48] Goloboff P.A. (1999). Analyzing Large Data Sets in Reasonable Times: Solutions for Composite Optima. Cladistics.

[49] Nixon K.C. (1999). The parsimony ratchet, a new method for rapid parsimony analysis. Cladistics.

[50] Nixon K.C. WinClada ver. 1.0000 Published by the Author, Ithaca, NY, USA, 1999. http://www.cladistics.com.

[51] Schouteden H. (1913). Reduviidae, Nabidae et Pyrrhocoridae recueillis au Congo para le Dr. J. Bequaert. Rev. Zool. Afr..

[52] Stål C. (1864). Hemiptera africana/descripsit Carolus Stal. Hemiptera Afr./Descripsit Carolus Stal..

[53] Walker F. (1873). Catalogue of the specimens of Hemiptera: Heteroptera in the collection of the British Museum. Part VIII Print. Trustees London.

[54] Gerstaecker C.E.A., von der Decken C.C. (1873). Gliederthiere (Insekten, Arachniden, Myriopoden undIsopoden). Baron Carl Claus von der Decken’s Reisen in Ost-Afrika in Den Jahren 1859–1865.

[55] Stål C., Stål C. (1874). Enumeratio Reduviidarum Europae, Africae, Asiae, et Australiae. Enumeratio Hemipterorum, IV..

[56] Zhang G., Weirauch C. (2014). Molecular phylogeny of Harpactorini (Insecta: Reduviidae): Correlation of novel predation strategy with accelerated evolution of predatory leg morphology. Cladistics.

[57] Zhang J., Weirauch C., Zhang G., Forero D. (2016). Molecular phylogeny of Harpactorinae and Bactrodinae uncovers complex evolution of sticky trap predation in assassin bugs (Heteroptera: Reduviidae). Cladistics.

