# Supplementary material for: Revision and Phylogenetic Analysis of the Genus Phonoctonus Stål, 1853 (Heteroptera, Reduviidae, Harpactorinae)
Source: Insects. 2021 Dec 8;12(12):1100. doi: 10.3390/insects12121100 (PMC8705300; doi:10.3390/insects12121100)
Supplement: Supplementary file 1 [file insects-12-01100-s001.zip › Supporting information S2 Detailed list of examined materials.pdf]

Supporting information

Revision and phylogenetic analysis of the genus *Phonoctonus* Stål, 1853 (Heteroptera, Reduviidae, Harpactorinae).

Agnieszka Bugaj-Nawrocka[1] and Dominik Chłond[2]*

[1,2] University of Silesia, Faculty of Natural Sciences, Institute of Biology, Biotechnology and Environmental Protection, Bankowa 9, 40-007 Katowice, Poland.

*Correspondence: Dominik Chłond, Faculty of Natural Sciences, Institute of Biology, Biotechnology and Environmental Protection, University of Silesia in Katowice, Bankowa 9, 40-007 Katowice, Poland.

E-mail: dominik.chlond@us.edu.pl

Supporting information S2: Detailed list of examined materials.

Detailed list of examined materials

Quoting the labels of specimens: (/) is used to divide data on different rows on the label, (;) is used to divide data on different labels, ([]) is used for authors comments.

Abbreviations used:

HNHM – Hungarian Natural History Museum, Budapest, Hungary

MHNG – Muséum d'Histoire Naturelle, Geneva, Switzerland

MMBC – Moravske Muzeum, Brno, Czech Republic

MNCN – Museo Nacional de Ciencias Naturales, Madrid, Spain

MNHN – Muséum National d'Histoire Naturelle, Paris, France

MSNM – Museo Civico di Storia Naturale, Milano, Italy

MZH – Finnish Museum of Natural History, Helsinki, Finland

NHMUK – The Natural History Museum, London, United Kingdom

NHMW – Naturhistorisches Museum Wien, Wien, Austria

NHRS – Naturhistoriska riksmuseet, Stockholm, Sweden

NMPC – National Museum (Natural History), Prague, Czech Republic

RBINS – Royal Belgian Institute of Natural Sciences, Brussels, Belgium

RMCA – Musee Royal de l'Afrique Centrale, Tervuren, Belgium

SAMC – Iziko Museum of Capetown, Cape Town, South Africa

USNM – National Museum of Natural History, Washington D.C., USA

ZMMU – Zoological Museum of the Moscow State University, Moscow, Russia

ZMUH – Zoologisches Institut und Zoologisches Museum, Universität von Hamburg, Hamburg, Germany

ZMUC – University of Copenhagen, Zoological Museum, Copenhagen, Denmark

ZSM – Zoologische Staatssammlung, Munich, Germany

1. ***Phonoctonus bifasciatus***

TYPE material: • OYEM (Gabon) / G. Le Testu; HOLOTYPE; Museum Paris (MNHN).

Material examined:

• GABON / Makokou - Colline / Mission biologique / 13.X.67 / G. Bernardi; Museum Paris (MNHN). • Camerun / merid.; Nat. Hist. Mus / Budapest / coll. Hemiptera (HNHM).

2. ***Phonoctonus caesar***

TYPE material: • Camerun.; Sjöstedt.; Phonoctonus / Caesar Hagl. / ♀ Typ!; Typus; NHRS-GULI / 000000556 (NHRS).

Material examined:

• Museé du Congo / Danga / 1913 / M: Hutereau; R. Dét. / D / 2580 (RMCA). • Museé du Congo / Lukolela / -IX-1923 / Lt J. Ghesquière; R. Dét. / D / 2580 (RMCA). • Museé du Congo / Kamerun / Coll. Schouteen; Phonoctonus Caesar Hagl.; R. Dét. / E / 2580 (RMCA). • Museé du Congo / Congo Belge: / Tanganyka: Kasange / Coll. Schouteden; R. Dét. / E / 2580 (RMCA). • Coll. Mus. Congo / Tshuapa: Lukolela / De Guide (RMCA). • Congo Belge. P.N.A. / R. Tungula piste Mwenda / Katuka 1000 m; 20-IV-948 / réc. J. de Wilde 59; Coll. Mus. Tervuren; Phonoctonus / Caesar Haglund / A. Villiers det. 1951 (RMCA). • Congo belge P.N.A. / R. dr. Mangbeleu 950 m / forêt 24-III-1949 / J. de Wilde 218 (RMCA). • Is Fernando Poo / Bahia de S. Carlos / I-III 1902 / L. Fea (MNHN). • Is Fernando Poo / Bahia de S. Carlos / I-III 1902 / 0-200m / L. Fea (MNHN). • Muséum Paris / Cameroun / B. de Miré; N Kolbisson / 3-II-70 (MNHN). • Cameroun / Bipindi; Phonoctonus / Caesar / Haglund. / A. Villiers det.; Muséum Paris / don / A. Villeirs (MNHN). • Kamerun. / Dendeng. / 16.IV.1914. / 1919-4 (NHMUK). • G.R. 70; GOLD COAST / Ashanti / Nyabo / 24.III.1947 / J. Bowden / On cocoa farm.; Imp. Inst. Ent. / Coll. no 10631; Pres by / Imp. Inst. Ent. / B.M. 1947-29 (NHMUK).

3. ***Phonoctonus elegans***

TYPE material: • Phonoctonus / elegans / S. Var / Kamerun; Sintipo; MNCN / Cat. Tipos Nº / 8352; MNCN_Ent / 104115 (MNCN).

Material examined:

• HOLOTYPUS; Museé du Congo / Bumbuli / I-IV-1915/ R. Mayné; R. Dét. / J. / 2581; Phonoctonus elegans v. stricta Sch. / Types (RMCA). • [7x] PARATYPUS; Museé du Congo / Bumbuli / I-IV-1915/ R. Mayné; R. Dét. / J. / 2581 (RMCA). • PARATYPUS; Museé du Congo Belge / Kasai: Hibo / (Don A. Shévy); R. Dét. / J / 2581 (RMCA). • PARATYPUS; Museé du Congo / Sankuru: Komi / 23-IV-1930 / J. Ghesquière; R. Dét. / J / 2581 (RMCA). • PARATYPUS; Museé du Congo / Ingende / 24-XII-1914 / R. Mayné; R. Dét. / J. / 2581 (RMCA). • PARATYPUS; Museé du Congo / Bena Bendi / V-1915 / R. Mayné; R. Dét. / J. / 2581 (RMCA). • PARATYPUS; Museé du Congo /

Equateur: Bokote / 1-II-1927 / R.P. Hulstaert; R. Dét. / J. / 2581 (RMCA). • PARATYPUS; Museé du Congo / Sankuru: Lonkala / -III-1925 / Lt J. Ghesquière; R. Dét. / J. / 2581 (RMCA). • PARATYPUS; Museé du Congo / Sankuru: Yomi / VII-1930 / J. Ghesquière; R. Dét. / J. / 2581 (RMCA). • PARATYPUS; Museé du Congo / Sankuru: Lomela / -IV-1925 / J. Ghesquière; R. Dét. / J. / 2581 (RMCA). • Congo; Kuilu. / NHRS-GULI; 000006189 (NHRS). • Gabun / Staudgr. / NHRS-GULI; 00000690 (NHRS). • Congo; Kuilu. / NHRS-GULI; 000006191 (NHRS). • [♀] Urw. Mawambi / Grauer, 1910 (NHMW). • [♂] Urwald-Beni / Sept. Okt. 10. Grauer (NHMW). • Kamerun. / Sardi nr. / Dengdeng / 9.IV.1914 / 1919-4 (NHMUK). • Belgian Congo: / Beni / Ituri Forest. / IX.1946; T.H.E. Jackson. / B.M. 1946-354 (NHMUK). • CRS Nigeria 29-30.XII.81 / N.E. Calabar – 99 km / Mc e Rd. Edge R.P [unreadable] / Main forest – WB% mature, / From mixen sample je Reid; 14; CIE A18541; Phonoctonus / sp. / det. M.S.K. Ghauri, 1987 (NHMUK). • Congo belge / Bambesa / 15-16-X-1937 / J. Vrydagh; R. Mus. Hist. Nat. / Belg. I.G. 11.927; Phonoctonus / elegans / Varela / A. Villiers det. 1969 (RBINS). • Bambesa / 26-vii-1937 / J. Vrydagh; R. Mus. Hist. Nat. / Belg. I.G. 11.472 (RBINS). • Congo belge / Bambesa / 13-ix-1937 / J. Vrydagh; R. Mus. Hist. Nat. / Belg. I.G. 11.948; Phonoctonus / elegans / v. stricta Sch. / A. Villiers det. 1969 (RBINS). • Congo belge / Bambesa / 1-ix-1937 / J. Vrydagh; Muséum Paris / don / A. Villiers (MNHN). • [2x] Musée du Congo / Haut-Uele: Moto / -IV-V-1923 / L. Burgeon; R. Dét. / L / 2581 (RMCA). • Musée du Congo / Bas-Uele: Koteli / 1-21-I-1925 / Dr H. Schouteden; R. Dét. (RMCA). • Musée du Congo / Poko - Nala - Rungu / 1912 / Me Hutereau; R. Dét. (RMCA). • Coll. Mus. Congo / Kasai: Djeka (Lodja) / I-1954 / R. Roiseux (RMCA). • Musée du Congo / Uelé: Dingila / VI-1933 / H.J. Bredo; Phonoctonus / elegans var. (RMCA). • Musée du Congo / Ubangi: Gemena / III-1936 / C. Léontovitch (RMCA). • Mus. Congo / Bambesa / 17.III.-1937 / J. Vrydagh (RMCA). • Mus. Congo / Bambesa / X-1937 / J. Vrydagh (RMCA). • Mus. Congo / Bambesa / 6-IV-1937 / J. Vrydagh (RMCA). • Mus. Congo / Bambesa / 4-V-1937 / J. Vrydagh (RMCA). • [2x] Mus. Congo / Bambesa / I-II 1934 / H.J. Bredo (RMCA). • [2x] Mus. Congo / Bambesa / XI-1933 / H.J. Bredo (RMCA). • [4x] Mus. Congo / Bambesa / X-1933 / H.J. Bredo (RMCA). • Mus. Congo / Bambesa / I-1934 / H.J. Bredo (RMCA). • Mus. Congo / Bambesa / -1938 / P. Hernard (RMCA). • Mus. Congo / Bambesa / 11-V-1938 / P. Hernard (RMCA). • Mus. Congo / Bambesa / 15-IV-1937 / J. Vrijdagh (RMCA). • Mus. Congo / Bambesa / 3-IX-1937 / J. Vrijdagh (RMCA). • Museé du Congo / Eala / III-1935 / J. Ghesquière (RMCA). • Museé du Congo / Eala / 30-VIII-1933 / A. Corbisier (RMCA). • Museé du Congo / Bambesa / I-II-1934 / H.J. Bredo (RMCA). • Museé du Congo / Bambesa / II-1934 / H.J. Bredo (RMCA). • Museé du Congo / Bambesa / X-1933 / H.J. Bredo (RMCA). • Museé du Congo / Bambesa / 11-V-1937 / J. Vrydagh (RMCA). • Coll. Mus. Congo / Yangambi / M. Ferrand (RMCA). • Congo Belge: Bangui / 3.XII.51 Leg. R. Mussard; Phonoctonus / fasciatus / (Pal. Beauv.) / det. Rédei D., 2009 (MHNG). • Congo Belge: Kamituga / II.1952 Leg. R. Mussard (MHNG). • [2x] Coll. Mus. Tervuren / Oubanghi-Chari: / Bangui I/III.1968 / ex. coll. Breuning (RMCA). • [2x] Coll. R. I. Sc. N. B. / Congo Bel: Mosanga / Dist. Tshuapa / Massart (USNM). • Coll. R. I. Sc. N. B. / Congo Belge / Rwankwi / 31-V-1951 / J. V. Leroy (USNM).

4. *Phonoctonus fairmairei*

TYPE material: • HOLOTYPE / Juillet; Museum Paris / Congo / Brazzaville / Mission Chari-Tchad / D[r] J. Decorse 1904 (MNHN).

Material examined:

• Camerun / merid.; Nat. Hist. Mus / Budapest / coll. Hemiptera (HNHM). • [4x♀] Mawambi. Ukaika, / Grauer, Nov. Dez. 10 (NHMW). • [1x♂, 1x unknown] Ukaika, Mawambi / 1911. Grauer (NHMW). • [3x] DINGILA / MAI. 1933 / H.J. BREDO; Pres. by / Imp. Inst. Ent. / B.M. 1933-521 (NHMUK). • DINGILA / MAI. 1933 / H.J. BREDO; Pres. by / Imp. Inst. Ent. / B.M. 1933-521; Phonoctonus / elegans (NHMUK). • Dingila, Uele Ibimbiri / Mr. Lugard / Sept. Nov., 1930 / Soudan Gort.; 3s; Ent. Coll. / C9591 (NHMUK). • 4,500 ft. / June 18-21.2011. / S.A.Neave.; Brit.E.Africa. / Ilala. / Maramas Dist. / 14 m. E of Mumias; Pres.by / Imp.Bur.Ent. / Brit. Mus. / 1927-85. (NHMUK). • Musée du Congo / Dingila / H.J.Bredo; E1 / 1-4-33 / Parasite of Dysdercus supersitiosus; Phonoctonus / elegans / Varela? / Det. B. Uvarov 1933; Pres.by / Imp. Inst. Ent. / B.M. 1933-415. (NHMUK). • Iambuga / R. Aruwimi / (Borry); Distant Coll. / 1911-383.; Phonoctonus / elegans Varela? / det. W.E. China 1931 (NHMUK). • Entebbe / Uganda / 13.III.1913 / No. 1644 (NHMUK). • Uganda / Magunsa / Coffe Estete / Nimpumu / 24.III.1912 / Miss. M. Robertson; Pres.by / Imp. Bur. Ent. / Brit. Mus. / 1927-85. (NHMUK). • Lukungu / Ch. Haas; Phonoctonus / elegans Varela / Det. N.C.E. Miller 1952; Mus. Zool. Helsinki / Loan No. / HE 2013-34 (MZH). • Congo belge / Musosa / x-1939 / H.J. Brédo; R. Mus. Hist. Nat. / Belg. I.G. 13.212; Phonoctonus / fasciatus / fairmairei / A. Villiers det. 1972 (RBINS). • Congo belge / Mpese / 16-VII-1937 / J. Mertens; R. Mus. Hist. Nat. / Belg. I.G. 11.438 (RBINS). • Congo belge / Ngowa / V.VI-1939 / R.P.J. Mertens; R. Mus. Hist. Nat. / Belg. I.G. 12.537 (RBINS). • Congo belge / Bambesa / 5-viii-1937 / J. Vrydagh nº 45; R. Mus. Hist. Nat. / Belg. I.G. 11.467 (RBINS). • Congo belge / Bambesa / 3-i-1940 / J. Vrydagh nº 114; R. Mus. Hist. Nat. / Belg. I.G. 13.009 (RBINS). • Congo belge / Bambesa / 22-23-ix-1937 / J. Vrydagh; R. Mus. Hist. Nat. / Belg. I.G. 11.458 (RBINS). • Bambesa / 26-viii-1937 / J. Vrydagh; R. Mus. Hist. Nat. / Belg. I.G. 11.472 (RBINS). • [2x] Maniema / 1953 / P.C. Lafivra; Coll. R. Mayné / Comm. Et. Bois Congo / R. 2482; R.I.Sc.N.B. / I.G. 22.863 (RBINS). • Congo belge / Kaniama / 2.ii.1939 / H.J. Brédo; R. Mus. Hist. Nat. / Belg. I.G. 12.371 (RBINS). • Stanleyfalls / Malfeyt; R.I.Sc.N.B. / I.G. (RBINS). • Leopoldville / 3,6,1903 / Wilmin; R.I.Sc.N.B. / I.G. (RBINS). • [2x] Ngowa / xi-1938 / J. Mertens; R. Mus. Hist. Nat. / Belg. I.G. 12.142 (RBINS). • Congo; R.M.H.N.B 16.364 / Coll. J. Muller:; Phonoctonus / picturatus / Fairm. (RBINS). • [3x] Stanleyville / Congo; R.M.H.N.B. 16.364 / Coll. J. Muller; Phonoctonus / picturatus / Fairm. (RBINS). • [3x] Lukungu / Ch. Haas (RBINS). • Stanleyfall / Malfeyt (RBINS). • Popocabacca / E. Loos (RBINS). • Stanleyville / Congo; R.M.H.N.B. 16.364 / Coll. J. Muller: (RBINS). • Gabon; R.I.Sc.N.B. / I.G. (RBINS). • Coll. Mus. Tervuren / Lualaba: Kolwezi / X-1953 / Dr V. Allard (RMCA). • [3x] Coll. Mus. Tervuren / Zaïre: Mambasa (K.I) / III 1971 / J. Tarerniers (RMCA). • [9x] Museé du Congo / Maniema / 1936 / P. Henrard (RMCA). • Coll. Mus. Congo / Basoko / VIII-1948 / P.L.G. Benoit (RMCA). • Museé du Congo / Uele: Dingila / -1-VIII-193~~2~~3 / H.J. Bredo (RMCA). • Museé du Congo / Uele: Dingila / 1-VI-1933 / H.J. Bredo (RMCA). • Museé du Congo / Uele: Dingila / 15-VI-1933 / H.J. Bredo (RMCA). • Museé du Congo / Uele: Dingila / 4-VI-193~~2~~3 / H.J. Bredo (RMCA). • [2x] Museé du Congo / Uelé: Dingila / 21-VI-1933 / J. Leroy (RMCA). • Museé du Congo / Uelé: Dingila / 5-VII-1933 / J. Leroy (RMCA). • Col. Mus. Congo / Bambesa / V-1938 / H.J. Bredo (RMCA). • [6x] Museé du Congo / Bambesa / I-II-1934 / H.J. Bredo (RMCA). • [5x] Museé du Congo / Bambesa / I-1934 / H.J. Bredo (RMCA). • Museé du Congo / Bambesa / X-1933 / H.J. Bredo (RMCA). • Coll. Mus. Congo / Bambesa / 10-V-1937 / J. Vrydagh (RMCA). • Coll. Mus. Congo / Bambesa / -IV-1937 / J. Vrydagh (RMCA). • [2x] Museé du Congo / Bambesa / 25-III-1937 / J. Vrydagh (RMCA). • [2x] Museé du Congo / Bambesa / 8-VII-1937 / J. Vrydagh (RMCA). • [8x] Mus. Congo / Bambesa / 8-IV-1935 / J. Vrydagh (RMCA). • Museé du Congo / Bambesa / 4-V-1937 / J. Vrydagh (RMCA). • Museé du Congo / Bambesa / 10-V-1937 / J. Vrydagh (RMCA). • [2x] Museé du Congo / Bambesa / 9-IV-1937 / J. Vrydagh (RMCA). • [5x] Museé du Congo / Bambesa / IV-1937 / J. Vrydagh (RMCA). • [2x] Museé du Congo / Bambesa

/ 24-IV-1937 / J. Vrydagh (RMCA). • [2x] Museé du Congo / Bambesa / X-1937 / J. Vrydagh (RMCA). • Museé du Congo / Bambesa / 22- ~~IX~~ XI-1937 / J. Vrydagh (RMCA). • Museé du Congo / Bambesa / 1-VII-1937 / J. Vrydagh (RMCA). • Museé du Congo / Bambesa / 14-II- ~~III~~ -1938 / J. Vrydagh (RMCA). • Museé du Congo / Bambesa / 28-VIII-1937 / J. Vrydagh (RMCA). • Museé du Congo / Bambesa / 16- ~~II~~ -III-1939 / J. Vrydagh (RMCA). • Coll. Mus. Congo / Bambesa / XII-1946 / P.L.G. Benoit (RMCA). • Coll. Mus. Congo / Bambesa / 15-X-1933 / J. Leroy (RMCA). • [2x] Coll. Mus. Congo / Maniema: Katongo / IX-1936 / P. Hernard (RMCA). • [3x] Coll. Mus. Congo / Bas-Congo: Lemful / X-XII-1944 / Rév. P. De Beir (RMCA). • [2x] Coll. Mus. Congo / Bas-Congo: Mayidi / 1942 / R.P. Van Eyen (RMCA). • Coll. Mus. Congo / Bas-Congo: Sanda / 19… / Courtois (RMCA). • [5x] Coll. Mus. Congo / Bas-Congo / Lemful VI-1945 / Rév. P. De Beir (RMCA). • [8x] Coll. Mus. Congo / Bas-Congo / Lemful V-1945 / Rév. P. De Beir (RMCA). • Coll. Mus. Congo / Bas-Congo / Lemful II-1945 / Rév. P. De Beir (RMCA). • Coll. Mus. Tervuren / Bas-Congo: Kisantu / V.1945 / R.F. Anastase (RMCA). • Museé du Congo / Bomokandi (sourcus) / 26-XI/6-XII-1925 / S.A.R. Prince Léopold (RMCA). • Mus. Congo / Uele: Oganda / 22-XI-1937 / J. Vrydagh (RMCA). • [5x] Coll. Mus. Congo / Mayidi / 1945 / Rév. P. Van Eyen (RMCA). • [15x] Coll. Mus. Congo / Mayidi / 1942 / Rév. P. Van Eyen (RMCA). • Coll. Mus. Congo / Mayidi / 1943 / Rév. P. Van Eyen (RMCA). • [3x] Museé du Congo / Kafakumba / III-1932 / F.G. Overlaet (RMCA). • [2x] Museé du Congo / Kafakumba / II-1932 / F.G. Overlaet (RMCA). • Coll. Mus. Congo / Yangambi / XI-1937 / P. Henrard (RMCA). • Coll. Mus. Congo / Kibali-Ituri: Yindi / X-1948/III-1949 / A. E. Bertand (RMCA). • Coll. Mus. Congo / Brabanta (Basongo) / IV/V-1949 / P. Henrard (RMCA). • Museé du Congo / Uéle: Dingila / 21-X-1932 / J. Vrydagh (RMCA). • Museé du Congo / Kasai –X-1942 / Lt J. Ghesquière (RMCA). • [6x] Coll. Mus. Congo / Terr. de Dibaya / Kamponde 1945 / Rév. Fr. Allaer. (RMCA). • Museé du Congo / Kunungu (N'Kele) / -1973 / (Coll. Schouteden) (RMCA). • Museé du Congo / Penge-Bamboli / VI-1933 / Putnam (RMCA). • Museé du Congo / Uelé: Bambesa / -1933 / Lefêrre (RMCA). • Museé du Congo / Uele - Itimbiri: Bondo / 4-IV-1933 / J. Vrydagh (RMCA). • Museé du Congo / Stanleyville / V-1926 / J. Ghesquière (RMCA). • Museé du Congo / Stanleyville / 19/25-IX-1925 / A. Collart; R. Dét. / H / 2581 (RMCA). • Museé du Congo / Stanleyville / 1/3-I-1930 / A. Collart; R. Dét. / H / 2581 (RMCA). • Museé du Congo / S. Kamerun / Fanggebiet / Coll. Schouteden; Sûr Kamerun / Fanggebiet; Phonoctonus / picturatus / Fairm.; R. Dét. / H / 2581 (RMCA). • Museé du Congo / Bas-Uélé: Bambesa / 5-VII-1922 / (Degang); R. Dét. / H / 2581 (RMCA). • [5x] Museé du Congo / Bas-Uélé: Bambesa / 3-VII-1922 / (Degang); R. Dét. / H / 2581 (RMCA). • Museé du Congo / Mongende / 19-IV-1921 / Dr H. Schouteden; R. Dét. / H / 2581 (RMCA). • Museé du Congo / Uelé: Kongoli / 5-VII-1914 / Dr. Rodhain; R. Dét. / H / 2581 (RMCA). • Leopoldville / Belg. Kongo / G. Frey I.1952; Coll. Mus. Congo / ex. coll. G. Frey (RMCA). • Museé du Congo / Ubangi: Libenge / IV-1936 / C. Léontovitch (RMCA). • Museé du Congo / Ubangi: Gemena / 24-VI-1937 / C. Léontovitch (RMCA). • Museé du Congo / Mongbwalu (Kilo) / -1939 / Mme Scheitz (RMCA). • Museé du Congo / Ruwenzori: Kurukwata / 13-VI-1935 / H.J. Bredo (RMCA). • Museé du Congo / Lulua: Jandoa / X-1930 / G.F. Overlaet; R. Dét. / H / 2581 (RMCA). • Museé du Congo / Katanga: Sawamba / 10-V-1923 / F.G. Overlaet; R. Dét. / H / 2581 (RMCA). • [2x] Museé du Congo / Uele: / Van Kerkhovenville / Degreef; R. Dét. / H / 2581 (RMCA). • Museé du Congo / Kikwit / ~~XI~~-1920 / P. Vanderijst (RMCA). • [3x] Museé du Congo / Kikwit / ~~X~~-1920 / P. Vanderijst (RMCA). • Coll. Mus. Congo / Lokandu / -III-1939 / Capt. Marée (RMCA). • Museé du Congo / Kinshasa -1928 / (Chanainesses de Saint Augustin) (RMCA). • Museé du Congo / Congo Belge: / Lukungu / Coll. Schouteden; R. Dét. / H / 2581 (RMCA). • Museé du Congo / Tshofa / XII-1934 / Mme Gillardin (RMCA). • Museé du Congo / Itoka / X-1912 / R. Mayné (RMCA). • Museé du Congo / Uelé: Lakulu / -1928/32 / Vandenbranden (RMCA). • Museé du Congo / DIMA 24.IX.08

/ A. Koller; R. Dét. / H / 2581 (RMCA). • Museé du Congo / Mayumbe: Zobe / 4an12-I-191 / R. Mayné; R. Dét. / H / 2581 (RMCA). • Museé du Congo / Poko-Nala-Rungu / 1-1912 / Me Huterau; R. Dét. / H / 2581 (RMCA). • Museé du Congo / Ponthierville / 22-X-1910 / Dr. Bequaert; R. Dét. / H / 2581 (RMCA). • Museé du Congo / Tua / Jin 6-1913 / Dr. J. Maes; R. Dét. / H / 2581 (RMCA). • Museé du Congo / Maniema / -1922 / 35 / (Blommaert); R. Dét. / H / 2581 (RMCA). • Museé du Congo / Haut-Ituri: Faradje / VI-1915 / Blommaert; R. Dét. / H / 2581 (RMCA). • Museé du Congo / Uele: / Dungu / De Greeft; R. Dét. / H / 2581 (RMCA). • Museé du Congo / Léopoldville / -1933 / A. Tinant (RMCA). • Coll. Mus. Congo / Sankuru: Djeka / 1955/1956 / R. Roiseux (RMCA). • Coll. Mus. Congo / Station de Gandajika / I.N.E.A.C. 1637 1957 / P. de Francquen (RMCA). • [3x] Maniema / 1953 / P.C. Lefivre; Coll. R. Mayné / Comm. Et. Bois Congo / R.2482; Coll. Mus. Congo / Don R. Mayné (RMCA). • [3x] Museé du Congo / Lulua: Sandoba / IV-1932 / F.G. Overlaet (RMCA). • [2x] Museé du Congo / Lulua: Sandoba / X.1930 / F.G. Overlaet; R. Dét. / H / 2581 (RMCA). • [2x] Museé du Congo / Lulua: Sandoba / V 1932 / F.G. Overlaet (RMCA). • Museé du Congo / Lomami: Kiabukwa / -III-V-1932 / P. Quarré (RMCA). • Coll. Mus. Tervuren / Kasongo / III 1960 / P.L.G. Benoit (RMCA). • [7x] Mus. Roy. Afr. Centr. / Uele: Bambesa / 16.XII.1938 / J. Vaydagh; R. Dét. / 7768 (RMCA). • Congo belge: P.N.U. / Kaziba (1.140 m.) / 7-12-II-1948 / Mis. G.F. de Witte. 1258a; Coll. Mus. Tervuren; Phonoctonus / fasciatus Sign / A. Villiers det., 1952 (RMCA). • Congo: Bouloungui / 17-III-73 / sans cottonieur (MNHN). • Museum Paris / Ile M'Bamou / Cameroun / XII 1868. Grillot (MNHN). • Congo Belge / Bambesa / 29-X-1937 / J. Vrydagh (MNHN). • Zoo / Brazzaville / 10-3-1953; Museum Paris / Congo, 1960 - 1965 / O RSTOM / J.P. Adam rec. (MNHN). • Kadjuju (Kiwu) / Congo belge (MNHN). • Gabon / Makokou – Colline / Mission biologique / 13.X.67 / G. Bernardi (MNHN). • Congo / Brazzaville / Brazzaville / Coll. J. Coffin / VIII 1968 (MNHN). • Bas Congo / Lemfu III-1945 / R.P. de Beer; Muséum Paris (MNHN). • Djoumouna / 21-09-71; Recolteur / J.P. Geillot; Museum Paris (MNHN). • Congo belge: P.N.U. / Mukana (1810 m.) / 15-19-I-1948 / Mis. G.F. de Witte. 1235a (MNHN). • Uele: Bambesa / 16.XII.193… / J. Vrydagh (MNHN). • Bambesa / I-1934 / H. J. Bredo (MNHN). • Bambesa / 7.ix.1937 / J. Vrydagh; Muséum Paris / don / A. Villeirs (MNHN). • Bambesa / 7.x.1937 / J. Vrydagh; Muséum Paris / don / A. Villeirs (MNHN). • Bambesa / 6.xi.1937 / J. Vrydagh; Muséum Paris / don / A. Villeirs (MNHN). • Bambesa / 23.ix.1937 / J. Vrydagh; Muséum Paris / don / A. Villeirs (MNHN). • Bambesa / x.1938 / J. Vrydagh; Muséum Paris / don / A. Villeirs (MNHN). • Coll. R. I. Sc. N. B. / Rep. Dem. CONGO / Nord – Kivu / Beni 1 150 m / Coll. R. Ducarme (USNM). • [2x] Congo Belge / Bambesa / 28-IX-1937 / J. Vrydagh; R. Mus. Hist. Nat. / Belg. I. G. 11.713 (USNM). • Bambesa / Grotte de Gwane / 27-VIII-1939 / J. Vrydagh; R. Mus. Hist. Nat. / Belg. I. G. 11.391 (USNM).

5. *Phonoctonus fasciatus*

TYPE material: Neotype: • R. fasciatus / Pal. de Bauv. / Guinea; Mus. Westerm; ♂ (ZMUC).

Material examined:

• Kilimandjaro / Sjöstedt. 1905-6; Kibonoto / 1300 - 1900 m; 25 april / NHRS-GULI / 000006448 (NHRS). • Camerun / merid.; Nat. Hist. Mus / Budapest / coll. Hemiptera (HNHM). • Phonocotnus / fasciatus Beauv. / det. Dioli 1981; Guinea Port. / Bubaque / VI-56 Benassi (MSNM). • [♂] fasciatus / det. Mayr. (NHMW). • [♂] Guinea / Coll. Signoret.; Beauvoisié / det. Stal (NHMW). • [3x] Nigeria.

/ A.W.J. Pomeroy. / B.M. 1925-575. (NHMUK). • Cameron S. / Escalera. / 1903-355. (NHMUK). • Ibadan Nigeria / On Rice leaves / B.A.B. 26.8.64; R4; Phonoctonus sp. / M.S.K. Ghauri dst. 1965 (NHMUK). • B. Cameroons: / Kumba. / 7.X.1949. / H. Oldroyd. / B.M. 1950-2. (NHMUK). • Cameroons. / Escalera. / 1903-355. (NHMUK). • B. Cameroons: / Kumba. / 17.X.1949 / B.M. 1950-2. (NHMUK). • B. Cameroons: / Kumba. / 7.X.1949 / H. Osdroyd. / B.M. 1950-2. (NHMUK). • 4-3 / 36; Harpactor. / fasciatus; Walker's Catal. (NHMUK). • Osomba CRS Nigeria / 13.9. [unreadable] / etc Edge by road / je Reid; 16; CIE A18541 (NHMUK). • Abijang CRS Nigeria / 20-22.1.88. Semi- / deciduous forest / area je Reid; 33; CIE A20099; Phonoctonus / ?fasciatus / Beauvois / det. G. Stonedahl, 1988 (NHMUK). • S. Nigeria: / Ibadan. / 10.I.1923 / A.W.J.Pomeroy; Pres.by / Imp.Bur.Ent. / Brit. Mus. / 1923-459.; S.177.22 (NHMUK). • S. Nigeria: / Ibadan. / 27.III.1923. / A.W.J.Pomeroy; Pres.by / Imp.Bur.Ent. / Brit. Mus. / 1923-459.; S.154.22 (NHMUK). • S. Nigeria: / Ibadan. / 27.II.1923. / A.W.J.Pomeroy; Pres.by / Imp.Bur.Ent. / Brit. Mus. / 1923-459.; S.138.22 (NHMUK). • [2x] S. Nigeria: / Ibadan. / 30.III.1923. / A.W.J.Pomeroy; Pres.by / Imp.Bur.Ent. / Brit. Mus. / 1923-459.; 9.203-13 (NHMUK). • S. Nigeria: / Ibadan. / 12.I.1923. / A.W.J.Pomeroy; Pres.by / Imp.Bur.Ent. / Brit. Mus. / 1923-459.; S.171-22 (NHMUK). • S. Nigeria: / Ibadan. / 6.III.1923. / A.W.J.Pomeroy; Pres.by / Imp.Bur.Ent. / Brit. Mus. / 1923-459.; S.148-22 / 6-3-23 (NHMUK). • S. Nigeria: / Ibadan. / 3.III.1923. / A.W.J.Pomeroy; Pres.by / Imp.Bur.Ent. / Brit. Mus. / 1923-459.; S.148-22 / 3-3-23 (NHMUK). • S. Nigeria: / Ibadan. / 28.II.1923. / A.W.J.Pomeroy; Pres.by / Imp.Bur.Ent. / Brit. Mus. / 1923-459.; S.148-22 / 28/2/23 (NHMUK). • S. Nigeria: / Ibadan. / 27.1.1923. / A.W.J.Pomeroy; Pres.by / Imp.Bur.Ent. / Brit. Mus. / 1923-459.; S.162-22 / 27/2/23 (NHMUK). • S. Nigeria: / Ibadan. / 13.1.1923. / A.W.J.Pomeroy; Pres.by / Imp.Bur.Ent. / Brit. Mus. / 1925-459.; S.137.22 (NHMUK). • S. Nigeria: / Ibadan. / 29.III.1923. / A.W.J.Pomeroy; Pres.by / Imp.Bur.Ent. / Brit. Mus. / 1923-459. (NHMUK). • S. Nigeria: / Ibadan. / 12.IV.1923. / A.W.J.Pomeroy; Pres.by / Imp.Bur.Ent. / Brit. Mus. / 1923-425.; S.203-C (NHMUK). • S. Nigeria: / Ibadan. / 12.IV.1923. / A.W.J.Pomeroy; Pres.by / Imp.Bur.Ent. / Brit. Mus. / 1923-423.; S.146.22 (NHMUK). • Nigeria / Ibadan / 1924-25 / J.D. Golding / Bred.; Pres. by / Imp.Bur.Ent. / Brit. Mus. / 1926-118.; P ♂ (NHMUK). • Nigeria / Ibadan / 1924-25 / J.D. Golding / Bred.; Pres. by / Imp.Bur.Ent. / Brit. Mus. / 1926-118.; F2 ♂ (NHMUK). • Nigeria / Ibadan / 1924-25 / J.D. Golding / Bred.; Pres. by / Imp.Bur.Ent. / Brit. Mus. / 1926-118.; F1 ♂ (NHMUK). • [4x] Nigeria / Ibadan / 1924-25 / J.D. Golding / Bred.; Pres. by / Imp.Bur.Ent. / Brit. Mus. / 1926-118.; Spare / F4 ♂ (NHMUK). • [2x] Nigeria / Ibadan / 1924-25 / J.D. Golding / Bred.; Pres. by / Imp.Bur.Ent. / Brit. Mus. / 1926-118.; Spare / F4 ♀ (NHMUK). • [2x] Nigeria / Ibadan / 1924-25 / J.D. Golding / Bred.; Pres. by / Imp.Bur.Ent. / Brit. Mus. / 1926-118.; Spare / F2 ♀ (NHMUK). • Nigeria / Ibadan / 1924-25 / J.D. Golding / Bred.; Pres. by / Imp.Bur.Ent. / Brit. Mus. / 1926-118.; F3 ♂ (NHMUK). • Nigeria / Ibadan / 1924-25 / J.D. Golding / Bred.; Pres. by / Imp.Bur.Ent. / Brit. Mus. / 1926-118.; P ♀ (NHMUK). • Nigeria / Ibadan / 1924-25 / J.D. Golding / Bred.; Pres. by / Imp.Bur.Ent. / Brit. Mus. / 1926-118.; F3 ♀ (NHMUK). • Nigeria / Ibadan / 1924-25 / J.D. Golding / Bred.; Pres. by / Imp.Bur.Ent. / Brit. Mus. / 1926-118.; Spare / F2 ♂ (NHMUK). • Nigeria / Ibadan / 1924-25 / J.D. Golding / Bred.; Pres. by / Imp.Bur.Ent. / Brit. Mus. / 1926-118.; F3 ♂ (NHMUK). • Nigeria / Ibadan / 1924-25 / J.D. Golding / Bred.; Pres. by / Imp.Bur.Ent. / Brit. Mus. / 1926-118.; F1 ♀ (NHMUK). • Nigeria / Ibadan / 1924-25 / J.D. Golding / Bred.; Pres. by / Imp.Bur.Ent. / Brit. Mus. / 1926-118.; F5 ♂ (NHMUK). • NIGERIA / Ibadan / 1924-25 / J. D. Golding / Bred.; Pres.by / Imp.Bur.Ent. / Brit. Mus. / 1926-118; F4 ♂ (NHMUK). • [3x] Nigeria. / A.W.J. Pomeroy. / B.M. 1925-575. (NHMUK). • Cameroous. / Escalera. / 1903-365. (NHMUK). • Uganda, / Kampala, / 1-10.I.1917 / C.C. Gowdey; 1918-65 (NHMUK). • fasciatus / P.B.; Isubu. / No.; Distant Coll. / 1911-383 (NHMUK). • West Africa / Fernando Po. / Rev. W. Cooper.; Brit. Mus. 1925-176. (NHMUK). • Kogin Sirikin / Pawa, N. Nigeria.

/ Nov. 1910 / J.W. Scott-Macfie. / 1911-417. (NHMUK). • Kamerun / Mbili nr. / Badere. / 19.IV.1914. / 1914-4 (NHMUK). • Nyasaland. / S.E. shore L. Nyasa. / Btwn. Ft. Maguire / & Ft. Johnson. / 6-7 Mch. 1910 / S.A. Neave (NHMUK). • Gold Coast / Aburi / 1912-13 / W.H.Patterson; Phonoctonus / fasciatus / P.B. / Det. B. Uvarov. (NHMUK). • [3x] Cameroous: / Batouri District. / Lat. 3.45.N. Long. 13.45.E. / 1-17.III.1935. / F.G. Merfield.; Brit. Mus. 1935-473. (NHMUK). • H/1038/2; Mozawa / 29-X-36 / Rama; Brit. Mus. / 1966-336 (NHMUK). • H/1038/1; Phonoctonus / fasciatus / S.B. Leaf / 26:10:53; Brit. Mus. / 1966-336 (NHMUK). • Van Someren / Bwamba valley / July 1945.; Coll. Inst. Ent. / Coll. No. 10445; Brit. Mus. / 1965-274 (NHMUK). • S. Nigeria: / Ibadan. / 6.II.1923. / A. W. J. Pomeroy; Pres. by / Imp. Bur. Ent. / Brit. Mus. / 1925-459.; R.B.C. (NHMUK). • B.E. Africa: / Lake Victoria, Is. / Hyagwe Coast / Mango Bay. / 9.II.1919. / Dr. G.D.H. Carpenter. / 1920-201; 823 ERB (NHMUK). • NIGERIA / Ibadan / 1924-25. / J.D. Golding / Bred.; Pres. by / Imp. Bur. Ent. / Brit. Mus. / 1926-118.; Spare / F4. ♂ (NHMUK). • Uganda / Mpumu / miss M. Robertson; Pres. by / Imp. Bur. Ent. / Brit. Mus. / 1927-85.; Note resemblance / to common cotton / bug (NHMUK). • NIGERIA / Ibadan / 1924-25. / J.D. Golding / Bred.; Pres. by / Imp. Bur. Ent. / Brit. Mus. / 1926-118.; Spare / F2. ♂ (NHMUK). • 51 / *107 / West Afric; fasiatus Pal. Beauv / Cape Coast. 563.; Harpactor. / fasciatus. / Walker's Catal. (NHMUK). • Entebbe, / Uganda. / 26.8.11. / C. C. Gowdey. / 1912-101. (NHMUK). • Entebbe, / Uganda. / 6 June, 1913. / C. C. Gowdey.; Pres by. / Imp. Bur. Ent. / Brit. Mus. / 1927-78. (NHMUK). • 279. / SENEGAL / ~~Bambey~~ / Bowake / J. Risbec. (NHMUK). • Entebbe, / Uganda. / 18 June, 1913. / C. C. Gowdey.; Pres. by / Imp. Bur. Ent. / Brit. Mus. / 1927-78.; Phonoctonus / fasciatus / P.B. / Det. B. Uvarov. (NHMUK). • Uganda. / Entebbe. / 14.V.12. / C. C. Gowdey / 1913-42.; Phonoctonus / fasciatus P. de. B. (NHMUK). • Nigeria / Ibadan / 1924-25. / J.D. Golding / Bred.; Pres. by / Imp. Bur. Ent. / Brit. Mus. / 1926-118.; Spare / F1. ♂. (NHMUK). • Entebbe, / Uganda. / Sep.1912. / C.A.Wiggins.; 1913-171 (NHMUK). • Uganda, / Kampala / 1-12.9.1915 / C.C.GODWEY; 1915-413 (NHMUK). • Juabin, / Ashanti. / A.E. Evans.; 1914-521 (NHMUK). • San Pedo, / French Ivory Coast. / Guy Chetwynd. / 1915-306. (NHMUK). • Entebbe, / Uganda. / Sep. 1912. / C.A. Wiggins.; 1913-171 (NHMUK). • Congo belge / Libenge / 27.XI -1947 / R. Cremer - M. Neuman; R.I.Sc.Nat.Belg. / I.G. 16.655; Phonoctonus / fasciatus / Pal. Beauv. / A. Villiers det 195 (RBINS). • Congo belge / Lac Tumba / Loondo 1940-1947 / Gonze de Loneux / Nº 34; R. Mus. Hist. Nat. / Belg. I.G. 15.945 (RBINS). • Congo belge / Ngowa / x.xi.1938 / R.P.J. Mertens; R. Mus. Hist. Nat. / Belg. I.G. 12.068 (RBINS). • Congo belge: / District du Bas-Congo / Mayumbe: vi-1936; R. Mus. Hist. Nat. / Belg. I.G. 11.680 (RBINS). • Gandajika 1953 / de Francquen / E.B; Coll. R. Mayné / Comm. Et. Bois Congo / R. 2494; R.I.Sc.N.B. / I.G. 22.863 (RBINS). • Ngowa / 30-x-1932 / R.P.J. Mertens; R. Mus. Hist. Nat. / Belg. I.G. 12.068 (RBINS). • Museé du Congo / Lulua: Kapanga / VI-1933 / G.F. Overlaet (RMCA). • Museé du Congo / Ubangi: Libenge / XII-1935 / C. Léontovitch (RMCA). • Museé du Congo / Eala / VII-1933 / A. Corbisier (RMCA). • Museé du Congo / Kabinda / -1935 / P. Henrard (RMCA). • Umg. Kamerunberg / Ekona, 5.-11.10. 1935.; Dr. F. Zumpt leg. / Eing. Nr. 7, 1936.; Coll. Mus. Tervuren / Coll. H. Schouteden (RMCA). • Vielma / Kamerun; Coll. Mus. Tervuren / Coll. H. Schouteden (RMCA). • Togo; Coll. Mus. Tervuren / Coll. H. Schouteden (RMCA). • Abutshi / R. Niger; Coll. Mus. Tervuren / Coll. H. Schouteden (RMCA). • Abutshi / R. Niger; no 89; Coll. Mus. Tervuren / Coll. H. Schouteden (RMCA). • Umg. Kamerunberg / Victoria, 1.-10.11.35.; Dr. F. Zumpt leg. / Eing. Nr. 7, 1936.; Coll. Mus. Tervuren / Coll. H. Schouteden (RMCA). • NO. Tanganyika / Urundi 16.11.97 / Ramsay & Hösemann S.; Coll. Mus. Tervuren / Coll. H. Schouteden (RMCA). • [2x] Museé du Congo / Ituri: Forêt de Kawa / 18-IV-1929 / A. Collart; R. Dét. / C / 2581 (RMCA). • [2x] GABON: / Tchibanga / 28.3.1986 / A Panly leg. / aubergine (RMCA). • Museé / du Congo Belge / BANZVILLE / Royaux; R. Dét. / D / 2581 (RMCA). • [2x] Museé du Congo / Maniema / -1942 / 35. / (Blammaert); R. Dét.

/ D / 2581 (RMCA). • Museé du Congo / Kamerun: / Victoria / Coll. Schouteden; R. Dét. / D / 2581 (RMCA). • Museé du Congo / Nyangwe / V-1918 / R. Mayné; R. Dét. / D / 2581 (RMCA). • Museé du Congo / Uelé: Mamor / 23-VI-1914 / Dr. Rodhain; R. Dét. / D / 2581 (RMCA). • Museé du Congo / Inkisi / V-1925 / S.A.R. Prince Leopold; R. Dét. / D / 2581 (RMCA). • [3x] Museé du Congo / Maniema: Kasongo / 1936 / P. Henrard (RMCA). • [5x] Coll. Mus. Congo / Maniema: Kasongo. / IX-1936 / P. Henrard; Récolté sur cotonnier (RMCA). • Coll. Mus. Congo / Bas-Congo: Lemfu / I-1945 / Rév. P. De. Beir (RMCA). • [35x] Coll. Mus. Congo / Yakoma / -IX-1939 / Leontovitch (RMCA). • Coll. Mus. Congo / Bosodula / 15-X-1941 / J. Vrydagh; S / Jatropha / Curcas (RMCA). • Coll. Mus. Congo / Ubangi: Libenge / 26-I-1936 / C. Léontovitch (RMCA). • Coll. Mus. Congo / Ubangi: Libenge / 31-I-1936 / C. Léontovitch (RMCA). • Museé du Congo / Uelé: Doruma / 236 / -1934 / R.P. De Graer (RMCA). • Museé du Congo / Bandouinville / 16-I-1933 / L. Burgeon (RMCA). • Museé du Congo / Maniema : / -1936 / 15. / P. Henrard (RMCA). • [2x] Museé du Congo / Lomami champ / de coton –IX.1936 / 14.T. / P. Hernard (RMCA). • Coll. Mus. Congo / Bambesa / 15-X-1934 / J. Leroy (RMCA). • Coll. Mus. Congo / Station de Gandajika / I.N.E.A.C. 1799 1957 / P. de Francquen (RMCA). • Coll. Mus. Congo / Station de Gandajika / I.N.E.A.C. 1801 1957 / P. de Francquen (RMCA). • [4x] Coll. Mus. Tervuren / Cote d'Ivoire: Bingerville / V.1964 / J. Decelle; B / R. Dét. / 7756 (RMCA). • Coll. Mus. Tervuren / Cote d'Ivoire: Bingerville / XI.1961 / J. Decelle (RMCA). • Coll. Mus. Tervuren / Cote d'Ivoire: Bingerville / II.1964 / J. Decelle (RMCA). • Coll. Mus. Tervuren / Cote d'Ivoire: Bingerville / II.1964 / J. Decelle; Phonoctonus / fasciatus Beau. / A. Villiers det 1986 (RMCA). • Coll. Mus. Tervuren / Cote d'Ivoire: Bingerville / X.1963 / J. Decelle (RMCA). • Coll. Mus. Tervuren / Terr. De Kasongo, / Riv. Lumami II.1960 / P.L.G. Benoit (RMCA). • Coll. Mus. Tervuren / Maniema: Kasongo / VIII/IX.1959 / P.L.G. Benoit; Phonoctonus / fasciatus P. d. B. / A. Villiers det. 1967 (RMCA). • Coll. Mus. Tervuren / Ubangi: Boyasegne / XI-1957 / Cotonco; Phonoctonus / fasciatus P. d. B. / det. A. Villiers 1967 (RMCA). • Coll. Mus. Tervuren / Maniema: Wamaza / III/IV.1957 / Cotonco; Phonoctonus / fasciatus P. d. B. / det. A. Villiers 1967 (RMCA). • Coll. Mus. Congo / Lac Léopold II: Bokalakala 1957 / N'Kele; Phonoctonus / fasciatus P. d. B. / det. A. Villiers 1967 (RMCA). • Coll. Mus. Tervuren / Zaire: Kinshasa / 1 – 1986 / P. M. Elsen (RMCA). • Museé du Congo / Eala / TP / 29-VIII-1915 / R. Mayné (RMCA). • Congo Belge P.N.A. / Katuka (Kikingi) / 12 10-1946 / J. de Wilde: 20; Coll. Mus. Tervuren; A. Villiers det. 1952 / Phonoctonus / fasciatus / immitis Stal. (RMCA). • [3x] Museé du Congo / de Luebo / à Luluabourg -1921 / Lt. J. Ghesquière; R. Dét. / 2582 (RMCA). • [9x] Museé du Congo / Kikwit / -XI-1920 / P. Vanderijst; R. Dét. / 2582 (RMCA). • Museé du Congo / de Luebo à Mloi / -II-1921 / L. Achteu; R. Dét. / 2582 (RMCA). • [19x] Museé du Congo / Kikwit / 1920 / P. Vanderijst; R. Dét. / 2582 (RMCA). • [10x] Museé du Congo / Kikwit / ~~X~~ -1920 / P. Vanderijst; R. Dét. / 2582 (RMCA). • [8x] Museé du Congo / Kikwit / -XII-1920 / P. Vanderijst; R. Dét. / 2582 (RMCA). • [2x] Museé du Congo / Luluabourg / P. Callewaert; R. Dét. / 2582 (RMCA). • [3x] Musée du Congo / Kasai : Ipamu / -1922 / P. Vanderijst; R. Dét. / 2582 (RMCA). • [9x] Museé du Congo / Region Kwilu - Kasaï / II-III-1921 / P. Vanderijst; R. Dét. / 2582 (RMCA). • Museé du Congo / Gemro / (Jancl.); R. Dét. / 2582 (RMCA). • [38x] Museé du Congo / Nyangwe / -~~II~~-1920 / Lt. Ghesquière; R. Dét. / 2582 (RMCA). • Museé du Congo / Nyangwe / 11-V-1930 / (R. Steyaeut) (RMCA). • Museé du Congo / Kunungu / -1938 / (NKele) / Dr H. Schouteden (RMCA). • Museé du Congo / Kunungu / 4-IV-1921 / Dr H. Schouteden (RMCA). • Museé du Congo / Kunungu / -1938 / Dr H. Schouteden (RMCA). • [14x] Museé du Congo / Ubangi: Libenge / XII-1935 / C. Léontovitch (RMCA). • [6x] Museé du Congo / Kabinda / -1935 / P. Henrard (RMCA). • Museé du Congo / Lulua: Kapanga / -XII-1932 / F.G. Overlaet (RMCA). • [2x] Museé du Congo / Lulua: Kapanga / -II-1932 / F.G. Overlaet (RMCA). • [2x] Museé du Congo / Lulua: Kapanga / -IX-1932 / F.G. Overlaet (RMCA). • Museé du Congo / Lulua: Kapanga

/ -XI-1933 / F.G. Overlaet (RMCA). • Museé du Congo / Lulua: Kapanga / -III-1933 / F.G. Overlaet (RMCA). • [4x] Museé du Congo / Eala / -1929 / (M. Maotsens) / ~~A. Corbisier~~ (RMCA). • [5x] Museé du Congo / Eala / VII-1933 / A. Corbisier (RMCA). • [3x] Museé du Congo / Eala / V-1932 / H.J. Brédo (RMCA). • [6x] Museé du Congo / Eala / 30-VIII-1933 / A. Corbisier (RMCA). • [2x] Museé du Congo / Eala: / -IV-1932 / H.J. Brédo (RMCA). • Museé du Congo / Eala / 14/29-X-1929 / H.J. Brédo (RMCA). • Museé du Congo / Eala / 4-X-1931 / H.J. Brédo (RMCA). • Museé du Congo / Eala / 17-4-1932 / H.J. Brédo (RMCA). • Museé du Congo / Eala / -I-1935 / J. Ghesquière (RMCA). • Museé du Congo / Eala / 4-VII-1933 / A. Corbisier (RMCA). • Museé du Congo / Eala / -XI-1934 / J. Ghesquière (RMCA). • [22x] Museé du Congo / Sankuru: Lodja / III-1929 / L. Ghesquière; R. Dét. / 2582 (RMCA). • Museé du Congo / Maniema / -1922 / 35. / (Blammaert) (RMCA). • [3x] Museé du Congo / Stanleyville / -XII-1929 / Lt. J. Ghesquière; R. Dét. / 2582 (RMCA). • Museé du Congo / Ganganijka / Kigoma –IX-1918 / R. Mayné; R. Dét. / 2582 (RMCA). • Museé du Congo / Kunungu -1938 / (NKele) / (coll. Schouteden) (RMCA). • Museé du Congo / Kunungu -1932 / Réc. NKele / (Coll. Schouteden) (RMCA). • Museé du Congo / Kunungu (NKele) / -1934 / (Coll. Schouteden) (RMCA). • Museé du Congo / Mayumbe: Zobe / 4an 12-I-1916 / R. Mayné; R. Dét. / 2582 (RMCA). • Museé du Congo / ~~Equateur: Bokoro~~ / Abissanduga / 11-1-26 / R.P. Hulstaert (RMCA). • Museé du Congo / Kasai / -XI-1924 / Lt J. Ghesquière (RMCA). • Museé du Congo / Barumbu / -VI-1925 / Lt J. Ghesquière (RMCA). • Museé du Congo / Mayumbe / VII-1917 / R. Mayné; R. Dét. / 2582 (RMCA). • Museé du Congo / Mayumbe 24-XI-15 / Makaia N'Tete / R. Mayné; R. Dét. / 2582 (RMCA). • Museé du Congo / Mayumbe: Tshela / 23-27-II-1916 / R. Mayné; R. Dét. / 2582 (RMCA). • [2x] Museé du Congo / Mayumbe IX-XI-1917 / Makaia N'Tete / R. Mayné; R. Dét. / 2582 (RMCA). • Museé du Congo / Kibali-Ituri: Mahagi / 1932 / Ch. Scope (RMCA). • Museé du Congo / Equateur: / Vevlaine (RMCA). • [5x] Museé du Congo / Eshofa / XII-1934 / Mme Gillardin (RMCA). • [2x] Museé du Congo / Leverville / 1928 / Mme J. Tinant (RMCA). • Museé du Congo / Urundi: Kitega / VII-VIII-1934 / Lefèvre (RMCA). • Museé du Congo / Pania - Mutombo / 9-I-1935 / Mme Gillardin (RMCA). • Museé du Congo / Banana / XI-1934 / P. Henrard. (RMCA). • Museé du Congo / Libengé / 19-XII-1931 / H.J. Brédo (RMCA). • [2x] Museé du Congo / Libengé / V-1938 / Leontovitch (RMCA). • Museé du Congo / Libenge / -I-1937 / Leontovitch (RMCA). • [2x] Museé du Congo / Basongo / -X-1921 / L. Achten; R. Dét. / 2582 (RMCA). • [4x] Coll. Mus. Tervuren / Maniema: Kasongo / VIII/IX.1959 / P.L.G. Benoit; Phonoctonus / fasciatus / immitis St. / A. Villiers det 1967 (RMCA). • Coll. Mus. Tervuren / Maniema: Terr. Kasongo, / Mufala 1960 / Mission P.L.G. Benoit; Phonoctonus / fasciatus / immitis St. / A. Villiers det 1967 (RMCA). • [5x] Museé du Congo / Bumbuli / I-1914 / Dr. J. Plaes; R. Dét. / 2582 (RMCA). • Museé du Congo / Mushie / II-1914 / Dr. J. Plaes (RMCA). • Coll. Mus. Congo / Sankuru: Tshumbe S. Marie / 4-VI-1948 / Don Dr R. Mouchamps (RMCA). • [2x] Coll. Mus. Congo / Bokalakala (Bolobo) / 1954 / R.C. Eloy (RMCA). • Museé / Du Congo Belge / Kwango: Atene / Charlier; R. Dét. / 2582 (RMCA). • Museé du Congo / Galli – Koko / Kasai / R. Carlier; R. Dét. / 2582 (RMCA). • [2x] Mus. Roy. Afr. Centr. / Terr. de Kasongo, / riv. Lumami I 1960 / P.L.G. Benoit (RMCA). • Museé du Congo / DIMA 30.IX.08 / A. Koller; R. Dét. / 2582 (RMCA). • Museé du Congo / Congo da Lemba / I-1913 / R. Mayné; R. Dét. / 2582 (RMCA). • Museé du Congo / Yambata / 10-XII-1912 / R. Mayné; R. Dét. / 2582 (RMCA). • Museé du Congo / Bakusu / 9-1912 / R. Mayné; R. Dét. / 2582 (RMCA). • [21x] Museé du Congo / Maniema: / 1936 / P. Henrard (RMCA). • [12x] Museé du Congo / Maniema: Kasongo / IX-1936 / P. Henrard (RMCA). • [13x] Museé du Congo / Kasongo / -IX-1936 / P. Henrard (RMCA). • [4x] Coll. Mus. Congo / Gandajika / 1947 / P. Henrard (RMCA). • [2x] Coll. Mus. Congo / Kivu: Kitwabaluzi / 1946 / P. Henrard (RMCA). • [6x] Coll. Mus. Congo / Terr. de Dibaya / Kamponde 1945 / Rév. Fr. Allaer. (RMCA). • [2x] Coll. Mus. Congo / Kasongo / VIII/IX.1959 / P.L.G. Benoit

(RMCA). • Coll. Mus. Congo / Kasongo / III-1960 / P.L.G. Benoit (RMCA). • [2x] Coll. Mus. Congo / Libenge / -II-1938 / Leontovitch (RMCA). • [12x] Coll. Mus. Congo / Libenge / V-1938 / Leontovitch (RMCA). • [23x] Coll. Mus. Congo / Libenge / 16-V-1938 / Leontovitch (RMCA). • [4x] Coll. Mus. Congo / Libenge / XII-1938 / Leontovitch (RMCA). • [3x] Coll. Mus. Congo / Sankuru: Gandajika / 163 / 1952 / P. de Francquen (RMCA). • Coll. Mus. Congo / Sankuru: Gandajika / 00163 28.IX.1950 / P. de Francquen (RMCA). • [5x] Coll. Mus. Congo / Sankuru: Gandajika / 00163 24.VIII.1950 / P. de Francquen (RMCA). • [2x] Coll. Mus. Congo / Bas-Congo / Lemfu VI-1945 / Rév. P.L. De Beir (RMCA). • Coll. Mus. Congo / Léopoldville / VII-1945 / J.M. Berteaux (RMCA). • Coll. Mus. Congo / Kivu: Bukavu / 1946 / L. Herinck (RMCA). • Coll. Mus. Congo / Mayidi / 1944 / Rév. P. Van Eyen (RMCA). • [2x] Coll. Mus. Congo / Ubangi: Bumba / ~~28 IX~~ 1956 / J. Eugéne (RMCA). • Museé du Congo / Lac Tanganyka: / Nyanza 28-XII-1932 / L. Burgeon (RMCA). • Coll. Mus. Congo / Tanganyka: Albertville / IV/~~V~~-1955 / H. Bomans (RMCA). • [3x] Museé du Congo / Lomami: champ / de coton –IX-1936 / 14.T / P. Hernard (RMCA). • Museé du Congo / S.W. Maniema: / 19 / Lufungulu VI-1936 / P. Hernard (RMCA). • [2x] Coll. Mus. Congo / Luluabourg: Katoka / 1939 / R.P.N. Vankerckhoven (RMCA). • Museé du Congo / prés du Lomani / 16. / VIII-1936 / P. Hernard (RMCA). • Museé du Congo / Bas-Congo: Manzadi / -1937 / Dr. Dartevelle (RMCA). • Museé du Congo / Manyema / Kindu -26-III-1918 / R. Mayné (RMCA). • Phonoctonus / fasciatus; Mozambique / Lourenco Marques P.E.A / 1949/04/01; SAM-HEM- / A003642 (SAMC). • G. Heinrich / Gilletts, w. of / Durban, NATAL; Phonoctonus / fasciatus / Beauv. / A. Villiers det 195; Zoologische / Staatssammlung / München (ZSM). • Bim- B. dar - Senegal / 9/8/1977; Phonoctonus / spec. / det. J. Deckert 1997; Zoologische / Staatssammlung / München (ZSM). • Togo, fl. Amu; Phonoctonus / fasciatus P. Beauv. / Det. N.C.E. Miller 1952; Mus. Zool. Helsinki / Loan No. / HE 2013 - 28 (MZH). • Mus. Westerm. (ZMUC). • Côte d'Ivoire / p. Cachan / Adiopodoumé 29-4-53 (MNHN). • Côte d'Ivoire / p. Cachan / Assakra 20-3-54 (MNHN). • III 1948 / Dundo / Angola (MNHN). • Museum Paris / 12.1930- IV.1931 / Ch. Allraud / & P.A. Chappuis; Cote d'Ivoire / Daloa (MNHN). • [2x] Museum Paris / Mission / A. DESCARPENTRIES et. A. Villiers / 1963-1964; SIBITI / CONGO / XI-1963 (MNHN). • Gandajika / Mai-juin-1986; Cottonier (MNHN). • Ubangi / L. Alliard / Don Lameors (MNHN). • Lamto (Toumodi) / Côte d'Ivoire / Forêt galerie / 29.10.1969 (MNHN). • Gabon / Crique-Tsini / G. Favarel 1914 (MNHN). • Assathea / 20-3-54 / B.-COTE-D'IVOIRE B (MNHN). • Museum Paris / Côté d'Ivoire / Bingerville / G. Melou 1914 (MNHN). • Museum Paris / Gabon / Belledame 1883 (MNHN). • Museum Paris / Nimba (Guinée) / M. Lamotte 11.IV.42; Keoulenta (MNHN). • Congo Belge / Libenge 20-Xi-1947 / Mission Mawuya / R. Cremer – M. Neuman (MNHN). • R. Delattre Rec. / Bounké / nº: 96 (MNHN). • Congo; Coll. Noualhier 1898 (MNHN). • Gabon; Coll. Noualhier 1898 (MNHN). • [3x] Museum Paris / Congo Franç. / Env de Brazzaville / M'Bamou / Montezer 1903 (MNHN). • [2x] Museum Paris / Congo Franç. / Libreville / Chalot 1898 (MNHN). • Museum Paris / Congo Belge / Kikou / Ouvira / Guy Babault 1927 (MNHN). • Museum Paris / Am Dam et A Béché / J. Bonnauf 1912 (MNHN). • ~~IFAN~~ – 194 / Dündo / Angola; ~~IFAN~~ XII 1948 / Angola A. de / Banos Machado / B-142; Muséum Paris / don / A. Villiers (MNHN). • ~~IFAN~~ – 1949 / Dündo / Angola; ~~IFAN~~ I 1949 / Angola A. de / Banos Machado / B-142; Muséum Paris / don / A. Villiers (MNHN). • Mai 1959 / Brazzaville / Congo L. Vincent (MNHN). • Museum Paris / Nimba (Guinée) / M. Lamotte II. VI. 42; Mt. To (1600 m) / Camp 1 (MNHN). • [2x] Senegal / Casamance / 25.VIII.1980 / B. Sigwalt leg.; Muséum Paris (MNHN). • Coll. Mus. Tervuren / Côte d'Ivoire : Bingerville / V.1964 / J. Decelle (MNHN). • [2x] III IFAN – 1948 / Dündo / Angola; III ~~IFAN~~ – 1948 / Angola A. de / Banos Machado / B-142; Muséum Paris / don / A. Villiers (MNHN). • IFAN / NIMBA (Guinée) / Lamotte et Roy / VII-XII 51; SM-18 (MNHN). • Museum Paris / Nimba (Guinée) / M. Lamotte II.VI.42; Nion (MNHN). • Sur

Ficus / Glumosa; Mifergui / Alt. 700 m / Guinée / Monts Nimba / 24 Avril 1993 / H. Perrin rec. (MNHN). • Yaoundé / Cameroun / Molez (MNHN). • Cote d'Ivoire / Taï / 21.6.79 / G. Couturier leg. (MNHN). • Champ de riz; Cote d'Ivoire / Taï / 25.IV.79 / G. Couturier leg. (MNHN). • Adiopodoume; Côte d'Ivoire / P. Cachan / W-B-Labo / 31-x-1-xi-58 (MNHN). • Côte d'Ivoire / Loc: Lamto / date: 18.06.65 / Gillon (MNHN). • [3x] Côte d'Ivoire / Loc: Bonako Toro / date: 14.10.70 / D. Duriard réc. (MNHN). • Lamto (Toumodi) / Côte d'Ivoire / 14.5.65 (MNHN). • Cote d'Ivoire / 18.2.1980 / G. Couturier leg.; Défrichement; Champ de /riz; Biotope nº12 / bloc Cavally (MNHN). • [2x] Cote d'Ivoire / Taï / 18.5.1979 / G. Couturier leg.; Défrichement; Polyculture / biotope nº11; Sangbikro (MNHN). • [3x] Cote d'Ivoire / Taï / 22.4.1979 / G. Couturier leg.; Défrichement; Polyculture / biotope nº11; Sangbikro (MNHN). • Muséum Paris / Nimba (Guinée) / M. Lamotte II. IV.42; Yalanzou (MNHN). • Phonoctonus fasciatus Beauv. / R. Jeaunel det.; Museum Paris / Cote d'Ivoire / Rég. De San Pedro / G. Thoiré 1901 (MNHN). • [4x] Museum Paris / Cote d'Ivoire / Rég. De San Pedro / G. Thoiré 1901 (MNHN). • NOVEMBRE; Museum Paris / Guinée Française / Dixme Foulah / Prés Konakry / P. Chamanaud 1919 (MNHN). • DECEMBRE; Museum Paris / Dar-Banda Mérid. / Krébédjé (Fort Sibut) / Mission Chari-Tchad / Dr J. Decorse 1904 (MNHN). • Museum Paris / Cote D'Ivoire / Bingerville / G. Melou 1914 (MNHN). • [2x] Museum Paris / Congo Franç. / Haute-Sanga / P.A. Ferrière 106-97 (MNHN). • [2x] SEPTEMBRE; Museum Paris / Haut Qubangui / Fort de Possel / Mission Chari-Tchad / Dr J. Decorse 1904 (MNHN). • Forêt classée / de Bignona / 19-XI-1961; Mission IFAN / en / Basse-Casamance (MNHN). • Museum Paris / Dahomey / env. de Porto-Novo / Waterlot 1908 (MNHN). • [2x] Nimba (Guinée) / Lamotte, Amiet, / Vanderplaetsen / XII 56 – V 57; Ziéla / barrie 57 (MNHN). • MARS; Museum Paris / Dahomey / Plat. De Zagnanado & Ketou / Region Forest. des Hollis / D'Adia-Quéré Illémon / P. Ducorps 1910 (MNHN). • Gabon (MNHN). • Museum Paris / Nimba (Guinée) / M. Lamotte II. VI. 42; Toulereon (MNHN). • Museum Paris / Nimba (Guinée) / M. Lamotte II. VI. 42; Dieché (MNHN). • [2x] IFAN / Nimba (Guinée) / Lamotte et Roy / VII-XII 51 (MNHN). • [2x] Coll. Mus. Tervuren / Côte d'Ivoire: Bingerville / V. 1964 / J. Decelle (MNHN). • Coll. Mus. Tervuren / Nord Côte d'Ivoire: / Ferkessédougou / J. Decelle 10/20.V.1964 (MNHN). • Region Macenta / Guinée 1953 / R. Pujoi rec. (MNHN). • Cameroun / Baigom / rég. Barmoun; Cafeier (MNHN). • Sibiti / Congo / XI-1963; Muséum Paris / Mission / A. Descarpentries / et A. Villiers / 1963-1964 (MNHN). • R. Delattre Rec. / Bouaké nº>96 (MNHN). • Congo Belge / Libenge / 22.X.1947 / R. Cremer – M. Neuman; R. I. Sc. Nat. Belg. / I. G. 16 655 (MNHN). • Ubangi / L. Altard / Don Lamecre (MNHN). • [3x] Hibiscus-Sculentus; Lamto (Toumodi) / Côte d'Ivoire / 15-XI-1966 / R. Vuattoux (MNHN). • XII 1949 / Angola. A. des / Barros-Machado / A. Villiers (MNHN). • Yalinga (Oubangui) / G. le Testu (MNHN). • Côte d'Ivoire / P. Cachan / baï 8-1-55 (MNHN). • Museum Paris / Mission / A. Descarpentries / et. A. Villiers / 1963-1964; Dimonika / (Mayumbe) / Congo / I-1964 (MNHN). • [5x] Olokemeji / Ibadan / Nigeria; Bridwell / collection (USNM). • [2x] Victoria / Cameroons; Collection / Rosenberg (USNM). • Victoria / Cameroons; Coll. R. I. Sc. N. B. (USNM). • [2x] Oil River W. Afr.; Ach.-Arth. Speyer / 1899; Coll. R. I. Sc. N. B. (USNM). • ElatCam / WAfrica / FHHope; 6-8-32 (USNM). • Yaounde / FrCAM / W.Afr'36 / V-29-30; Elev. / 2600 ft; VanZwal- / uwenburg / &McGough (USNM). • Yaounde / FrCAM / W.Afr'36; Altitude / 2300 ft; VanZwal- / uwenburg / &McGough (USNM). • [2x] Coll. R. I. Sc. N. B. / Congo Belge / Kibangula / 20/24-IX-1958 / L. Henry (USNM). • [3x] Coll. R. I. Sc. N. B. / Congo Belge / Ishenga-Oswe / 18-III-1953 / P. Hostie (USNM).

6. *Phonoctonus grandis*

TYPE material: • Madagasc. / Coll. Signoret; grandis / det. Signoret; SYNTUPUS / Phonoctonus / grandis Signoret. 1860 / etik. Hecher 1996 / REDV. 316/1 (NHMW). • [♀] Madagasc. / Coll. Signoret; grandis / det. Signoret; SYNTUPUS / Phonoctonus / grandis Signoret. 1860 / etik. Hecher 1996 / REDV. 316/2 (NHMW). • Madagasc. / Coll. Signoret; grandis / det. Signoret; SYNTUPUS / Phonoctonus / grandis Signoret. 1860 / etik. Hecher 1996 / REDV. 316/3 (NHMW).

Material examined:

• Museum Paris / Madagascar / Forêt Bambre / et Maevatanana / Cervoni 1907 (MNHN). • Museum Paris / Madagascar / Reg. de Sakarani / Maurice de Rothschild / 1905 (MNHN). • Tulear / Lavania; Collection / E de Bergeriu (MNHN). • Sahafary / I / Vadon - / Peyrieras; Museum Paris (MNHN). • Ramena / 1971 / Rec / Dr Niawssat; Museum Paris (MNHN). • [x2] Anjouan Bambao / 16-1-1974 / L. Matile Rec. (MNHN). • Tanandava / pres Mozambe / R. Delaure; Pre da leur / Dysdercus / flavidus (MNHN). • [3x] West Madagascar / nr. Kirindi N.P., Andranomena / Spec. Res., 27m (Andranovory / nampela), S20°08'52"E44°30'52" / 1-4.ii. 2013, M. Trýzna lgt. (MMBC) • [11x] ALDABRA: / South Island, / Dune Jean-Louis. / 13-20.III.1968. / B. Cogan & A. Hutson; Aldabra Atoll, / Royal Society / Expedition, 1967-68. / B.M. 1968-333. (NHMUK). • ALDABRA: / South Island, / Cinq Cases / 3-16.I.1968. / B. Cogan & A. Hutson; Aldabra Atoll, / Royal Society / Expedition, 1967-68. / B.M. 1968-333. (NHMUK). • Gr. Comoro, / Morotzo; Phonoctonus / grandis Sign., var; Mus. Zool. Helsinki / Loan No / HE 2013 - 30 (MZH).

7. *Phonoctonus immitis*

TYPE material: • Old-Ca- / lahar./ Stål; subimpictus Stål; Typus; NHRS-GULI / 000000558 (NHRS). • SYNTYPUS / Phonoctonus subimpictus Stål, 1865 / etik. Hecher 1996 / REDV. 315/1; Guinea / Coll. Signoret; subimpictus / det. Stål (NHMW).

Material examined:

• Staudgr. / NHRS-GULI; 000006457 (NHRS). • Congo. / NHRS-GULI; 000006456 (NHRS). • Congo; Kuilu. / NHRS-GULI; 000006458 (NHRS). • Gabun.; Staudgr.; NHRS-GULI; 000006459 (NHRS). • Gabun.; Staudgr.; NHRS-GULI; 000006460 (NHRS). • Camerun / Victoria; Phonoctonus / picturatus v. / subimpictus; Hung. Nat. Hist. Mus / Budapest / coll. Hemiptera (HNHM). • Banana / 31.03.1903 (NHMW). • [2x] Sierra Leone (NHMW). • Entebbe, / Uganda. / 20-25 April, 1914 / C.C. Cowdey.; Imperial / Bureau of / Entomology.; 1916-40 (NHMUK). • U.C. Ibadan / 16: II: 1951 / Coll. G.H. Caswell; Zea mays; Leaf; Brit. Mus. / 1966-336 (NHMUK). • Calahar; Distant Coll. / 1911-383 (NHMUK). • [2x] Kamerun. / Jaunde, / Bejoa, / Bejenge. / 1919-4 (NHMUK). • [4x] Calabar; Distant Coll. / 1911-383 (NHMUK). • subimpictus / Stal; Calabar; Distant Coll. / 1911-383 (NHMUK). • Congo; Harpactor. / fasciatus. / Walker's Catal. (NHMUK). • S. Nigeria: / Ibadan. / 12.IV.1923. / A.W.J.Pomeroy.; Pres.by / Imp.Bur.Ent. / Brit. Mus. / 1923-459.; S.264.22 (NHMUK). • S. Nigeria: / Ibadan / 1.III.1923. / A.W.J.Pomeroy.; Pres.by / Imp.Bur.Ent. / Brit. Mus. / 1923-459.; S.203 / 22.e. / 1.3.23. (NHMUK). • A.D. Peawrk / S. Nigeria; Pres.by / Imp.Bur.Ent. / Brit. Mus. / 1927-85. (NHMUK). • Kamerun. / Dendeng. / 16.IV.1914 / 1914-4 (NHMUK). • [2x] Angola; 73/66 (NHMUK). • Juabin, / Ashanti. / A.E.Evans.; 1916-259 (NHMUK). • [3x] Cameroons. / Escalera. / 1903-355. (NHMUK). • GOLD COAST / ACHINOTA / FeB 1953 / G.O.STRIDE; B.M. / 1953-128 (NHMUK). • S. Nigeria: / Ibadan. / 27.XII.1922. / A.W.J.Pomeroy.; Pres.by / Imp.Bur.Ent. / Brit.

Mus. / 1923-459.; S.179.22 (NHMUK). • JOWATI / Sierra Leone, / 19.VIII.1912 / Jas. J. Simpson.; Pres.by / Imp.Bur.Ent. / Brit. Mus. / 1927-85 (NHMUK). • Ganya / Sierra Leone, / 1.IV.1912. / Jas. J. Simpson.; Pres.by / Imp.Bur.Ent. / Brit. Mus. / 1927-85. (NHMUK). • Juabin, / Ashanti. / A.E.Evans.; 1916-259; Phonoctonus sp. / no name B.M. (NHMUK). • Cameroon: / Victoria. / July 1916 – Aug. 1917. / Cdr.F.H. FitzRoy. / 1919.-327. (NHMUK). • Nigeria. / A.W.J.Pomeroy. / B.M. 1925-575.; Ibadan 22.1.22 / S. Nigeria; S.206.22 (NHMUK). • 1171; S. Nigeria / Ibadan / 1922. / A.W.J.Pomeroy.; Pres.by / Com.Inst.Ent. / B.M. 1950-96.; Phonoctonus / sp. prob. new. / Det. B. Uvarov. (NHMUK). • [2x] GOLD COAST / ABURI / 1912-12 / W.H. PATTERSON; Pres.by / Com.Inst.Ent. / B.M. 1950-96. (NHMUK). • LIBERIA: / 1953 / W. PETERS / B.M. 1954-727 (NHMUK). • Uganda / Dwoli / 21.VI.1927. / H. Hargreaves (NHMUK). • Ivory Coast / Bouake / 20.VII.1944 / Coleno / on cotton/ seeds / 527.; Pres.by / Com.Inst.Ent. / B.M. 1948-536.; Phonoctonus / subimpictus / Stål / J.W. Evans det. 194 (NHMUK). • Nigeria. / A.W.J. Pomeroy. / B.M. 1925-575.; Ibadan 18.I.23 / S. Nigeria; S.178.22 (NHMUK). • W. Africa: / Principe 1. / 29.XII.1932. / W.H.T. Tams. / B.M. 1933-39 (NHMUK). • U.C. Ibadan / 16.II.1951 / Coll. G.H. Caswell; Zea mays; Leaf; Brit. Mus. / 1966-336 (NHMUK). • Cameroons: / Batouri District. / Lat. 3.45.N Long. 13.45.E. / I-30.III.1935. / F.G. Merfield; Brit. Mus. / 1935-473. (NHMUK). • 3,800 – 4,000 ft. / July 28 Aug. 1, 1911. / S.A.Neave.; Uganda Prot. / Between Jinja &/ Busia or Mbwago's, / E. Busoga.(Some Forest); Pres.by / Imp.Bur.Ent. / Brit. Mus. / 1927-85. (NHMUK). • BWASYBA / 7-45; IMP.Inst.Ent. / Coll.no. 10720; Brit. Mus. / 1965-274 (NHMUK). • GOLD COAST, / ACHINOTA / Feb.1953 / G.O.STRIDE; B.M. / 1953-129 (NHMUK). • van Someren / Bwamba Valley / W. Uganda / May 1954; V.G.L. van Someren / Collection. / Brit. Mus. 1959-468. (NHMUK). • [♂] Gazi b. Yangambi / Congo Belge / 4.9.54, leg. H. Franz; Phonocotnus / fasciatus Br. / E. Wagner det. 1967 (NHMW). • [2x] Lukungu / Ch. Haas; Phonoctonus / fasciatus var. / immitis (RBINS). • [5x] Boma Sundi / P. Rolin; Phonoctonus / fasciatus var. / immitis (RBINS). • Boma Sundi / P. Rolin; Ph. fasciatus / v. immitis (RBINS). • Zambi / Ch. Haas; Phonoctonus / fasciatus var. / immitis (RBINS). • [2x] Congo / G. Hoton; Phonoctonus / fasciatus var. / immitis (RBINS). • Coll. Thirot / Congo; R. Mus. Hist. Nat. / Belg. I.G 11.128 (RBINS). • Congo / Deleval; R.I.Sc.N.B. / I.G. (RBINS). • R.M.H.N.B. 16.364 / Coll. J. Muller: (RBINS). • Mayumbe.7.1899 / Don.de Contreras; R.I.Sc.N.B. / I.G. (RBINS). • Elisabethville / -i-1939 / H.J. Brédo; R. Mus. Hist. Nat. / Belg. I.G 12.371 (RBINS). • Maniema / 1953 / P.C. Lafavre; Coll. R. Mayné / Comm. Et. Bois Congo / R. 2482; R.I.Sc.N.B. / I.G. 22.863 (RBINS). • Congo belge: / District du Bas-Congo / Mayumbe: vi-1936; R. Mus. Hist. Nat. / Belg. I.G. 11.680 (RBINS). • Congo belge: / Kaniama / 2.iii.1939 / H.J. Brédo; R. Mus. Hist. Nat. / Belg. I.G. 12.371 (RBINS). • Mpese / 21.28.7.36 / J. Cooreman; R. Mus. Hist. Nat. / Belg. I.G 11.160 (RBINS). • Congo belge / Ligenge 20-xi-1947 / Mission Mawuya / R. Cremer - M. Neuman; R.I.Sc.Nat.Belg. / I.G. 16.655 (RBINS). • Congo belge / Ligenge / 27.X.1947 / R. Cremer - M. Neuman; R.I.Sc.Nat.Belg. / I.G. 16.655 (RBINS). • Congo belge / Ngowa / x.xi.1938 / R.P.J. Mertens; R. Mus. Hist. Nat. / Belg. I.G. 12.068 (RBINS). • Congo belge / Ngowa / v.vi.1939 / R.P.J. Mertens; R. Mus. Hist. Nat. / Belg. I.G. 12.537 (RBINS). • Congo-belge: Kwango / Ngowa-23-X-1937 / R.P.J. Mertens; R. Mus. Hist. Nat. / Belg. I.G. 11.520 (RBINS). • Congo-belge: Kwango / Ngowa-29-XI-1937 / R.P.J. Mertens; R. Mus. Hist. Nat. / Belg. I.G. 11.648 (RBINS). • Ngowa / 1.7.39 / J. Mertens; R. Mus. Hist. Nat. / Belg. I.G. 12.717 (RBINS). • Ngowa / 15 au 31.7.39 / J. Mertens; R. Mus. Hist. Nat. / Belg. I.G. 12.717 (RBINS). • Congo belge / Ngowa / vi-1939 / R.P.J. Mertens; R. Mus. Hist. Nat. / Belg. I.G. 12.671 (RBINS). • Ngowa / 24-VI-1939 / R.P.J. Mertens; R. Mus. Hist. Nat. / Belg. I.G. 12.142 (RBINS). • Ngowa / 12-VI-1939 / R.P.J. Mertens; R. Mus. Hist. Nat. / Belg. I.G. 12.142 (RBINS). • [2x] Gandajika 1953 / de Francquen / E.B.; Coll. R. Mayné / Comm. Et. Bois Congo / R. 2494; R.I.Sc.N.B. / I.G. 22.863 (RBINS). • [10x] Ubanghi / L. Allard / DonLameore; R.I.Sc.N.B. /

I.G. (RBINS). • Ubanghi / L. Allard / DonLameore; R.I.Sc.N.B. / I.G.; Phonoctonus/ fasciatus / var. immitis Stål / A. Villiers det. 1959 (RBINS). • [2x] Mukonje Farm / Kameroun / R. Rohde; R.I.Sc.N.B. / I.G. (RBINS). • Congo belge / Wenga Ifomi / E. Quineaux; R. Mus. Hist. Nat. / Belg. I.G. 10565 (RBINS). • Rutshuru / 20-24-VI-1934 / 442 / G.F. de Witte / Parc Nat. Albert; H. Schouteden det., 1945: / PHONOCTONUS subimpictus Stal.; Phonoctonus subimpictus. Stål / det. H. Schouteden; R. Mus. Hist. Nat. / Belg. I.G. 14.937 (RBINS). • Congo Belge P.N.A. / Nchobulo / 29-10-1946 / J-de Wilde: 19; R.I.Sc.N.B. / I.G. 24.054; A. Villiers det., 1952 / Phonoctonus / fasciatus var. / subimpictus Stål (RBINS). • Congo Belge P.N.A. / Ondo / 1200 m. 29-XII-1935 / Mission H. Damas: (A) 283; R.I.Sc.N.B. / I.G. 24.054; A. Villiers det., 1952 / Phonoctonus / fasciatus var. / immitis Stål (RBINS). • Congo-Belge: P.N.A. / Mutsora / 1939 / Hackars; A. Villiers det., 1952: / Phonoctonus / fasciatus / immitis Stål; R.I.Sc.N.B. / I.G. 24.054 (RBINS). • Congo belge / Bambesa / 30-x-1937 / J. Vrydagh; R. Mus. Hist. Nat. / Belg. I.G. 11.930; Phonoctonus / fasciatus / var. / subimpictus Stål / A. Villiers det 1959 (RBINS). • Congo belge / Bambesa / 6-xi-1937 / J. Vrydagh; R. Mus. Hist. Nat. / Belg. I.G. 11.948 (RBINS). • Congo belge / Bambesa / 15-16-x-1937 / J. Vrydagh; R. Mus. Hist. Nat. / Belg. I.G. 11.927 (RBINS). • Coll. Mus. Congo / Yangambi / 1-X-1951 / J. Decelle (RMCA). • Africa occ. / Elfenbeinküste / Abidian / 18-30.VIII.1952 / leg. L. Sheljuzhko; Phonoctonus / fasciatus / v. subimpictus / Stål / Wygodzinsky det. 1958; Zoologische / Staatssammlung / München (ZSM). • Go / Uelle Distr. / Coll. Mieheli; Phonoctonus / fasciatus / (Beauv.) / Wygodzinsky det. 1954; Zoologische / Staatssammlung / München (ZSM). • Guldkysten / 12/3 1947 / Ib Andersen (ZMUC). • Tanzania, Uluguru Mts., / Kimboza Forest, 250 m / 18.vii.1981 / M. Stoltze & N. Scharff leg. / Zool. Museum, Copenhagen (ZMUC). • Mus. Westerm.; ♂; Phonoctonus / subimpictus / Stål (ZMUC). • Mus. Westerm.; R. fummipennis / Germ. / Guinea (ZMUC). • Kongo / Waetzold (ZMUC). • Kongo / Waetfold (ZMUC). • Togo, fl. Amu; Phonoctonus /subimpictus Stål / Det. N.C.E. Miller 1952; Mus. Zool. Helsinki / Loan No. / HE 2013-32 (MZH). • Togo, fl. Amu; Phonoctonus /subimpictus Stål / Det. N.C.E. Miller 1952; Mus. Zool. Helsinki / Loan No. / HE 2013-33 (MZH). • [2x] Fayala /r. Wamba/ / Bas Congo / lgt. Lad. Skrčený; Collectio / J.L. Stenlík / Mor. Museum, Brno (MMBC). • Ghana, 5.12.1966 / Kade / lgt. A. Lelek; Collectio / J.L. Stenlík / Mor. Museum, Brno (MMBC). • Museé du Congo / Lomami-Kaniama / -III.VI-1932 / R. Massart (RMCA). • Musée du Congo / Maniema: / -1936 / P. Henrard / 22 (RMCA). • Museé du Congo / Kikwit / 1920 / P. Vanderijst (RMCA). • Coll. Mus. Congo / Bokalakala (Bolobo) / 1954 / R.C. Eloy (RMCA). • Museé du Congo / Kisantu / 1927 / R.P. Vanderyst (RMCA). • [4x] Victoria / Kamerun; Coll. Mus. Tervuren / Coll. H. Schouteden (RMCA). • [2x] Coll. Mus. Tervuren / Bas-Congo: Kisantu / 7.III.1947 / R.F. Anastase (RMCA). • Coll. Mus. Tervuren / Cameroun: Ville Kin- / sasa V/VII-1970 / Robert Brisson (RMCA). • [2x] Congo Belge: P.N.A. / 25-VIII-1951 / R. Christiaens 526-27; Secteur Nord / Kambature lieu-dit / sur piste Wat a linga / 1.150 m (RMCA). • Congo belge: P.N.A. / Rég. Oycha: 1100 m / (forêt) IV-V-1950 / J. de Wilde: 371 (RMCA). • Congo belge: P.N.A. / Mangbeleu af. Dr. Butahu / 1100 m 26-XII-1949 / J. de Wilde: 447 (RMCA). • Congo belge: P.N.A. / R. Bianeolo 1900 m piste / Mwenda-Katuka; 14-XII-49 / J. de Wilde 446 (RMCA). • Museé du Congo / Boma / 16-VII-1920 / Dr H. Schouteden; R. Dét. / 2582 (RMCA). • [2x] Museé du Congo / Bokoro / 20-III-1915 / R. Mayné; R. Dét. / 2582 (RMCA). • Museé du Congo / Bokola / X-1915 / R. Mayné; R. Dét. / 2582 (RMCA). • Museé du Congo / Wombali / VII-1913 / P. Vanderijst; R. Dét. / 2582 (RMCA). • Museé du Congo / Wombali / VII-1913 / P. Vanderijst; R. Dét. / 2582 (RMCA). • Museé du Congo / Leverville / 1928 / Mme J. Tinant (RMCA). • Lamto (Toumodi) / Côte d'Ivoire / Forét galerie / 29.10.1968; Côte d'Ivoire / C. Girard col. (MNHN). • Lamto (Toumodi) / Côte d'Ivoire / IX 1968; Côte d'Ivoire / C. Girard Col. (MNHN). • [3x] Is. Principe / Bahia de Oeste / V.1901 / 0-300 m. L.Fea; Mus. Civ. Genova (MNHN). • Calabar; Distant Coll. / 1911-383 (MNHN).

• [2x] Bismarkburg / (Togo, Guinée) / [Dr. Kraatz]; Museum Paris / Coll. Noualhier 1898 (MNHN). • Museum Paris / Congo Français / Rég. du Haut-Ivindo (Affl. Ogooué) / (Dr J. Gravot) 1906 / Cap^{e} Cottes (MNHN). • Assinie / Afrique occid. / (Treich) 1885 (MNHN). • Museum Paris / Dahomey / Athieme / (J.M. Renou) / R. Oberthur 1898 (MNHN). • Museum Paris / Congo Franç. / Env. de Brazzaville / M'Bamou / Montezer 1903 (MNHN). • Museum Paris / Congo Belge Central / Prov. de Maniema / Kindu / L. Burgeon 1911 (MNHN). • Museum Paris / Congo Franç. / Haute-Sanga / P.A. Ferriére 106-97 (MNHN). • Museum Paris / Cote d'Ivoire / Bingerville / G. Melou 1914 (MNHN). • Museum Paris / Bas-Oubangui / Ballois / 0°30' Lat. Nord / Mission Chari-Tchad / D.J. Decorse 1904 (MNHN). • Lamto (Toumodi) / Côte d'Ivoire / 13-II-1967 / R. Vygttoux / S.R.; Piège lumineux (MNHN). • Fleur / Adiopodoume / 6-12-48 (MNHN). • [2x] R. DelattreRec. / Bauaké / n°: 94 (MNHN). • Museum Paris / Côte d'Ivoire / Réserve du Banco / R. Paulian & G. Delamare (MNHN). • Museum Paris / Nimba (Guinée) / M. Lamotte II. VI. 42; Toulemon (MNHN). • Nimba (Guinée) / Lamotte, Amiet, / Vanderplaetsen / XII 56-V 57 / Ziela U.V. 18 feu. (MNHN). • Lamto (Toumodi) / Côte d'Ivoire / Foût. Galnie 20.1.1969 / C. Girard rec. (MNHN). • VII. 1964 / Rep. Centrafric. / La Maboke / J. Carayon 1964 (MNHN). • Mukonje Farm / Kameroun / R. Rohde; R.I.Sc.N.B. / I.G. (MNHN). • Ngowa / 1.7.39 / J. Mertena (MNHN). • Museum Paris / Bauaké / Côte d'Ivoire / R. Delattre. 9 (MNHN). • Côte d'Ivoire: Bingerville / I.1964 / J. Decelle (MNHN). • Côte d'Ivoire: Bingerville / IV.1964 / J. Decelle (MNHN). • Côte d'Ivoire / Jover / Adiopodoumé 6-12-48 (MNHN). • Museum Paris / Cameroun / B. de Miré; Village / Zualouma / 9-XI-65 (MNHN). • Museum Paris / Cameroun / B. de Miré; TEST/ CACAO; Me yo / 27-XI-68 (MNHN). • Museum Paris / Riv. Senito Congo / de Brazza 1892 (MNHN). • Mai 1959 / Brazzaville / Congo L. Vincent (MNHN). • Neu – Kamerun / Sardi b. Dengdeng / 30.-31.III.1914 / Mildbraed S.G. (MNHN). • Côte d'Ivoire / P. Cachan / Adiopodoumé 25-12-54 (MNHN). • Côte d'Ivoire: Koun- / Abronso / J. Decelle IX.1961 (MNHN). • Côte d'Ivoire : Divo / 28.XI.1963 / J. Decelle (MNHN). • [2x] Forêt du / Zougué / vers 800m; battage / arbistes / 19.VI.1991; Guinée / Mt Nimba / C. Girard; det. C. Weirauch 10/2008 / Phonoctonus / fasciatus var. / subimpictus (MNHN). • [5x] Côte d'Ivoire / rég. de Taï / 30.1.1985 / G. Couturier & / V. van Zeijst Réc.; ORSTOM-Paris / Mission UNESCO; Biotope n° 23 / Frondaison; Forêt dense / Humide / Sempervirente (MNHN). • Cote d'Ivoire / rég. de Taï / 16.2.1985 / G. Couturier & / V. van Zeijst Réc.; ORSTOM-Paris / Mission UNESCO; Biotope n° 21; Emvirons / Village de / Gouleako / Forêt / Secondaire / de 17 ans (MNHN). • Cote d'Ivoire / rég. de Taï / 18.2.1985 / G. Couturier & / V. van Zeijst Réc.; ORSTOM-Paris / Mission UNESCO; Biotope n° 21; Emvirons / Village de / Gouleako / Forêt / Secondaire / de 17 ans (MNHN). • [2x] Cote d'Ivoire / rég. de Taï / 21.1.1985 / G. Couturier & / V. van Zeijst Réc.; ORSTOM-Paris / Mission UNESCO; Biotope n° 21 / Sous bois; Emvirons / Village de / Gouleako / Forêt / Secondaire / de 17 ans (MNHN). • [2x] Cote d'Ivoire / rég. de Taï / 16.1.1985 / G. Couturier & / V. van Zeijst Réc.; ORSTOM-Paris / Mission UNESCO; Biotope n° 21 / Sous bois; Emvirons / Village de / Gouleako / Forêt / Secondaire / de 17 ans (MNHN). • [2x] Cote d'Ivoire / rég. de Taï / 17.1.1985 / G. Couturier & / V. van Zeijst Réc.; ORSTOM-Paris / Mission UNESCO; Biotope n° 21; Emvirons / Village de / Gouleako / Forêt / Secondaire / de 17 ans (MNHN). • [2x] Cote d'Ivoire / rég. de Taï / 14.2.1985 / G. Couturier & / V. van Zeijst Réc.; ORSTOM-Paris / Mission UNESCO; Biotope n° 21; Emvirons / Village de / Gouleako / Forêt / Secondaire / de 17 ans (MNHN). • Cote d'Ivoire / rég. de Taï / 19.2.1985 / G. Couturier & / V. van Zeijst Réc.; ORSTOM-Paris / Mission UNESCO; Biotope n° 21; Emvirons / Village de / Gouleako / Forêt / Secondaire / de 17 ans (MNHN). • [3x] Cote d'Ivoire / rég. de Taï / 23.1.1985 / G. Couturier & / V. van Zeijst Réc.; ORSTOM-Paris / Mission UNESCO; Biotope n° 21; Emvirons / Village de / Gouleako / Forêt / Secondaire / de 17 ans (MNHN). • Cote-d'Ivoire / Taï / 14.2.78 / G. Couturier leg.; Frondaison / Foret dense / sempervirente (MNHN). • Cote-d'Ivoire / Taï

/ 17.1.78 / G. Couturier leg.; Défrichment / Metec (MNHN). • Cote-d'Ivoire / Taï / 26.1.78 / G. Couturier leg.; Défrichment / Metec (MNHN). • [2x] Man / Côte d'Ivoire / M. Houillier / P. Doho / 15-11-61 (MNHN). • Cote d'Ivoire / 15.2.1980 / G. Couturier leg.; Forêt dense / Humide / Sempervirente; Sous-bois / foret primaire; Biotope 7 / Bloc Saugbé (MNHN). • Cote d'Ivoire / 12.2.1980 / G. Couturier leg.; Forêt dense / Humide / Sempervirente; Sous-bois / foret primaire; Biotope nº 7 (MNHN). • [3x] Côte-d'Ivoire / 17.5.1979 / G. Couturier leg.; Défrichement; Champ de /riz; Biotope nº12 / bloc Cavally; Gouléako (MNHN). • [2x] Cote d'Ivoire / 25.4.1979 / G. Couturier leg.; Défrichement; Champ de /riz; Biotope nº12 / bloc Cavally; Gouléako (MNHN). • Olokemeji / Nigeria / Afr V-7-'36; VanZwal- / uwenburg / &McGough (USNM). • [11x] Olokemeji / Ibadan / Nigeria; Bridwell / collection (USNM). • MtCoffee / Liberia; Mes Sharp / Coll. 1891 (USNM). • MtCoffee / Liberia; 1896; GNCollins / Collector (USNM). • MtCoffee / Liberia; Mar. / 1897; RPCurrie / Collector (USNM). • Canboca / 30.II.03.; Collection / Rosenberg (USNM). • LIBERIA / Bendija / WMMann; Smithsonian / Firestone / Exp 1940 (USNM). • LIBERIA / GrandGedehCo. / 25KmN Zwedru / VII-21-1971 / J. A. Gruwell (USNM). • LIBERIA / GrandGedehCo. / 25KmN Zwedru / VII-10-1971 / J. A. Gruwell (USNM). • [2x] Coll. R. I. Sc. N. B. / Congo Belge / Bala / 28-III-1953 / P. Hostie (USNM).

8. *Phonoctonus luridus*

TYPE material: • TYPE; Brit.E.Africa / Uchweni Forest, / near Witu. / 25-27 Feb.1912. / S.A.Neave.; 1912-333.; Phonoctonus / luridus sp. n. / det. N.C.E. Miller. 1949 (NHMUK).

Material examined:

• Dr. Van Someren / Rabai / Feb 1930; Phonoctonus / sp. / det. N.C.E. Miller. 1949 (NHMUK). • Dr. Van Someren / Rabai / Jan. - Feb 1929.; Phonoctonus / sp. / det. N.C.E. Miller. 1949 (NHMUK). • van Someren / Rabai / August 1930; Phonoctonus / sp. / det. N.C.E. Miller. 1949 (NHMUK).

9. *Phonoctonus lutescens*

TYPE material: Neotype • Guinea Portoghese / Bolama / VI-XII.1899 L Fea; Museum Civ. / Genova (MNHN).

Material examined:

• Afr. occ / S. Thomé / Mocquerys; Phonoctonus / fasciatus P.B.; Phonoctonus / fasciatus; Nat. Hist. Mus / Budapest / coll. Hemiptera (HNHM) • Phonocotnus / lutescens G.P. / det. Dioli 1981; Guinea Port. / Suzana / VI-58 Andreoletti (MSNM). • [2x] Phonocotnus / lutescens G.P. / det. Dioli 1981; Guinea Port. / Bafatà / VI-54 Benassi (MSNM). • Bamako / Coleno leg 4/31; Cotonnier (MNHN). • Angola (MNHN). • Museum Paris / Sénégal / Dakar / G. Melou / 1905 (MNHN). • Museum Paris / Haut-Sénégal / Niger / Fabien Giraud / 1914 (MNHN). • [2x] Museum Paris / 1932 / Mission M. Griaula; Soudan fr. / Kila (MNHN). • Dahomey (Benin) (MNHN). • Fort Sibut / Haut-Chari Congo Français / Collection le Monlu; 4S2S; Collection / E. de Bergerin (MNHN). • Soudan / Malzy (1938) (MNHN). • Bamako / Soudan 1956 / P. Malzy rec. (MNHN). • Beladjia / 3-V-61; Museum Paris / Tchad (MNHN). • [4x] Nigeria: Jos / 1964 / E. Bot Gwang (MNHN). • [3x] Angola : Luanda, Belas

/ 1969 / Mme Giraudet; Muséum Paris / A. Villiers (MNHN). • [3x] GOLD COAST: / ACHIMOTA / IV.1954.G.STRIDE / B.M. 1954-227. (NHMUK). • NIGERIA / Ibadan / 1924-25 / J.D. Golding / Bred.; Pres by / Imp. Bur. Ent. / Brit. Mus. / 1926-118. / F3: ♀ (NHMUK). • NIGERIA / Ibadan / 1924-25 / J.D. Golding / Bred.; Pres by / Imp. Bur. Ent. / Brit. Mus. / 1926-118.; Spare / F2: ♂ (NHMUK). • NIGERIA / Ibadan / 1924-25 / J.D. Golding / Bred.; Pres by / Imp. Bur. Ent. / Brit. Mus. / 1926-118. Spare / F2: ♀ (NHMUK). • NIGERIA / Ibadan / 1924-25 / J.D. Golding / Bred.; Pres by / Imp. Bur. Ent. / Brit. Mus. / 1926-118.; P. ♀; Phonoctonus / lutescens / Det. B. Uvarov. G. et P. (NHMUK). • NIGERIA / Ibadan / 1924-25 / J.D. Golding / Bred.; Pres by / Imp. Bur. Ent. / Brit. Mus. / 1926-118.; Spare / F3 ♂ (NHMUK). • NIGERIA / Ibadan / 1924-25 / J.D. Golding / Bred.; Pres by / Imp. Bur. Ent. / Brit. Mus. / 1926-118.; F4: ♂ (NHMUK). • NIGERIA / Ibadan / 1924-25 / J.D. Golding / Bred.; Pres by / Imp. Bur. Ent. / Brit. Mus. / 1926-118.; F4: ♀ (NHMUK). • NIGERIA / Ibadan / 1924-25 / J.D. Golding / Bred.; Pres by / Imp. Bur. Ent. / Brit. Mus. / 1926-118.; F2: ♀ (NHMUK). • Sudan. 1967 (NHMUK). • N. Nigeria / J.J. Simpson; DEKIMA 13/I/II (NHMUK). • [2x] lutescens / G. & Pr; Distant Coll. / 1911-383 (NHMUK). • J6-55 / Angrla (NHMUK). • 1794 (NHMUK). • Congo (NHMUK). • Gawl; Saunders. / 65 · 13.; Harpactor. / fasciatus. / Walker's Catal. (NHMUK). • 40 / W3/ h01; Rhynocoris / lutescens Pereh / Guert Pereh. / Gendei Moll; Harpactor. / fasciatus. / Walker's Catal. (NHMUK). • H/1002/7; SAMARU Z. / N. Nigeria / 7-X-1955 / M.G.E. / Rama; Brit. Mus. 1966-336 (NHMUK). • H/1002/6; SAMARU Z. / N. Nigeria / 6-X-1955 / M.G.E. / Cotton / N.W.EXT.; Brit. Mus. 1966-336 (NHMUK). • [3x] Nigeria / Ibadan / 1924-25 / J.D. Colding / Brad.; Pres.by / Imp. Bur. Ent / Brit. Mus. / 1926-118.; Spare / F2 ♀ (NHMUK). • Nigeria / Ibadan / 1924-25 / J.D. Colding / Brad.; Pres.by / Imp. Bur. Ent / Brit. Mus. / 1926-118.; F1 ♂ (NHMUK). • [2x] Nigeria / Ibadan / 1924-25 / J.D. Colding / Brad.; Pres.by / Imp. Bur. Ent / Brit. Mus. / 1926-118.; F2 ♂ (NHMUK). • Nigeria / Ibadan / 1924-25 / J.D. Colding / Brad.; Pres.by / Imp. Bur. Ent / Brit. Mus. / 1926-118.; F3 ♂ (NHMUK). • Nigeria / Ibadan / 1924-25 / J.D. Colding / Brad.; Pres.by / Imp. Bur. Ent / Brit. Mus. / 1926-118.; F4 ♂ (NHMUK). • Nigeria / Ibadan / 1924-25 / J.D. Colding / Brad.; Pres.by / Imp. Bur. Ent / Brit. Mus. / 1926-118.; P ♂ (NHMUK). • [2x] Nigeria / Ibadan / 1924-25 / J.D. Colding / Brad.; Pres.by / Imp. Bur. Ent / Brit. Mus. / 1926-118.; Spare / F3 ♂ (NHMUK). • Nigeria / Ibadan / 1924-25 / J.D. Colding / Brad.; Pres.by / Imp. Bur. Ent / Brit. Mus. / 1926-118.; F4 ♀ (NHMUK). • Nigeria / Ibadan / 1924-25 / J.D. Colding / Brad.; Pres.by / Imp. Bur. Ent / Brit. Mus. / 1926-118.; F1 ♀ (NHMUK). • H/1002/8; Phonoctonus / lutescens / G+P; Brit. Mus. 1966-336 (NHMUK). • Nigeria. / A.W.J. Pomeroy / B.M. 1925-575. (NHMUK). • S.Nigeria. / Ibadan. / 25.IV.1923. / A.W.J. Pomeroy (NHMUK). • H/1002/3; Samar w.z. / N. Nigeria / 7-X-1953 / M.G.E. / Rama. prey Dysdercus (NHMUK). • H/1002/4; Samar w.z. / N. Nigeria / 7-X-1953 / M.G.E. / Rama; Brit. Mus. 1966-336 (NHMUK). • H/1002/5; Samar w.z. / N. Nigeria / 7-X-1953 / M.G.E. / Rama Prey / on Phoedonia; Brit. Mus. 1966-336 (NHMUK). • [2x] S.Nigeria. / Ibadan. / 25.IV.1923. / A.W.J. Pomeroy; Pres.by / Imp.Bur.Ent. / Brit. Mus. / 1923-459. (NHMUK). • NIGERIA: / Benue Prov. / Ibl. 1935. / R. Orme-Smith. / B.M. 1935-393. (NHMUK). • H/1002/9; ZARIA / B.C.G.A. / 4-II-56 / Waltheria / Americana; Brit. Mus. / 1966-336 (NHMUK). • SENEGAL / Sinthiou- / Maléme / 1943 / J.Risbec; Pres.by / Com.Inst.Ent. / B.M. 1948-154 (NHMUK). • H/1002/1; Phonoctonus / lutescens / G+P; 17-II-IVsk. / 25-II-Vsk / 12-12-Adult / fed on nymphs / of D. superstitiosus; KONTAGORA. N. / N.Nigeria / 17-II-1954 / M.G.E. / on cotton fed; Brit. Mus. / 1966-336 (NHMUK). • Central African Rep. / Ombella-Mpoko Pr. / 20 km NW Yaloke / 20.03.2010 400 m. / A. Kudrna Jr. Lgt. (NMPC). • East Africa / G. Kristensen; Coll. Jensen-Baarup; Phonoctonus / grandis? (ZMUC). • Ethiopia / Illubabor Prov. / Alwero River / 25 km W Abobo / 20.10.1987 / L.Rybalov leg.; on cotton; Phonoctonus / sp. (ZMMU). • Ethiopia / Illubabor Prov. / Alwero River / 25 km W Abobo / 20.10.1987 / L.Rybalov leg.; on cotton; Phonoctonus / sp. / Yu. Popov det. 2005

(ZMMU). • Coll. Mus. Tervuren / Cameroun: M’ Bakaou / VI. 1971 / J.L. Chemin (RMCA). • Coll. Mus. Congo / Kibali-Ituri: Mahagi-Port / 2-VIII-1953 / J. Hecq (RMCA). • [5x] Museé du Congo / Lac Albert : Ishwa / IX-1935 / H.J. Bredo (RMCA). • [4x] Museé du Congo / Lac Albert : Iswa / IX-1935 / H.J. Bredo (RMCA). • [2x] Museé du Congo / Congo da Lemba / IV à VI-1911 / R. Mayné (RMCA). • Museé du Congo / Congo da Lemba / 1910 / R. Mayné (RMCA). • Coll. Mus. Congo / Sénégal : M’Bambey / 1939 / J. Risbec (RMCA). • [3x] Coll. Mus. Congo / Libenge / 1938 / Leontovitch (RMCA). • Museé du Congo / Uele / 22-X-1933 / J. Vrydagh (RMCA). • Museé du Congo / Uele / 22-X-1933 / J. Vrydagh (RMCA). • [14x] Coll. Mus. Tervuren / Nigeria: Jos / 1964 / E. Bot Gwong; D / R. Det. / 7768 (RMCA). • Coll. Mus. Congo / Kibali-Ituri: Mahagi-Port / 2-VIII-1953 / J. Hecq (RMCA). • [7x] Coll. Mus. Tervuren / Angola: Luanda, Belas / 1969 / Mme Giraudet (RMCA). • Coll. R. I. Sc. N. B. / Congo Belge / Bili / 14-IV-1954 / Ch. Verbeke (USNM). • Coll. R. I. Sc. N. B. / Congo Belge / Bili / 14-IV-1954 / Ch. Verbeke (USNM).

10. *Phonoctonus nigrofasciatus*

TYPE material: • Caffra- / ria.; J. Wahlb.; nigro- fasciatus Stål; Typus; NHRS-GULI / 000000557 (NHRS). • HOLOTYPUS; Museé du Congo / Beni à Lesse / fin VII 1911 / Dr. Murtula; R. Dét. / A / 2581 (RMCA). [var. poultoni] • PARATYPUS; Museé du Congo / Beni à Lesse / fin VII 1911 / Dr. Murtula; R. Dét. / A / 2581 (RMCA). [var. poultoni] • [2x] PARATYPUS; Museé / du Congo Belge / Beni / Lt. Borgerhoff; R. Dét. / A / 2581 (RMCA). [var. poultoni]

Material examined:

• Kenya, Malindi / Gedi Forest / May 1973 / Hans Gønget leg. (ZMUC). • Phonocotnus / fasciatus Beauv. / det. Dioli 1981; Kiwu / IV 1951 / D. Calcagno (MSNM). • [2x♂] Mawambi-Beni / Grauer, 1910 (NHMW). • [2x♂, 1x♀] Urwald-Beni / Sept. Okt. 10. Grauer (NHMW). • Gabon / Coll. Signoret.; fasciatus / det. A. [unreadable] / det. Signoret (NHMW). • [♀] Uganda W.SW / of Hoima, Rwera / env., 30.11.2001 / leg. M. Snizek (NHMW). • [♂] Uganda C / Mubende env. / 19..22.11.2001 / leg. M. Snizek (NHMW). • Bwamba / W. Uganda / Feb. Mar. 1957 / R. Carcasson; Phonoctonus / fasciatus / P.B.; Kenya Natl. / Mus. exchange (USNM). • van Someren / Bwamba Valley / W. Uganda. May 1954; V.G.L. van Someren / Collection. / Brit. Mus. 1959-463. (NHMUK). • Uganda. / C.C. Gowdey. / 1912-401.; Entebbe / 18.VIII.11 / No. 3000; Phonocotnus / nigrofasciatus Stål (NHMUK). • Uganda / C. C. Godwey. / 1912-401.; Entebbe / 18.VIII.II. / No. 3000; Phonoctonus / nigrofasciatus Stål (NHMUK). • Entebbe, / Uganda. / 20-25 April, 1914. / C. C. Gowdey.; IMPERIAL BUREAU DF ENTOMOLOGY.; 1916-40 (NHMUK). • Tanganyika: / W. Shore of.L.Manyara. / Feb.-May.1935. / B. Cooper. / B.M. 1935-418 (NHMUK). • B.E.Africa: / Lake Victoria, Is. / Kyagwe coast / Manga Bay / 3.II.1919 / Dr. G.D.H. Carpenter. / 1920-201; Pres.by / Imp.Bur.Ent. / 823.ERB (NHMUK). • Uganda / Mpumu / Miss M. Robertson; Pres.by / Imp.Bur.Ent. / Brit. Mus. / 1927-85.; Note resemblanse / to [unreadable] cotton bug (NHMUK). • Nyasaland. / Dr. J.E.S. Old. / 1913-167. (NHMUK). • Marvern, / Natal. / 5.97 (NHMUK). • Marvern / Natal / 7.97 (NHMUK). • BWASYBA / 7.45; Imp.Bur.Ent. / Coll. No 10720; Brit. Mus. / 1965-274 (NHMUK). • Uganda / Kigazi Lake / Saturama plaine / II-1928 / G.D.H. Carpenter (NHMUK). • Uganda / Dwoli / 21.IV-1927. / H. Hargreaves (NHMUK). • Uganda / L. Edward. / 1931. / Dr. E. B. Worthington.; Cambridge Univ. Exp. / B.M. 1931-545. (NHMUK). • Mabira Forest, / Uganda. / Sep. 27 1913. / C.C.Godwey.; Pres.by / Imp.Bur.Ent. / Brit. Mus. / 1927-78. (NHMUK). • van Someren / Bwamba Valley / W. Uganda.

May 1954; V.G.L. van Someren / Collection. / Brit. Mus. 1959-468. (NHMUK). • [2x] GOLD COAST / ACHIMOTA / Dec. 1952 / G. Stride / B. M. 1953-25 (NHMUK). • [2x] Uganda Prot. / Semliki Plains, / near S. shore of / L. Albert. 2,200 ft.; 25-27 Nov. 1911. / S. A. Neave.; 1912-193 (NHMUK). • Entebbe, / Uganda. / 5 May 1913. / C. C. Gowdey.; Pres. by / Imp. Bur. Ent. / Brit. Mus. / 1927-78. (NHMUK). • Entebbe, / Uganda / Dr. C. A. Wiggins.; 1913-171. (NHMUK). • Entebbe. / Uganda. / Aug. 1912. / C. C. Gowdey. (NHMUK). • July 16-25.1911 / S. A. Neave.; Uganda Prot. / Mabira Forest, / Chagwe. / 3,500-3,800 ft.; Pres. by / Imp. Bur. Ent. / Brit. Mus. / 1927-85. (NHMUK). • Entebbe, / Uganda. / 3.4.13. / C. C. Gowdey. (NHMUK). • Uganda Prot. / Budongo Forest, / Unyoro. 3,400 ft. / 11-15 Dec. 1911. / S. A. Neave. (NHMUK). • Entebbe, / Uganda. / Sep. 1912 / C. A. Wiggins. (NHMUK). • NIGERIA / Ibadan / 1924-25. / J. D. Golding / Bred.; Spare / F4. ♂ (NHMUK). • Entebbe. / Uganda. / Sep. 1912. / C. C. Gowdey. (NHMUK). • Katonga Rv., / Uganda / Nov. 4-6, 1913. / C. C. Gowdey. (NHMUK). • UGANDA. / KAMPALA, / 10-19.XI.1917 / C. C. Gowdey.; Pres. by / Imp. Bur. Ent.; 1918-43 (NHMUK). • van Someren / Budongo Forest. / Bunyoro Uganda / Apl-May 1954; V.G.L. van Someren / Collection / Brit. Mus. 1959-468. (NHMUK). • Entebbe, / Uganda. / Dr. C.A. Wiggins.; 1913-171. (NHMUK). • Entebbe, / Uganda. / 24-25 May 1913; C.C. Godwey; 1913-350. (NHMUK). • Tero / 14.IV.11 / No. 2278; Phonoctonus sp. (NHMUK). • Kampala / Uganda / 10.I.1917 / No. 3000; Phonoctonus / not in BM (NHMUK). • van Someren / pre… [unreadable] / at. 44; Com. Inst. Ent. / Coll. No 9795; Brit. Mus. 1965-274 (NHMUK). • Ruwenzori: Mutwanga / (1000 – 1300 m) II / III-37 / Hackars / 2 / Parc Nat. Albert; R.I.Sc.N.B / I.G. 24.054; A. Villers det., 1952 / Phonoctonus / fasciatus var. / poultoni Schout. (RBINS). • Ruwenzori: Mutwanga / 1000 – 1300 m XI-36 / II-37 / Hackars / 2 / Parc Nat. Albert; A. Villers det., 1952 / Phonoctonus / fasciatus var. / poultoni Schout. (RBINS). • Rutshuru / 12.20 - VI-1934/ G.F. de Witte / 436 / Parc Nat. Albert; H. Schouteden det., 1945: / PHONOCTONUS / poultoni Scht; R. Mus. Hist. Nat. / Belg. I.G. 14.937 (RBINS). • Rutshuru / 12.20 - VI-1934/ G.F. de Witte / 436 / Parc Nat. Albert; H. Schouteden det., 1945: / PHONOCTONUS / poultoni Scht; R.I.Sc.N.B. / I.G. 24.054 (RBINS). • Rutshuru (1285) / 23-30-X-1934 / G.F. de Witte / 715. / Parc Nat. Albert; H. Schouteden det., 1945: / PHONOCTONUS / poultoni Scht; Phonoctonus / poultoni Schout. / det. Schouteden; R. Mus. Hist. Nat. / Belg. I.G. 14.937 (RBINS). • Rutshuru (1285) / 23-30-X-1934 / G.F. de Witte / 713. / Parc Nat. Albert; H. Schouteden det., 1945: / PHONOCTONUS / poultoni Scht; Phonoctonus / poultoni Schout. / det. Schouteden; R. Mus. Hist. Nat. / Belg. I.G. 14.937 (RBINS). • Plaine Semliki / (900-1100 m) IV / X-37 / Hackars / Parc Nat. Albert; R.I.Sc.N.B. / I.G. 24.054; A. Villiers det., 1952: / Phonoctonus/ fasciatus Pal de Beauv. (RBINS). • Congo Belge: P.N.A. / Mutsora / 1939 / Hackars; A. Villiers det., 1952: / Phonoctonus / fasciatus Pal. de Beauv.; R.I.Sc.N.B. / I.G. 24.054 (RBINS). • Coll. Mus. Congo / N. Lac Kivu: Rwankwi / VII-1951 / J.V. Leroy (RMCA). • Museé du Congo / Semliki (Kivu) / X-1936 / (Lisfrane) (RMCA). • Coll. Mus. Congo / N. Lac Kivu: Rwankwi / VI-1951 / J.V. Leroy (RMCA). • [3x] Plaine Semliki / (900-1100 m) IV/X-37 / Hackars / Parc Nat. Albert; Coll. Mus. Tervuren; A. Villiers det., 1952: / Phonoctonus / faciatus Pal. de. Beauv. (RMCA). • Coll. Mus. Congo / Rwanda: contref. Est / Muhavura 2100 m. / P. Basilewsky 28-I-53 (RMCA). • Congo Belge: P.N.A. / 11-VIII-1957 / P. Vanschuytbroeck / VS-114; Massif Ruwenzori / Kahonge, 1800 m / riv. Babalwakitaka / affl. Dr. Butahu; Coll. Mus. Tervuren; Phonoctonus / fasciatus / Pal. Beauv. / A. Villiers det 195 (RMCA). • Museé du Congo / B.E.A.: Campi Simba / 317-VI-1913 / Dr. Bayer; R. Dét. / C / 2581 (RMCA). • Museé du Congo / Kivu: Rutshuru / 22-V/1-VI-1934 / G.F. de Witte / Parc Nat. Albert (RMCA). • Coll. Mus. Congo / Victoria Nyanza: / Ukerewe III-1938 / R.P. Conrads (RMCA). • Congo Belge: P.N.A. / Ondo / 1200 m 29-vii-1935 / Mission H. Damas: (a) 283; Coll. Mus. Tervuren (RMCA). • Museé du Congo / Région des Lacs / Dr. Sagona; R. Dét. / B / 2581 (RMCA). • Museé du Congo / Kibga (S. Bishoke) / (2400) 16-18-19-II-35 / G.F.

de Witte / Parc Nat. Albert (RMCA). • Museé du Congo / Rutshuru (1285) / 1/6-VI-1934 / G.F. de Witte / Parc Nat. Albert; 426; Phonoctonus Poultoni Schout. (RMCA). • Museé du Congo / Rutshuru (1285) / 1/6-VI-1934 / G.F. de Witte / Parc Nat. Albert; 427; Phonoctonus Poultoni Schout. (RMCA). • Museé du Congo / Kivu: Rutshuru / 22-V 11-VI-1934 / G.F. de Witte / Parc Nat. Albert; 417 (RMCA). • Congo Belge: P.N.A. / 14-VIII-1955 / P. Vanschnytbroeck / 13. 341-44; Mont Hoyo / Piste Matupi / 1.200 m; Coll. Mus. Tervuren; Phonoctonus / poultoni / Schout. / A. Villiers det 195... (RMCA). • Coll. Mus. Congo / W. Ruwenzori: Mut- / wanga -1932 / Dr. Van Hoof (RMCA). • Coll. Mus. Congo / N. Lac Kivu: Rwankiwi / XI-1951 / J.V. Leroy (RMCA). • Coll. Mus. Congo / N. Lac Kivu: Rwankiwi / IX-1951 / J.V. Leroy (RMCA). • Ruwenzori: Mutwanga / (1000-1300 m) II/III-37 / 2 / Hackars / Parc Nat. Albert; Coll. Mus. Tervuren; A. Villiers det. 1952: / Phonoctonus / fasciatus var. / poultoni Schout. (RMCA). • W. Ruwenzori / (1200-1500 m) –III-37 / 11. / Hackars / Parc Nat. Albert; Coll. Mus. Tervuren; A. Villiers det. 1952: / Phonoctonus / fasciatus var. / poultoni Schout. (RMCA). • W. Ruwenzori / (3000-4000 m) –III-37 / 10 / Hackars / Parc Nat. Albert; Coll. Mus. Tervuren; P. fasciatus / var. poultoni Schout. / A. Villiers det. 1951 (RMCA). • Congo belge P.N.A. / 9-VII-1954 / P. Vanschuytbroeck & / H. Synave 9194; Secteur Nord / Mulingo, 1.350 m / Secteur Kikura / Région Baniangala (RMCA). • [2x] Coll. Mus. Tervuren / Erytracea / Asmara / Coll. H. Schouteden (RMCA). • Ruwenzori: Mutwanga / 1000-1300 m XI-36/II-37 / Hackars / Parc Nat. Albert; Museum Paris / A. Villiers (MNHN). • Victoria Nyanza / Arcip. di SESSE / Bugala…. 1908 / Dr. E. Bayon; Museum Paris (MNHN). • Congo Belge: P.N.A. / Mutsora / 1939 / Hackars; Museum Paris / A. Villiers (MNHN). • Mars 1960 / Bururi – Urundi / Congo Belge / R.P. Giraudin; Museum Paris (MNHN).

11. *Phonoctonus picta*

TYPE material: • HOLOTYPUS / Museé du Congo / Bandoinville / fin XI-1918 / R. Mayné; R. Dét. / E / 2581 (RMCA). • PARATYPUS / Museé du Congo / Bandoinville / fin XI-1918 / R. Mayné; R. Dét. / E / 2581 (RMCA).

Material examined:

• [2x] Museé du Congo / Tanganyka: Lusaka / -1936 / R.P. Debbaudt (RMCA). • Musée du Congo / Katanga: Kibombo / -VI-1930 / Ch. Seydal / R. 4319; Kibombo / Juin 1930 / Ch. Seydal; R. 4319 (RMCA). • Museé du Congo / Bandouinville / 16-I-1933 / L. Burgeon (RMCA). • Coll. Mus. Congo / Maniema: Kasongo / IX-1936 / R. Henrard; Récolté sur Cotonnier (RMCA). • Coll. Mus. Congo / Kibali - Ituri: ~~Kikanga~~ / Kinawa 1-II-1940 / A. Lepersonne (RMCA). • Coll. Mus. Congo / Yakoma / -IX-1939 / Leontovitch (RMCA). • Coll. Mus. Congo / Ubangi: Musa / XI-1941 / J. Vrydagh / V.H. 153; Voir Cocl. / en alcool; Musa / Ubangi / XI-41 / S / Légumineuse / VH. 153 / J. VRYDAGH (RMCA). • Coll. Mus. Tervuren / Maniema: Kasongo / VIII/IX.1959 / P.L.G. Benoit; Phonoctonus / fasciatus / bifasciatus Vill. / A. Villiers det 1967 (RMCA). • Coll. Mus. Tervuren / Rég. Du Haut Uele / Niangara 1952 / Cotonco; Phonoctonus / fasciatus / bifasciatus Vill. / A. Villiers det 1967 (RMCA). • Coll. Mus. Congo / Station de Gandanijka / I.N.E.A.C. 1800 1957 / P. de Francquen (RMCA). • Museé du Congo / Kasongo / -IX-1936 / P. Henrard; S/ Cotonnier (RMCA). • [9x] Museé du Congo / Maniema / 1936 / P. Henrard (RMCA). • [2x] Coll. R. I. Sc. N. B. / Congo Belge / Sohe / 10-V-1958 / L. Henry (USNM). • Coll. R. I. Sc. N. B. / Congo Belge / Kibangula / V-1956 / L. Henry (USNM). • Congo Belge / Kibangula / II-1955 / L. Henry; R. I. Sc. N. B. / I. G. 20.520

(USNM). • [2x] Congo Belge / Nyunzu / Kabeya-Mulonga / 1955-L. Henry; R. I. Sc. N. B. / I. G. 20.520 (USNM). • Entebbe / Uganda / May 1952 / E. Finbey; Kenya Natl. / Mus. exchange (USNM).

12. *Phonoctonus picturatus*

TYPE material: • 2401 / 83; Nimocoris / picturatus / n.sp. / Phonoctonus / picturatus / E03.; HOLOTYPE (MNHN). • HOLOTYPUS; Museé du Congo / Mayumbe: Buku / Jembe -10-X-1924 / A. Collart; R. Dét. / F' / 2581; Phonoctonus / fasciatus var. / discalis Scht. (RMCA). • [2x] PARATYPUS; Museé du Congo / Congo da Lemba / ~~V~~ -1912 / R. Mayné; R. Dét. / F' / 2581 (RMCA). • PARATYPUS; Museé du Congo / Mayumbe: Tshela / 19-27-II-1916 / R. Mayné; R. Dét. / F' / 2581 (RMCA). • [2x] PARATYPUS; Museé du Congo / Luali / 29-VIII-1913 / Dr. Bequaert; R. Mayné; R. Dét. / F' / 2581 (RMCA). • [4x] PARATYPUS; Museé du Congo / Mayumbe 24-XI-15 / Makaia N'Tete / R. Mayné; R. Dét. / F' / 2581 (RMCA). • [2x] PARATYPUS; Museé du Congo / Kiniati - Zobe / fin XII-1915 / R. Mayné; R. Dét. / F' / 2581 (RMCA). • PARATYPUS; Museé du Congo / Mayumbe / Tsehobo / de Briey; R. Dét. / F' / 2581 (RMCA). • PARATYPUS; Museé du Congo / Mayumbe: Zobe / 4an 12-I-1916 / R. Mayné; R. Dét. / F' / 2581 (RMCA).

Material examined:

• Africa / Gabun; Phonoctonus / picturatus; Hung. Nat. Hist. Mus / Budapest / coll. Hemiptera (HNHM). • Africa / Gabun; Phonoctonus / picturatus Fairm; Phonoctonus / picturatus; Hung. Nat. Hist. Mus / Budapest / coll. Hemiptera (HNHM). • [2x] Gabon / Coll. Signoret.; picturat. / det. Signoret (NHMW). • ANGOLA (A26) / Salazar, I.I.A.A. / 9-15.III.1972; Southeren / African Exp / B.M. 1972-1 (NHMUK). • picturatus / Fairm.; Camerun; Distant Coll. / 1911-383 (NHMUK). • Congo belge / Mayumbe / vi-1936; R. Mus. Hist. Nat. / Belg. I.G. 12.627; Phonoctonus / fasciatus / var. discalis / Schout / A. Villieus det 1969 (RBINS). • Congo belge: / District du Bas-Congo / Mayumbe: vi-1936; R. Mus. Hist. Nat. / Belg. I.G. 11.680 (RBINS). • Gabon; 3844.; R.I.Sc.N.B. / I.G. (RBINS). • Kassongo / Á Stanleyfalls / Rom (RBINS). • [2x] Chutes de Samlla / Riv. N. Gambie / Mocquerys (1 ma II 93.) (RBINS). • [3x] Museum Paris / Congo Français / Rég. du Haut - Ivindo (Affl. Ogooné) / (Dr J. Gravot) 1906 / Cap[e] Cottes (MNHN). • Museum Paris / Congo Franç. / Env de Brazzaville / M'Bamou / Montezer 1903 (MNHN). • (Sibang) Gabon / 28 Sept. 1958 ♂ / G. Minet. leg. (MNHN). • Libreville ♀ / GABON / 28 Sept. 1958 / G. Minet Leg.; Museum Paris / Coll. / J.P. Orvoen; Museum Paris (MNHN). • Brazzaville / Congo / 10-1963; Museum Paris / Mission / A. Descarpentries / et A. Villiers / 1963-1964 (MNHN). • Museum Paris / Congo Franc. / Env. de Brazzaville / E. Rouband et. A. Weiss / 1907 (MNHN). • Museum Paris / Landana Congo / P. Klein 1875 (MNHN). • [4x] Museum Paris / BAS-OGOOUÉ / Entre Lambaréné / et La Mer / E. Haug 1901 (MNHN). • Gabon [Lethierry] (MNHN). • Dimonika / (Mayumbe) / Congo / I-1964; Museum Paris / Mission / A. Descarpentries / et A. Villiers / 1963-1964; Museum Paris (MNHN). • [2x] Sibiti / Congo / XI-1963; Museum Paris / Mission / A. Descarpentries / et A. Villiers / 1963-1964; Museum Paris (MNHN). • Brazzaville / Congo / 10-1963; Museum Paris (MNHN). • Sibang Gabon / 20 Sept. 1959 ♂ / G. Mineb leg.; Museum Paris / Coll. / J. P. Orvoen (MNHN). • [5x] Libreville Gabon / 19 Oct. 1958 ♀/ G. Minet Legit. (MNHN). • Sibang Gabon / 20 september 1959 / G. Minet leg ♀; Muséum Paris (MNHN). • Rep. Pop. Congo / Dimonika, Makaba / 25.II.1978 / J.-J. Menier rec. (MNHN). • Gabon (MNHN). • [4x] Museum Paris / Gabon / Coll. Noualhier 1898 (MNHN). • Haut Oggoné / Lambaréné (MNHN). • Sibang (Gabon) / 11 NOV 1958 ♂ / G. Minet leg.; Museum Paris /

Coll. / J.P. Orvoen (MNHN). • Gabon; Coll. Mus. Tervuren / Coll. H. Schouteden (RMCA). • Museé du Congo / Congo francais / Lambarene / Coll. Schouteden; R. Dét. / F' / 2581 (RMCA). • Coll. Mus. Congo / Kunungu -1938 / (Nkele) / (Coll. Schouteden) (RMCA). • Museé du Congo / Mayumbe: Zobe / 4an 12-I-1916 / R. Mayné (RMCA). • Coll. Mus. Congo / Bas. Congo: / XI.1954 / (L. Hassewer). • Coll. Mus. Congo / Bokalakala (Bolobo) / 1954 / R.C. Eloy (RMCA). • Gabon; 197.; Phonoctonus / picturatus Frm.; Mus. Zool. Helsinki / Loan No. / HE 2013-31 (MZH).

13. ***Phonoctonus principalis***

TYPE material: • 169. / Quilimane / 9.II.89. / Coll. Stuhlmann; A. Gerstäcker / determ. 1891.; Phonoctonus / principalis / Gerst. (ZMUH).

• Quilimane; Phonoctonus / principalis Gerst; Phonoctonus / validus Horv.; Phonocotnus / validus; Hung. Nat. Hist. Mus / Budapest / coll. Hemiptera (HNHM).

Material examined:

• Zanzibar.; Schmeltz.; NHRS-GULI / 000006449 (NHRS). • Mombo / Sjöstedt; Usambara; juni; NHRS-GULI / 000006452 (NHRS). • Zanzibar; Schmeltz.; NHRS-GULI / 000006450 (NHRS). • Bagamoyo.; NHRS-GULI / 000006453 (NHRS). • Bagamoyo.; NHRS-GULI / 000006454 (NHRS). • Bagamoyo.; NHRS-GULI / 000006455 (NHRS). • Lindi / Ost. Afrika.; NHRS-GULI / 000006451 (NHRS). • S. Rhodesia: / Odzi dist / 26.V.48 / N.C.E. Miller.; 165.; Phonoctonus / nigrofasciatus / Stål; N.C.E. Miller det. 1958; Phonoctonus / nigrofasciatus; Nat. Hist. Mus / Budapest / coll. Hemiptera (HNHM). • S. Rhodesia: / Odzi dist / 26.V.48 / N.C.E. Miller.; 165.; Phonoctonus / nigrofasciatus / Stål; N.C.E. Miller det. 1958; Phonoctonus / nigrofasciatus; Nat. Hist. Mus / Budapest / coll. Hemiptera (HNHM). • Africa / Bagomoyo; Phonocotnus / validus; Hung. Nat. Hist. Mus / Budapest / coll. Hemiptera (HNHM). • Dtsch. O. / Afrika; Phonoctonus / fasciatus P.B. / var. immitis Stål; Phonoctonus / fasciatus v. / immitis; Nat. Hist. Mus / Budapest / coll. Hemiptera (HNHM). • Africa / Bagomoyo; Phonoctonus / fasciatus v. / immitis; Nat. Hist. Mus / Budapest / coll. Hemiptera (HNHM). • Phonctonus / principalis / Gerst. / A. Villiers det. 1962 (NHMW). • D.O. Afrika / Dar es Salam, / H. Mayer, 96. (NHMW). • [♂] Lindi / D.O. Afr. / Ertl. (NHMW). • [♂] Hanser. 96 / [IRnta.; unreadable] Ost. afr. (NHMW). • [♀] Pugu, Hinterld. / v. Daressalaam, / D.O.Afr. Ertl (NHMW). • [1x♂, 1x♀] D.O. Afrika / [Morogoro] / Nachl. Schmitt (NHMW). • [2x♀] Pachinger / D.-OstAfrika (NHMW). • [♀] Lukuledi, / D.O. Afr. Ertl. (NHMW). • [♀] Daressalaam, / D.O. Afr. Ertl (NHMW). • [♂] West Usambara / D.O. Afr. Ertl (NHMW). • [♀] Ost. Afrika, / Dar es Salam, / H. Mayer, 96. (NHMW). • [3x♂, 1x♀] D.O. Afrika, / Dar es Salam, / H. Mayer, 96. (NHMW). • [♀] D.O. Afrika, / Dares Salam, / H. Mayer, 96. (NHMW). • [♂] Ost Afrika / Neûhuet 88 (NHMW). • [♀] Zanzibarküste / Steind. D. 1888 (NHMW). • Tanganyika- / Terr. Tanga / 14.XI.'35 Zerny (NHMW). • [2x] On Mgoza / Mar 17th 1958; Nyasaland / Lower Shire Valley / R.C.H. Sweeney (NHMUK). • On Mgoza / Mar 10th 1958; Nyasaland / Lower Shire Valley / R.C.H. Sweeney (NHMUK). • On Mgoza / Mar 8th 1958; Nyasaland / Lower Shire Valley / R.C.H. Sweeney (NHMUK). • On Mgoza / 18th Mar 1958; Nyasaland / Lower Shire Valley / R.C.H. Sweeney (NHMUK). • On Mgoza / 12th Feb 1958; Nyasaland / Lower Shire Valley / R.C.H. Sweeney (NHMUK). • [4x] On Mgoza / 13th-Feb 1958; Nyasaland / Lower Shire Valley / R.C.H. Sweeney (NHMUK). • On Mgoza / 13th-Feb 1958; Nyasaland / Lower Shire Valley / R.C.H. Sweeney; Presby / Com Inst Ent / BM 1958-301 (NHMUK). • Nairobi Museum / Mombasa 3.21 / van Someren; Phonoctonus / sp. / det. N.C.E.Miller.1949.

(NHMUK). • [6x] S.Rhodesia: / Odzi dist / 28.V.48 / N.C.E.Miller.; 165 (NHMUK). • Nyasaland. / E. Ballard. / 1913-490.; Reduviidae / Phonoctonus / nigrofasciatus / Stål (NHMUK). • [4x] Chiromo / Mgoza, Jan 1958; Nyasaland / Lower Shire Valley / R.C.H. Sweeney (NHMUK). • Chiromo / Mgoza, Dec 1958; Nyasaland / Lower Shire Valley / R.C.H. Sweeney (NHMUK). • Brit. A. Af. / Kibwezi . 3,000 ft. / Apl. 2-4.1911. / S.A. Neave; 1917-177 (NHMUK). • N. Rhodesia / Feira. / 12.7.11 / F.V.Bruce Miller. / 1913-204. (NHMUK). • Brit. E. Africa. / L. Mpeketoni, / near Kipini. / 4-5 March, 1912. / S.A. Neave. (NHMUK). • Fort Johnston / Nyasaland / (P. Rendal); Distant Coll. / 1911-383. (NHMUK). • Tanganyika T. / Tanga / VI. 1932; Pres. by / Com. Inst. Ent. / B. M. 1948-548; Mrs I. Ogilvie (NHMUK). • Nyasaland. / Chiromo. / R. C. Wood; Phonoctonus / nigrofasciatus / Det. B. Uvarov. St. (NHMUK). • Mozambique. / 98-136. (NHMUK). • Brit. E. Africa. / Mombasa. / 12-13 Feb. 1912. / S.A. Neave; 1912-333. (NHMUK). • Nyasaland. / E. Ballard / 1913-490. (NHMUK). • GOLD COAST / ABURI / 1912-13 / W.H. PATTERSON; Pres by / Com. Inst. Ent. / B.M. 1950-96 (NHMUK). • W. Afrika. / Gambia; Pres by / Imp. Bur. Ent. / Brit. Mus. / 1925-94 (NHMUK). • Kenya: / Mombasa / K. G. Preston- / Mafham / B.M. 1984-440 (NHMUK). • E. Africa / 92-23. (NHMUK). • At light; Tanganyika: / Tanga Prov. / IV-V 1950. / R.C.H. Sweeney. / B.M. 1950-493. (NHMUK). • Tanganyika / Kilosa / 17.II.65 / E.S. Brown / 941; Brit. Mus. / 1982-145 (NHMUK). • Nyasaland / E. Ballard. / 1913-490. (NHMUK). • Tanganyika / Tinde / 29.VIII.41; 29.8.41 / under Baobab / Tru / Tinde; Coll. Inst. Ent. / Coll. No. 11599; Pres by / Com. Inst. Ent. B.M. 1950-262; E.D. Burtt. / B.M. 1948-39.; Tanganyika / Dr. E. Burtt; Phonoctonus / nigrofasciatus Stål / … / det. N.C.E. Miller 1950 (NHMUK). • Nyasaland. / Chiromo. / R.C. Wood; Pres. by / Imp. Inst. Ent. / B.M. 1936-27. (NHMUK). • Nyasaland. / Mlanje. / 5 Feb. 1914 / S.A. Neave.; Imperial / Bureau of / Entomology; 1916-40 (NHMUK). • Nyasaland / Port Herald. / Apl. – June, 1913. / Dr. J.E.S. Old.; 1913-560 (NHMUK). • Nyasaland / Dr. J.E.S. Old. / 1913-167. (NHMUK). • [2x] Van Someren / Rabai / August 1930; V.G.L. van Someren / Collection. / Brit. Mus. 1959-469. (NHMUK). • [2x] Mombasa 3.21 / van Someren; V.G.L. van Someren / Collection. / Brit. Mus. (NHMUK). • [2x] van Someren / KILIFI 6.46; V.G.L. van Someren / Collection. / Brit. Mus. (NHMUK). • Port. E. Africa / Anturabe / 4.VIII. 1918 / G.D.H. Carpenter. 1919_36 (NHMUK). • San Pedro, / French Ivory Coast. / Guy Chetwynd. / 1915_306. (NHMUK). • van Someren / Taveta For: / Kenya 5 47. (S); V.G.L. van Someren / Collection. / Brit. Mus. (NHMUK). • van Someren / Rabai 8-37.; V.G.L. van Someren / Collection. / Brit. Mus. (NHMUK). • Dr. van Someren / Rabai / Jan-Feb. 1929.; V.G.L. van Someren / Collection. / Brit. Mus. (NHMUK). • S. Rhodesia: / Odzi dist. / 28.V.48 / N.C.E. Miller.; 165 (NHMUK). • ON MGOZA / Mar 17 1958; Nyasaland / Lower Shire Valley / R.C.H. Sweeney (NHMUK). • [3x] Nyasaland. / Dr. J. E. S. Old. / 1913-167. (NHMUK). • Brit. E. Africa. / Mombasa. / 12-13 Feb. 1912. / S. A. Neave.; 1912-36 (NHMUK). • N. W. Shore of L. Nyasa. / fm. Florence Bay to Karonga. / 30 June 6 July 1910. 1,650 ft. / S. A. Neave. (NHMUK). • Nyasaland. / Mlanje. / July 12, 1913. / S. A. Neave.; 1914-156.; Phonoctonus / principalis, Gerst. (NHMUK). • E. Africa. / 92-23. (NHMUK). • Zanzibar. / Dr. W. M. Aders. / 1913-206.; Hem 32; Z’bai. / Beit el Ras / 8.11.12 / On maize (NHMUK). • Germ. E. Africa. / Lulanguru. / Nov 1917. / G.D.H. Carpenter. / 1918-56; Pres. by / Imp. Bur. Ent.; principalis / (err. as lutescens) (NHMUK). • [11x] S.RODESIA: / Odzi dist. / 28.V.48 / N.C.E. Miller; 165 (NHMUK). • [2x] S.RODESIA: / Odzi dist. / 11.VI.48 / N.C.E. Miller; 165 (NHMUK). • van Someren / Kilifi (J.) / Kenya x48; V.G.L. van Someren / Collection. / Brit. Mus. 1959-468. (NHMUK). • Mombasa 3.21 / van Someren; V.G.L. van Someren / Collection. / Brit. Mus. 1959-468. (NHMUK). • van Someren / Rabai / August 1930; V.G.L. van Someren / Collection. / Brit. Mus. 1959-468. (NHMUK). • van Someren / Rabai / August 1930; Phonoctonus / nigrofasciatus / Dist. / Det. B. Uvarov 1931 (NHMUK). • van Someren / Taveta For: / Kenya 8 47. (S); V.G.L. van Someren / Collection. / Brit. Mus. 1959-468. (NHMUK). • ON

MGOZA / MAR 17 1958; Nyasaland / Lower Shire Valley / R.C.H. Sweeney (NHMUK). • Chiromo / Mgoza, Jan 1958; Nyasaland / Lower Shire Valley / R.C.H. Sweeney (NHMUK). • Nyasaland. / Ruo Valley. 1,000-2,000 ft / 21-25 Apr.1910. / S.A.Neave.; Africa. / 1913-417. (NHMUK). • Tanganyika / Kiloza / 17.II.65 / E.S. Brown / 941; Brit. Mus. / 1982-145 (NHMUK). • [2x] Chiromo / Mgoza, Dec. 1957; Nyasaland / Lower Shire Valley / R.C.H. Sweeney (NHMUK). • Tanganyika: / Lake Nyassa. 1,600 ft. / 34°00'E. 9°30'S. / 28.VIII.1959.; Cambridge E. African. / Exped / B.M. 1960-50. (NHMUK). • Nairobi, B.E.A. / June & July, 1912. / Dr. A. D. Milne. / 1913-192. (NHMUK). • Nyasaland. / S.W. of / Lake Chilwa. / 9.Jan.1914. / S.A.Neave.; Imperial / Bureau of / Entomology; 1916-40. (NHMUK). • Nyasaland. / S.W. of / Lake Chilwa. / 12.Jan.1914. / S.A.Neave.; Imperial / Bureau of / Entomology; 1916-40. (NHMUK). • Nyasaland / Port Herald. / Apr.-June, 1913. / Dr. J.E.S. Old.; 1913-560 (NHMUK). • Afrique australe / de Sétys Fauson; M.R. Belg.; 6751; Phonoctonus / principalis / Gerst. / A. Villiers det 1969 (RBINS). • Congo belge: P.N.U. / R. Bowa affl. dr. Kalule N. / près Kiamalwa; 13-III-1949 / Mis. G.F. de Witte. 2408a; R.I.Sc.N.B. / I.G. 24.054; A. Villiers det., 1945: / Phonoctonus / grandis Signoret. (RBINS). • Congo belge: P.N.U. / Mabwe (Lac Upemba) / (585 m.) 1-12.VIII-1947 / Mis. G.F. de Witte. 660a; A. Villiers det., 1945: / Phonoctonus / grandis Signoret; R.I.Sc.N.B. / I.G. 24.054 (RBINS). • [2x] Congo belge: P.N.U. / Mabwe (Lac Upemba) / (585 m.) 1-12.VIII-1947 / Mis. G.F. de Witte. 660a; A. Villiers det., 1945: / Phonoctonus / grandis Signoret (RBINS). • N.O. Afrika / Songoro Nofrela / 2.7.1901 / V. Brianger; 2.VII.01; R.I.Sc.N.B. / I.G. (RBINS). • Congo / Cammaert; R.I.Sc.N.B. / I.G. (RBINS). • Gandajika 1953 / de Francquen / E.B; Coll. R. Mayné / Comm. Et. Bois Congo / R. 2494; R.I.Sc.N.B. / I.G. 22.863 (RBINS). • Rhodésie du Nord / Mweru-Wantipa / 21-IV-1944 / H.-J. Brédo; R.I.Sc.N.B. / I.G. 15.333 (RBINS). • Ost-Afrika / Dar-es-Salaam / ex. Coll. Fruhstorfer; R.I.Sc.N.B. / I.G. (RBINS). • Daressalam. / Afrique orient. allemande. / Dr J. Carl.; Phonoctonus / principalis / Gerst. (MHNG). • [2x] Ost-Africa / Ingenieur Sonntag / ded.8.IX.1894. (ZMUH). • 170. / Quilimane / 9.II.89. / Coll. Stuhlmann; A. Gerstäcker / determ. 1891. (ZMUH). • 173. / Quilimane / 9.II.89. / Coll. Stuhlmann; A. Gerstäcker / determ. 1891. (ZMUH). • 231. / Mhonda / 6.IX.88. / Coll. Stuhlmann; A. Gerstäcker / determ. 1891. (ZMUH). • Phonoctonus / principalis / Gerst.; Mozambique / Lourenco Marques P.E.A / 1949/04/01; SAM-HEM- / A003643 (SAMC). • Phonoctonus / principalis / Gerst.; SAM-HEM- / A003644 (SAMC). • Afr. Or.; Coll. Mus. Tervuren / Coll. H. Schouteden (RMCA). • Dtsch O. / Africa; Coll. H. Schouteden (RMCA). • Museé du Congo / Afr. orien. ang. / Mombasa / Coll. Schouteden; Brit. Afriq / Mombasa; [unreadable]; R. Dét. / F / 2580 (RMCA). • Museé du Congo / D.O.Afr: / Luitpoldkette / Coll. Schouteden; D.O. Afrika: / Luitpoldkette; R. Dét. / F / 2580 (RMCA). • Museé du Congo / Afr. orient. ang. / Mombasa / Coll. Schouteden; Br. O. Afrika / Mombasa; R. Dét. / F / 2580 (RMCA). • Museé du Congo / Afr. orient. ang: / Mombasa / Coll. Schouteden; Brit. E. africa / Mombasa; R. Dét. / F / 2580 (RMCA). • Museé du Congo / Usambara / Coll. Schouteden; principalis; Usambara; Sjöstedt.; R. Dét. / F / 2580 (RMCA). • Coll. Mus. Congo / Kaniama / -1931 / R. Massart (RMCA). • NO. Tanganyjka / Ruanda 31.III.97 / Ramsay & Hosemann S.; Phonoctonus grandis Sign.; Coll. Mus. Tervuren / Coll. H. Schouteden (RMCA). • [2x] Congo belge P.N.A. / Mabwe (lac Upemba) / (585 m.) 1-12-VIII-1947 / Mis. G.F. de Witte. 660a; A. Villiers det., 1954 / Phonoctonus / grandis Signoret. (RMCA). • Museé du Congo / Katanga: Kasileshi / (R. Kameji) 19-V-1923 / F.G. Overlaet; R. Dét. / G / 2580 (RMCA). • Museé du Congo / BAUDOINVILLE / fin XI 1918 / R. Mayné; R. Dét. / G / 2580 (RMCA). • Museé du Congo / BAUDOINVILLE / fin XI-1918 / R. Mayné; R. Dét. / G / 2580 (RMCA). • [2x] Museé du Congo / Nioka / -1934 / P. Lefèvre (RMCA). • Museé du Congo / Niunzu / 20-30-V-1930 / (Girard); R. Dét. / H / 2580 (RMCA). • Museé du Congo / Lulua: Yandoa / X.1930 / G.F. Overlaet; R. Dét. / H / 2580 (RMCA). • Museé du Congo / VIII ~~Ruwenzeri~~ 1932 / Nyangwe / Mme L. Lebrun; R. Dét. / H / 2580

(RMCA). • Coll. Mus. Congo / Rég. Des Grands Lacs / Thys (RMCA). • [2x] Musée du Congo / Kafakumba / III-1932 / F.G. Overlaet; R. Dét. / H / 2580 (RMCA). • Coll. Mus. Congo / Kasai : Ipamu / -1922 / P. Vanderijst; R. Dét. / 2582 (RMCA). • Museé du Congo / Lomami : Kiabukwa / -III-V-1932 / P. Quarré (RMCA). • Museé du Congo / Kabassibare / 25-IX-1930 / J. Vrydagh / coton; R. Dét. / H / 2580 (RMCA). • Museé du Congo / Lulua: Muteba / VI-1932 / G.F. Overlaet; R. Dét. / H / 2580 (RMCA). • [2x] Museé du Congo / Lulua: Muteba / V-1932 / G.F. Overlaet (RMCA). • Museé du Congo / Katanga: Mwama- / Kusu. 4-VII-1930 / Ch. Seydel; R. Dét. / H / 2580 (RMCA). • Museé du Congo / Lomami : Lusuku / -XI-1930 / P. Quarré; R. Dét. / H / 2580 (RMCA). • Museé du Congo / Lulua: Kapanga / IX.1932 / F.G. Overlaet (RMCA). • [11x] Coll. Mus. Congo / Maniema: Kasongo. / IX-1936 / P. Henrard; Récolté sur / Cotonnier (RMCA). • Museé du Congo / Lulua: Muteba / VI-1932 / G.F. Overlaet (RMCA). • Museé du Congo / Lulua: Kapanga / X-1932 / G.F. Overlaet (RMCA). • Museé du Congo / Lulua: Kalenge / X-II-1934 / F.G. Overlaet (RMCA). • Museé du Congo / Ituri-Nioka / -1934 / Lefêvre (RMCA). • Museé du Congo / Lulua: Kapanga / IX-1932~~3~~ / F.G. Overlaet (RMCA). • Museé du Congo / Mulongo (Niunzu) / 20/30-V-1930 / Dr P. Gérard (RMCA). • Museé du Congo / Kabinda / -1935 / P. Henrard (RMCA). • Museé du Congo / Lulua: Tshibalaka / 11-X-1933 / F.G. Overlaet (RMCA). • Museé du Congo / Lulua: Kapanga / IX-1932 / F.G. Overlaet (RMCA). • Museé du Congo / Lulua: Kapanga / X-1932 / G.F. Overlaet (RMCA). • Museé du Congo / Lulua: Kapanga / IX-1932 / G.F. Overlaet (RMCA). • Museé du Congo / Lulua: Tshibalaka / X-1933 / F.G. Overlaet (RMCA). • [2x] Coll. Mus. Congo / Katanga: Katompe / II-1935 / Ch. Seydel; Katompe / Février. 235 / Ch. Seydel (RMCA). • Museé du Congo / Lulua: R. Lulua / 26-IX-1933 / G.F. Overlaet (RMCA). • [6x] Museé du Congo / Lomami: champ / de coton –IX.1936 / 14.T. / P. Hernard (RMCA). • Musée du Congo / Maniema: / -1936 / P. Henrard (RMCA). • Museé du Congo / Sandoa / XII-1931 / G.F. Overlaet (RMCA). • Musée du Congo / Kasongo / -IX-1936 / P. Henrard (RMCA). • Museé du Congo / Lomami: champ / de coton .IX.1936 / 14.T. / P. Hernard; 14T (RMCA). • [4x] Museé du Congo / Lulua: Kapanga / XI.1932 / F.G. Overlaet (RMCA). • Musée du Congo / Kivu: Katana / X-1932 / L. Burgeon (RMCA). • Coll. Mus. Congo / Kaniama / -1931 / R. Massart (RMCA). • Museé du Congo / Lomami: champ / de coton .IX.1936 / 14.T. / P. Hernard; raliohs (RMCA). • Museé du Congo / Lac Tanganyka : / Nyanza 6º I-1933 / L. Burgeon (RMCA). • [2x] Museé du Congo / Lac Tanganyka : / Nyanza -28-XII-1932 / L. Burgeon (RMCA). • [2x] Museé du Congo / Lulua: Sandoa / -VI-1932 / F.G. Overlaet (RMCA). • Museé du Congo / Lac Tanganyka : / Nyanza finXII-1932 / L. Burgeon (RMCA). • Museé du Congo / Kapanga / -XI.1932 / F.G. Overlaet (RMCA). • Museé du Congo / Kapanga / -XII.1932 / G.F. Overlaet (RMCA). • Musée du Congo / Kasongo / IX-1936 / P. Henrard (RMCA). • Musée du Congo / Kasongo / IX-1936 / P. Henrard; s/cotonnier (RMCA). • Coll. Mus. Congo / Tanganika: Albertville / /III-1955 / H. Bomans (RMCA). • Congo belge: P.N.U. / Mabwe (lac Upemba) / (585 m.) 1-12-VIII-1947 / Mis. G.F. de Witte. 660a; Coll. Mus. Tervuren; Phonoctonus / grandis / Signoret / A. Villiers det., 193 (RMCA). • Congo belge: P.N.U. / Mabwe (lac Upemba) / (585 m.) 21-28-VIII-1947 / Mis. G.F. de Witte. 719a; Coll. Mus. Tervuren; A. Villiers det., 1954: / Phonoctonus / grandis Signoret. (RMCA). • Coll. Mus. Tervuren / Kabalo / 1952 / Cotonco; Phonoctonus / grandis / Sign. / A. Villiers det 1957 (RMCA). • Congo belge: P.N.U. / Mabwe (lac Upemba) / (585 m.) 1-12-VIII-1947 / Mis. G.F. de Witte. 660a; Coll. Mus. Tervuren; A. Villiers det., 1954: / Phonoctonus / grandis Signoret. (RMCA). • Congo belge: P.N.U. / Mabwe (lac Upemba) / (585 m.) 21-23-VIII-1947 / Mis. G.F. de Witte. 716a; Coll. Mus. Tervuren; Phonoctonus / grandis / Signoret / A. Villiers det., 1931 (RMCA). • Museé du Congo / Afr. orien. ang. / Mombasa / Coll. Schouteden; Brit. africa / Mombasa; R. Dét. / P / 2580 (RMCA). • S. Rhodesia: / Odzi dist / 28.V.48 / N.C.E. Miller.; 165.; Phonoctonus / nigrofascialis / Stål; N.C.E. Miller det. 1955; Mus. Zool. Helsinki / Loan No / HE 2013 - 29 (MZH). • Tanganjika / Bez. Lindi

Ndanda / 300 m 7-8.XII.1958 / leg. C. Lindemann; Phonocotnus / grandis / Sign. / A. Villiers det. 195; Zoologische / Staatssammlung / München (ZSM). • Tanganjika / Usambara - mts / Mamboyaau / Mazindi / 6.XI.1962 / N. Pavlitzki leg; Zoologische / Staatssammlung / München (ZSM). • Tanganjika / Dar ed Salaam / 9.IX.63; Zoologische / Staatssammlung / München (ZSM). • Tanganjika / Dar ed Salaam / 14.9.61; Zoologische / Staatssammlung / München (ZSM). • Botswana mer. / Gaborone / IX-X.2000 / M. Hàiek lat. (MMBC). • Afrique Or. Allemande / Tanga / Alluand R Jeannel / Avril 1912 St. 74; Phonoctonus principalis Gerst. / R. Jeannel det. (MNHN). • Museum Paris / Moçambique / Vallée du Pungoué / Guengére / G. Vasse 1906 (MNHM). • [3x] Zanzibar (MNHN). • Côte d'Afrique or. angl. / TIWI / Alluand R. Jeannel / Nov 1911 St. 5 (MNHN). • Tanganijika / Bez. Lindi, Ndanda / 300 m 7-8 XII 1958 / leg. C. Lindemann (MNHN). • Museum Paris / Zambeze / Nova Choupanga / Pres Chemba / P. Lesne 1929; MAI (MNHN). • Tanganijika / Lindi, Ndanda / 300 m 3-8 VIII 1952 / Leg. Lindemann / und Parlitzki / Zoolog. / Staatsslg.; Muséum Paris / A. Villiers; Phonoctonus / spec. / Wygodzinsky det. 19 (MNHN). • Museum Paris / Zambéze / Tambare / P. Lesne 1929 (MNHN). • Museum Paris / Zambéze / Nova Choupanga / Pres Chemba / P. Lesne 1929 (MNHN). • Museum Paris / Zambeze / Nova Choupanga / Pres Chemba / P. Lesne 1929 (MNHN). • Congo Belge: P.W.U. / Mabwe (lac Upemba) / (585 m.) 1-12-VIII-1947 / Mis. G.F. de Witte 660a; Muséum Paris / don / A.Villiers (MNHN). • Maniema: Lusu / 1957 / Cotonco (MNHN). • Museum Paris / Zambéze / Nova Choupanga / Pres Chemba / P. Lesne 1929 (MNHN). • Museum Paris / ~~Kivu~~ Congo Belge / Kivou / Ouvira / Guy Babault 1927 (MNHN). • Museum Paris / Zambéze / Chiramba / P. Lesne 1929 (MNHN). • Museum Paris / Tanga (Afr. fr.) / Gierra 177-85 (MNHN). • Côte d'Afrique or angl. / Tiwi / Alluand R Jeannel / Nov. 1911 St. 5 (MNHN). • Museum Paris / Congo / Dybowski 128-96 (MNHN). • Zone Inférieure / Neu-Moschi / 800- / Avril 1912 St. 72; Afrique or. allemande / Kilimandjaro / Versant sud-est / Alluand R Jeannel (MNHN). • Museum Paris / Afrique Orient. / Portugaise / Nova Chupanga / P. Lesne 1928 (MNHN). • [2x] Angola: Luanda, Belas / 1969 / Mme Girandet (MNHN). • Museum Paris / Zambéze / Env. de Chemba / Bas Sangadré / P. Lesne 1929; 2 avril (MNHN). • Tang. Terr. / Ukerewe I. ? / Father Conrads; 283; Kenya Natl. / Mus. exchange (USNM). • Eigoma / Tanganyika Rep. / Sept-Nov. 1960 / Miss.Good…; Phonoctonus / Nigrofasci- / atus St.; Kenya Natl. / Mus. exchange (USNM). • [3x] Coll. R. I. Sc. N. B. / Congo Belge / Kibangula / 20/24-IX-1958 / L. Henry (USNM). • Coll. R. I. Sc. N. B. / Congo Belge / Kibangula / V-1956 / L. Henry (USNM). • Coll. R. I. Sc. N. B. / Congo Belge / Kapiri / II-1946 / R. P. De Caters (USNM). • Congo Belge / Kibangula / II-1955 / L. Henry; R. I. Sc. N. B. / I. G. 20.520 (USNM). • Congo Belge / Nyunzu / Kabeya-Mulonga / 1955-L. Henry; Coll. I. R. Sc. N. B. (USNM).
